# Supplementary material for: A general strategy for heterogenizing olefin polymerization catalysts and the synthesis of polyolefins and composites
Source: Nat Commun. 2022 Apr 12;13:1954. doi: 10.1038/s41467-022-29533-9 (PMC9005542; doi:10.1038/s41467-022-29533-9)
Supplement: Supplementary file 1 — Supplementary Information [file 41467_2022_29533_MOESM1_ESM.pdf]

Supplementary Information

A General Strategy for Heterogenizing Olefin Polymerization Catalysts and the Synthesis of Polyolefins and Composites

Chen Zou, Guifu Si, Changle Chen*

1. Supplementary Tables and Figures 2

2. Supplementary Methods 12

3. Supplementary Figures of Characterization of ligands and homogeneous catalysts 17

4. Supplementary Figures of ^{1}H NMR and ^{13}C NMR of copolymers 28

5. Supplementary Figures of DSC of copolymers 43

6. Supplementary Figures of GPC of copolymers 78

7. Supplementary References 120

1. Supplementary Tables and Figures

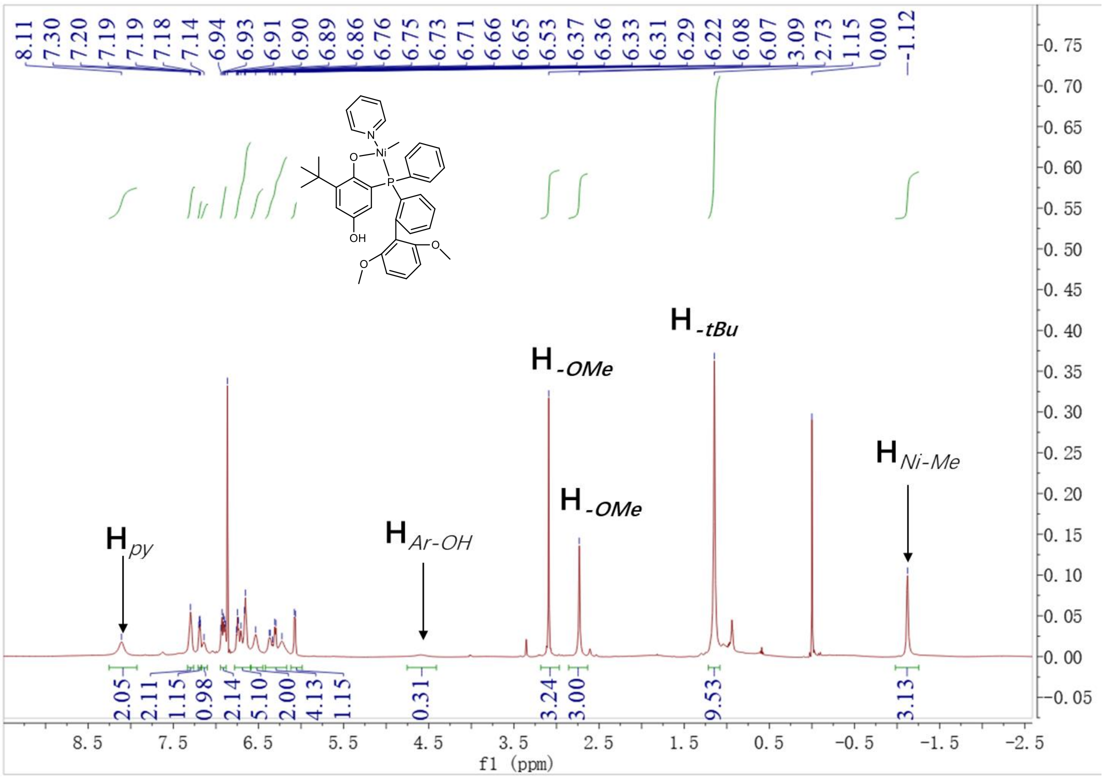

Supplementary Figure 1. Coexistence of Ni-Me and Ar-OH signals in **Ni-OH** in ^{1}H NMR analysis.

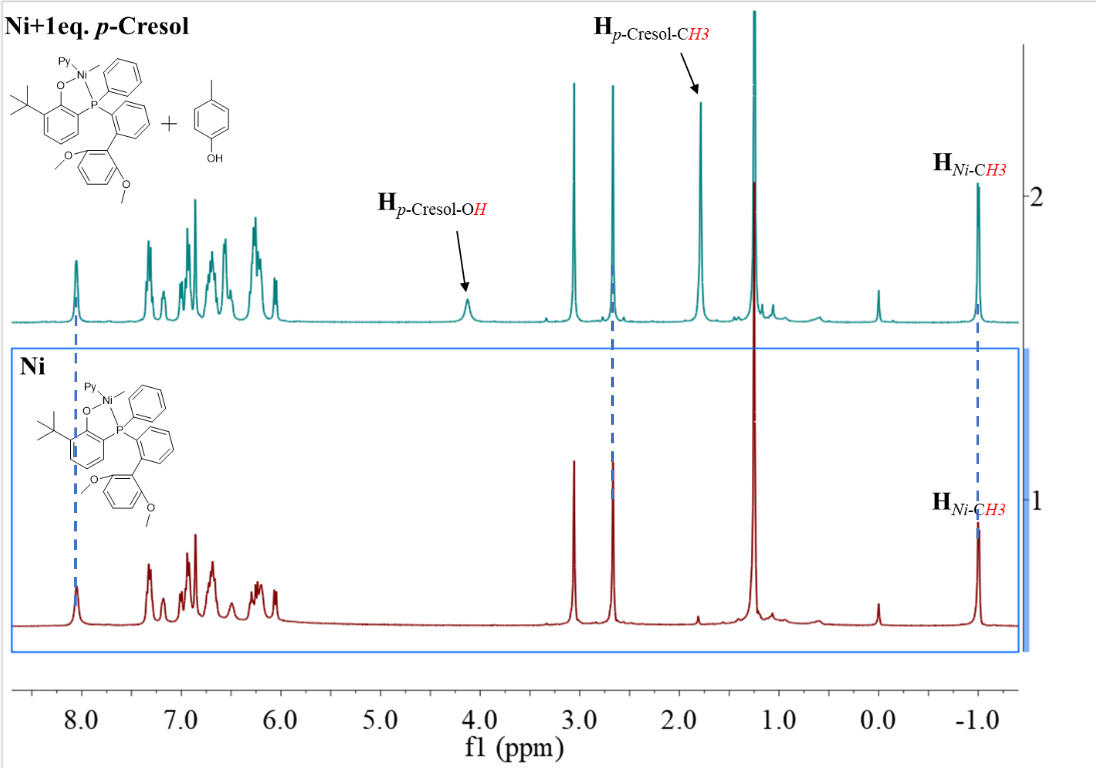

Supplementary Figure 2. ^{1}H NMR analysis showing no reaction between p-cresol and **Ni**.

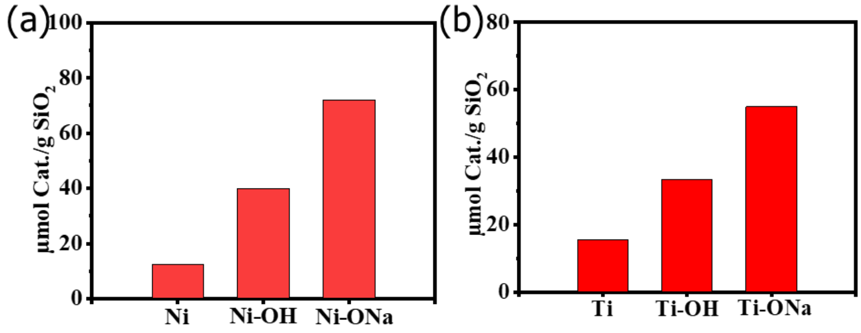

Supplementary Figure 3. The maximum adsorption capacity of different metal complexes on SiO_2. (a) The maximum adsorption capacity of catalysts **Ni**, **Ni-OH** and **Ni-ONa** on SiO_2. (b) The maximum adsorption capacity of catalysts **Ti**, **Ti-OH** and **Ti-ONa** on SiO_2.

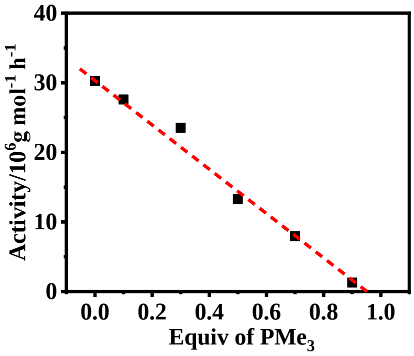

Supplementary Figure 4. Plot of equiv. of PMe_3 vs activity.

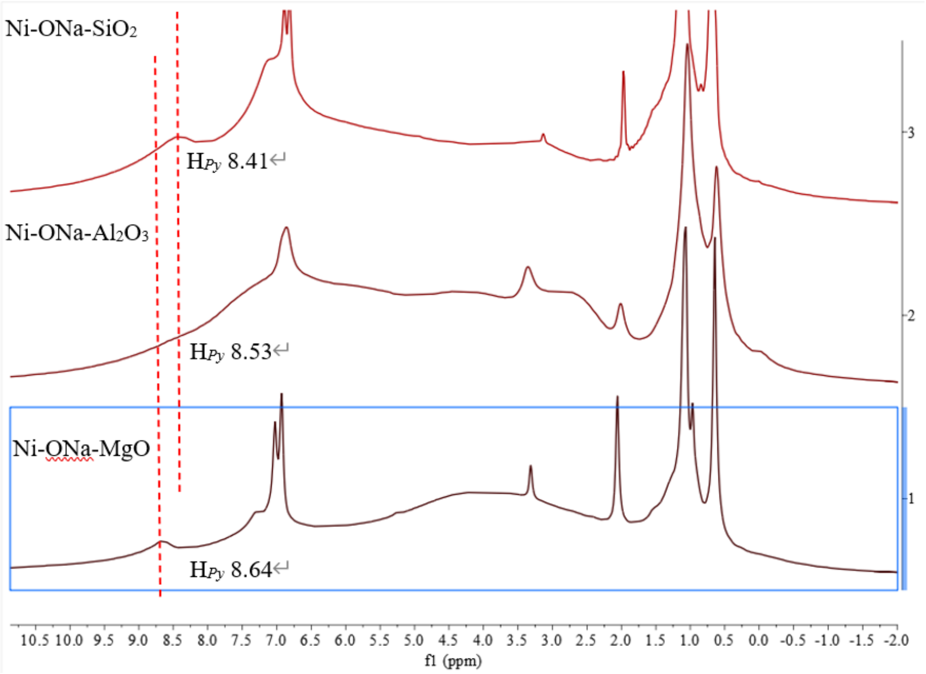

Supplementary Figure 5. ^{1}H MAS NMR spectrum of supported catalysts **Ni-ONa-SiO_2, Ni-ONa-Al_2O_3** and **Ni-ONa-MgO**.

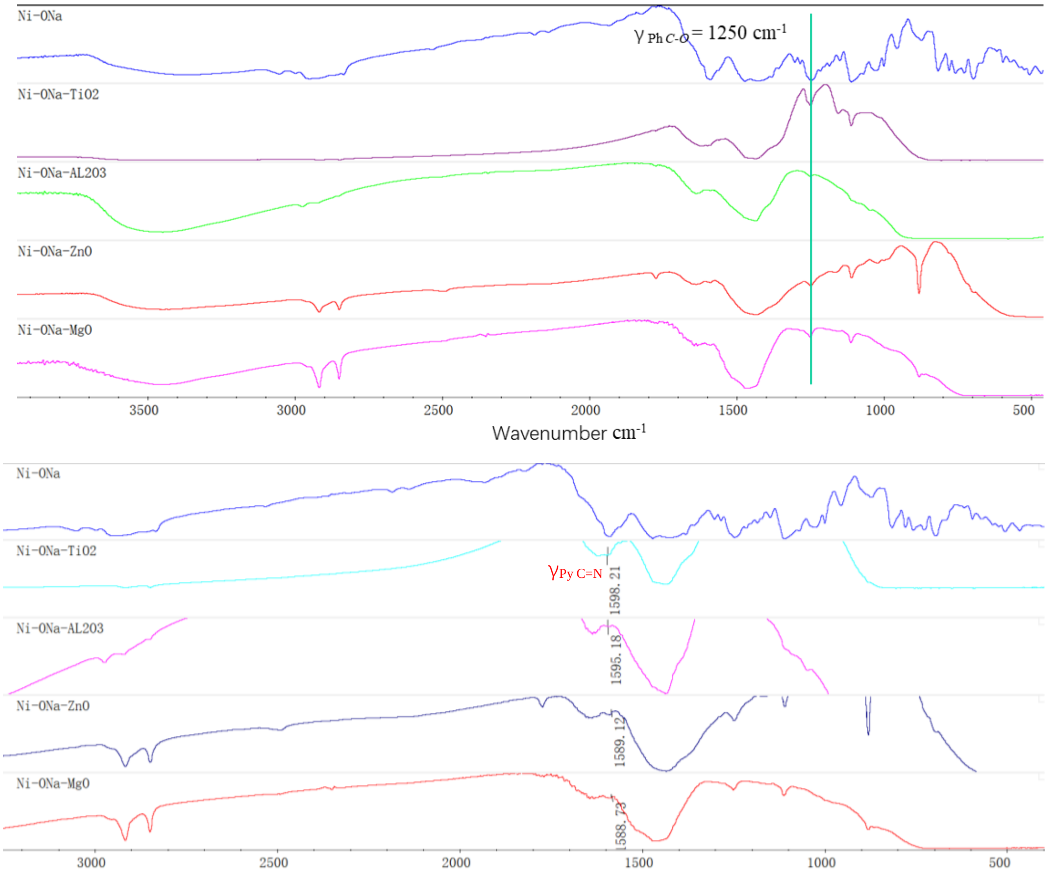

Supplementary Figure 6. IR spectra of Ni-based supported catalysts. The C=N stretching peak of **Ni-ONa-TiO$_2$**, **Ni-ONa-Al$_2$O$_3$**, **Ni-ONa-ZnO** and **Ni-ONa-MgO** are at 1598.21 cm^{-1}, 1595.18 cm^{-1}, 1589.12 cm^{-1} and 1588.73 cm^{-1}, respectively. The wavenumber of the C=N stretching peak decreases slightly with the increase of the alkalinity of the support, indicating that the electron density of the metal center increases with the alkalinity of the support.

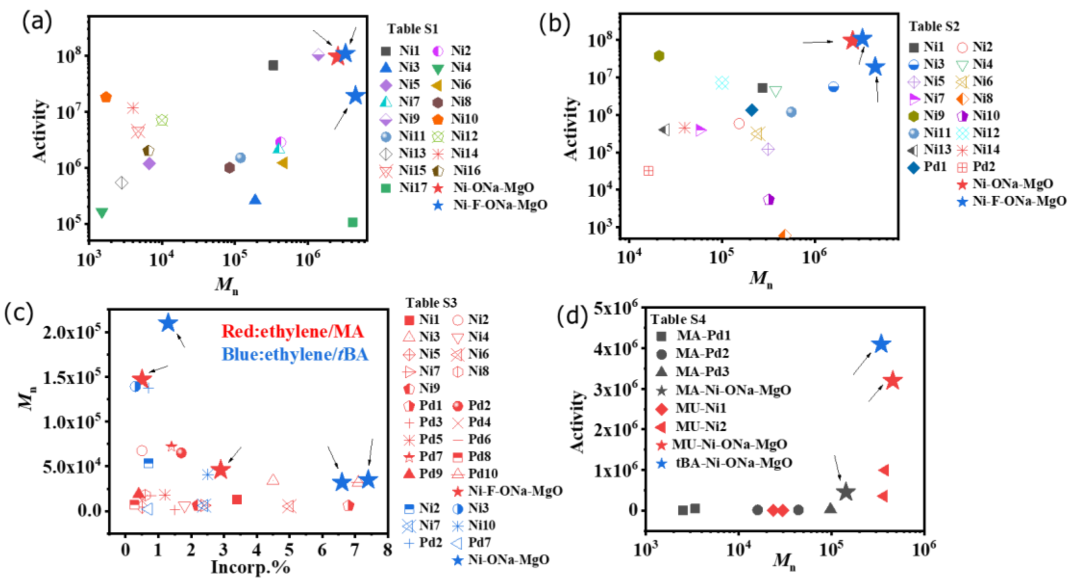

Supplementary Figure 7. (a) Comparison of activity and polymer molecular weight between representative reported homogeneous nickel catalysts and **Ni-ONa-MgO**/**Ni-F-ONa-MgO**. (b) Comparison of activity and polymer molecular weight between representative reported heterogeneous nickel and palladium catalysts and **Ni-ONa-MgO**/**Ni-F-ONa-MgO**. (c) Comparison of polymer molecular weight and comonomer incorporation between representative reported homogeneous nickel/palladium catalysts and **Ni-ONa-MgO**/**Ni-F-ONa-MgO** in the copolymerization of ethylene with methyl acrylate or *tert*-butyl acrylate. (d) Comparison of polymer molecular weight and comonomer incorporation between representative reported heterogeneous nickel/palladium catalysts and **Ni-ONa-MgO**/**Ni-F-ONa-MgO** in the copolymerization of ethylene with methyl acrylate, *tert*-butyl acrylate or 10-methyl undecanoate.

Supplementary Table 1. Comparison of activity and polymer molecular weight between representative reported homogeneous nickel catalysts and Ni-ONa-MgO/Ni-F-ONa-MgO.

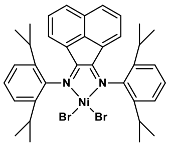

Ni1

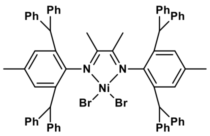

Ni2

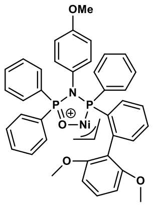

Ni3

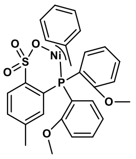

Ni4

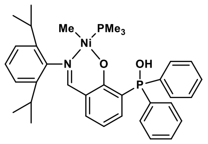

Ni5

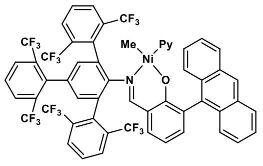

Ni6

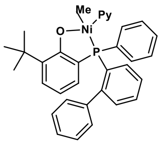

Ni7

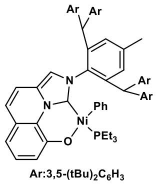

Ni8

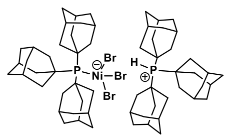

Ni9

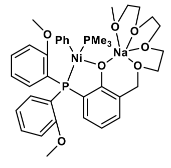

N10

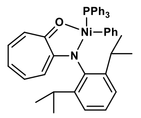

Ni11

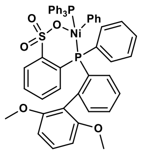

Ni12

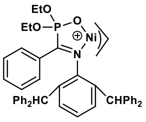

Ni13

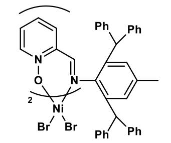

Ni14

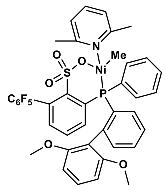

N15

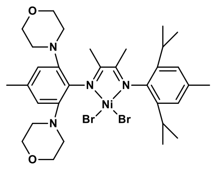

Ni16

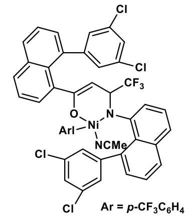

Ni17

Cat.	P/atm	T/°C	t/min	Act. *(10³)*	M_n	M_w/M_n	Reference
**Ni1** (0.83 μmol in 200 mL)	13.6	35	10	67200	337000	1.8	Brookhart (**4g**)[1]
**Ni2** (1.57 μmol in 100 mL)	6.8	100	10	2856	422000	1.2	Long (**2b**)[2]
**Ni3** (5.00 μmol in 20 mL)	8	25	60	260	188900	2.5	Chen (**Ni4**)[3]
**Ni4** (20.0 μmol in 30 mL)	20	25	120	163	1500		Jordan (**4a**)[4]
**Ni5** (10.0 μmol in 25 mL)	8	25	40	1184	6700	1.8	Marks (**1b**)[5]
**Ni6** (5.00 μmol in 100 mL)	40	30	40	1218	466100	1.6	Mecking (**2-CF3/Py**)[6]
**Ni7**(5.00 μmol in 100 mL)	10	30	20	2100	398000	1.5	Li (**2c**)[7]
**Ni8** (2.5 μmol in 8.5 mL)	40	30	30	1000	84000	2.0	Nozaki (**7c**)[8]
**Ni9** (0.50 μmol in 150 mL)	27.2	10	3.5	103600	1390000	1.4	Daugulis/Brookhart (**6**)[9]
**Ni10** (0.50 μmol in 100 mL)	30	30	60	18100	1710	1.5	Loi H. Do [10]

**Ni11** (7.6 µmol in 200 mL)	27.2	80	60	1500	119000	1.8	Brookhart (**3**)[11]
**Ni12** (10 µmol in 80 mL)	27.2	90	20	7028	10000	2.2	Scott (**3b**)[12]
**Ni13** (10 µmol in 20 mL)	8	60	60	540	2800	2.4	Chen (**Ni2-Ar***)[13]
**Ni14** (1 µmol in 20 mL)	8	20	30	11700	4000	2.2	Chen (**Ni-Ph**)[14]
**Ni15** (2 µmol in 50 mL)	8	80	30	4500	4700	2.7	Chen (**4**)[15]
**Ni16** (2 µmol in 50 mL)	8	RT	180	2000	6500	2.6	Chen (**NO-*i*Pr-Ni**) [16]
**Ni17** (2 µmol in 50 mL)	27.2	25	240	107	4100000	1.16	Daugulis(2f)[17]
**Ni-ONa-MgO**	30	80	10	96600	256.9	2.5	This work
**Ni-F-ONa-MgO**	30	80	10	109200	326.5	2.7	This work
**Ni-F-ONa-MgO**	30	30	10	19200	449.2	2.0	This work

Supplementary Table 2. Comparison of activity and polymer molecular weight between representative reported heterogeneous nickel and palladium catalysts and Ni-ONa-MgO/Ni-F-ONa-MgO.

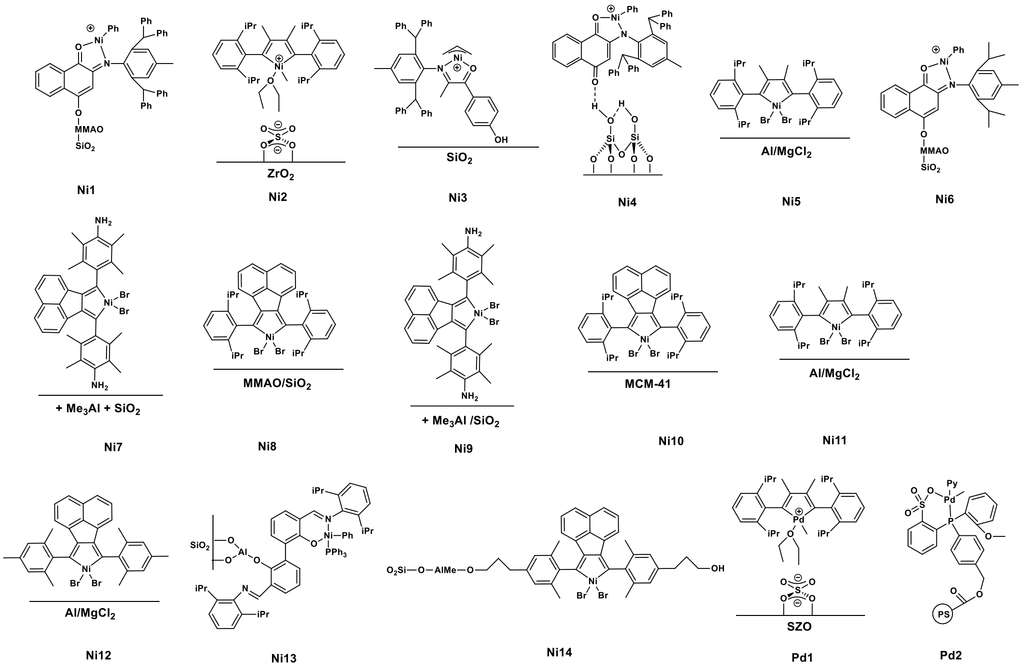

Cat.	P/atm	T/°C	t/min	Act. (10^3)	M_n	M_w/M_n	Reference
**Ni1** (5 µmol in 30 mL)	10	40	5	5200	273000	2.55	Cai (**1**)[18]
**Ni2** (1.4 µmol in 20mL)	3	40	15	568	153000	1.8	Conley, M. P. (**2**)[19]
**Ni3** (1µmol in 30 mL)	15	50	30	5600	1593000	3.0	Chen (**Ni–OH@SiO2**)[20]
**Ni4** (5µmol in 30 mL)	10	40	10	4580	380000	2.59	Cai (**Ni/SiO$_2$**)[21]
**Ni5** (in 500 mL)	1	25	60	120	313600	2.87	Mao (SPC-1)[22]

**Ni6** (10 µmol in 30 mL)	1	40		312	243000	7.42	Shiono (**1**)[23]
**Ni7** (0.50 µmol in 150 mL)		60	130	390	57360	3.8	Brookhart (**4**)[24]
**Ni8** (30mg in 400 mL)	13.6	30	30	0.592	477000	2.41	Zhu[25]
**Ni9** (6mg)	10	60	15	37800	21000	6.72	Soares (**3**)[26]
**Ni10** (17 mg in 400 mL)	13.6	35	60	5400	320000	2.8	Zhu (**MCM-Ni-2**)[27]
**Ni11**(in 500 mL)	5	50	60	1190	560000	2.3	Chadwick (**1**)[28]
**Ni12** (in 500 mL)	5	50	60	7158	100000	2.9	Chadwick (**4**)[28]
**Ni13** (in 60 mL)	21	40	60	400	24500	4.93	Li (**SC-8**)[29]
**Ni14** (in 100 mL)	10	60	120	450	39600	3.3	Brookhart (**1b1**)[30]
**Pd1 (**0.07 µmol in 5mL)	10	40	60	1342	210000	1.6	Conley, M. P. (**4**)[31]
**Pd2** (18 µmol in 100 mL)	10	90	450	31.6	16100		Mecking (**PS (18)-3-pyr**)[32]
**Ni-ONa-MgO**	30	80	10	96600	256.9	2.5	This work Table 1
**Ni-F-ONa-MgO**	30	80	10	109200	326.5	2.7	This work Table 1
**Ni-F-ONa-MgO**	30	30	10	19200	449.2	2.0	This work Table 1

Supplementary Table 3. Comparison of polymer molecular weight and comonomer incorporation between representative reported homogeneous nickel/palladium catalysts and Ni-ONa-MgO/Ni-F-ONa-MgO in the copolymerization of ethylene with methyl acrylate or *tert*-butyl acrylate.

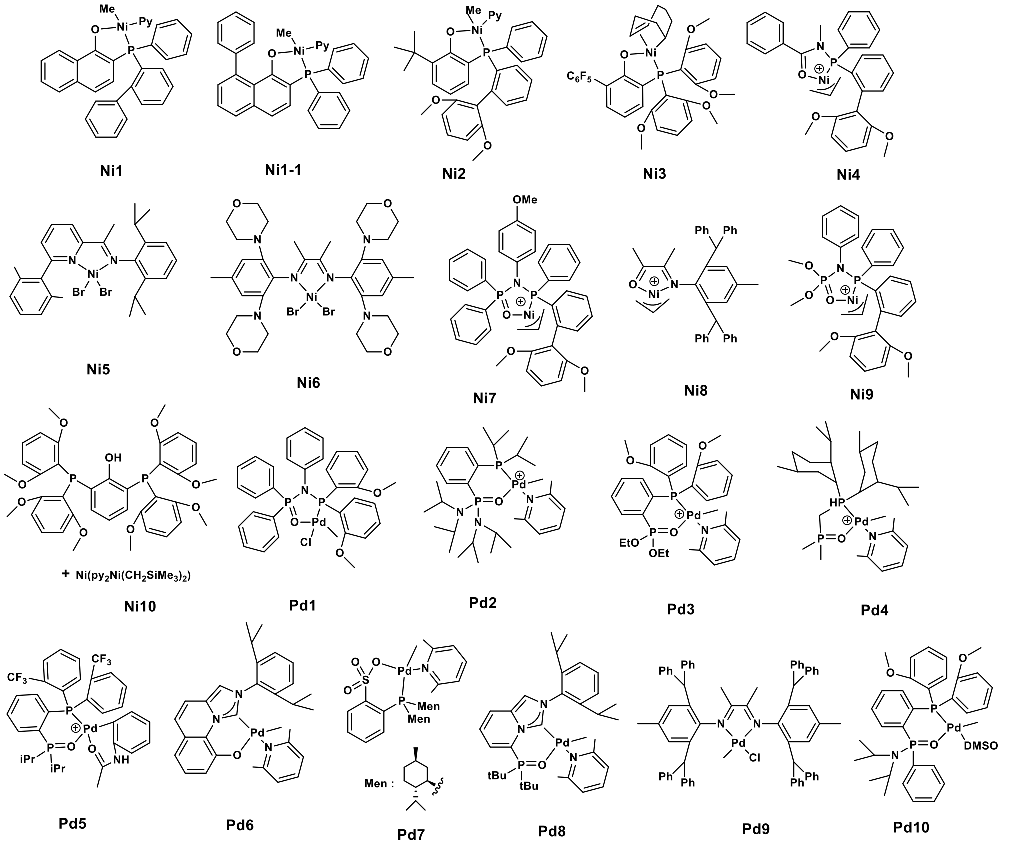

Cat.	comonomer	P/atm	T/°C	t/min	Act. (10^3)	incorp. (mol %)	M_n	M_w/M_n	Reference
**Ni1** (10 µmol in 250 mL)	MA	15	70	60	237.8	3.4	12900	1.7	Li (**2a”**)[33]
**Ni1-1** (10 µmol in 250 mL)	MA	15	70	60	404.7	2.7	666	1.8	Li (**2b**)[33]
**Ni2** (10 µmol in 50 mL)	MA	10	30	60	37	0.5	67500	1.6	Li (**2b**)[34]
**Ni3**(80 µmol in 1000 mL)	MA	30	70	60	86	4.5	34000	2.0	Shimizu (**2b**)[35]
**Ni4** (20 µmol in 20 mL)	MA	8	80	360	8.3	1.8	6000	2.34	Jian (**Ni4**)[36]
**Ni5** (10 µmol in 50 mL)	MA	6	40	240	15	0.5	4500	1.6	Claudio (**Ni1**)[37]
**Ni6**(10 µmol in 50 mL)	MA	8	R.T.	360	1	5.0	5500	2.12	Chen (**NO-Ni**)[16]
**Ni7** (20 µmol in 20 mL)	MA	8	80	360	9.2	2.4	6400	2.3	Chen (**Ni4**)[3]
**Ni8** (20 µmol in 20 mL)	MA	8	50	120	2.5	0.6	17900	2.0	Chen (**Ni2**)[20]
**Ni9** (20 µmol in 20 mL)	MA	8	80	360	10.1	2.2	6600	2.2	Tan (**Ni4**) [38]
**Pd1** (20 µmol in 20 mL)	MA	8	80	360	16.7	6.8	6100	2.5	Chen (**Pd1**)[3]
**Pd2** (15 µmol in 300 mL)	MA	30	90		740	1.7	65000	1.3	Carrow (**3b**)[39]
**Pd 3** (15.2 µmol in 50 mL)	MA	28	80		58	1.5	1500	2.36	Jordan (**3b**)[40]
**Pd4** (10 µmol in 15 mL)	MA	30	80	15h	20	0.5	12000	2.1	Nozaki. (**3f**)[41]
**Pd 5** (0.75 µmol in 15 mL)	MA	30	80	60	41	1.2	18000	2.3	Nozaki. (**2e**)[42]
**Pd 6** (15 µmol in 20 mL)	MA	40	100	180	10.6	0.8	17000	2.0	Nozaki. (**5a**)[43]
**Pd 7**(10 µmol in 15 mL)	MA	30	80	180	67	1.4	72000	2.5	Nozaki. (**1f**)[44]
**Pd 8** (5µmol)	MA	30	30	180	4.8	0.27	7000	2.3	Nozaki. (**4b**)[45]
**Pd 9**(10 µmol in 25 mL)	MA	1	20	15h	3.7	0.4	18900	1.78	Chen (**5**) [46]
**Pd 10** (20 µmol in 50 mL)	MA	9	100	60	14	7.1	32000	1.66	Chen (**3b**) [47]
**Ni2** (10 µmol in 50 mL)	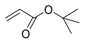	10	50	60	53.6	0.7	53000	1.6	Li (**2b**)[34]
**Ni3** (80 µmol in 1000 mL)	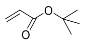	30	70	20	3230	0.3	139200	2.5	Shimizu (**2f**)[35]
**Ni7** (20 µmol	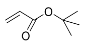	8	80	360	9.2	2.4	6400	2.3	Chen (**Ni4**)[3]

in 20 mL)									
**Ni10** (0.25 μmol in 5 mL)	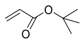	27	70	30	321	2.51	40900	2.3	Agapie (POPH+**Ni**)[48]
**Ni10** (0.25 μmol in 5 mL)	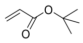	27	100	30	82	11.95	8680	2.2	Agapie (POPH+**Ni**)[48]
**Pd2** (15 μmol in 300 mL)	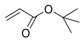	30	90		1000	0.7	13750 0	1.6	Carrow (**3b**)[39]
**Pd 7** (15 μmol in 20 mL)	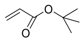	40	100	180	7.2	0.7	2300	3.2	Nozaki. (**5a**)[43]
**Ni-F-ONa-MgO**	MA	30	80	30	456	0.5	14700 0	2.9	This work Table 2
**Ni-F-ONa-MgO**	MA	8	80	30	124	2.9	46000	2.1	This work Table 2
**Ni-F-ONa-MgO**	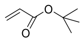	30	80	30	4100	0.3	34300 0	4.0	This work Table 2
**Ni-ONa-MgO**	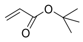	30	80	30	1660	0. 1	83400 0	3.0	This work Table 2
**Ni-ONa-MgO**	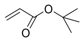	20	120	60	1300	1.3	21000 0	2.9	This work Table 4
**Ni-ONa-MgO**	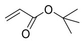	8	120	30	132	7.4	35000	2.5	This work Table 4
**Ni-ONa-MgO**	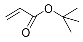	8	140	30	156	6.6	32000	2.3	This work Table 4

Supplementary Table 4. Comparison of polymer molecular weight and comonomer incorporation between representative reported heterogeneous nickel/palladium catalysts and Ni-ONa-MgO/Ni-F-ONa-MgO in the copolymerization of ethylene with methyl acrylate, *tert*-butyl acrylate or 10-methyl undecanoate.

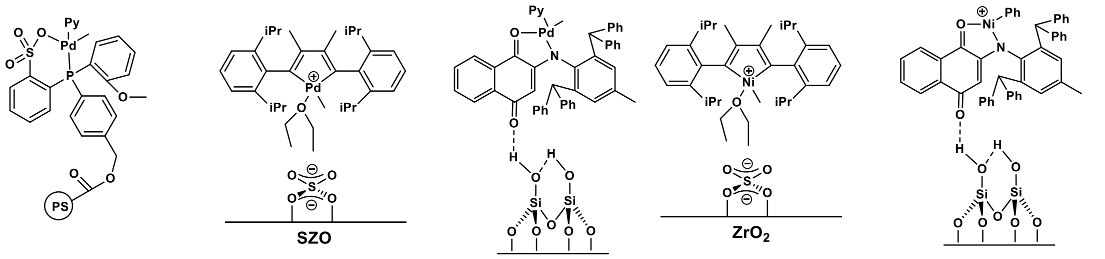

Pd1 **Pd2** **Pd3** **Ni1** **Ni2**

Cat.	comonomer	P/ atm	T/ ºC	t/ min	Act. (10^3)	Incorp. mol %	M_n	M_w/M_n	Reference
**Pd1** (18 μmol active Pd in 100 mL)	MA	10	90	10	46	2.2	3400		Mecking (**PS(18)-3-pyr**)[32]
**Pd1-1** (18 μmol active Pd in 100 mL)	MA	10	90	90	6.8	2.7	2500		Mecking (**PS(18)-3-pyr**)[32]
**Pd2** (0.35 μmol active Pd)	MA	5.4	40	900	14.9	0.33	4400	1.9	Conley, M. P. (**4**)[31]

**Pd2-1** (0.35 µmol active Pd)	MA	5.4	60	900	14.9	0.46	1600	2.3	Conley, M. P. (**4**)[31]
**Pd3** (10 µmol in 30 mL)	MA	20	40	60	21	0.7	98000	3.96	Cai (**Pd/SiO₂**)[21]
**Ni1**	$(\,)_8$COOMe	3	40		5.1	0.3	29700	5.17	Conley, M. P. (**2**)[19]
**Ni1-1**	$(\,)_8$COOMe	3	60		4.0	0.4	23600	4.15	Conley, M. P. (**2**)[19]
**Ni2** (5 µmol in 30 mL)	$(\,)_8$COOMe	10	40	10	996	0.4	378000	2.47	Cai (**Ni/SiO₂**)[21]
**Ni2-1** (5 µmol in 30 mL)	$(\,)_8$COOMe	3	40	30	356	0.7	366000	2.67	Cai (**Ni/SiO₂**)[21]
**Ni-ONa-MgO**	$(\,)_8$COOMe	20	120	30	3200	0.4	456000	2.9	This work Table 4

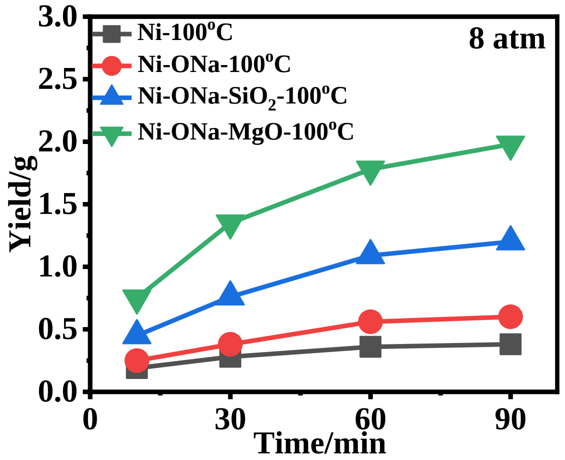

Supplementary Figure 8. Time-dependence studies (yield versus time) of the nickel catalysts (0.1 µmol) at 100 °C, 8 atm.

Supplementary Table 5. Ethylene polymerization studies with the Ni catalysts supported on different fillers.[a]

Ent.	Cat.	Yied[b]/g	Act. [b](10^6)	T_m[c]/°C	M_n [d](10^6)	M_w/M_n[d]
1	Ni-ONa-GR	1.03	6.18	133.1	31.2	4.6
2	Ni-ONa-EG	3.22	19.32	133.1	42.9	5.9
3	Ni-ONa-GF	3.96	23.76	135.3	17.3	4.8
4	Ni-ONa-APP	4.57	27.42	136.0	38.9	8.3

[a] Conditions: catalyst 1 µmol; 30 mL *n*-heptane; t = 10min; [b]Polymer yield and activity values are average of at least two runs. Activity = 10^6 g·mol^{-1}·h^{-1}. [c]Determined by DSC. [e]M_n: 10^4 g mol^{-1}, [d]M_n and M_w/M_n determined by GPC in trichlorobenzene at 160 °C. GR, EG, GF and APP represent Graphene, Expanded graphite, Glass fiber and Ammonium polyphosphate, respectively.

Supplementary Table 6. Relative Data of Cone Calorimeter for HDPE, HDPE-EG-10 and Ni-EG-10

sample	TTI/s	Time to PHRR/s	PHRR /KW/m^2	THR (MJ/m^2)	FGI (kW/m^{2}s)
HDPE	79	206	745.3	131.6	3.6
HDPE-EG-10	91	94	563.7	130.0	6.0
Ni-EG-10	115	146	491.7	129.8	3.4

The heat release rate (HRR); total heat release (THR); the time to ignition(TTI); the time to peek heat release rate, the peak heat release rate (PHRR)

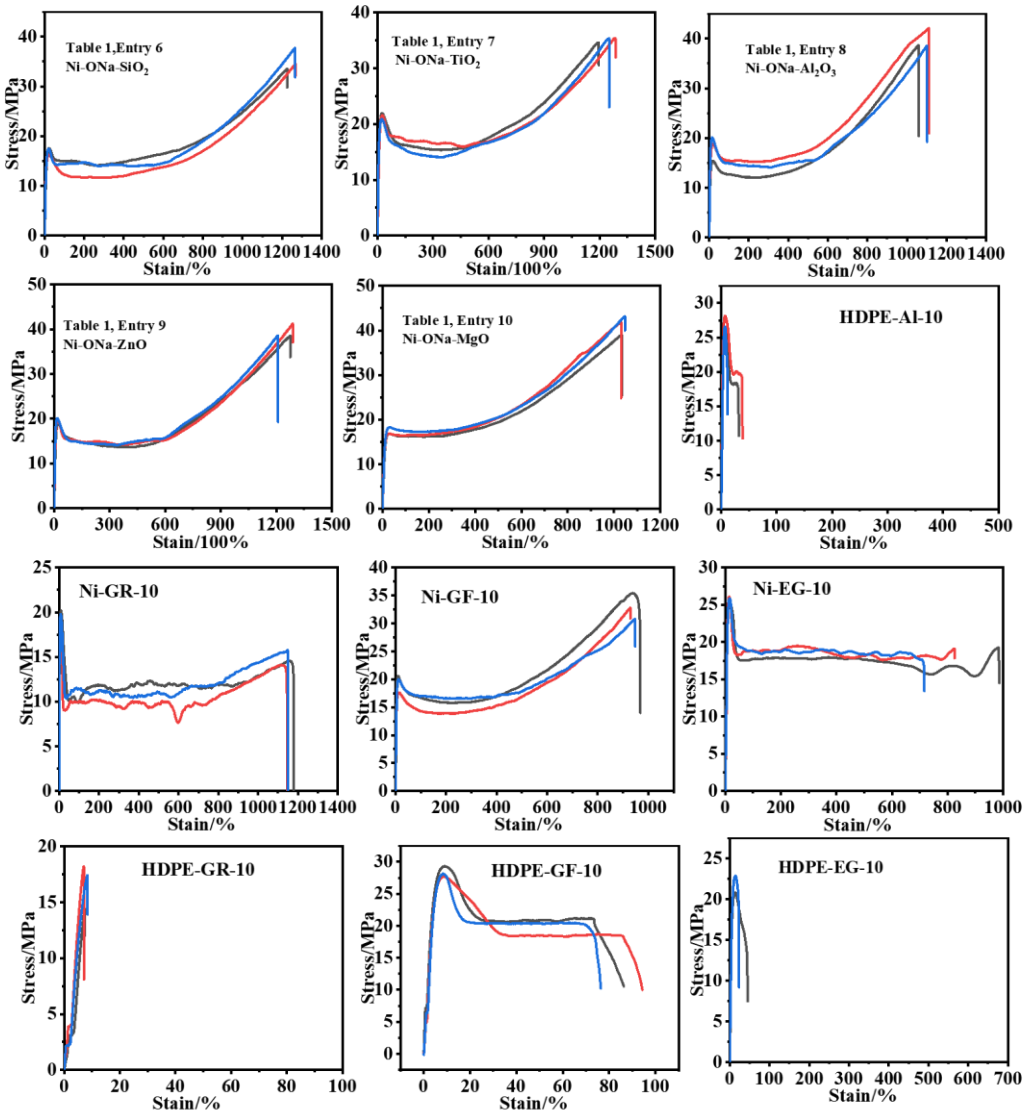

Supplementary Figure 9. Stress-Strain curve of polymers and composites.

2. Supplementary Methods

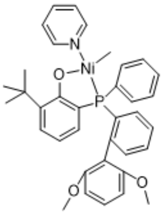

Ni

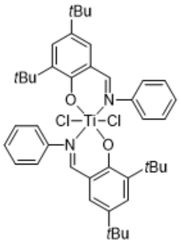

Ti

Ni: Under nitrogen, 2-(2-tert-butylphenoxy)-tetrahydro-2H-pyran (2.34 g, 10 mmol) was dissolved in 50 mL dried THF. n-BuLi (4.6 mL, 2.4 M in hexane, 11 mmol, 1.1 equiv.) was added dropwise and the reaction mixture was stirred for 2 h at 0 ^{o}C. The flask was transferred to a -78 ^{o}C bath, and a THF solution of chloro(2',6'-dimethoxy-[1,1'-biphenyl]-2-yl)(phenyl)phosphane (PhPArCl) (11 mmol,1.1 equiv) was added dropwise. The mixture was stirred for 1 hour, and warmed to room temperature to react for 12 hours. After quenching, the suspension was transferred to a round bottom flask and the THF was evaporated on a rotary evaporator. The crude product was extracted with DCM (3 x 200 mL), washed with H_2O (3 x 100 mL), and the organic phase was collected and dried over Na_2SO_4. After filtration and concentration, the crude product of the protected ligand can be obtained, which was used for the next reaction without further purification. The protected ligand was dissolved in methanol under nitrogen, and 3. eq of p-toluenesulfonic acid was added to react for 6 hours. The reaction solution was evaporated with a rotary evaporator to evaporate methanol. The crude material was extracted with DCM (3 x 200 mL), washed with H_2O (3 x 100 mL), and the organic phase was collected and dried over Na_2SO_4. After filtration and concentration, the pure ligand was obtained by column chromatography as a white solid (2.39 g, 51%). Under nitrogen, a toluene solution of 1 mmol (470 mg) ligand and 1.2 eq. Py_2NiMe_2 was stirred for 1 hour at room temperature. After concentration, the addition of n-hexane for recrystallization resulted in the formation of **Ni** (503 mg, 80%) as a yellow solid. The characterization results of **Ni** are consistent with those reported in literature.[34]

Ti: A mixture of 5 mmol 3,5-di-tert-butyl-2-hydroxybenzaldehyde, 5 mmol aniline and 1 mL formic acid in 150 mL of methanol was refluxed at 80 °C for 12 hours. After concentrating, the addition of n-hexane for recrystallization resulted in the formation of ligand as a yellow solid (1.35 g, 88%). Under nitrogen, a mixture of 1 mmol ligand and 1 eq. NaH was stirred in 30 mL toluene for 1 hour. 0.5 eq. $TiCl_4$ was added and reacted for another 1 hour. The solid was filtered and washed with toluene (20 mL) three times. The filtrate was concentrated, and addition of n-hexane for recrystallization resulted in the formation of catalyst **Ti** as a red solid (338mg,92%). The characterization results of

Ti are consistent with those reported in literature.[49]

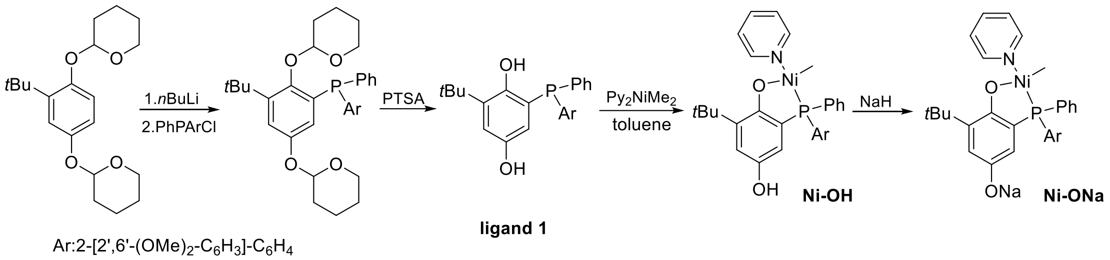

Ni-ONa: Under nitrogen, 2,2'-((2-(tert-butyl)-1,4-phenylene)bis(oxy))bis(tetrahydro-2H-pyran)(3.34g,10 mmol) was dissolved in 50 mL of dried THF. n-BuLi(4.6mL,2.4 M in hexane,11 mmol,1.1 equiv) was added dropwise and the reaction mixture was stirred for 2 h at 0 ^oC. The flask was transferred to a -78 ^oC bath, and the THF solution of PhPArCl (11 mmol,1.1 equiv) was added dropwise. The mixture was stirred for 1 hour, and warmed to room temperature to react for 12 hours. After quenching, the suspension was transferred to a round bottom flask and the THF was evaporated on a rotary evaporator. The crude material was then extracted with DCM (3 x 200 mL), washed with H_2O (3 x 100 mL), and then collect the organic phase and dried with Na_2SO_4. After filtration and concentration, the crude product of the protected ligand can be obtained, which can be directly used for the next reaction without further purification. Then, the protected ligand was dissolved in methanol under nitrogen, 3. eq of p-toluenesulfonic acid was added to react for 6 hours. Then, the reaction solution was evaporated with a rotary evaporator to evaporate methanol, the crude material was then extracted with DCM (3 x 200 mL), washed with H_2O (3 x 100 mL), and then collect the organic phase and dried with Na_2SO_4. After filtration and concentration, the pure **ligand 1** was obtained by column chromatography as a white solid (1.90 g, 39%). Under nitrogen, a toluene solution of 1 mmol (487 mg) of the ligand and 1.2 eq. Py_2NiMe_2 was stirred for 1 hour at room temperature. After concentrating, adding n-hexane for recrystallization resulted in the formation of **Ni-OH** (530 mg, 83%) as a yellow solid. Under nitrogen, add 1 eq. NaH to the toluene solution with 1 mmol **Ni-OH**, stir at room temperature for 1 hour, and drain the solvent to obtain **Ni-ONa** (610 mg, 92%). **ligand 1**:^{1}H NMR (600 MHz, Chloroform-*d*) δ 7.41 (dd, *J* = 7.5, 1.5 Hz,1H), 7.29-7.24(m, 5H), 7.24-7.16(m, 4H)6.76 (d, *J* = 3.0 Hz, 1H), 6.51 (dd, *J* = 8.3, 4.3 Hz, 2H), 6.24 (dd, *J* = 4.2, 3.0 Hz, 1H), 6.16 (d, *J* = 10.5 Hz, 1H), 4.41 (s, 1H), 3.51 (s, 3H), 3.45 (s, 3H), 1.32 (s, 9H).^{31}P NMR (243 MHz, $CDCl_3$) δ -37.22.^{13}C NMR (151 MHz, Chloroform-*d*) δ 157.75, 157.71, 152.29, 152.15, 148.32, 141.42, 141.21, 137.48, 135.97, 133.66, 133.54, 133.49, 131.14, 131.10, 129.60, 129.55, 128.46, 128.33, 128.29, 127.65, 122.03, 118.57, 117.37, 116.43, 103.92, 103.82, 55.66, 55.46, 34.93, 29.55. ESI-MS (m/z): $[M+H]^+$ Calcd for C30 H32 O4 P, 487.20327; Found: 487.20386. **Ni-OH**: ^{1}H NMR (600 MHz, C_6D_6) δ 8.11 (s, 2H), 7.30 (s, 2H), 7.23-7.09 (m, 1H), 6.91 (dt, *J* = 14.1, 6.6 Hz, 2H), 6.78 - 6.61 (m, 5H), 6.53 (s, 2H), 6.42 - 6.19 (m, 4H), 6.07 (d, *J* = 8.2 Hz, 1H), 3.09 (s, 3H), 2.73 (s, 3H), 1.15 (9, 3H), -1.12 (s, 3H, Ni-*Me*). ^{31}P NMR (243 MHz, C_6D_6) δ 22.62. ^{13}C NMR (151 MHz, C_6D_6) δ 159.51, 158.03, 150.50, 141.41, 141.30, 137.58, 136.71, 135.84, 134.82, 132.91, 132.35, 132.29, 129.48,

128.92, 128.04, 127.92, 127.76, 127.60, 126.33, 122.55, 119.90, 114.72, 103.08, 102.91, 54.66, 54.19, 34.85, 29.57, -14.72 (d, J = 36.6 Hz, Ni-*Me*). Anal. Calcd for C36H38NNiO4P: C, 67.73; H, 6.00; N, 2.19. Found: C, 67.52; H, 6.11; N, 2.25.

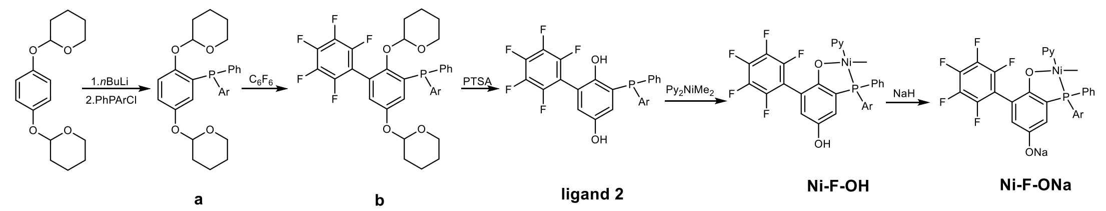

Ar:2-[2',6'-(OMe)$_2$-C_6H_3]-C_6H_4

Ni-F-ONa: Under nitrogen, 1,4-bis((tetrahydro-2H-pyran-2-yl)oxy)benzene (2.79 g,10 mmol) was dissolved in 50 mL dried THF. n-BuLi (4.6 mL,2.4 M in hexane,11 mmol, 1.1 equiv) was added dropwise and the reaction mixture was stirred for 2 h at 0 ^oC. The flask was transfer to a -78 ^oC bath, and a THF solution of PhPArCl (11 mmol,1.1 equiv) was added dropwise. The mixture was stirred for 1 hour, and warmed to room temperature to react for 12 hours. After the reaction was quenched and extracted, the crude product **a** was obtained. Without purification, the crude product **a** was dissolved in THF, and 1.1 eq. nBuLi was added at 0 °C. After 2 hours of reaction, hexafluorobenzene (33 mmol, 3.0 equiv) was added. After warming to room temperature, the reaction was carried out for 6 hours. After quenching, the suspension was transferred to a round bottom flask and the THF was evaporated on a rotary evaporator. The crude material was then extracted with DCM (3 x 200 mL), washed with H_2O (3 x 100 mL), and then collect the organic phase and dried with Na_2SO_4. After filtration and concentration, the crude product **b** can be obtained, which can be directly used for the next reaction without further purification. Then, the crude product **b** was dissolved in methanol under nitrogen, 3. eq of p-toluenesulfonic acid was added to react for 6 hours. Then, the reaction solution was evaporated with a rotary evaporator to evaporate methanol, the crude material was then extracted with DCM (3 x 200 mL), washed with H_2O (3 x 100 mL), and then collect the organic phase and dried with Na_2SO_4. After filtration and concentration, the pure **ligand 2** was obtained by column chromatography as a white solid (3.87g, 65%). Under nitrogen, a toluene solution of 1 mmol of the ligand and 1.2 eq. Py_2NiMe_2 was stirred for 1 hour at room temperature. After concentrating, adding n-hexane for recrystallization resulted in the formation of **Ni-F-OH (**610mg, 82%**)** as a yellow solid. Under nitrogen, add 1 eq. NaH to the toluene solution with 1 mmol **Ni-F-OH**, stir at room temperature for 1 hour, and drain the solvent to obtain **Ni-F-ONa** (700 mg, 91%). **ligand 2**: ^{1}H NMR (400 MHz, Chloroform-*d*) δ 7.46 (td, J = 7.5, 1.5 Hz, 1H), 7.36-7.28 (m, 4H), 7.27 (s, 2H), 7.24-7.20 (m, 3H), 6.71 (d, J = 3.0 Hz, 1H), 6.59-6.41 (m, 3H), 6.02 (s, 1H), 4.80 (s, 1H), 3.49 (s, 3H), 3.45 (s, 3H). ^{31}P NMR (162 MHz, $CDCl_3$) δ -33.49. ^{19}F NMR (377 MHz, Chloroform-*d*) δ -139.62 (ddd, J = 111.3, 23.4, 8.2 Hz), -155.80 (t, J = 21.1 Hz), -163.01 (td, J = 21.7, 21.0, 5.8 Hz).^{13}C NMR (101 MHz, $CDCl_3$) δ 157.68, 157.45, 150.59, 150.39, 148.62, 141.62, 141.29, 134.57, 133.74, 133.69, 133.55, 133.50, 133.42, 131.24, 131.17, 129.85, 129.63, 128.77, 128.51, 128.44, 127.81, 121.94, 121.91, 119.62, 118.52, 118.44, 113.98, 104.05, 104.01, 103.80, 77.35, 77.24, 77.03, 76.72, 55.56,

55.43. ESI-MS (m/z): $[M+H]^+$ Calcd for C32 H22 O4 F5 P, 597.12486; Found: 597.12488. **Ni-F-OH**: ^{1}H NMR (400 MHz, C_6D_6) δ 8.28 (s, 2H), 7.62-7.48 (m, 3H), 7.10-6.85 (m, 5H), 6.82-6.61 (m, 4H), 6.75-650 (m, 4H), 6.34 (s, 2H), 6.07 (d, *J* = 8.2 Hz, 1H), 3.35 (s, 3H),3.18 (s, 3H), -0.80(s, 3H, Ni-*Me*). ^{31}P NMR (161 MHz, C_6D_6) δ 20.70. ^{19}F NMR (377 MHz, Chloroform-*d*) δ -137.83, -139.84,-159.77, -159.84, -165.40. Anal. Calcd for C38H29F5NNiO4P: C, 60.99; H, 3.91; N, 1.87. Found: C, 60.79; H, 3.90; N, 1.91.

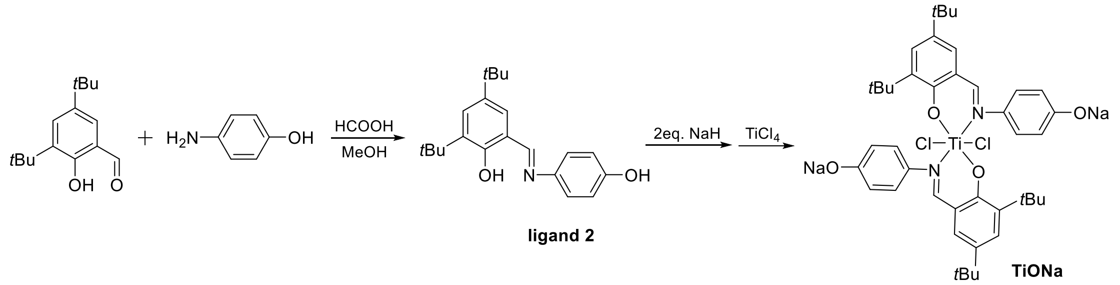

Ti-ONa: A mixture of 5 mmol of 3,5-di-tert-butyl-2-hydroxybenzaldehyde, 5 mmol of p-aminophenol and 1mL of formic acid in 150 mL of methanol was refluxed at 80°C for 12 hours. After concentrating, adding n-hexane for recrystallization resulted in the formation of **ligand 3** as a yellow solid (1.38g, 85%). Under nitrogen, a mixture of 1 mmol of **ligand 3** and 2 eq. NaH was stirred in 30 mL toluene for 1 hour. 0.5 eq. $TiCl_4$ was add and reacted for another 1 hour. Under nitrogen, filter through filter paper and add toluene (20 mL) to wash three times, collect the filtrate and concentrate, add n-hexane to recrystallize to obtain the catalyst **Ti-ONa** a red solid (370mg,91%). To a stirred solution of **ligand 3** (1 mmol) in dried Et_2O (300 ml) at –78 °C, a 2.50 M n-butyllithium n-hexane solution (1 mmol) was added dropwise. The solution was allowed to warm to room temperature and stirred for 3 h. 0.5 eq. $TiCl_4$ was added at –78 °C, and reacted for 1 hour. Concentration of the reaction mixture in vacuo yielded a crude product. Dried DCM was added to the crude product, and the mixture was stirred for 15 min and filtered. The solid residue was washed with dried DCM, and the combined organic filtrates were concentrated in vacuo to afford a brown solid of **Ti-OH** (310mg, 81%). **ligand 3**: ^{1}H NMR (600 MHz, Chloroform-*d*) δ 8.61 (s, 1H), 7.43 (d, *J* = 2.4 Hz, 1H), 7.24 -7.21 (m, 2H), 7.20 (d, *J* = 2.4 Hz, 1H), 6.87 (d, *J* = 8.5 Hz, 2H), 1.48 (s, 9H), 1.33 (s, 9H). ^{13}C NMR (151 MHz, Chloroform-*d*) δ 161.18, 157.36, 153.78, 141.23, 139.80, 136.20, 126.91, 125.87, 121.73, 121.72, 121.71, 121.70, 121.69, 117.71, 115.38, 115.36, 76.51, 76.30, 76.09, 34.38, 33.47, 30.81, 30.80, 30.78, 30.76, 30.75, 28.75. ESI-MS (m/z): $[M+H]^+$ Calcd for C21 H27 O2 N, 326.21146; Found: 326.21237.**Ti-ONa**: ^{1}H NMR (600 MHz, Benzene-*d*$_6$) δ 7.83 (s, 2H), 7.66 - 7.05 (m, 4H), 6.45 (d, *J* = 63.8 Hz, 4H), 6.02 (d, *J* = 130.8 Hz, 2H), 1.80 - 0.53 (m, 36H). Anal. Calcd for C42H50Cl2N2Na2O4Ti: C, 62.16; H, 6.21; N, 3.45. Found: C, 62.36; H, 6.17; N, 3.32. MALDI-TOF: m/z 775.27 $[M- Cl]^+$; 777.62 $[M-Cl]^+$. **Ti-OH**: ^{1}H NMR (600 MHz, Benzene-*d*$_6$) δ 7.42 (s, 2H), 7.27 (s, 2H), 7.14 (s, 2H), 6.85 – 6.69 (m, 2H), 6.54 (d, *J* = 8.3 Hz, 2H), 6.09 (d, *J* = 220.3 Hz, 2H), 1.38 (m, 18H), 0.96 (m, 18H). Anal. Calcd for C42H52Cl2N2O4Ti: C, 65.71; H, 6.83; N, 3.65. Found: C, 65.51; H, 6.87; N, 3.69. MALDI-TOF: m/z 731.31 $[M-Cl]^+$; 731.45 $[M-Cl]^+$.

Preparation of supported catalysts. The pretreated support was dispersed in toluene. The catalyst toluene solution was added dropwise and stirred for another hour. The solid was collected and rinsed with toluene until there was no catalyst in the filtrate, dried under vacuum and re-suspended in heptane for polymerization. **Ni-ONa-SiO_2, Ni-ONa-TiO_2, Ni-ONa-Al_2O_3, Ni-ONa-ZnO, Ni-ONa-MgO, Ni-F-ONa-MgO, Ti-ONa-SiO_2, Ti-ONa-Al_2O_3** and **Ti-ONa-MgO** were all prepared according to the above method. The same metal loading (20 µmol/g) was used for all the supports (10 µmol/g loading was used for the cases of **Ni-SiO_2** and **Ti-SiO_2**). The current loading is lower than the maximum loading value for all of these supports. This is not the maximum amount of catalyst that these supports can carry. **Ni-ONa-GR** (**Ni-ONa** catalyst was supported on pretreated graphene), **Ni-ONa-EG** (**Ni-ONa** catalyst was supported on pretreated Expanded graphite), **Ni-ONa-GF** (**Ni-ONa** catalyst was supported on pretreated glass fiber), **Ni-ONa-APP** (**Ni-ONa** catalyst was supported on pretreated ammonium polyphosphate) were all prepared according to the above method.

Determination of the maximum adsorption capacity of different metal complexes on Solid support. The pretreated support (1 g) was stirred in 5 mL toluene. A 10 µmol/mL catalyst toluene solution was added dropwise, until the supernatant liquid showed UV signal. The supported catalyst was filtered, rinsed with toluene three times until the filtrate showed no UV signal. The metal content was measured by ICP, and the maximum catalyst load was calculated accordingly.

3. Supplementary Figures of Characterization of ligands and homogeneous catalysts

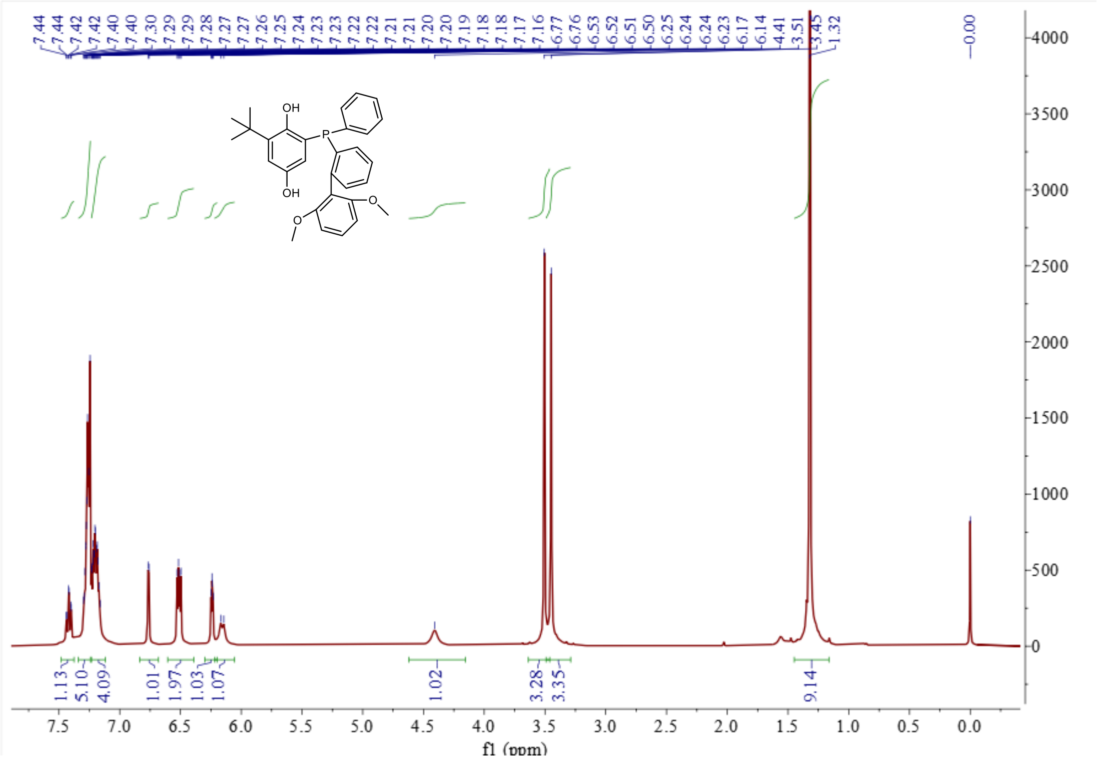

Supplementary Figure 10. ^{1}H NMR spectrum of the **ligand 1.** ($CDCl_3$)

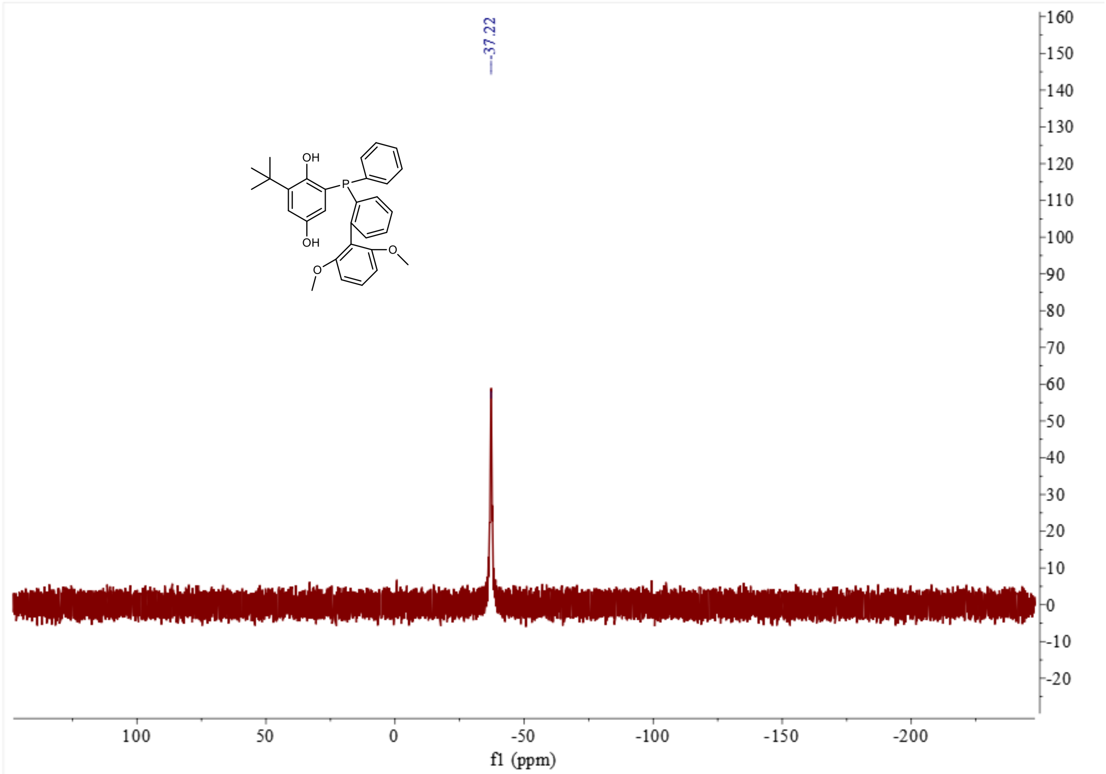

Supplementary Figure 11.^{31}P NMR spectrum of the **ligand 1.** ($CDCl_3$)

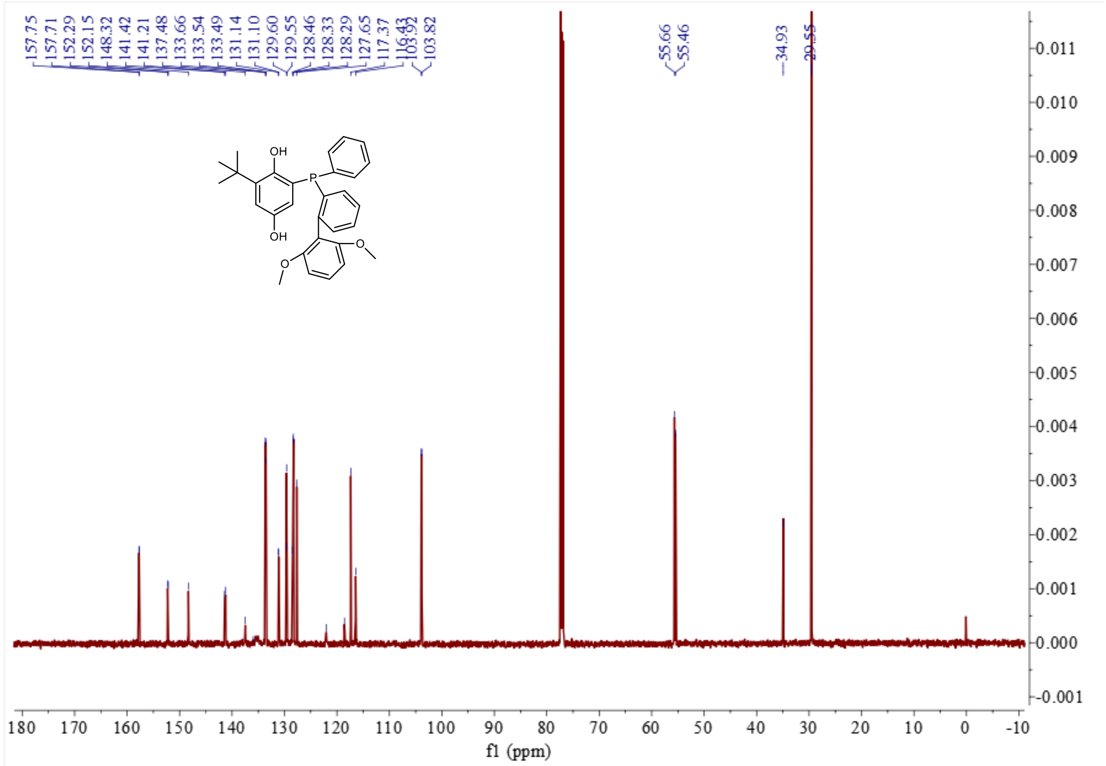

Supplementary Figure 12.^{13}C NMR spectrum of the **ligand 1.** ($CDCl_3$)

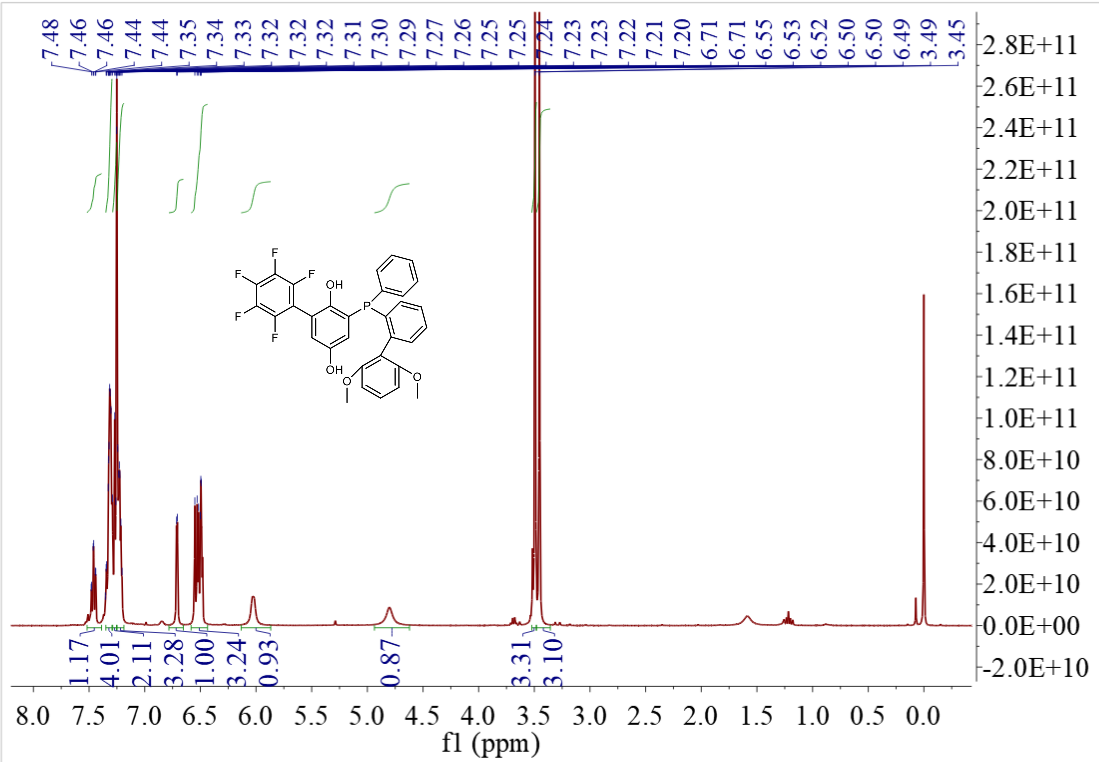

Supplementary Figure 13. ^{1}H NMR spectrum of the **ligand 2.** ($CDCl_3$)

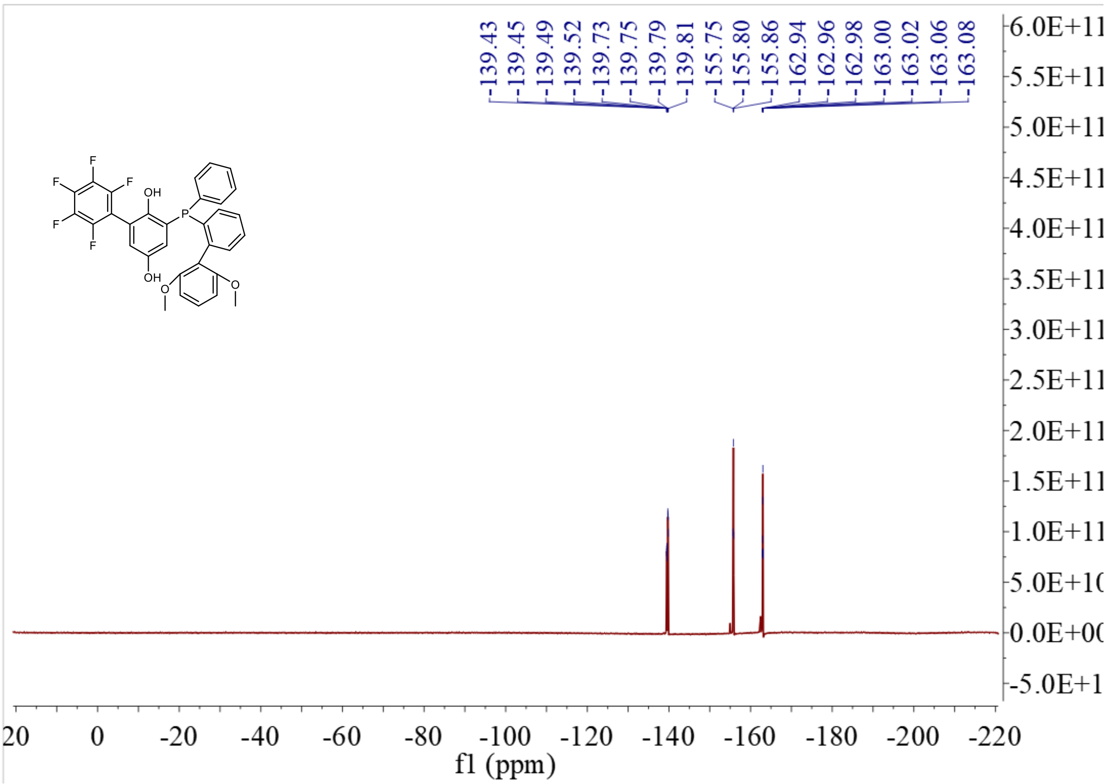

Supplementary Figure 14. ^{19}F NMR spectrum of the **ligand 2.** ($CDCl_3$)

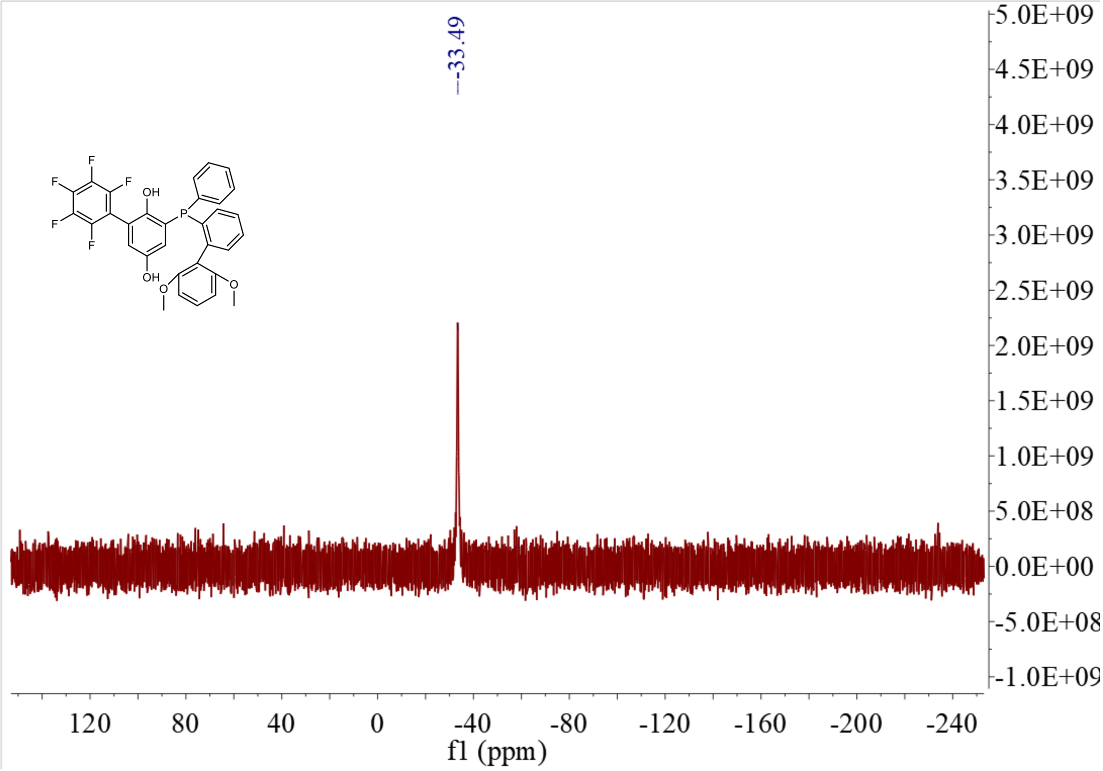

Supplementary Figure 15.^{31}P NMR spectrum of the **ligand 2.** ($CDCl_3$)

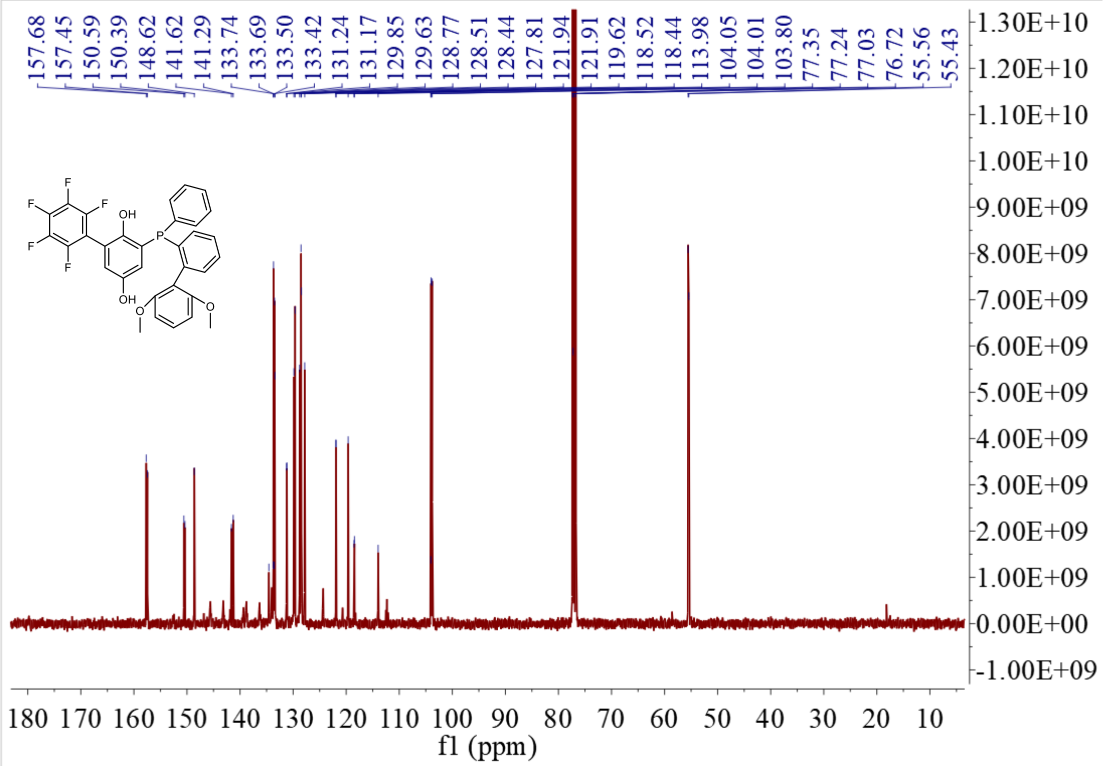

Supplementary Figure 16.^{13}C NMR spectrum of the **ligand 2.** ($CDCl_3$)

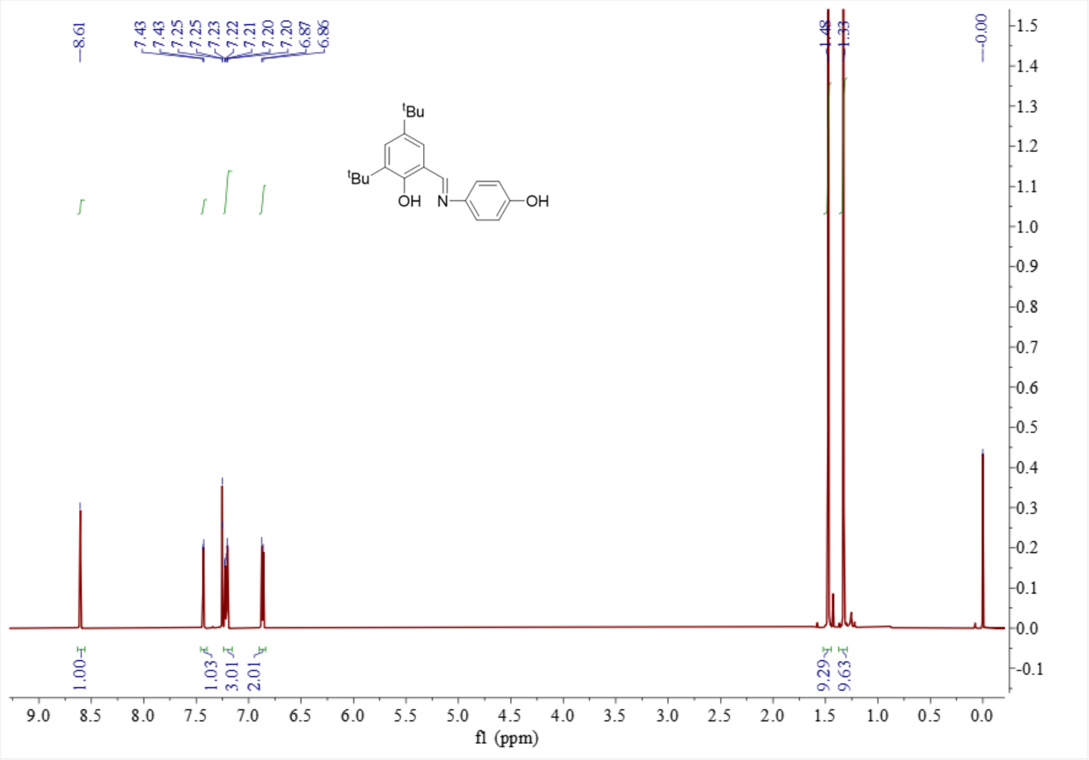

Supplementary Figure 17. ^{1}H NMR spectrum of the **ligand 3.** (CDCl$_3$)

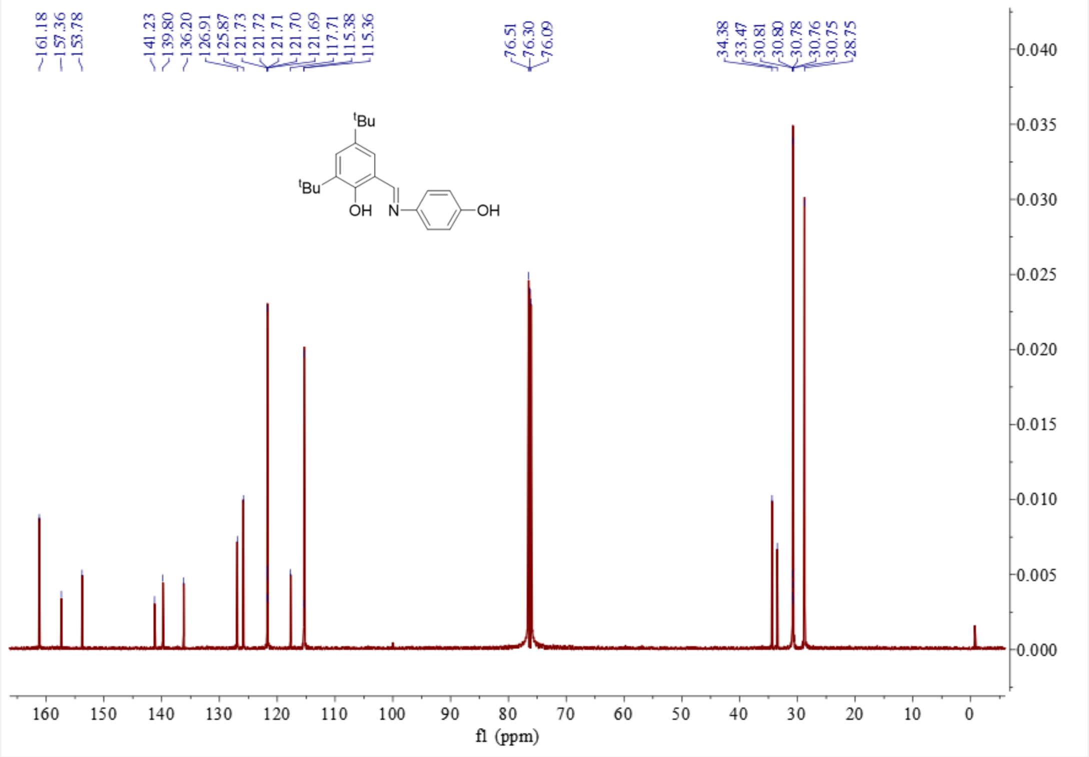

Supplementary Figure 18. ^{13}C NMR spectrum of the **ligand 3.** (CDCl$_3$)

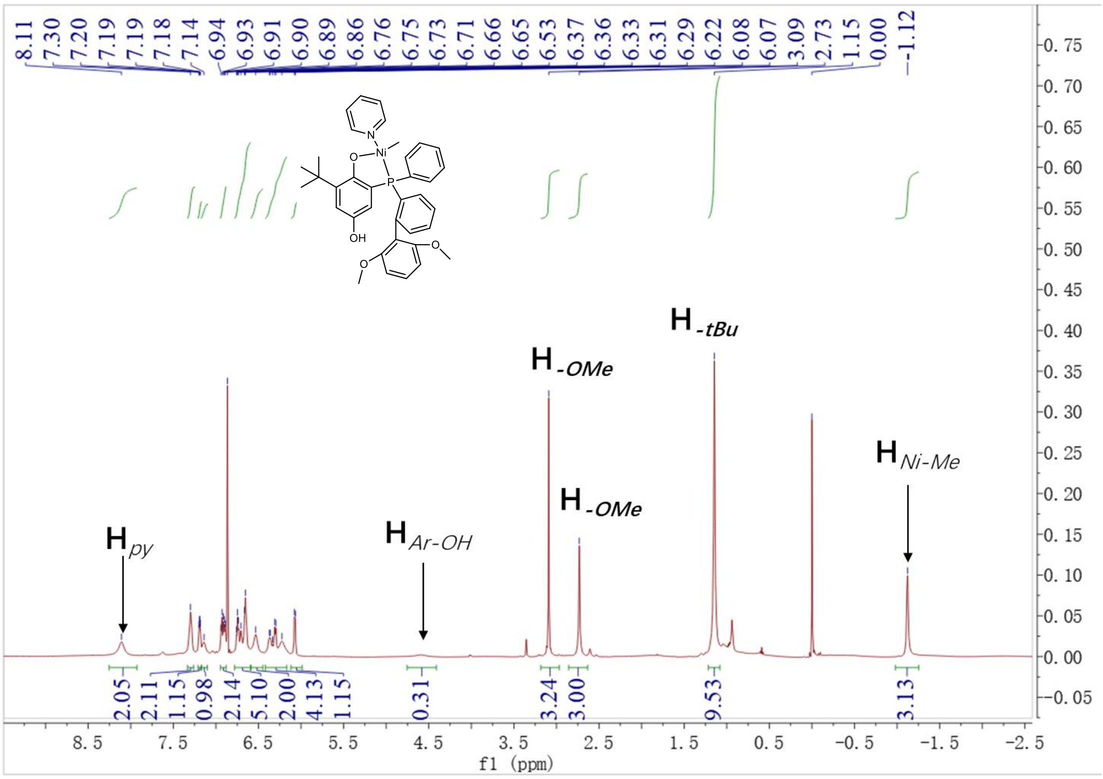

Supplementary Figure 19. ^{1}H NMR spectrum of the **Ni-OH.** (C_6D_6)

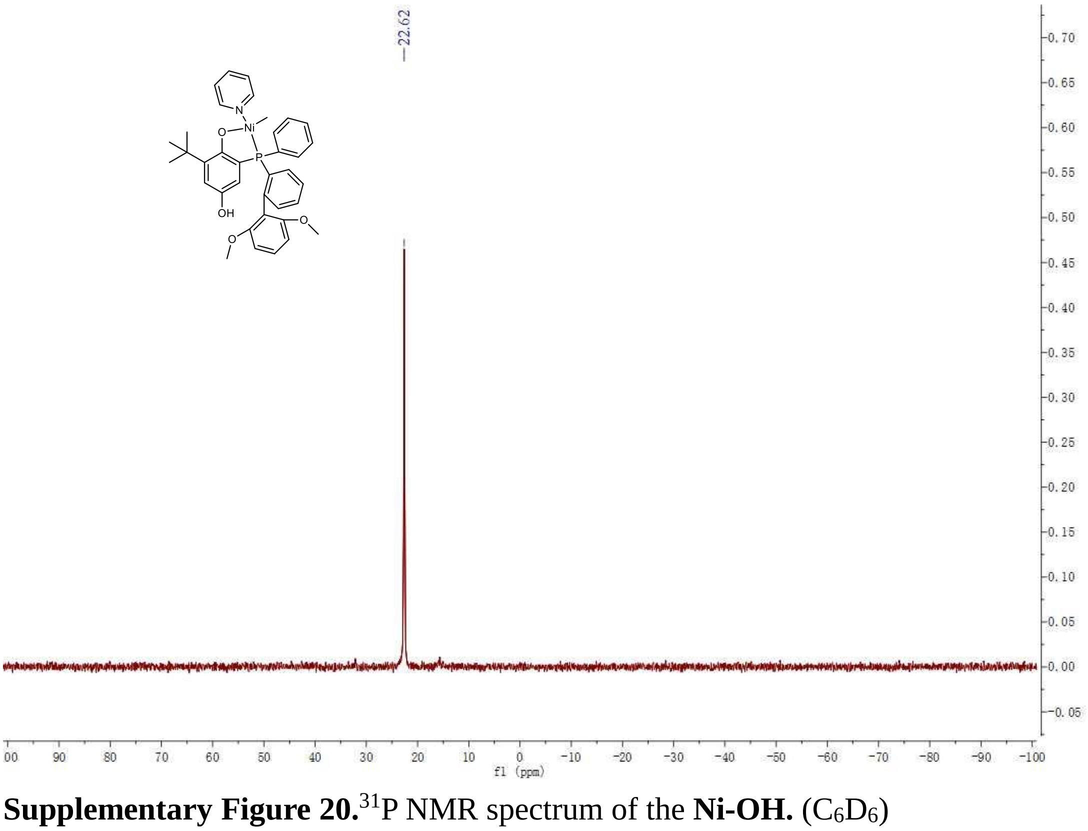

Supplementary Figure 20. ^{31}P NMR spectrum of the **Ni-OH.** (C_6D_6)

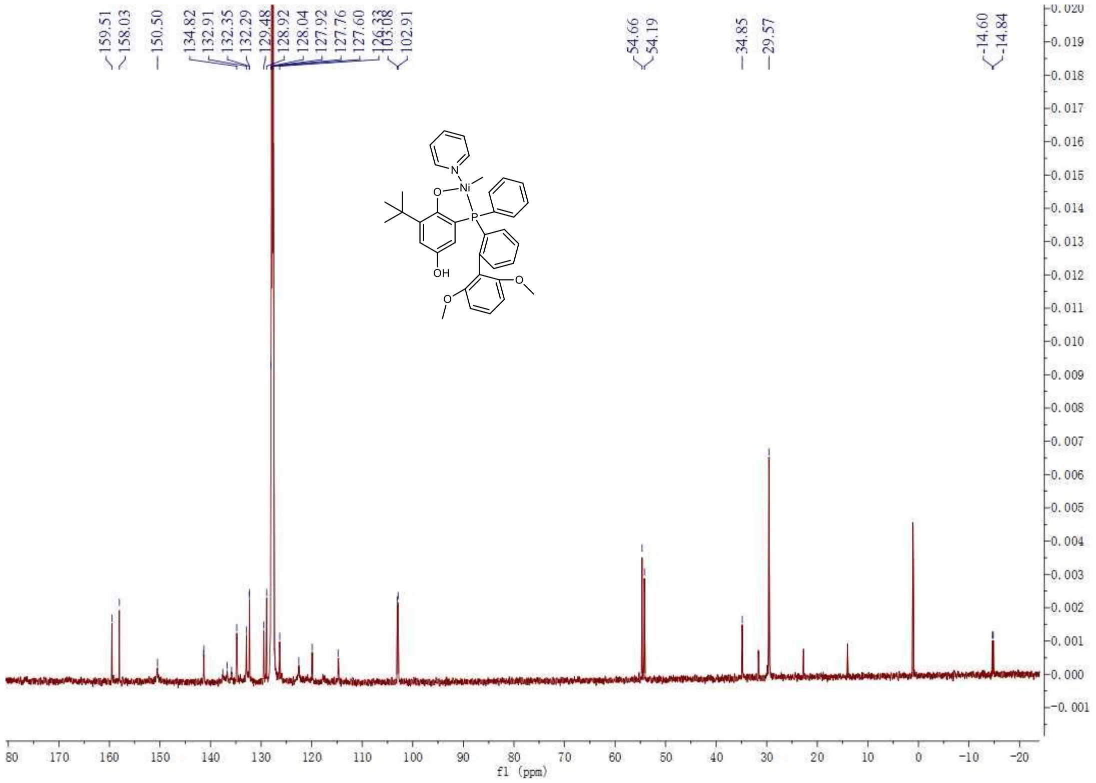

Supplementary Figure 21.^{13}C NMR spectrum of the **Ni-OH.** (C_6D_6)

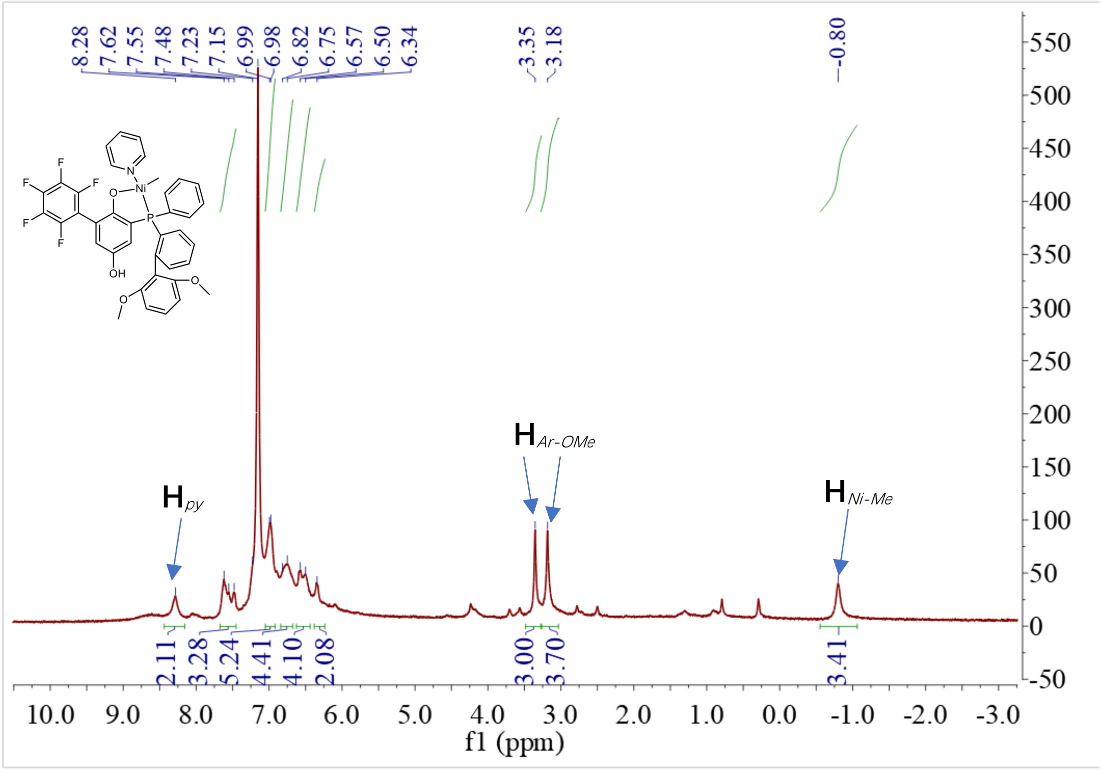

Supplementary Figure 22.^{1}H NMR spectrum of the **Ni-F-OH.** (C_6D_6)

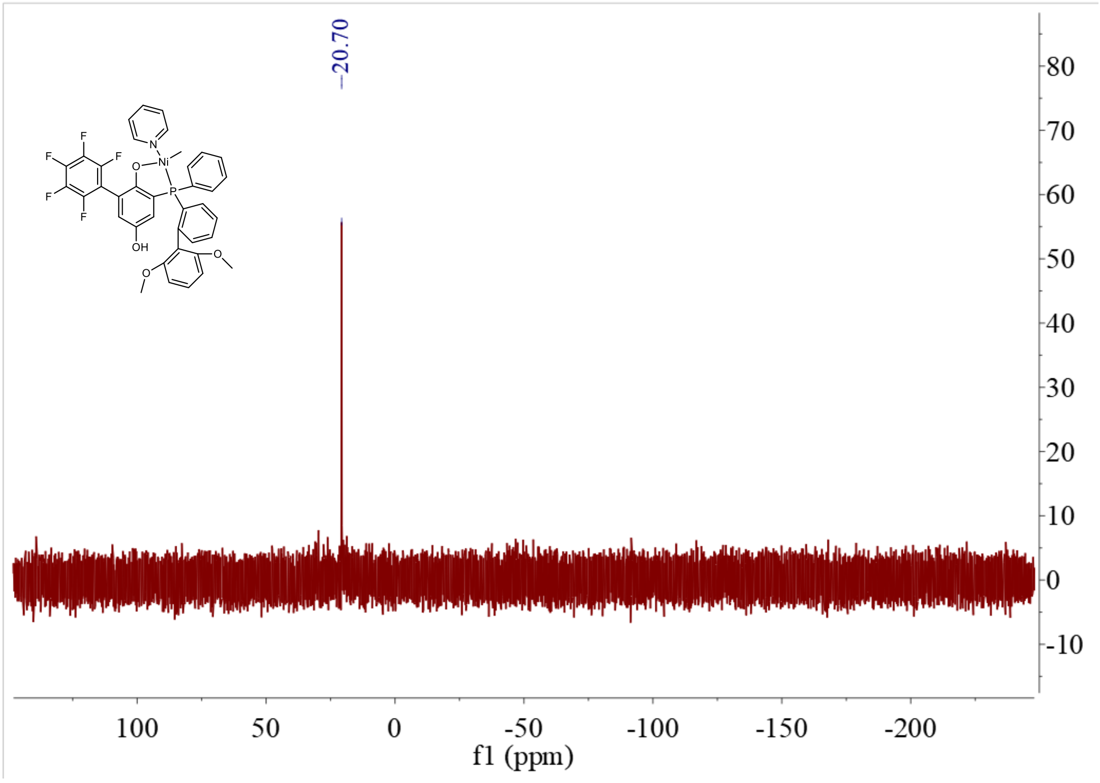

Supplementary Figure 23. ^{31}P NMR spectrum of the **Ni-F-OH.** (C_6D_6)

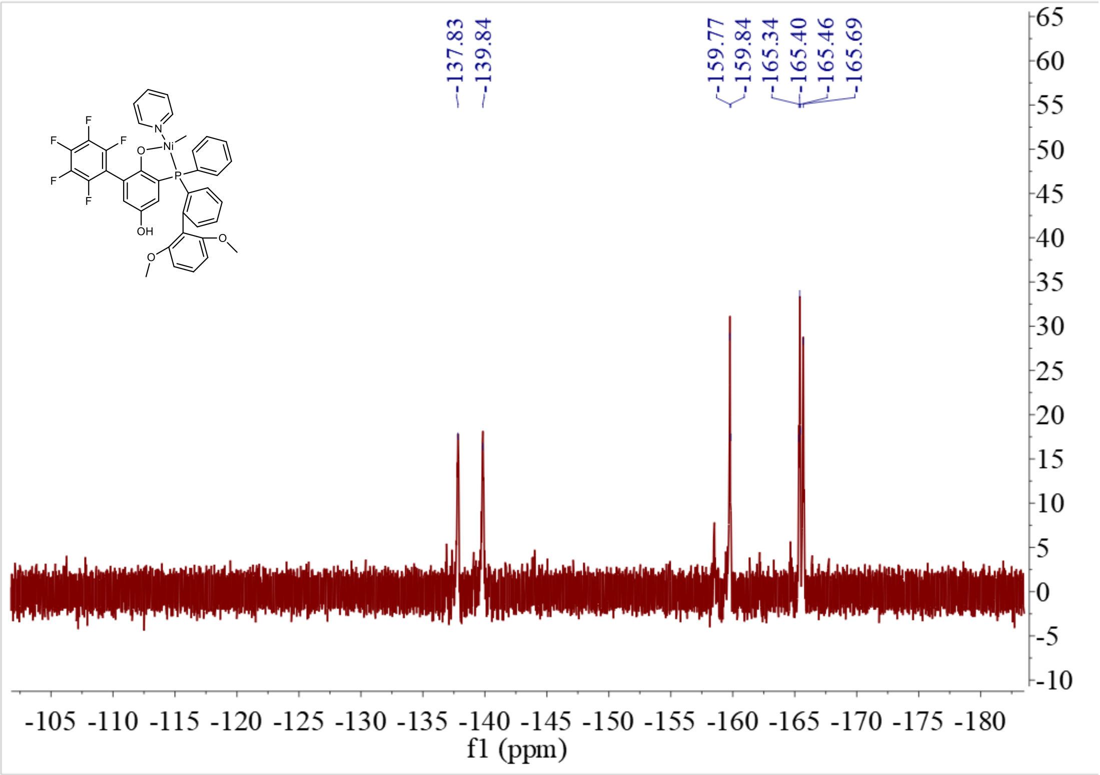

Supplementary Figure 24. ^{19}F NMR spectrum of the **Ni-F-OH.** (C_6D_6)

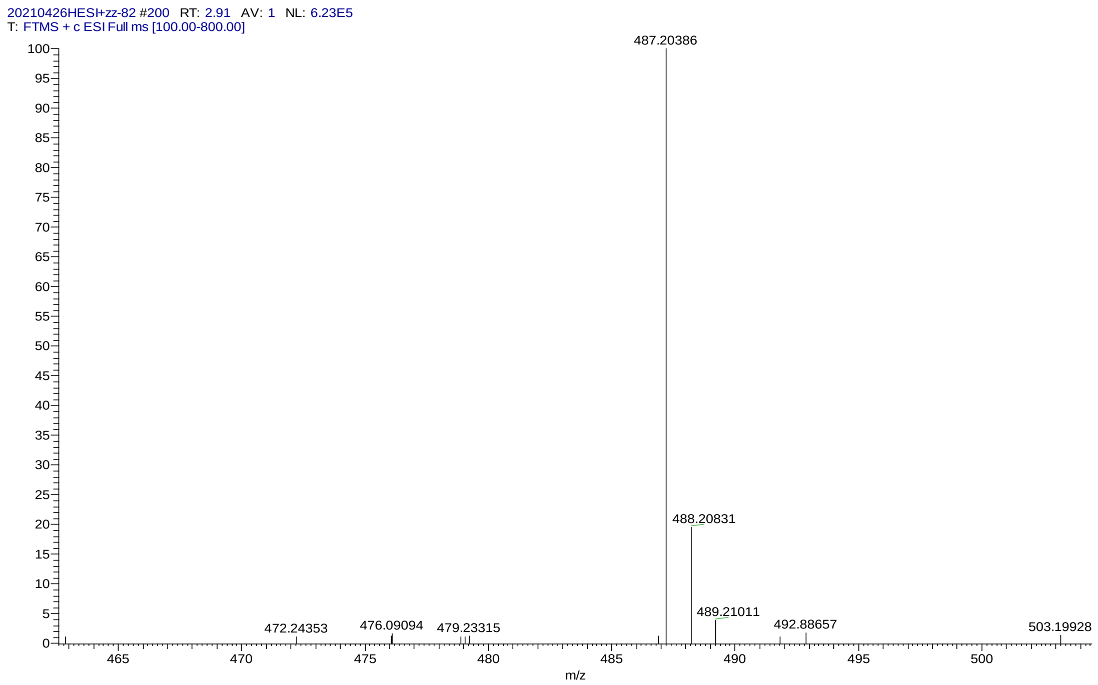

Supplementary Figure 25. ESI-MS of **ligand 1.**

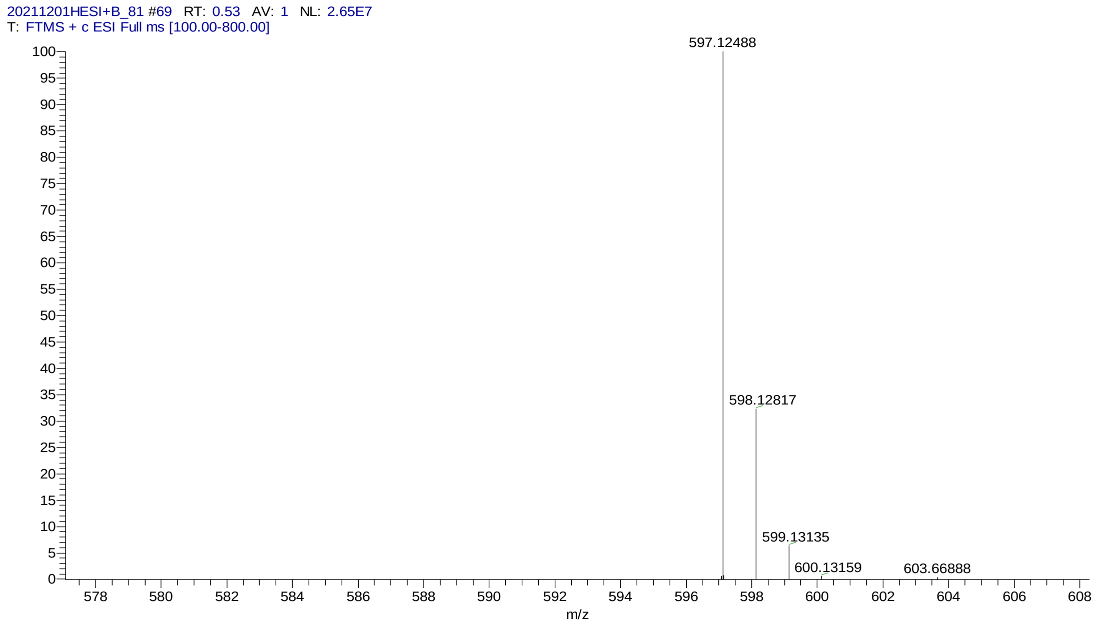

Supplementary Figure 26. ESI-MS of **ligand 2.**

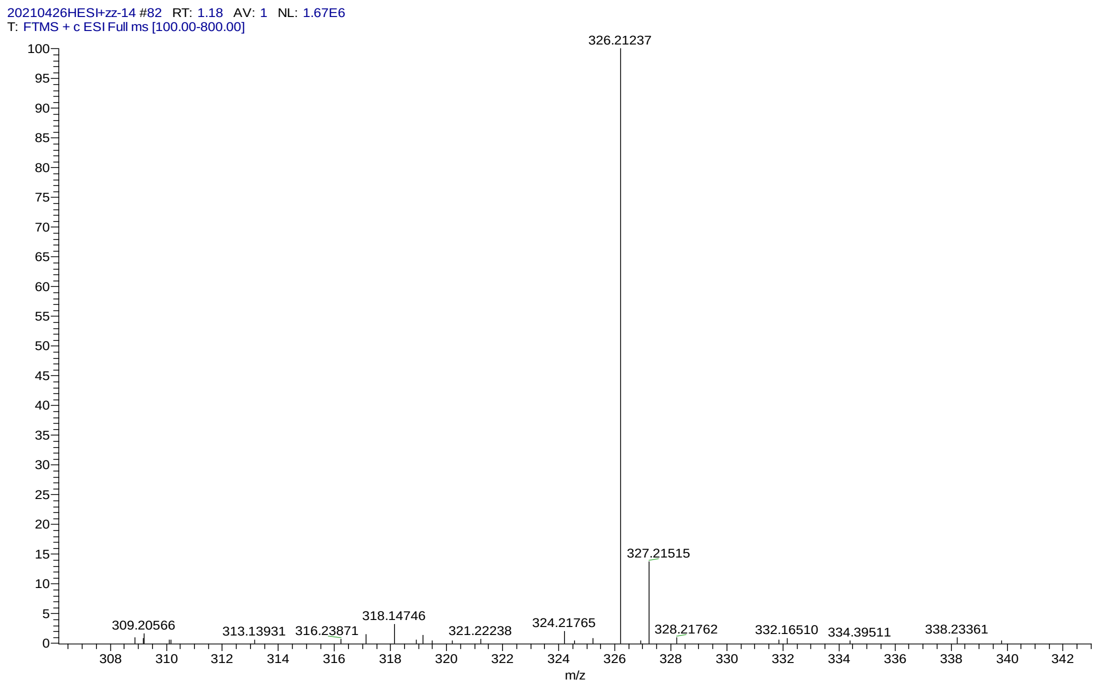

Supplementary Figure 27. ESI-MS of **ligand 3.**

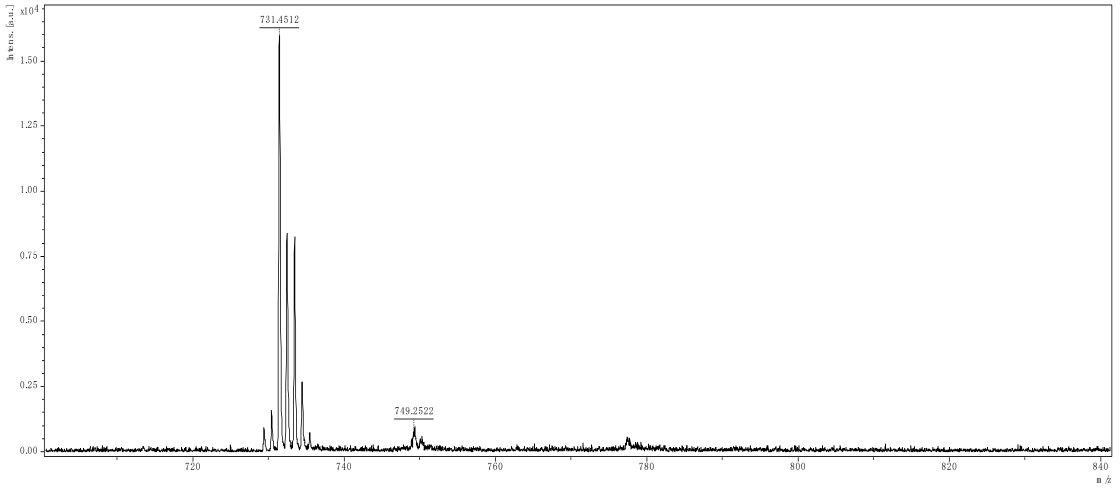

Supplementary Figure 28. MALDI-TOF of catalyst **Ti-OH**.

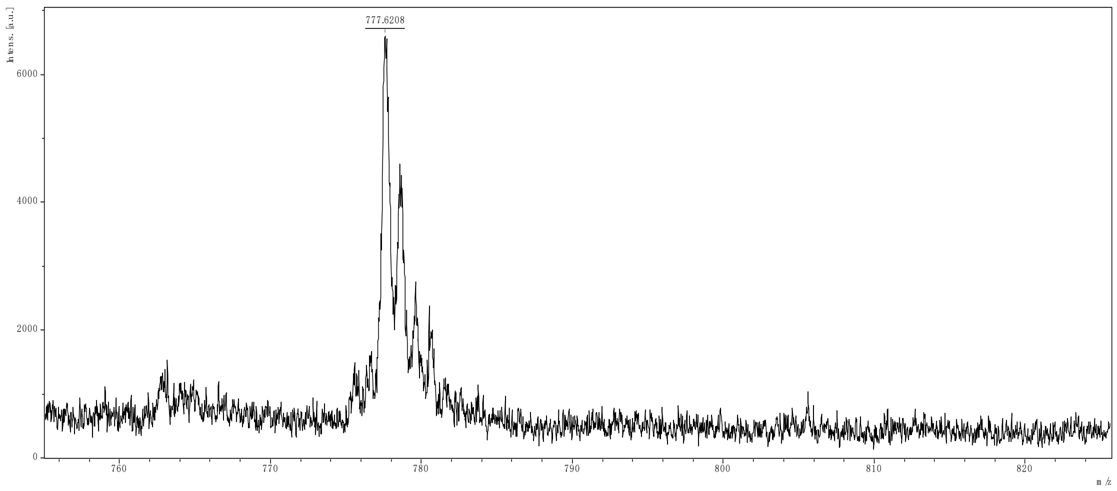

Supplementary Figure 29. MALDI-TOF of catalyst **Ti-ONa**.

4 Supplementary Figures of ^{1}H NMR and ^{13}C NMR of copolymers

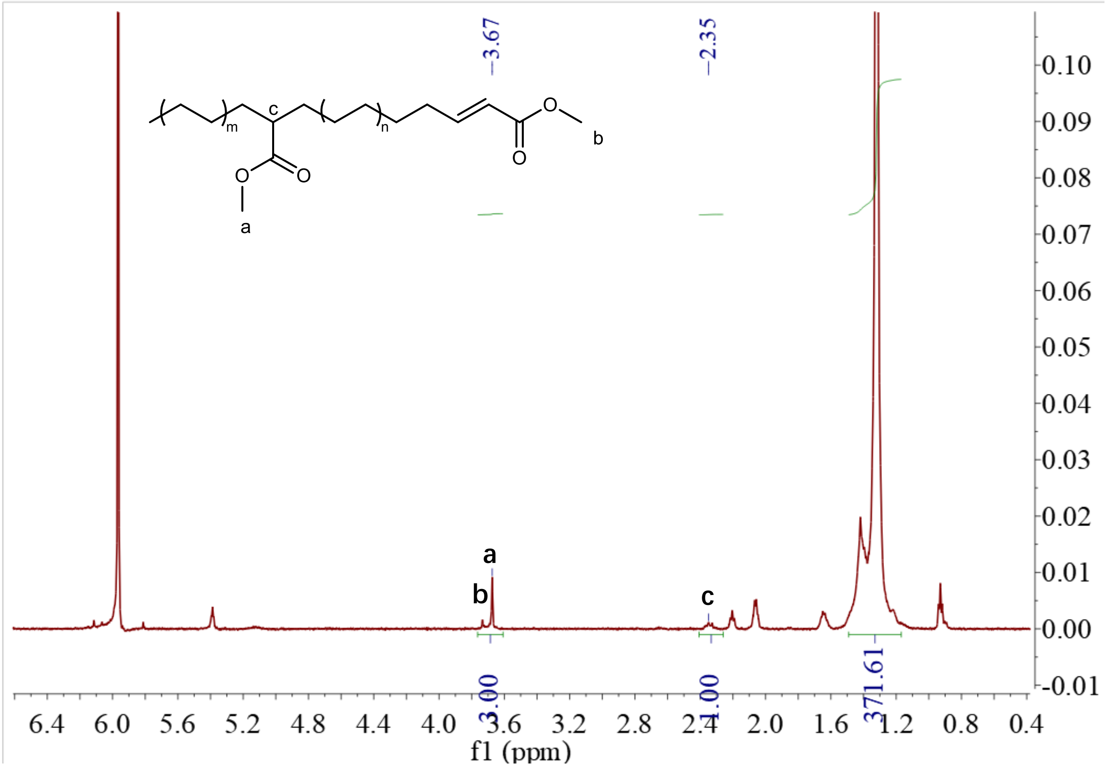

Supplementary Figure 30. ^{1}H NMR spectrum of the polymer from Table 2, Entry 1 ($C_2D_2Cl_4$, 120°C).

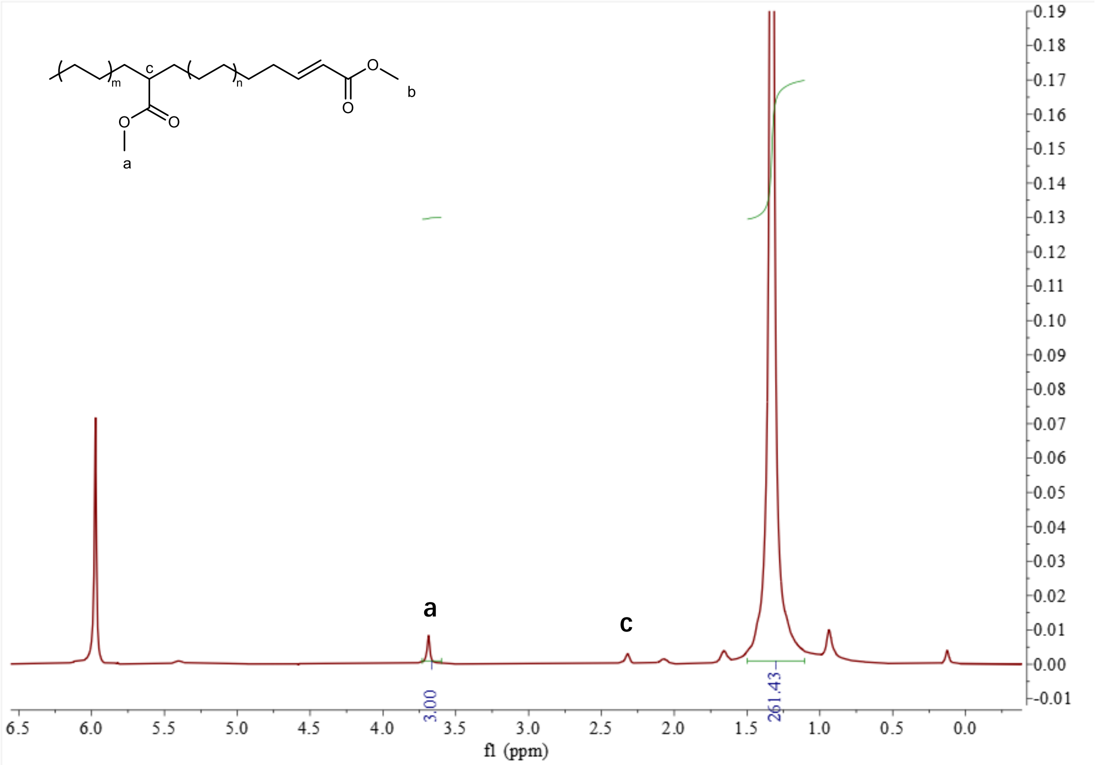

Supplementary Figure 31. ^{1}H NMR spectrum of the polymer from Table 2, Entry 2 ($C_2D_2Cl_4$, 120°C).

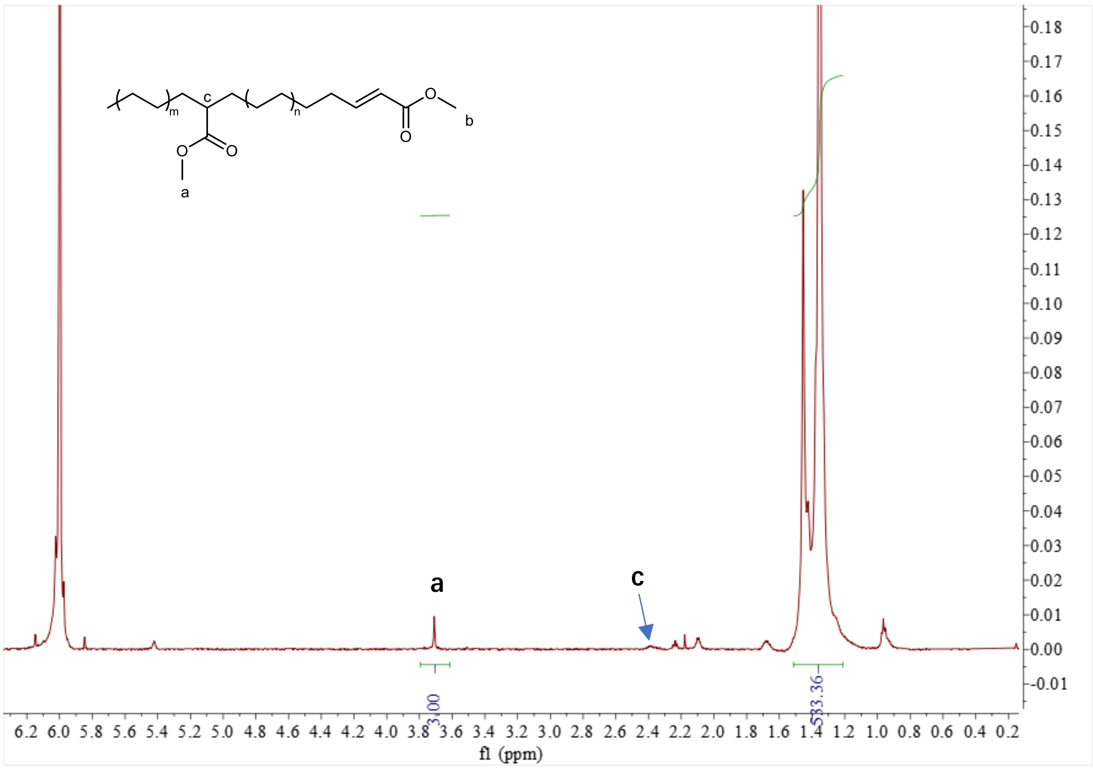

Supplementary Figure 32. ^{1}H NMR spectrum of the polymer from Table 2, Entry 4 ($C_2D_2Cl_4$, 120°C).

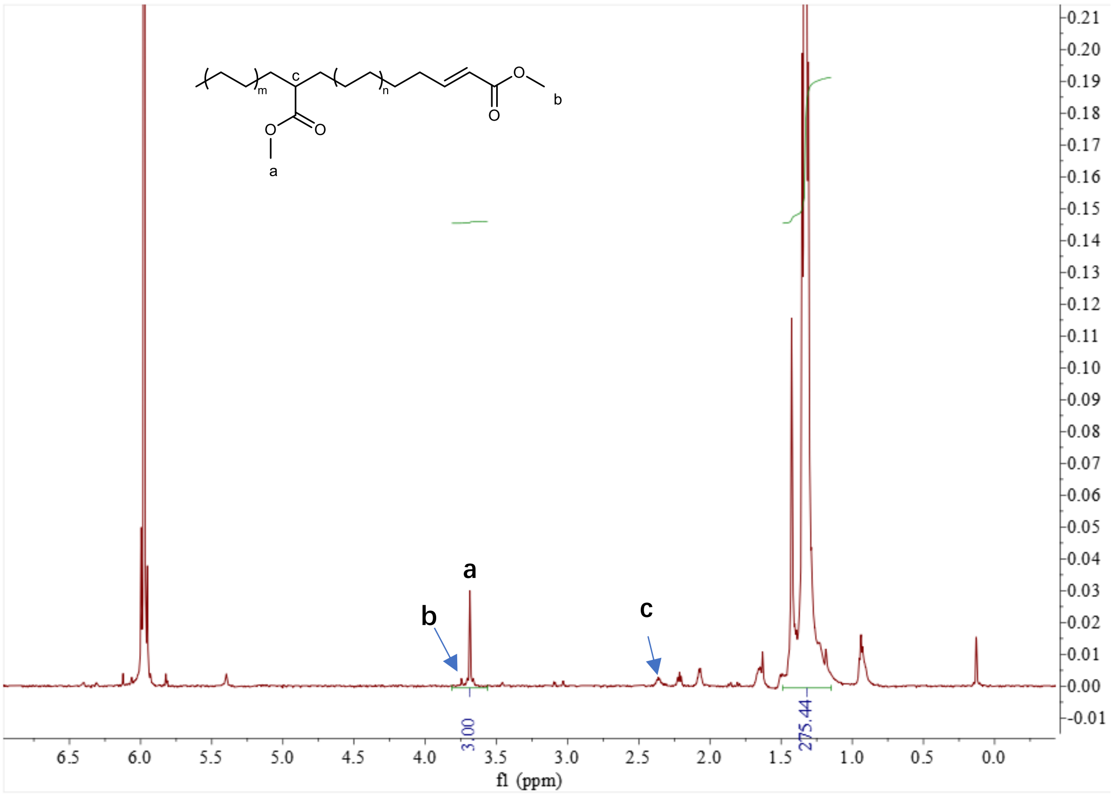

Supplementary Figure 33. ^{1}H NMR spectrum of the polymer from Table 2, Entry 5 ($C_2D_2Cl_4$, 120°C).

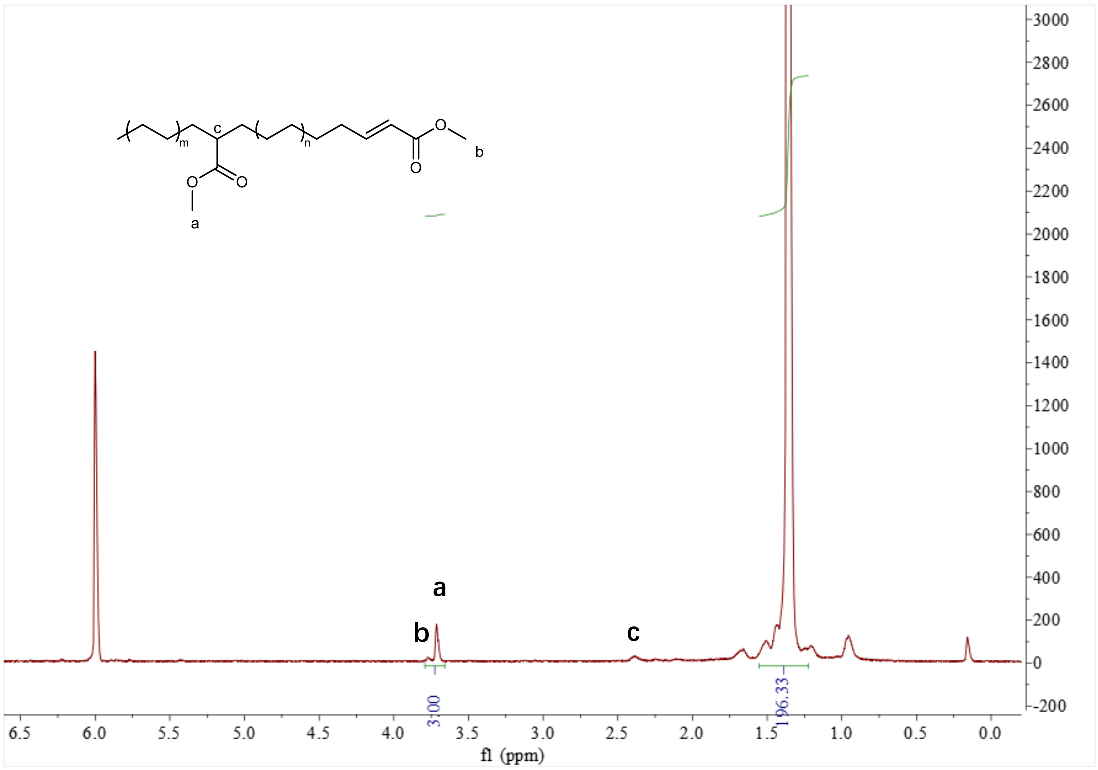

Supplementary Figure 34. ^{1}H NMR spectrum of the polymer from Table 2, Entry 6 ($C_2D_2Cl_4$, 120°C).

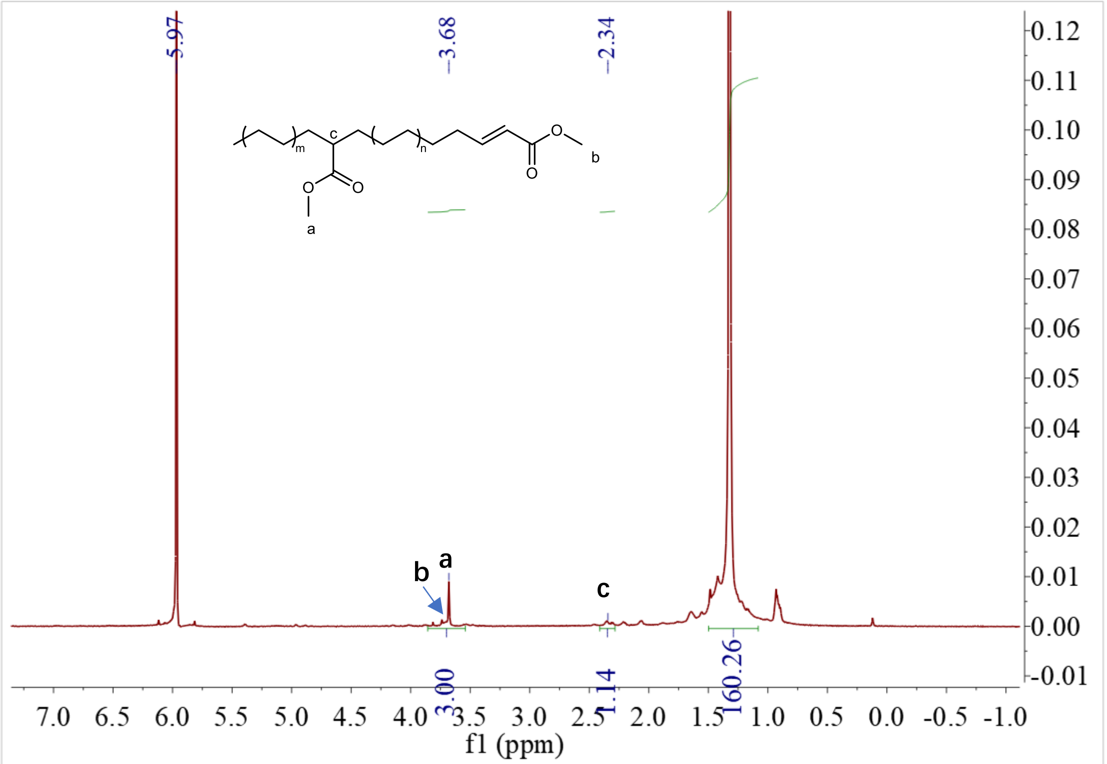

Supplementary Figure 35. ^{1}H NMR spectrum of the polymer from Table 2, Entry 7 ($C_2D_2Cl_4$, 120°C).

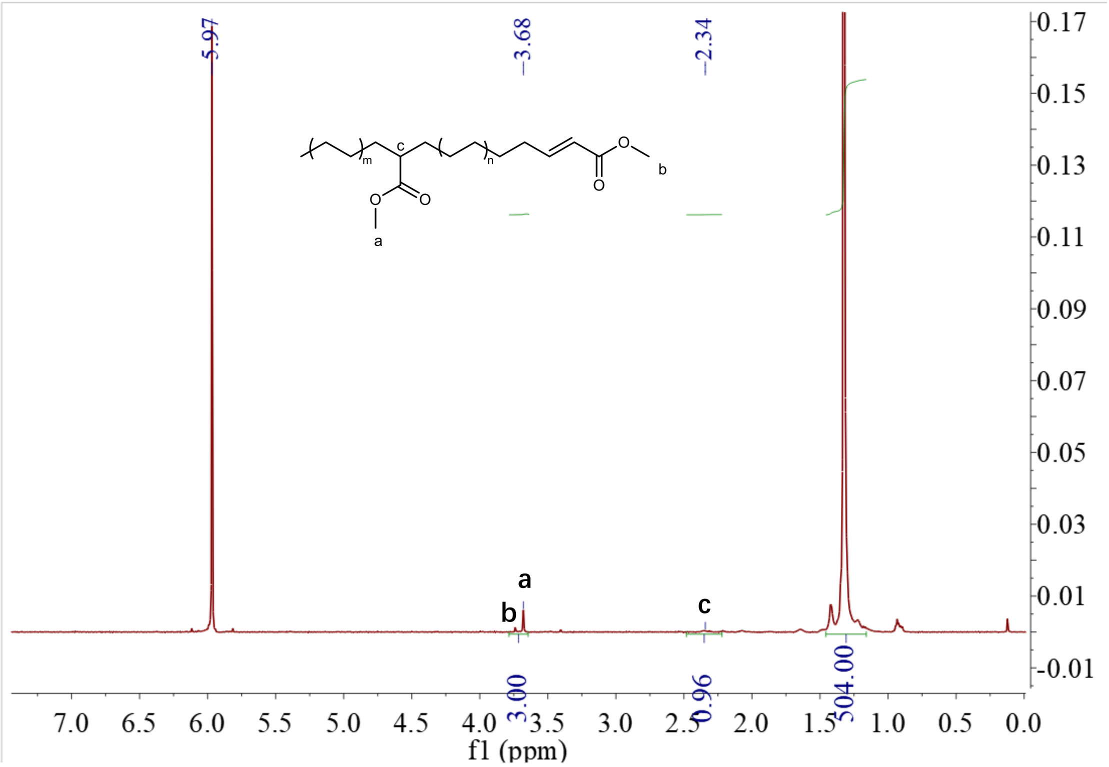

Supplementary Figure 36. ^{1}H NMR spectrum of the polymer from Table 2, Entry 8 ($C_2D_2Cl_4$, 120°C).

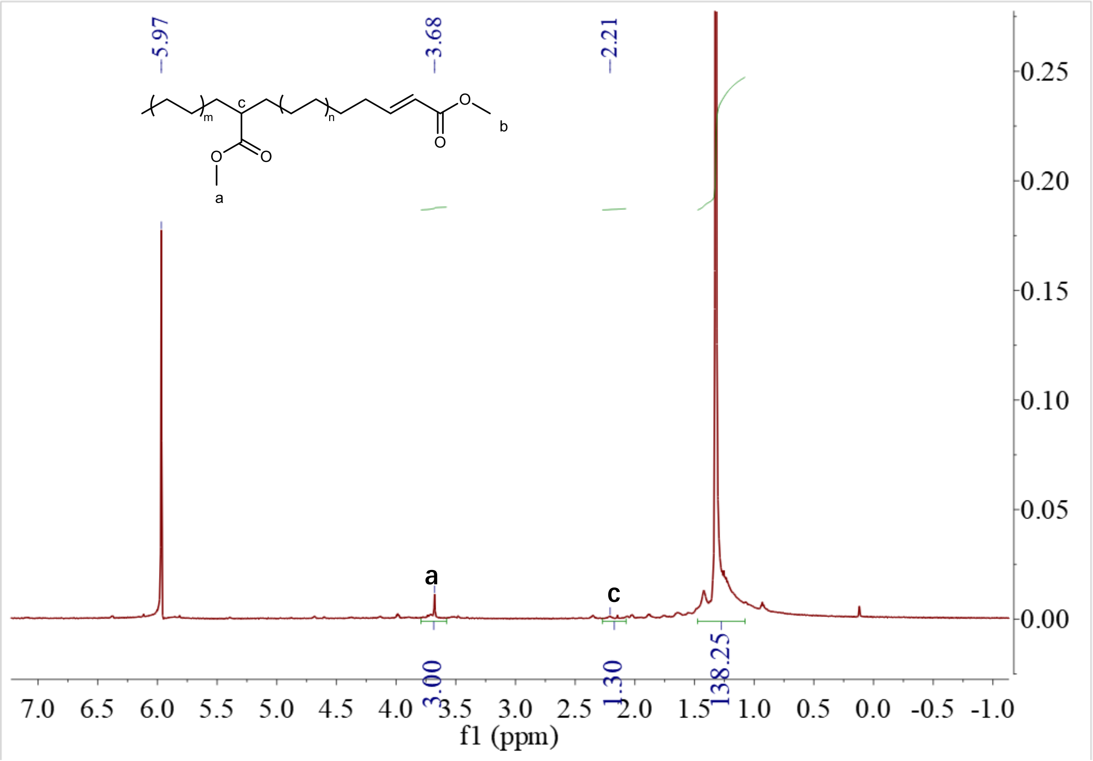

Supplementary Figure 37. ^{1}H NMR spectrum of the polymer from Table 2, Entry 9 ($C_2D_2Cl_4$, 120°C).

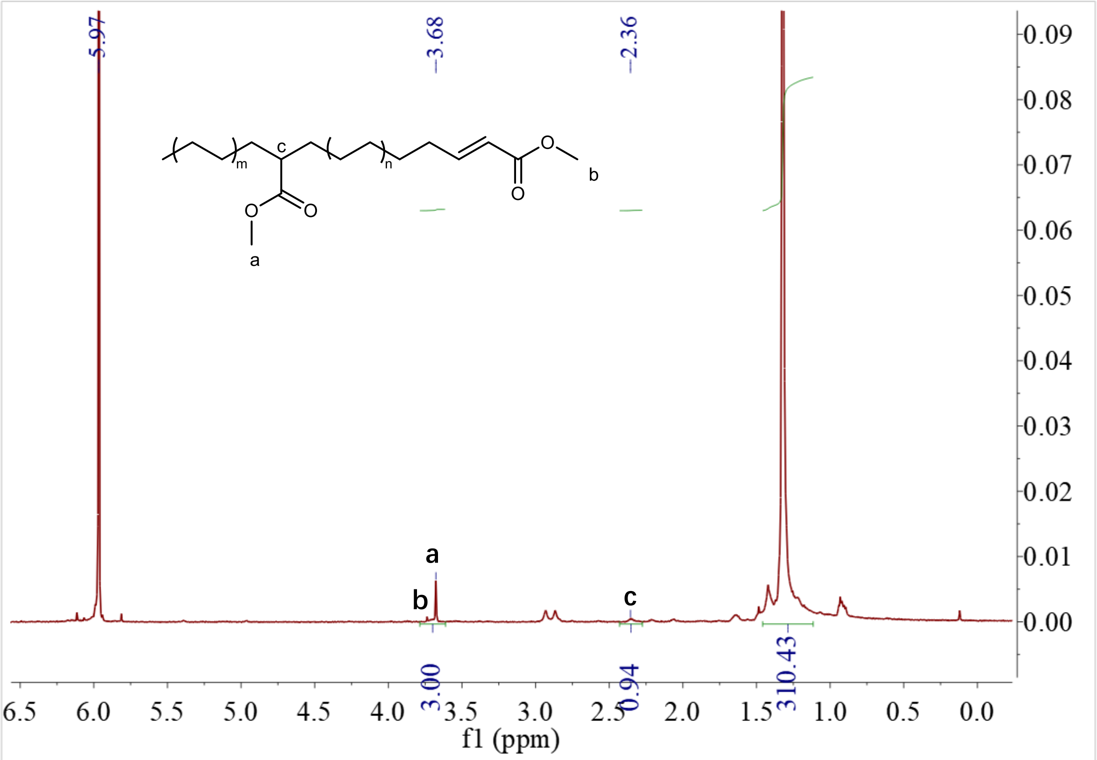

Supplementary Figure 38. ^{1}H NMR spectrum of the polymer from Table 2, Entry 10($C_2D_2Cl_4$, 120°C).

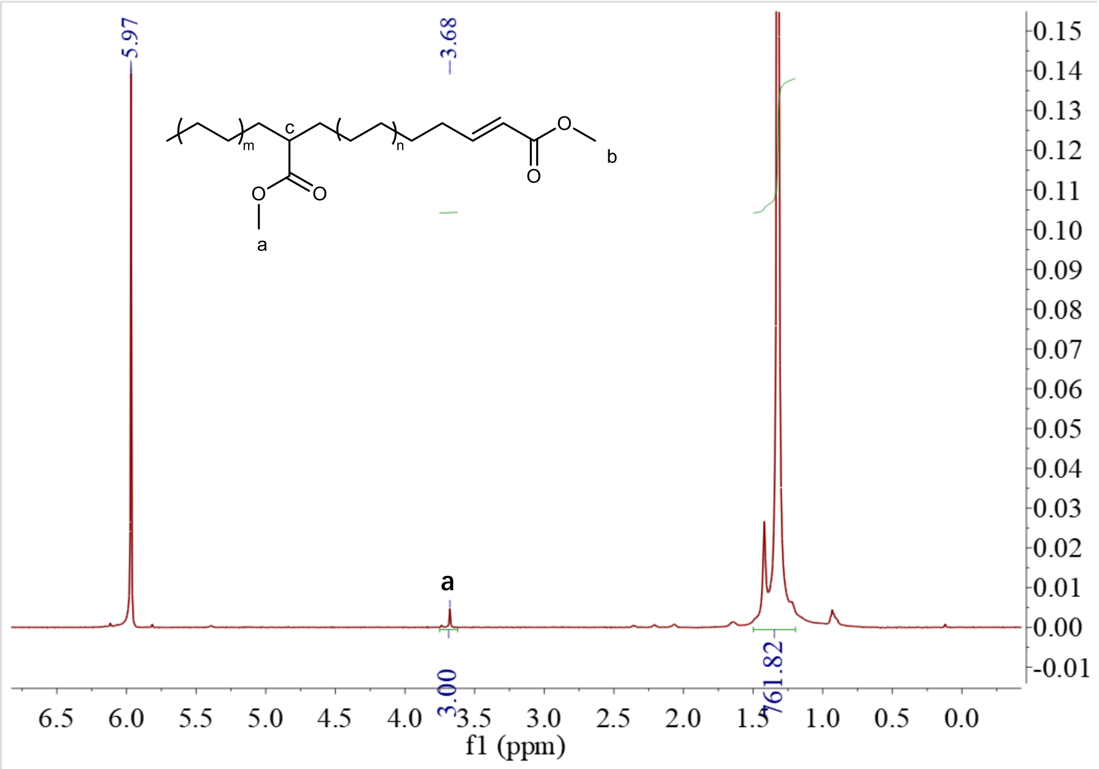

Supplementary Figure 39. ^{1}H NMR spectrum of the polymer from Table 2, Entry 11($C_2D_2Cl_4$, 120°C).

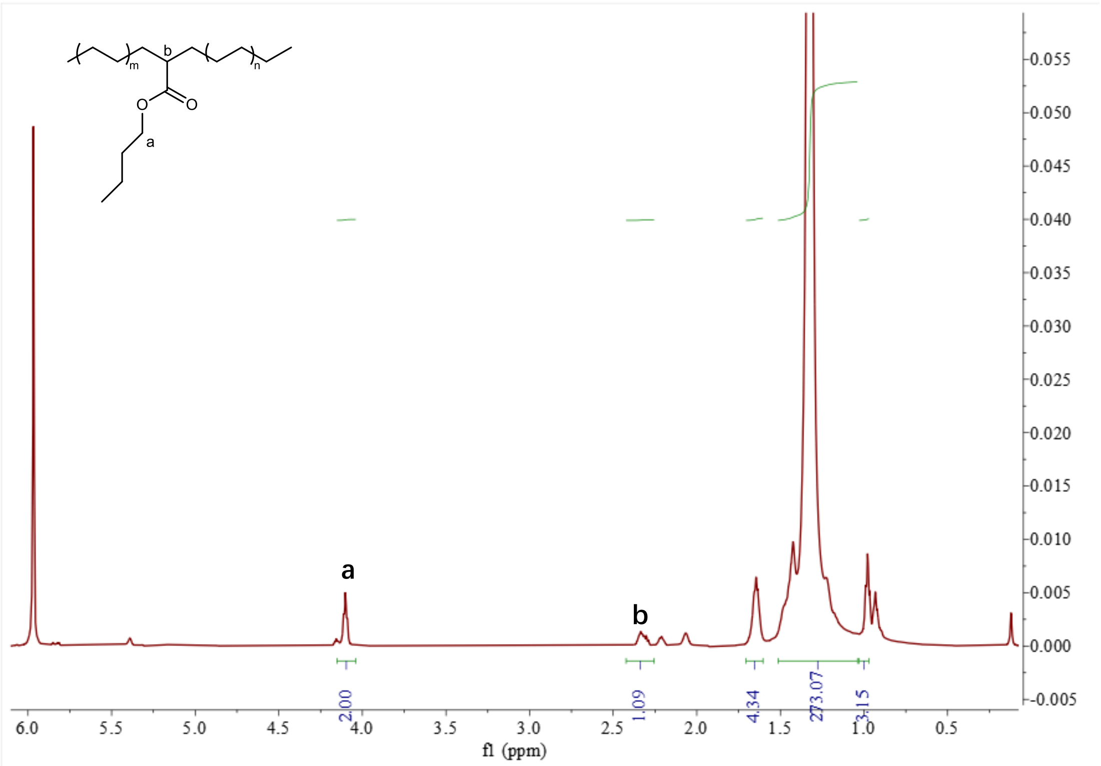

Supplementary Figure 40. ^{1}H NMR spectrum of the polymer from Table 2, Entry 12 ($C_2D_2Cl_4$, 120°C).

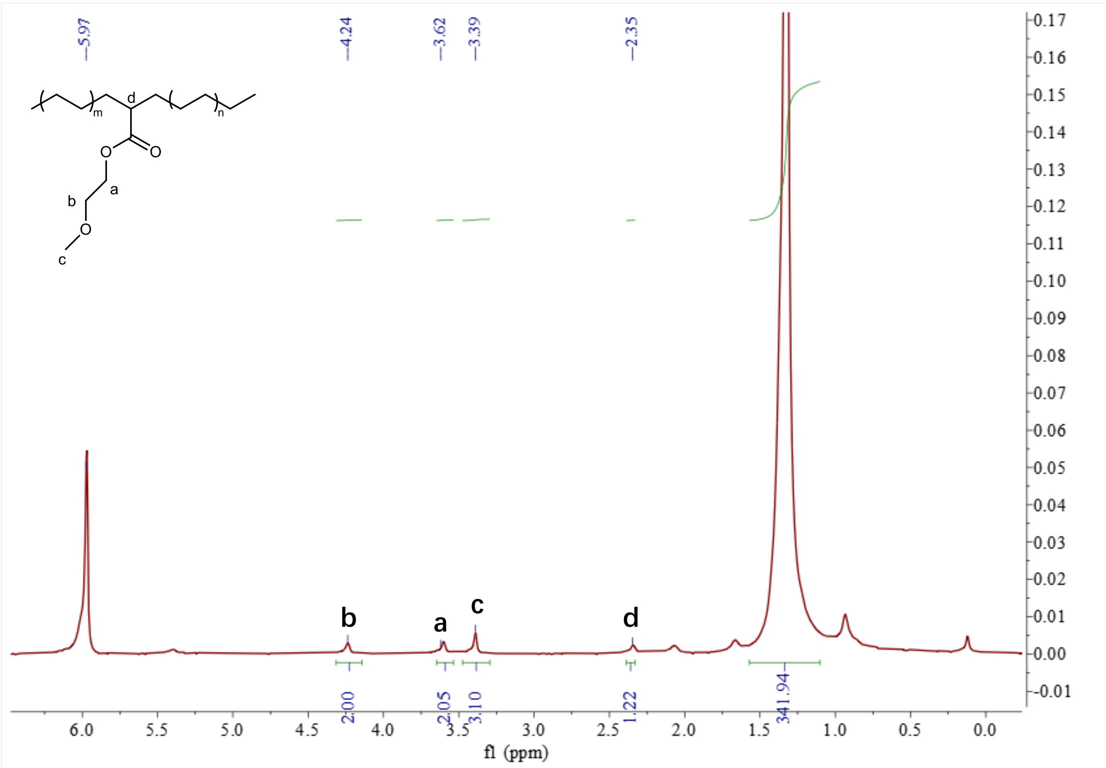

Supplementary Figure 41. ^{1}H NMR spectrum of the polymer from Table 2, Entry 13($C_2D_2Cl_4$, 120°C).

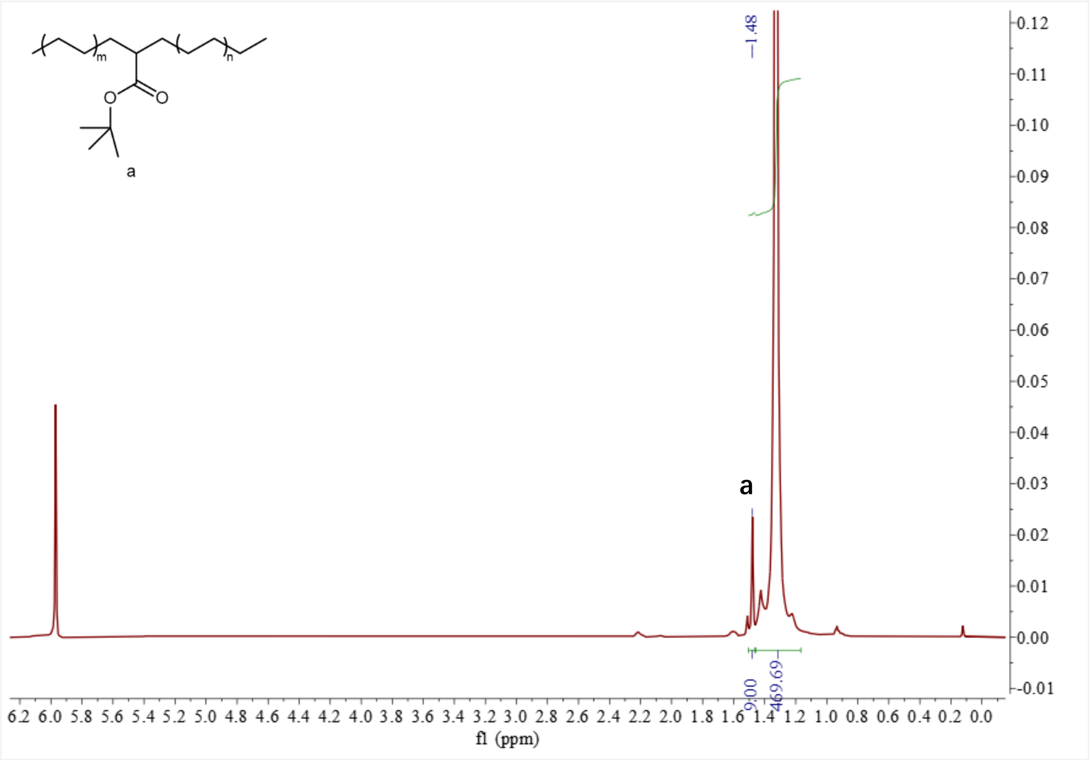

Supplementary Figure 42. ^{1}H NMR spectrum of the polymer from Table 2, Entry 14 ($C_2D_2Cl_4$, 120°C).

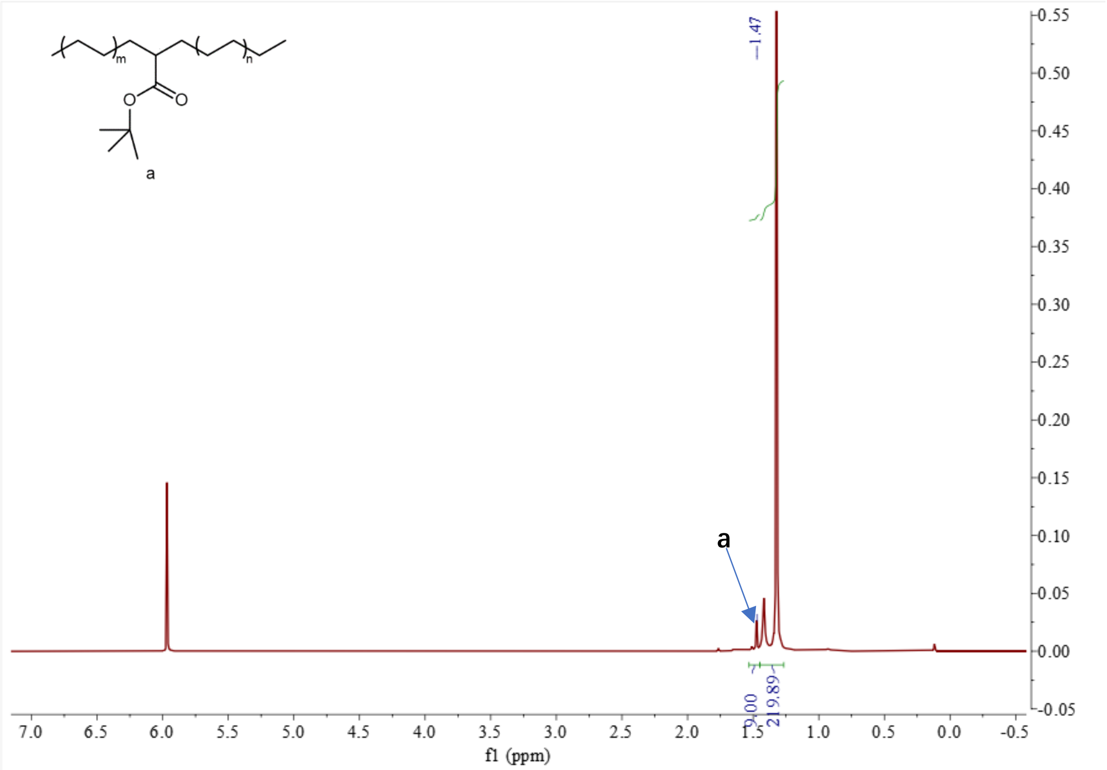

Supplementary Figure 43. ^{1}H NMR spectrum of the polymer from Table 2, Entry 15 ($C_2D_2Cl_4$, 120°C).

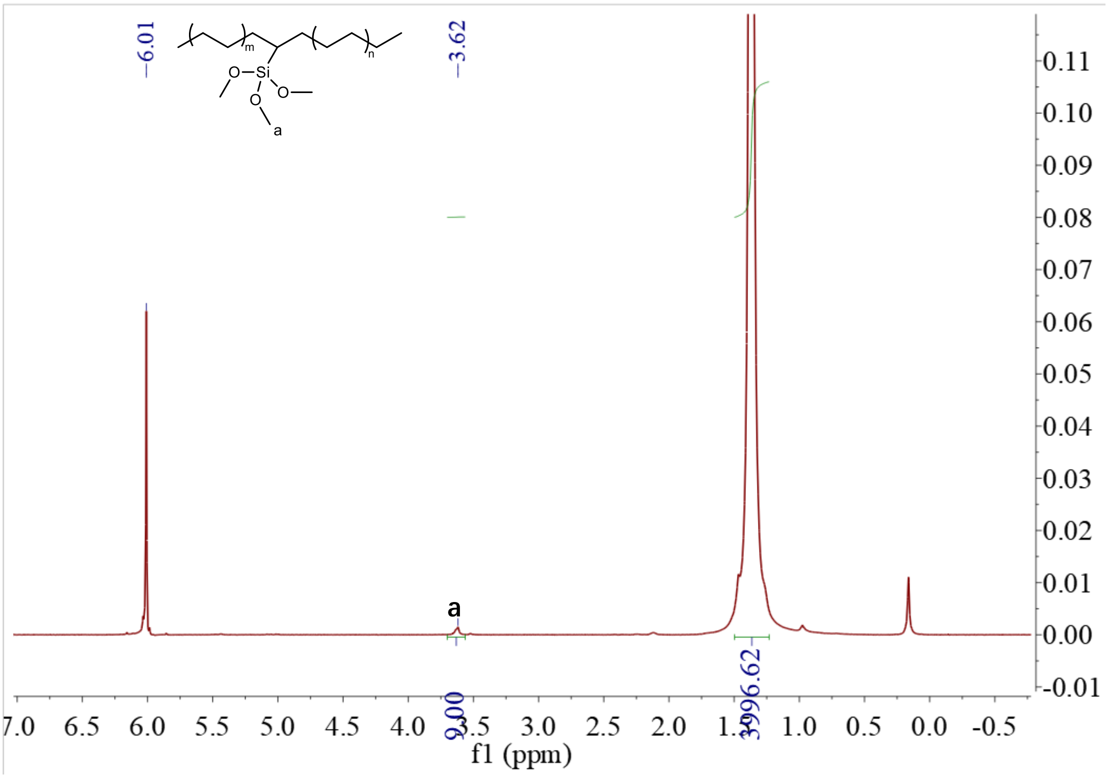

Supplementary Figure 44. ^{1}H NMR spectrum of the polymer from Table 2, Entry 16 ($C_2D_2Cl_4$, 120°C).

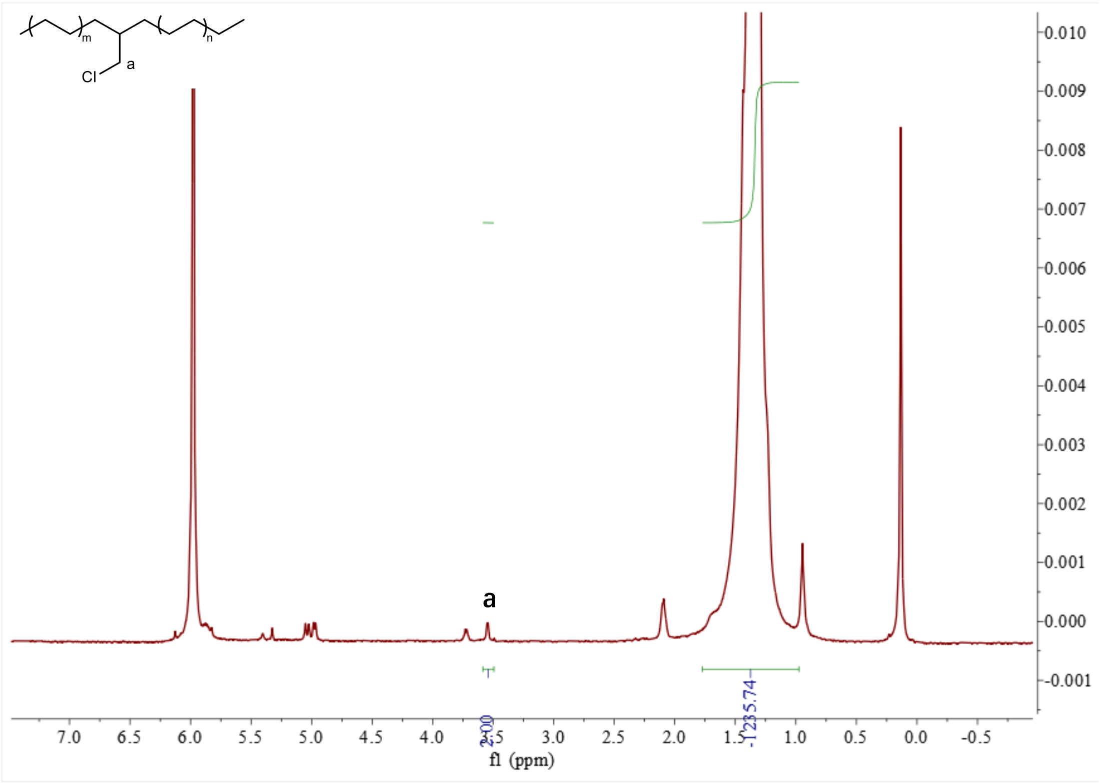

Supplementary Figure 45. ^{1}H NMR spectrum of the polymer from Table 2, Entry 17 ($C_2D_2Cl_4$, 120°C).

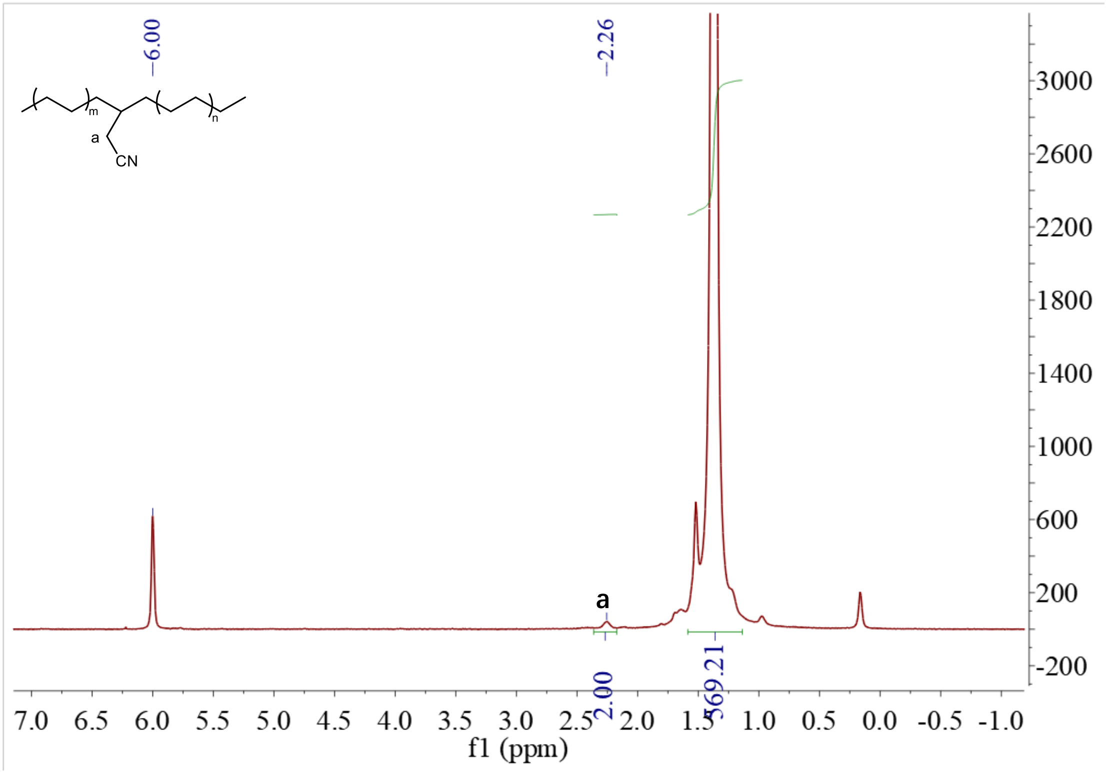

Supplementary Figure 46. ^{1}H NMR spectrum of the polymer from Table 2, Entry 18($C_2D_2Cl_4$, 120°C).

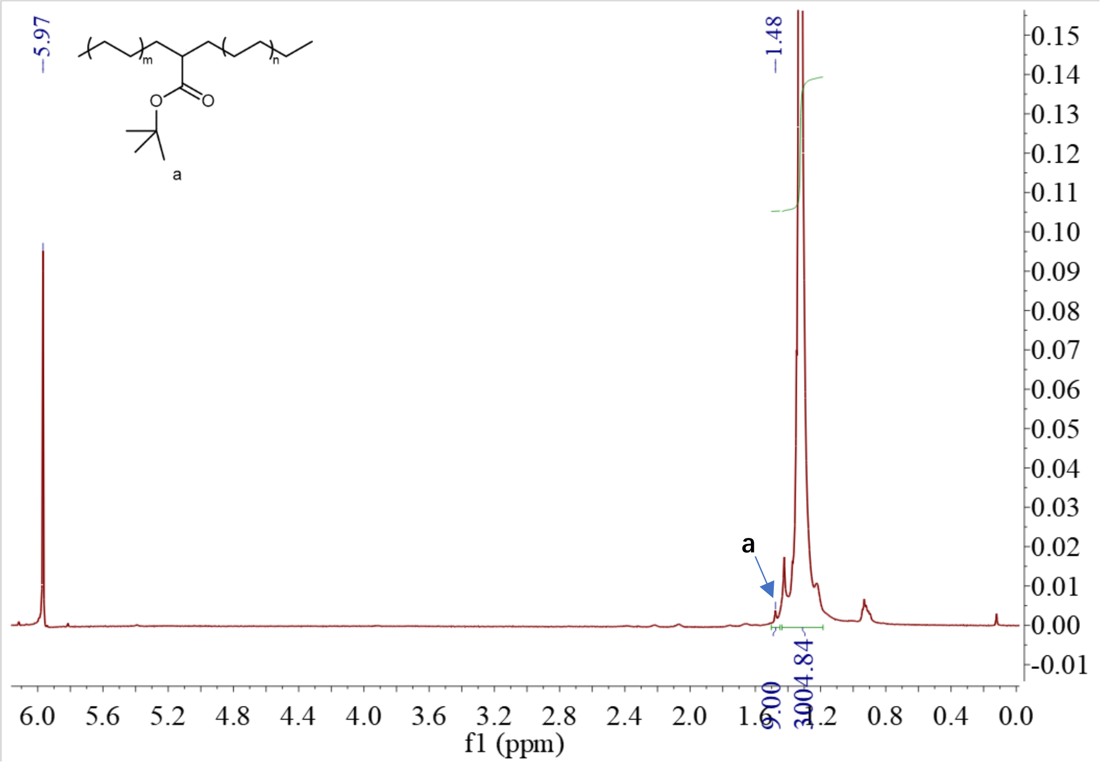

Supplementary Figure 47. ^{1}H NMR spectrum of the polymer from Table 2, Entry 19($C_2D_2Cl_4$, 120°C).

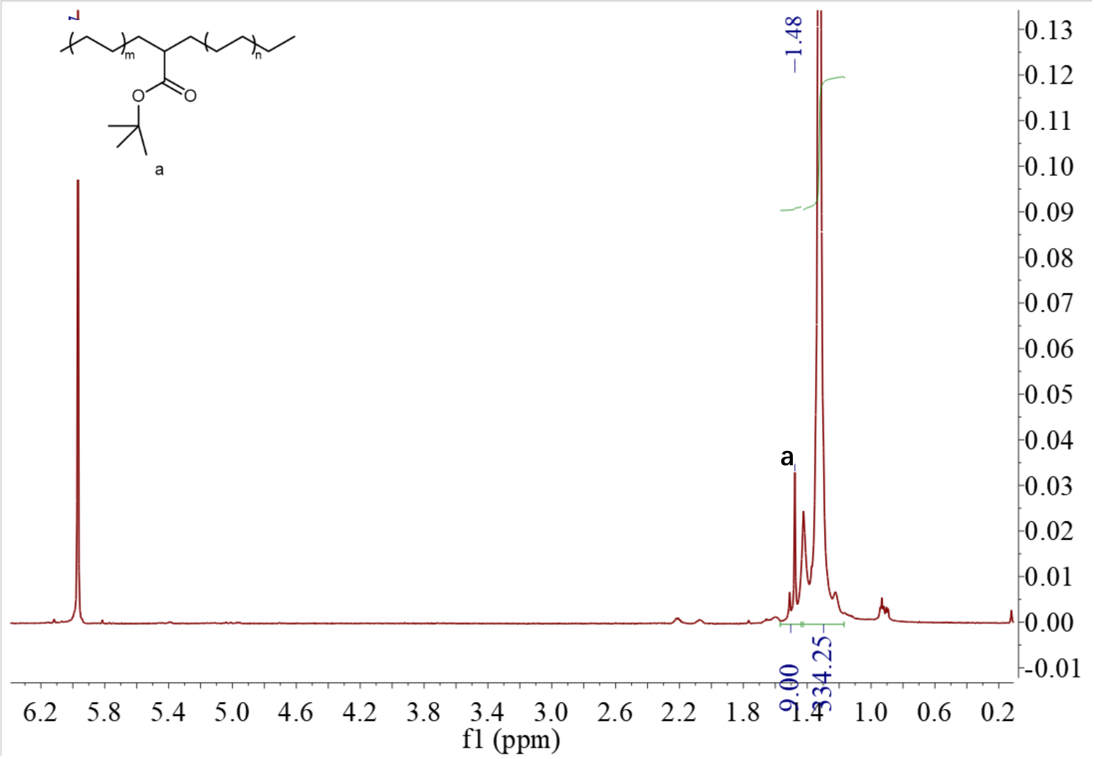

Supplementary Figure 48. ^{1}H NMR spectrum of the polymer from Table 2, Entry 20($C_2D_2Cl_4$, 120°C).

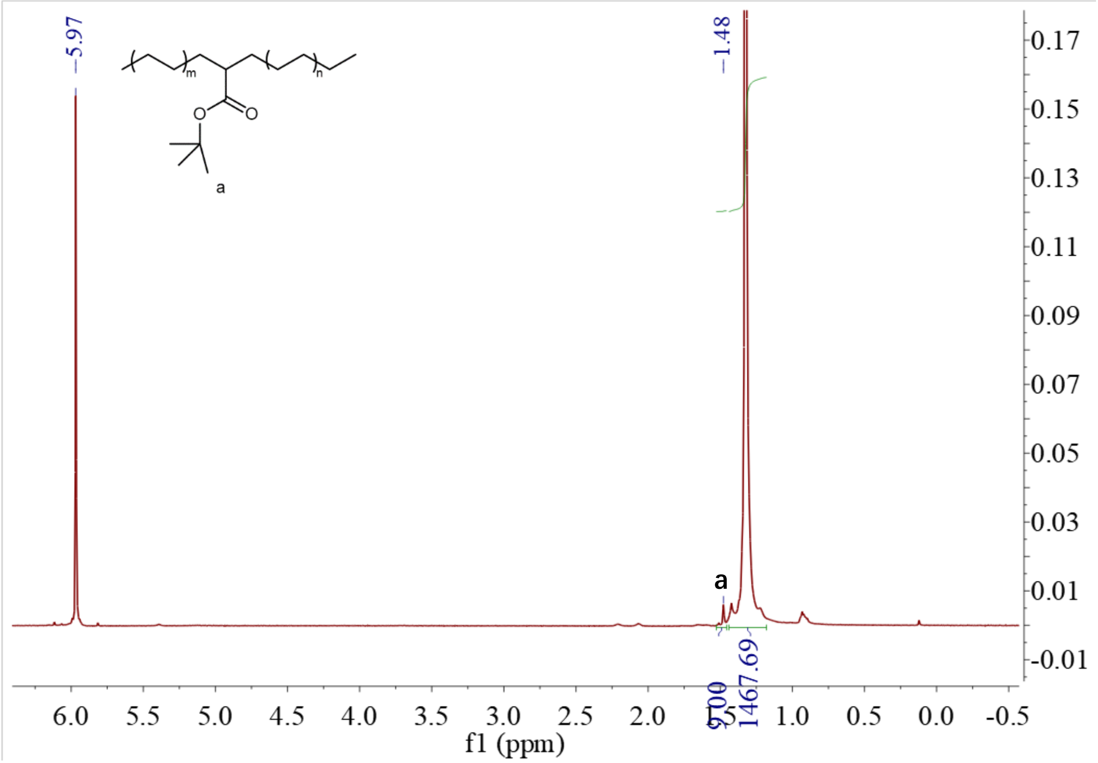

Supplementary Figure 49. ^{1}H NMR spectrum of the polymer from Table 2, Entry 21($C_2D_2Cl_4$, 120°C).

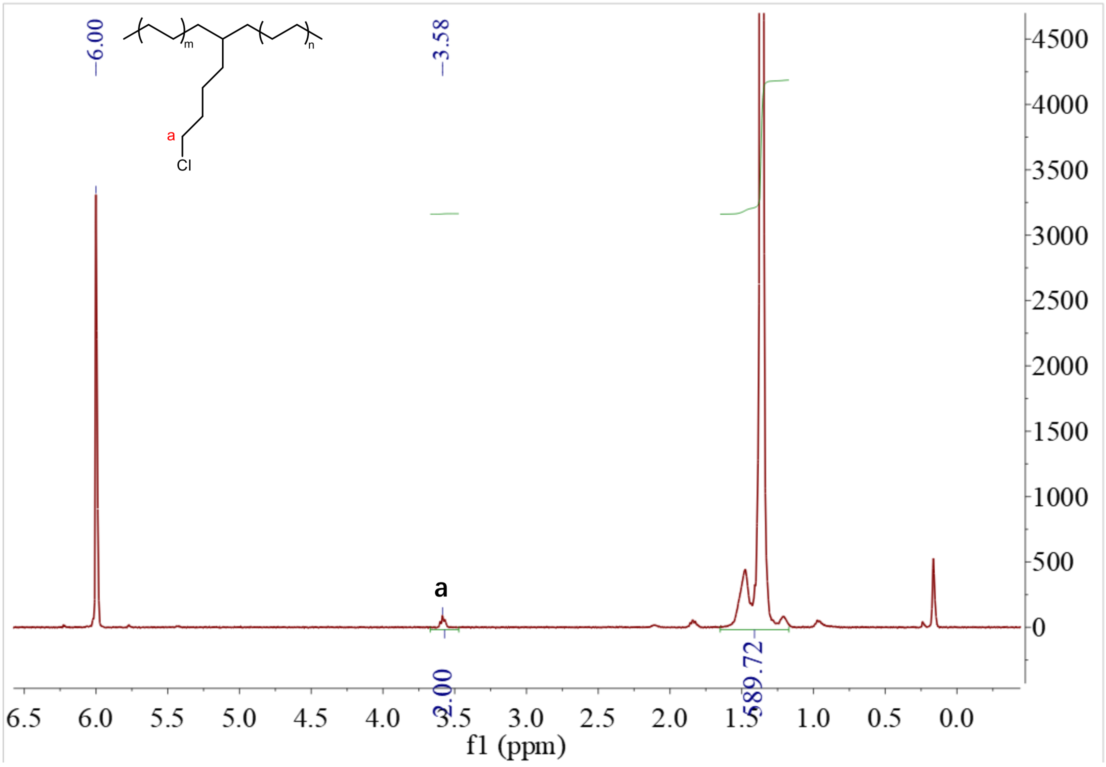

Supplementary Figure 50. ^{1}H NMR spectrum of the polymer from Table 2, Entry 22 ($C_2D_2Cl_4$, 120°C).

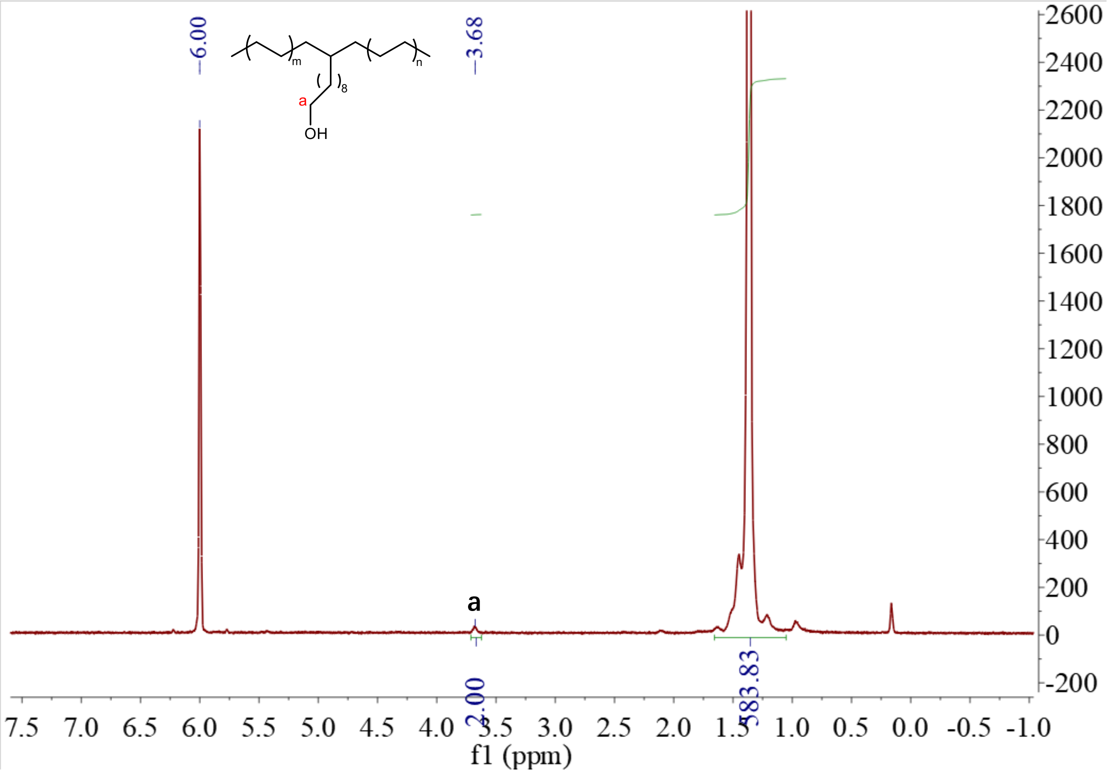

Supplementary Figure 51. ^{1}H NMR spectrum of the polymer from Table 2, Entry 23 ($C_2D_2Cl_4$, 120°C).

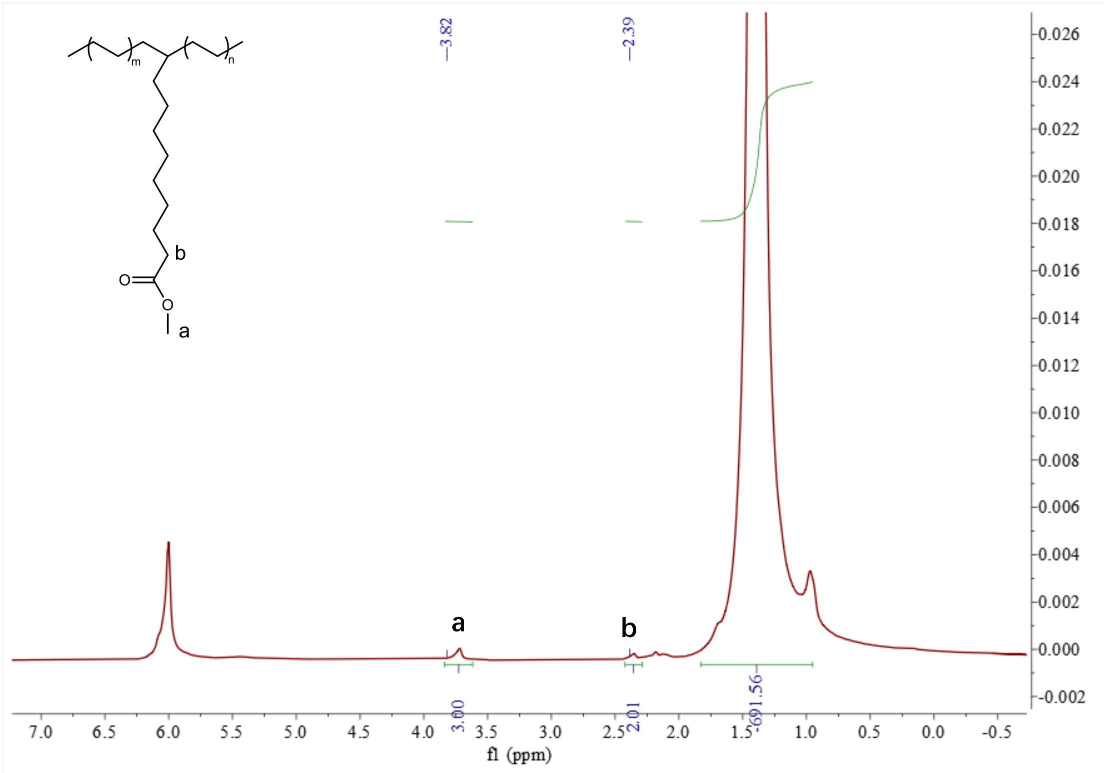

Supplementary Figure 52. ^{1}H NMR spectrum of the polymer from Table 2, Entry 24 ($C_2D_2Cl_4$, 120°C).

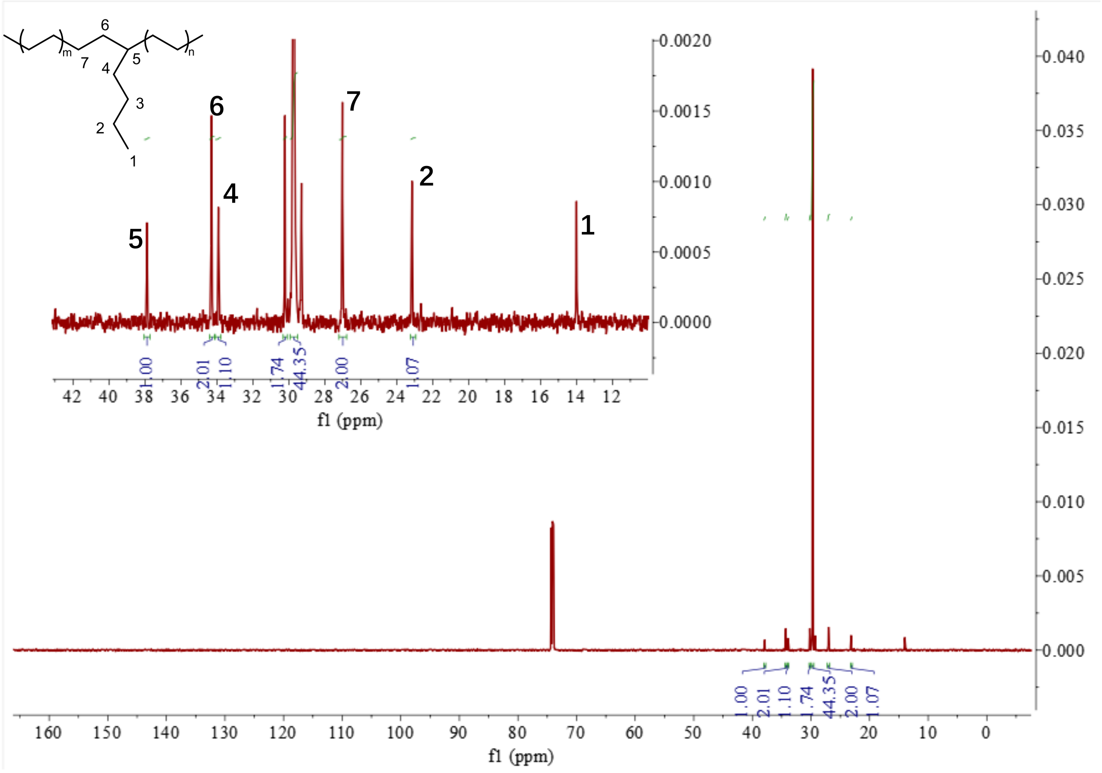

Supplementary Figure 53. ^{13}C NMR spectrum of the polymer from Table 3, Entry 9 ($C_2D_2Cl_4$, 120°C).

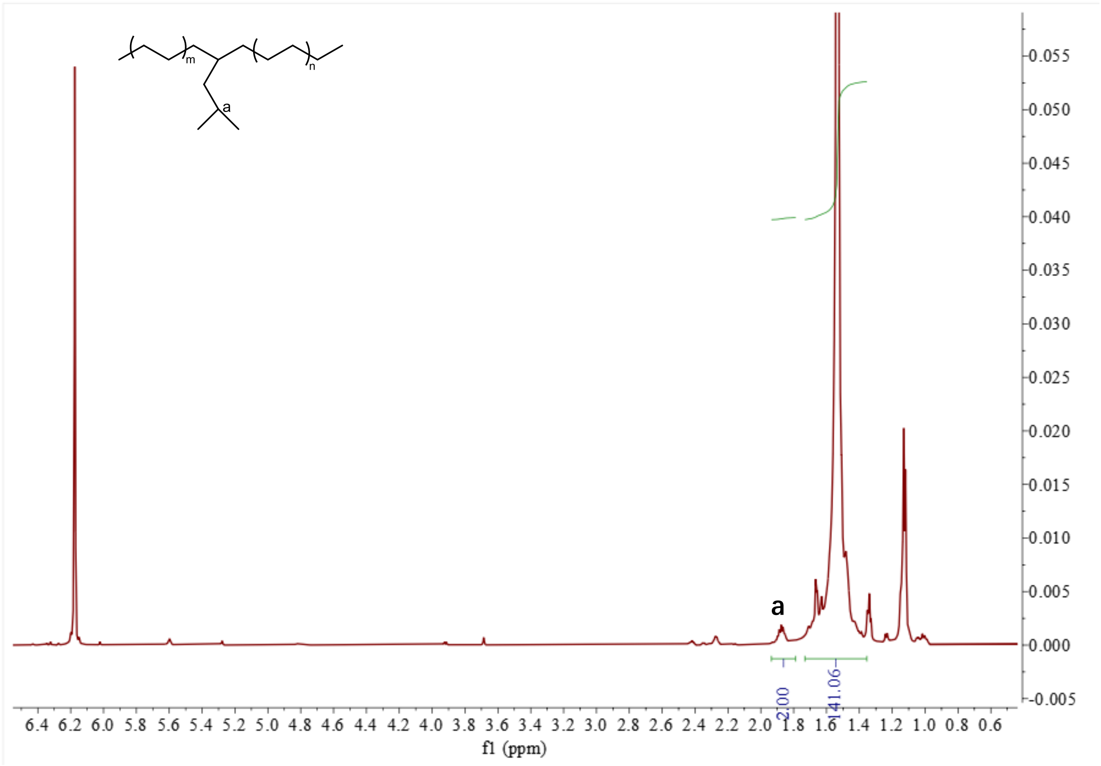

Supplementary Figure 54. ^{1}H NMR spectrum of the polymer from Table 3, Entry 13 ($C_2D_2Cl_4$, 120°C).

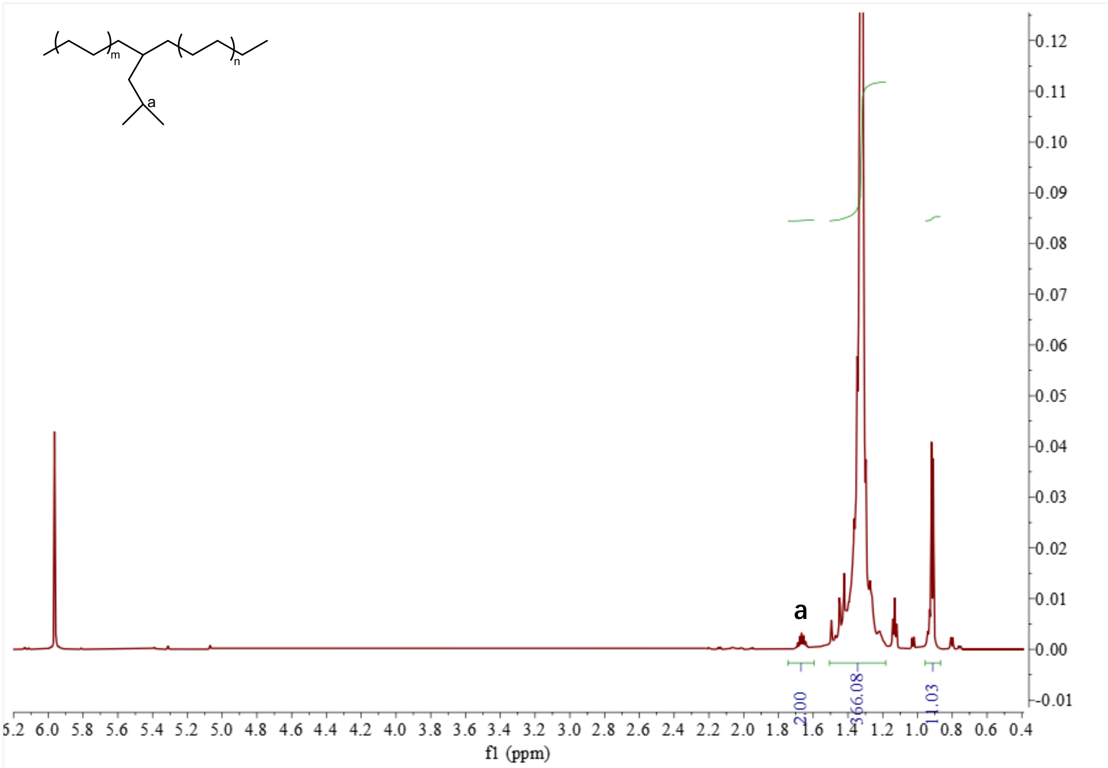

Supplementary Figure 55. ^{1}H NMR spectrum of the polymer from Table 3, Entry 14 ($C_2D_2Cl_4$, 120°C).

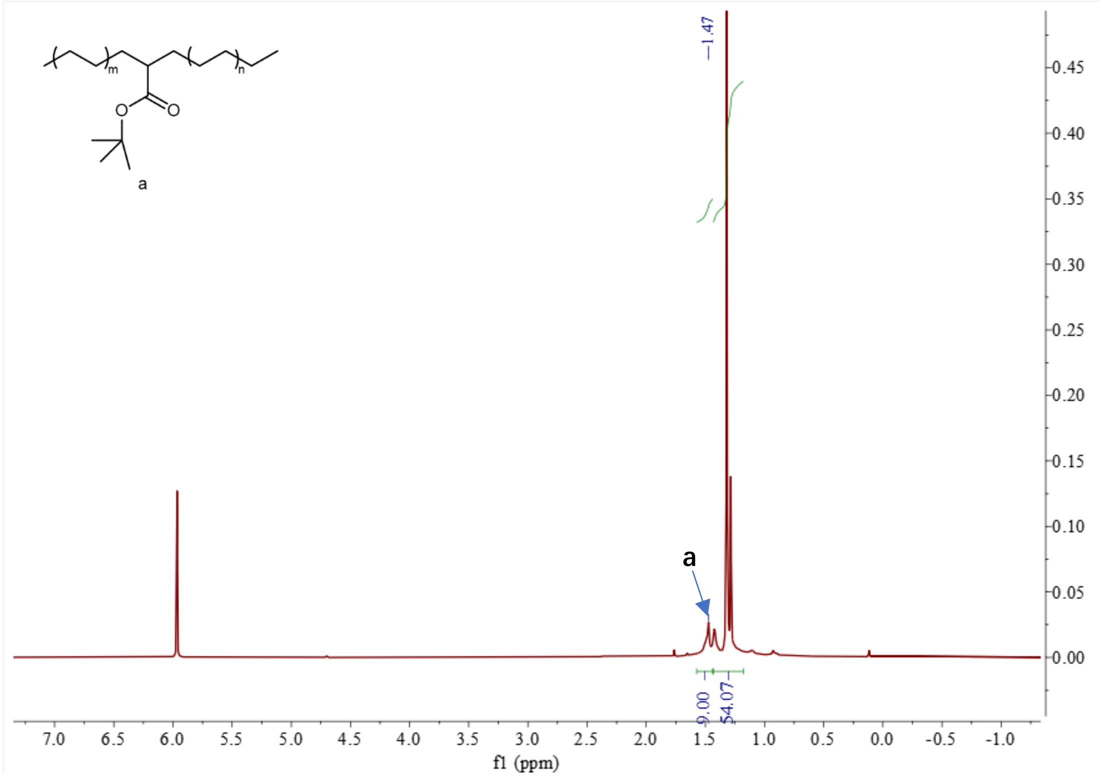

Supplementary Figure 56. ^{1}H NMR spectrum of the polymer from Table 4, Entry 5 ($C_2D_2Cl_4$, 120°C).

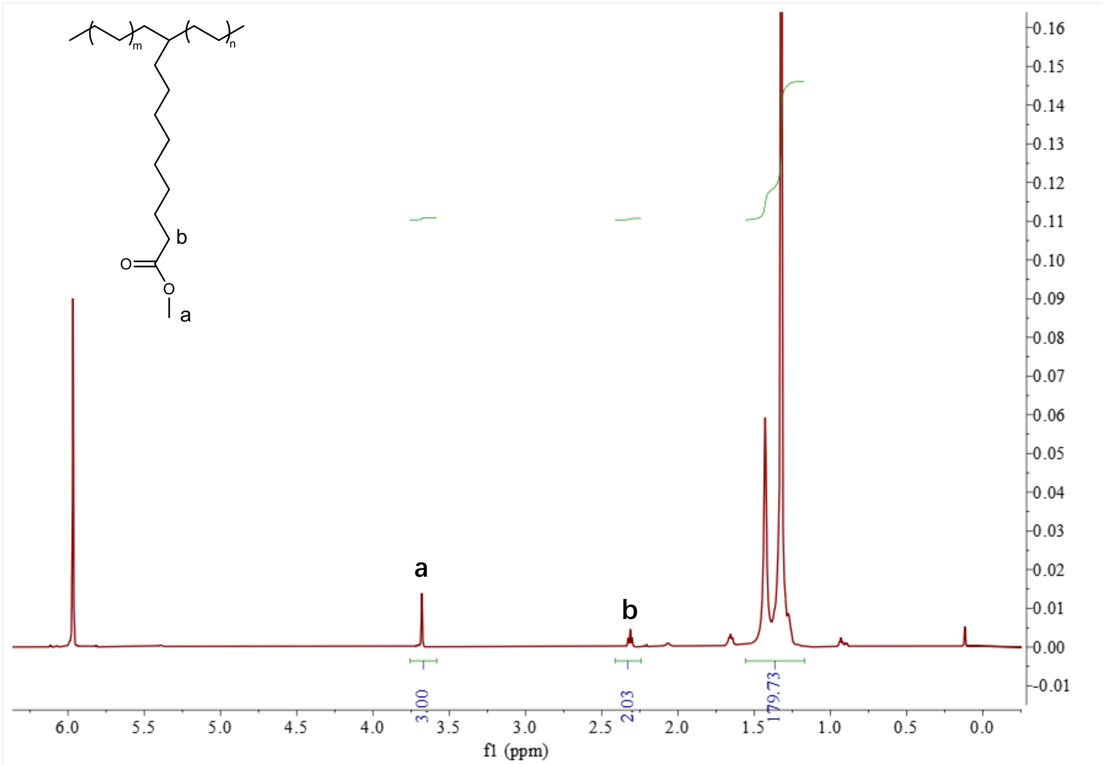

Supplementary Figure 57. ^{1}H NMR spectrum of the polymer from Table 4, Entry 9 ($C_2D_2Cl_4$, 120°C).

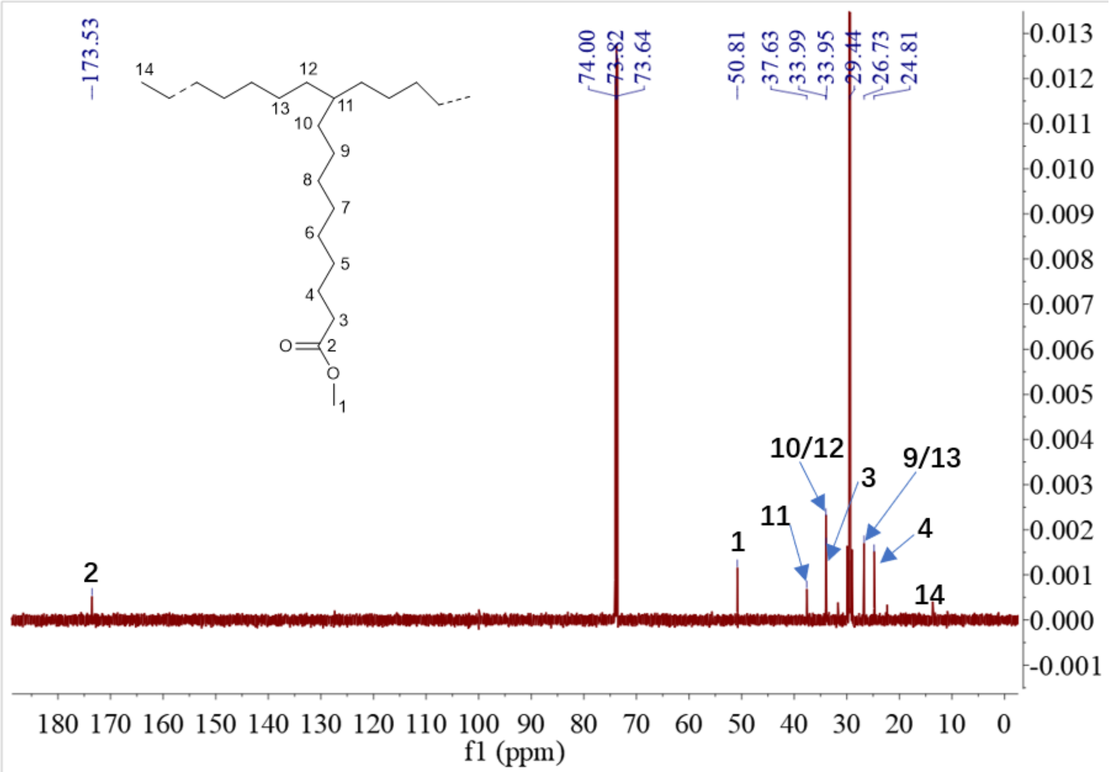

Supplementary Figure 58. ^{13}C NMR spectrum of the polymer from Table 4, Entry 9 ($C_2D_2Cl_4$, 120°C).

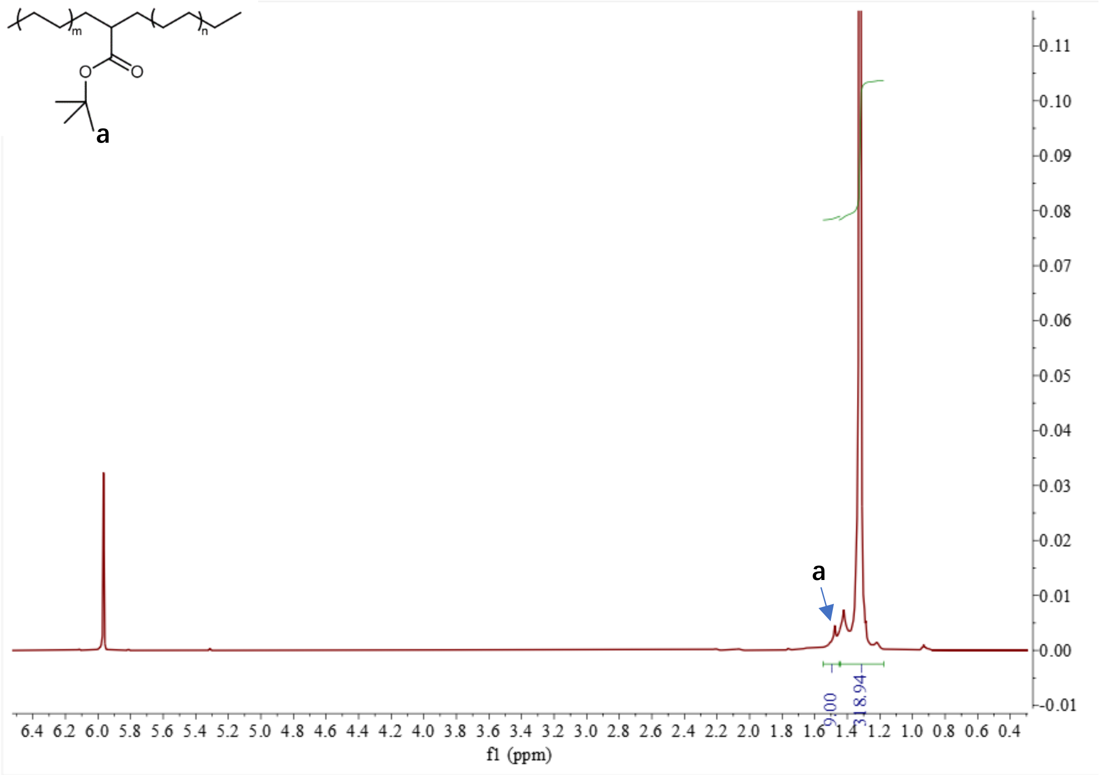

Supplementary Figure 59. ^{1}H NMR spectrum of the polymer from Table 4, Entry 13 ($C_2D_2Cl_4$, 120°C).

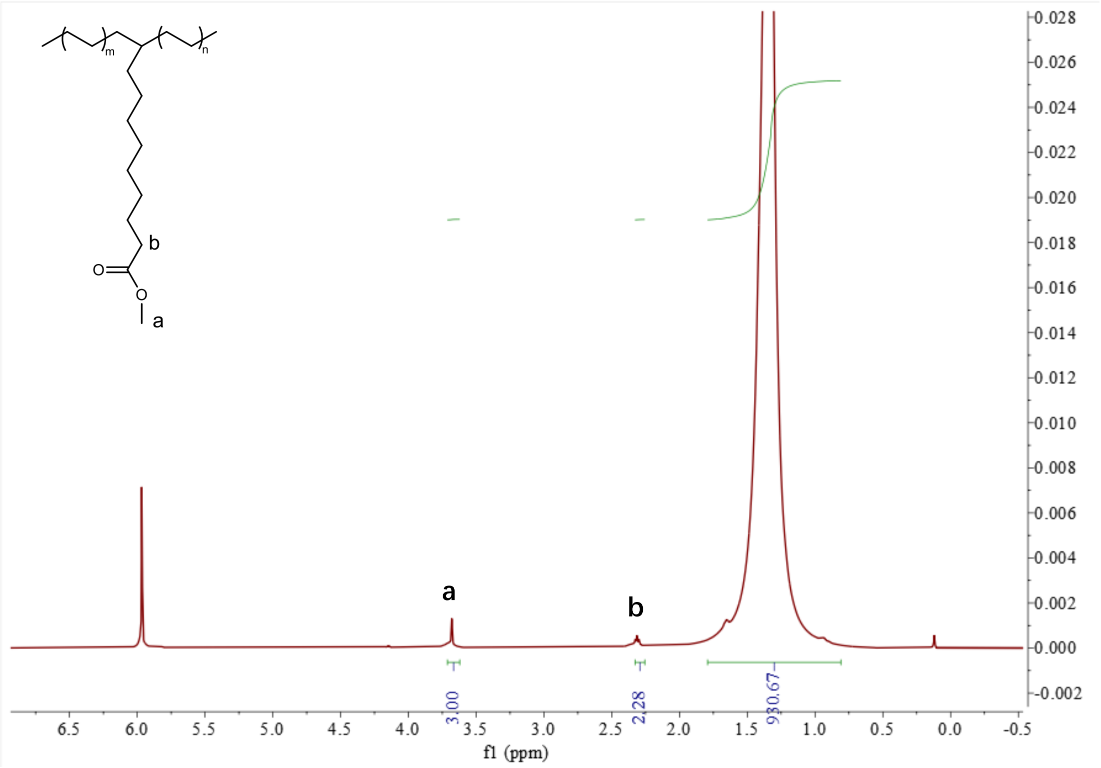

Supplementary Figure 60. ^{1}H NMR spectrum of the polymer from Table 4, Entry 14 ($C_2D_2Cl_4$, 120°C).

5 Supplementary Figures of DSC of copolymers.

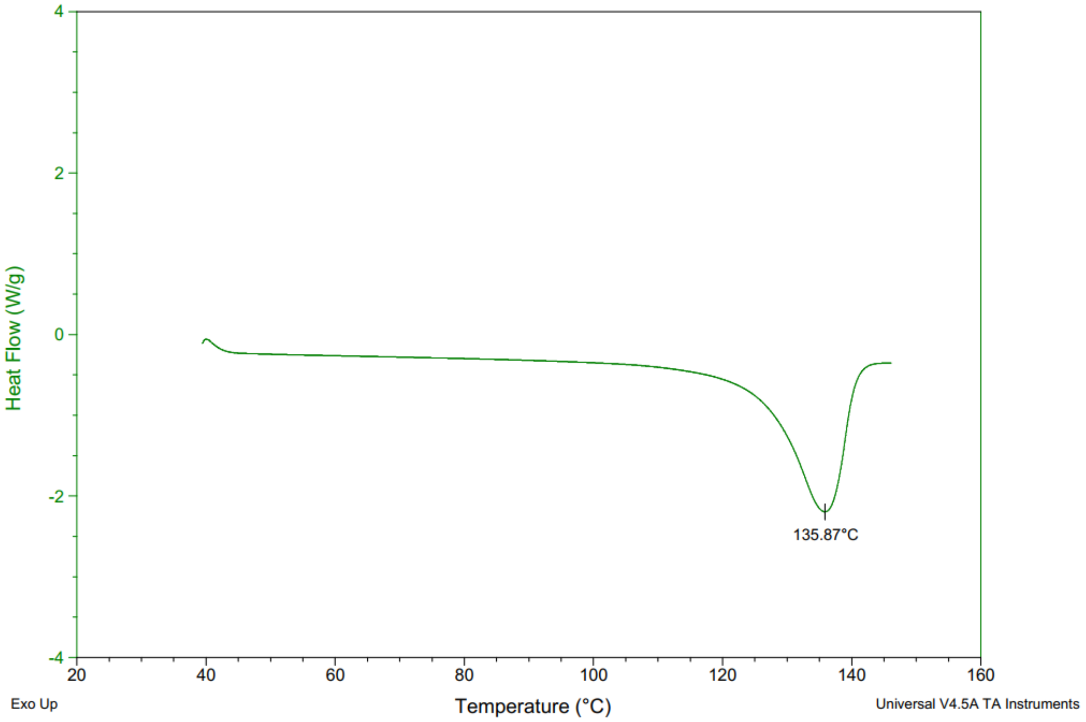

Supplementary Figure 61. DSC of the copolymer from Table 1, Entry 1.

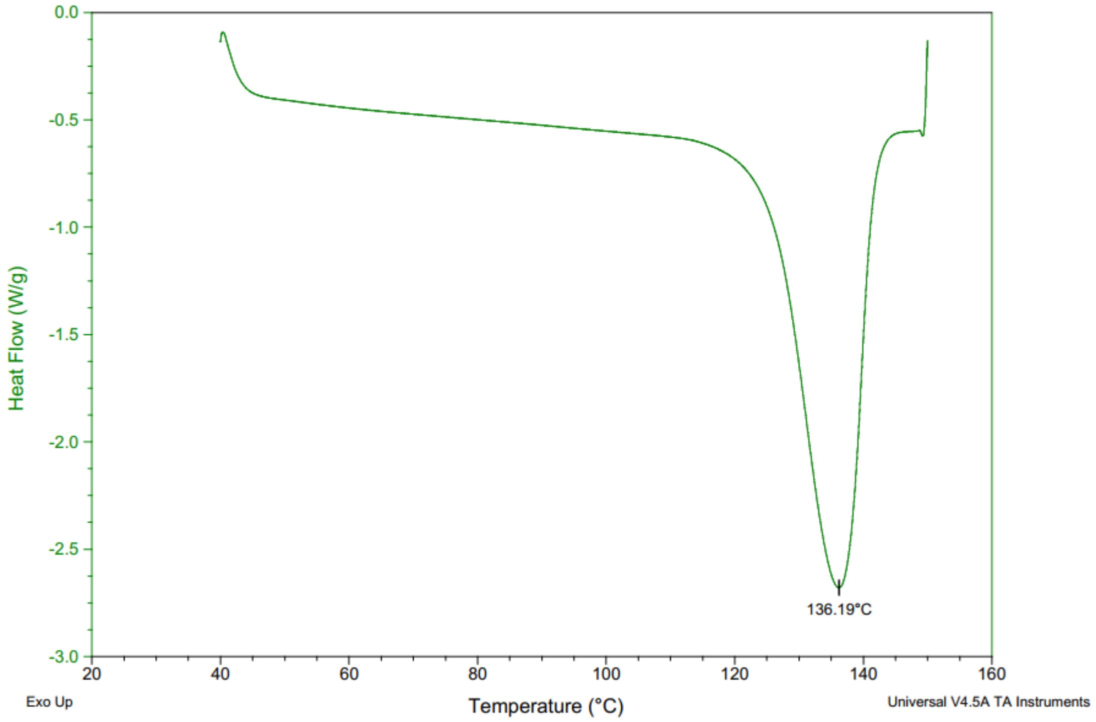

Supplementary Figure 62. DSC of the copolymer from Table 1, Entry 2.

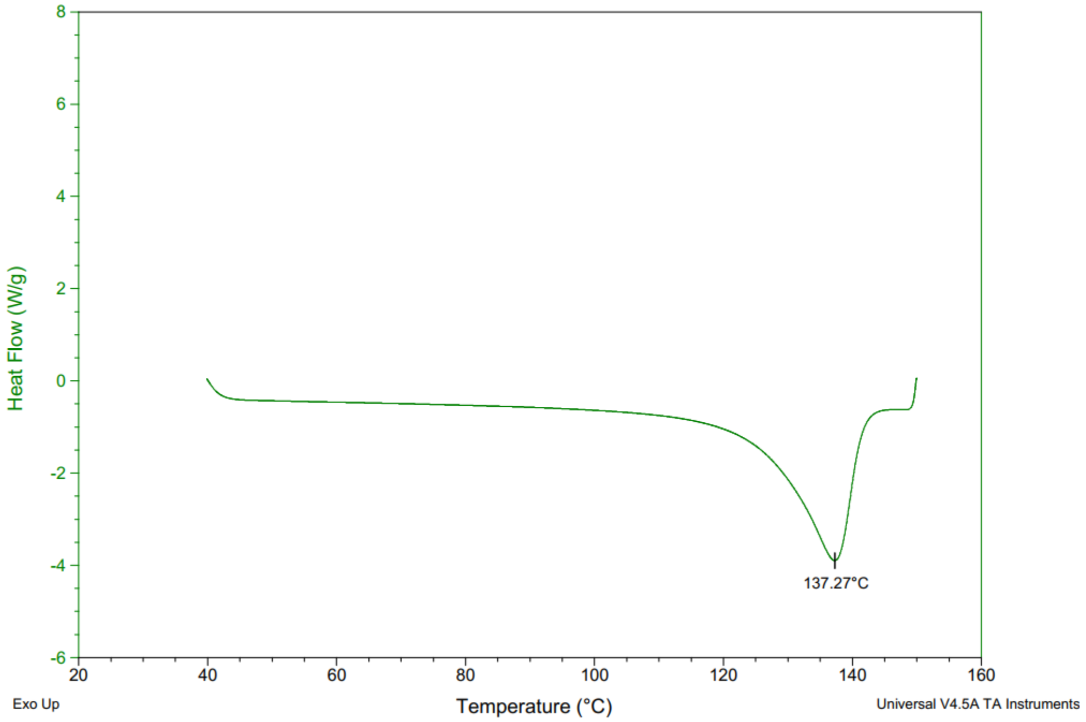

Supplementary Figure 63. DSC of the copolymer from Table 1, Entry 3.

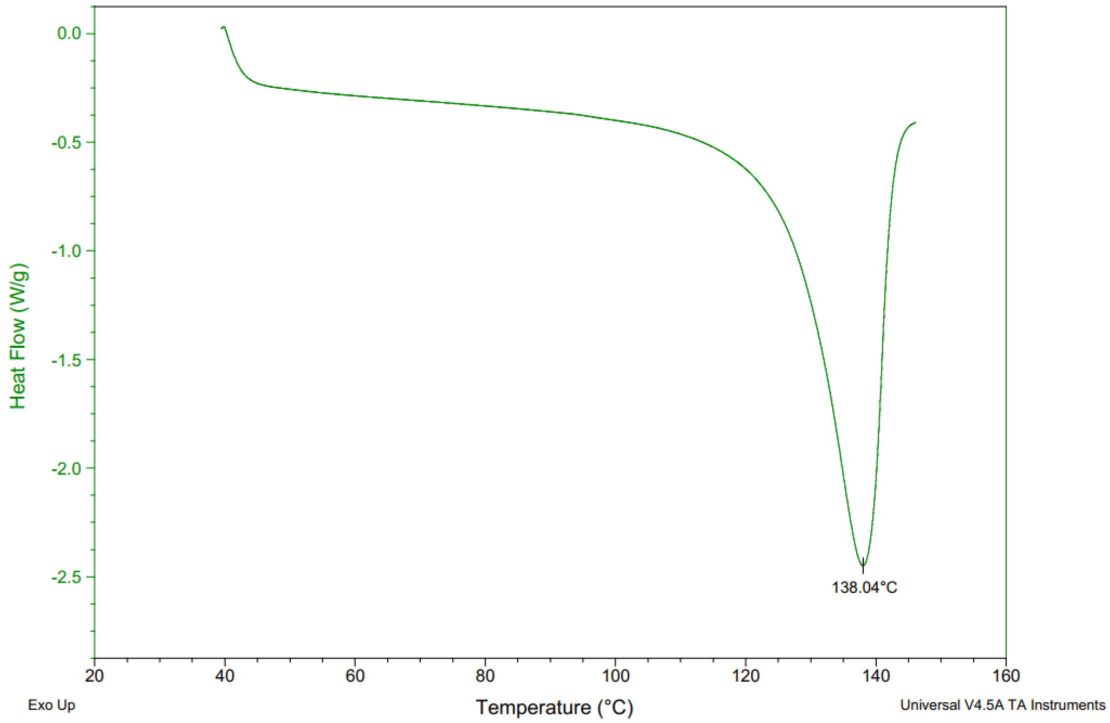

Supplementary Figure 64. DSC of the copolymer from Table 1, Entry 4.

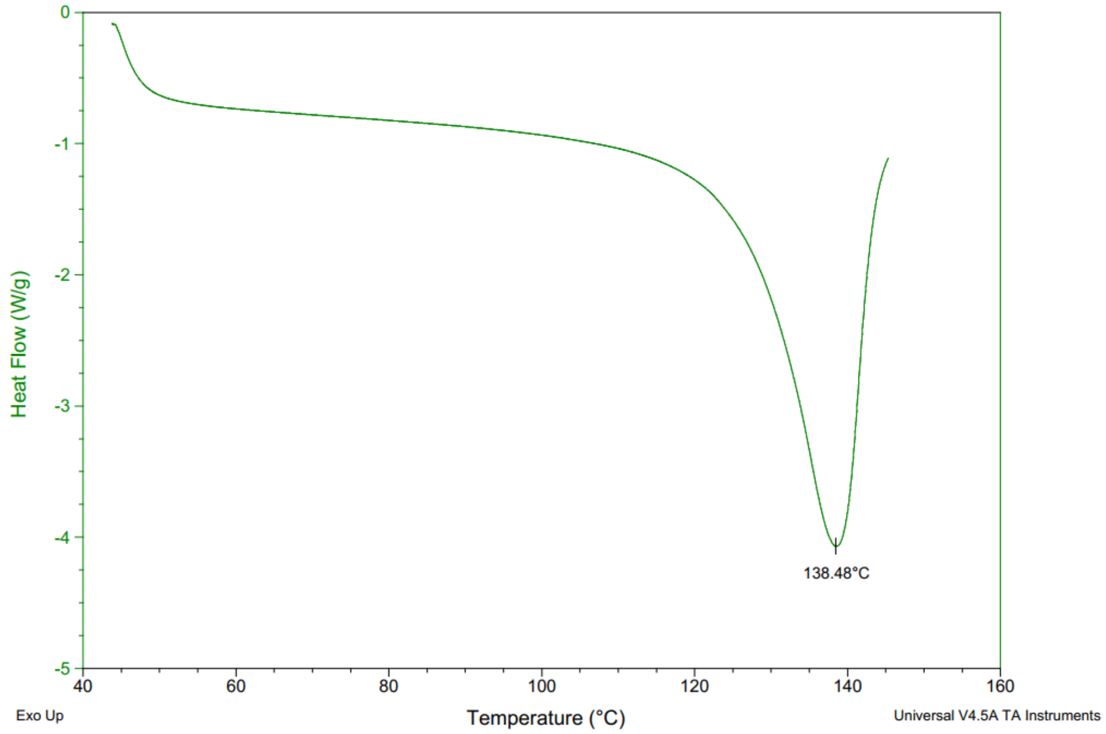

Supplementary Figure 65. DSC of the copolymer from Table 1, Entry 6.

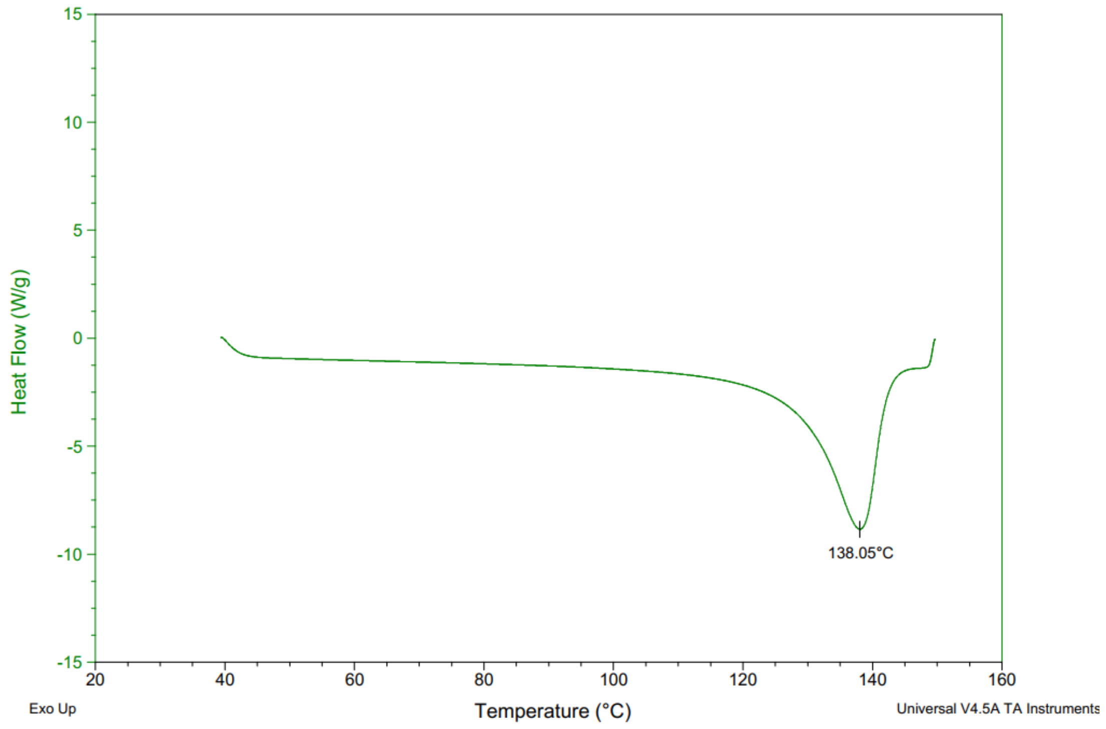

Supplementary Figure 66. DSC of the copolymer from Table 1, Entry 7.

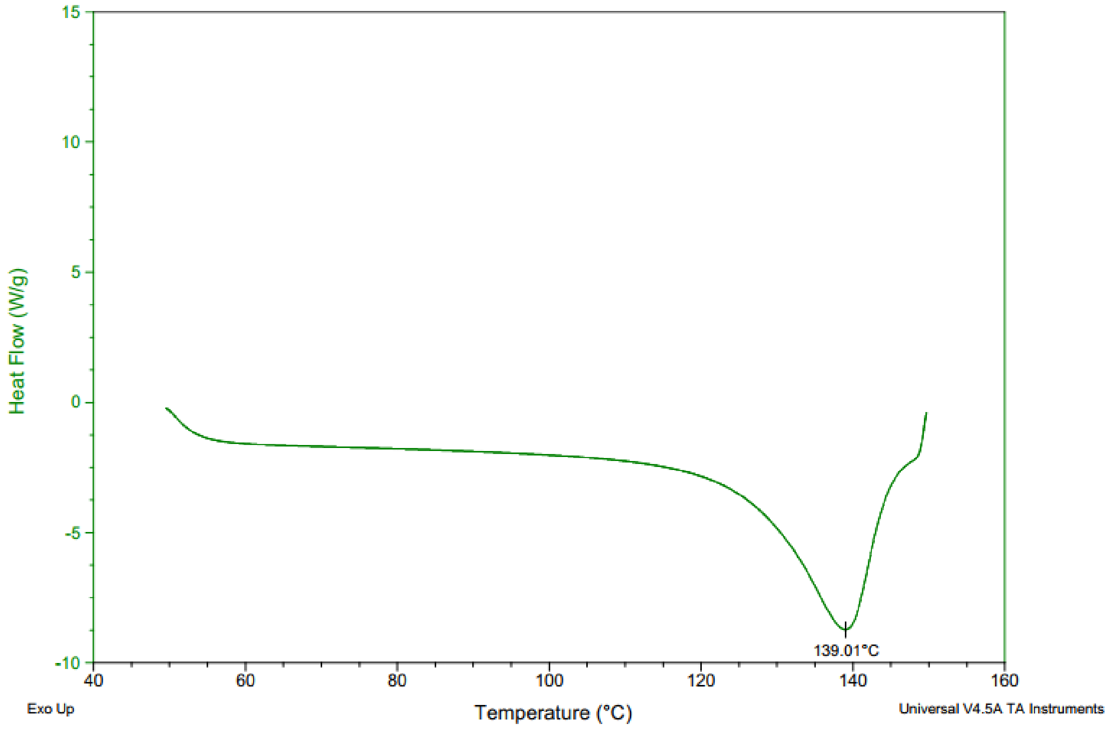

Supplementary Figure 67. DSC of the copolymer from Table 1, Entry 8.

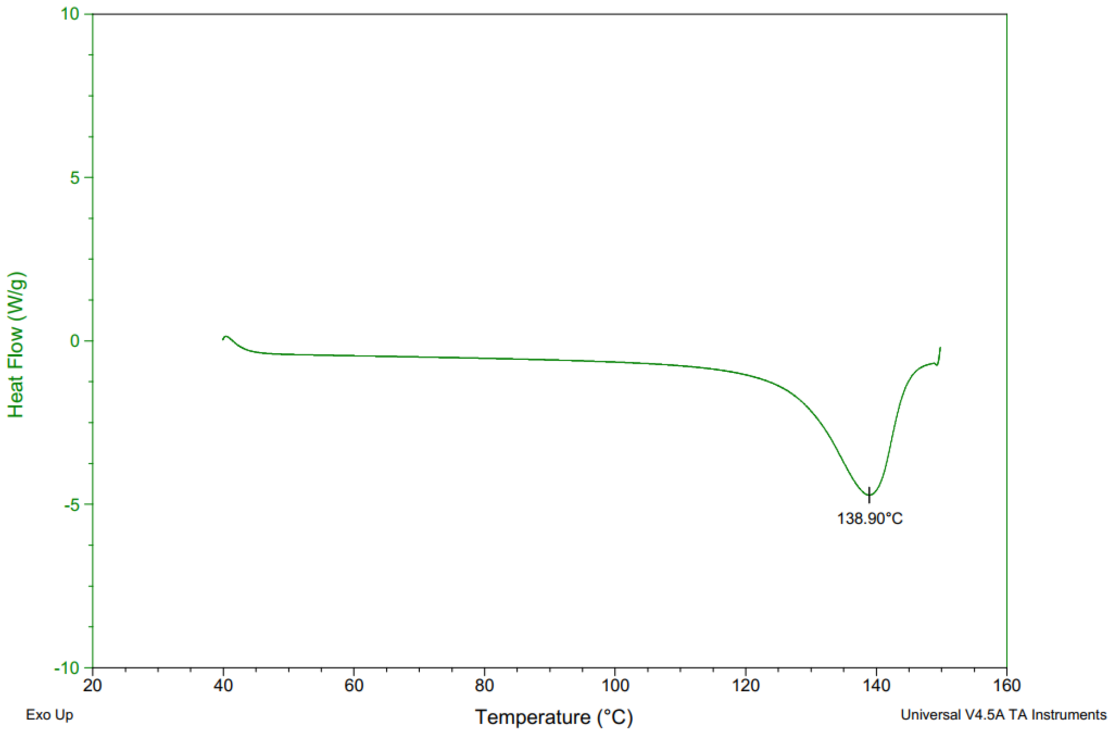

Supplementary Figure 68. DSC of the copolymer from Table 1, Entry 9.

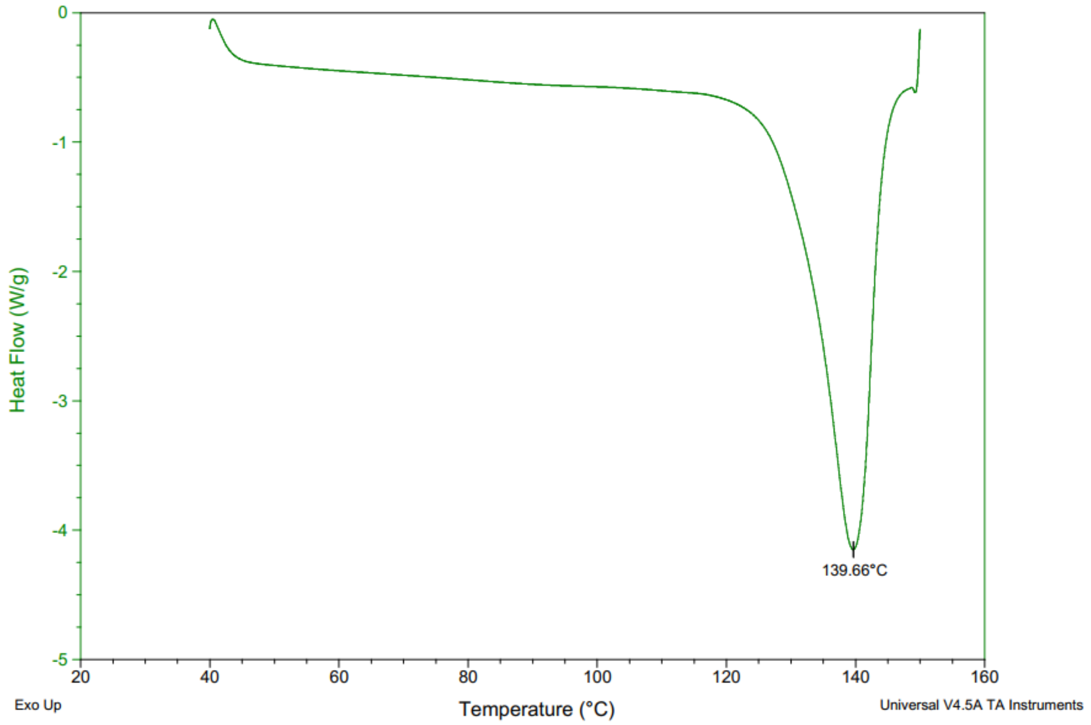

Supplementary Figure 69. DSC of the copolymer from Table 1, Entry 10.

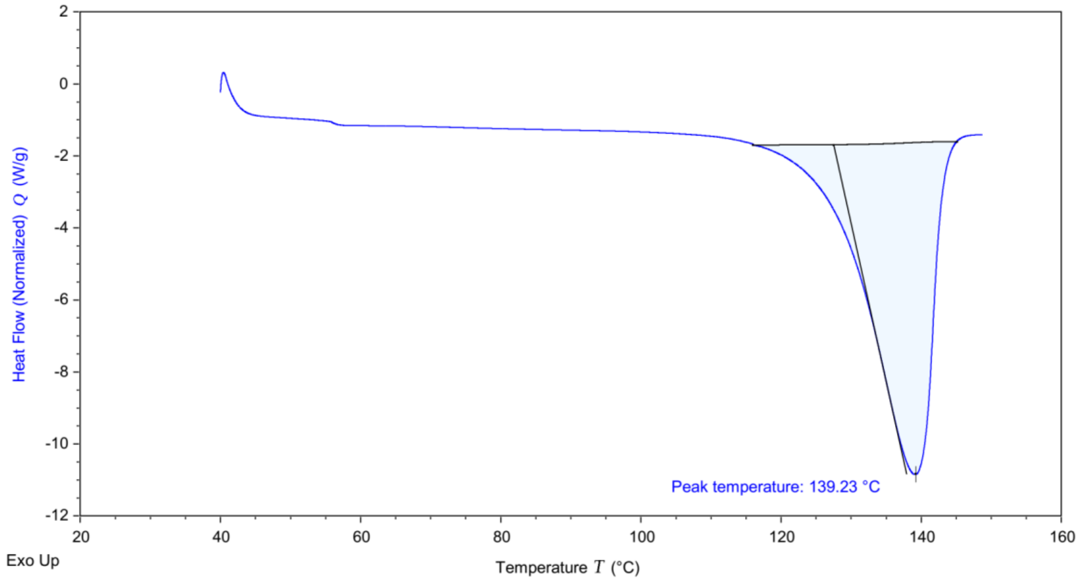

Supplementary Figure 70. DSC of the copolymer from Table 1, Entry 11.

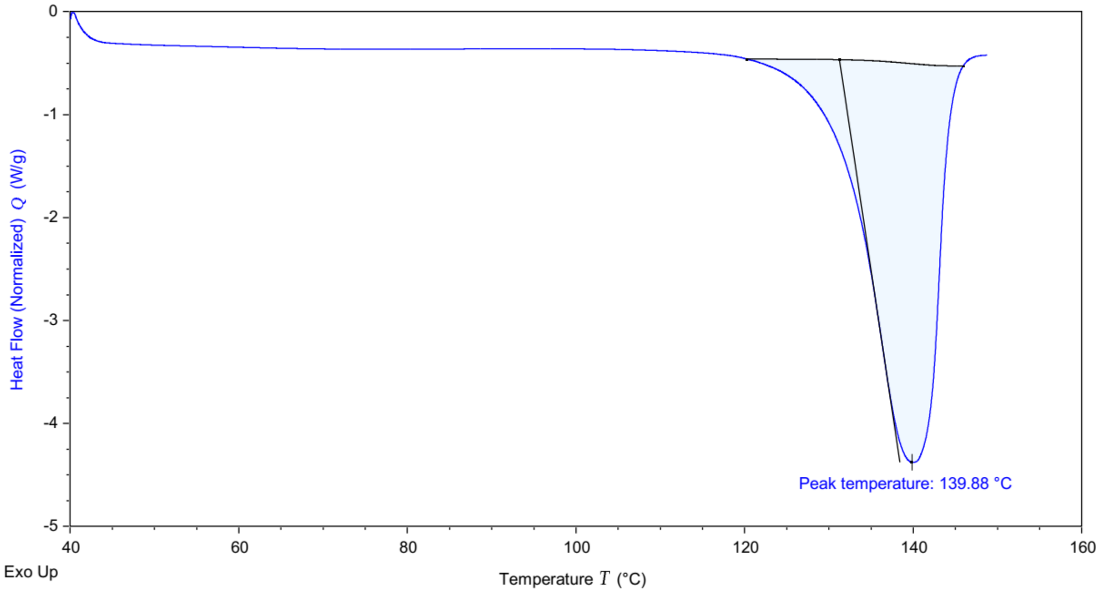

Supplementary Figure 71. DSC of the copolymer from Table 1, Entry 12.

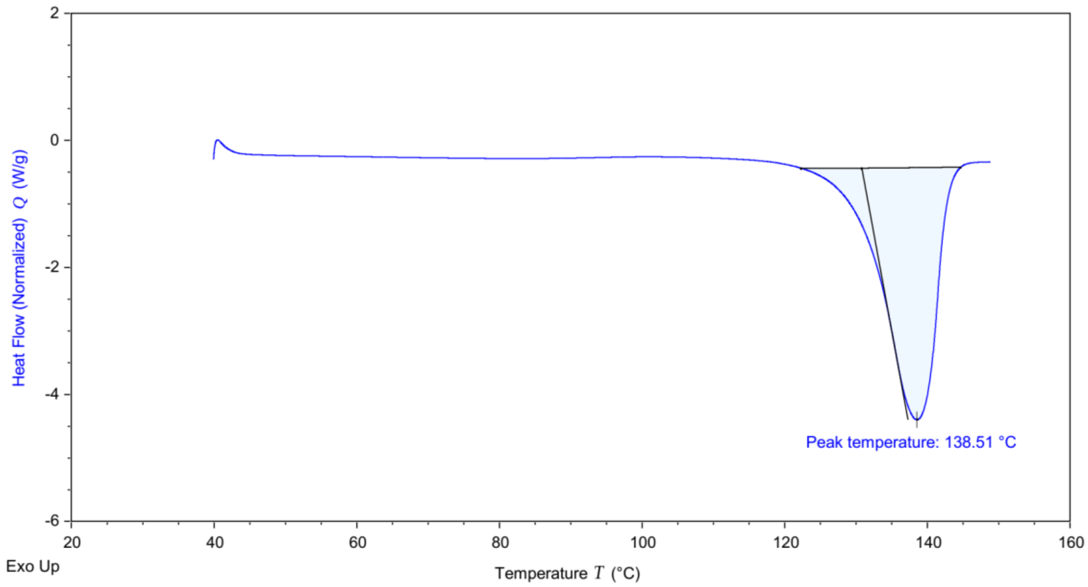

Supplementary Figure 72. DSC of the copolymer from Table 1, Entry 13.

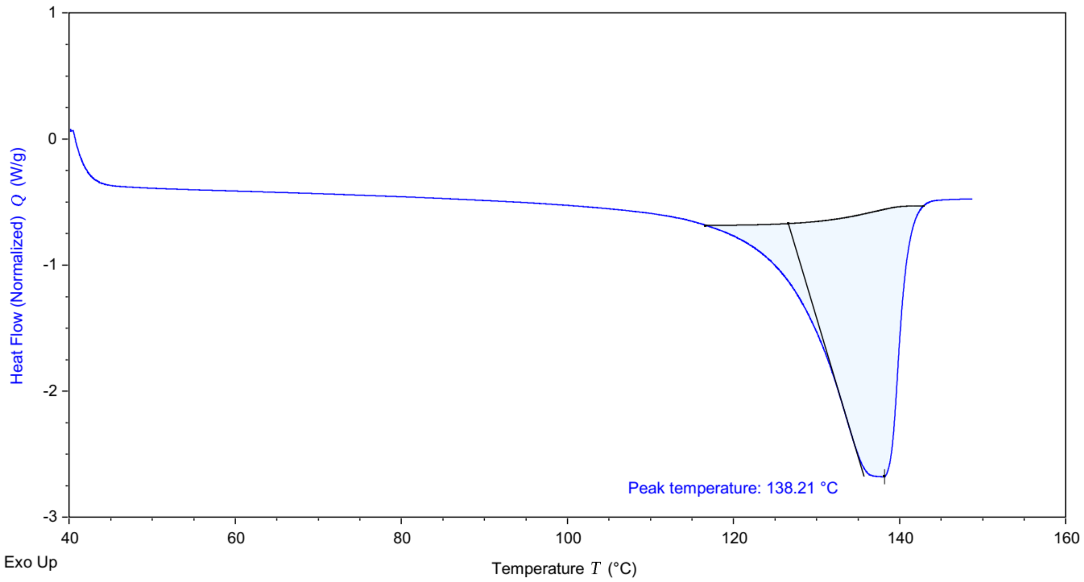

Supplementary Figure 73. DSC of the copolymer from Table 1, Entry 14.

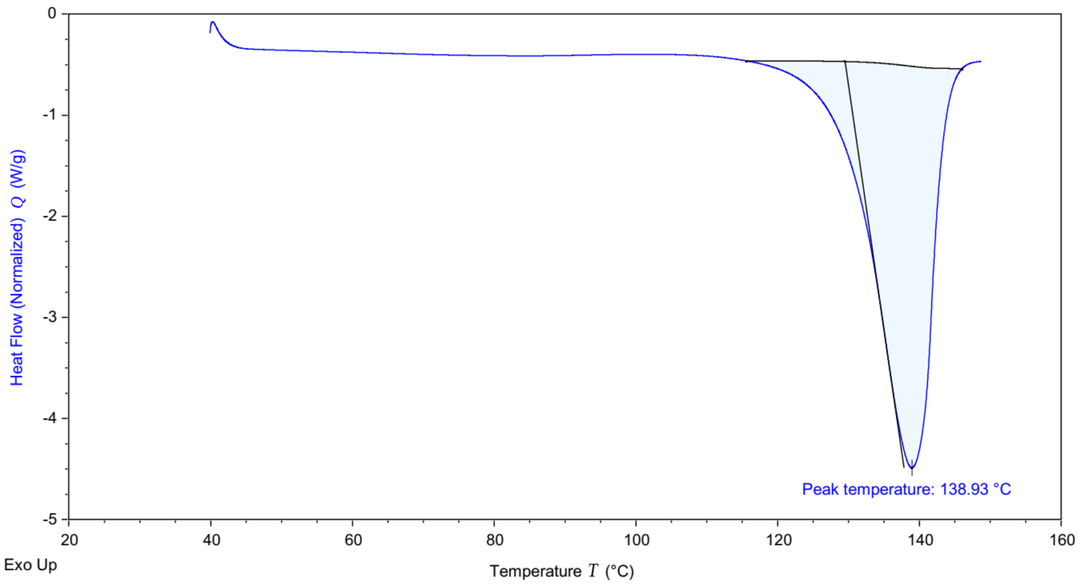

Supplementary Figure 74. DSC of the copolymer from Table 1, Entry 15.

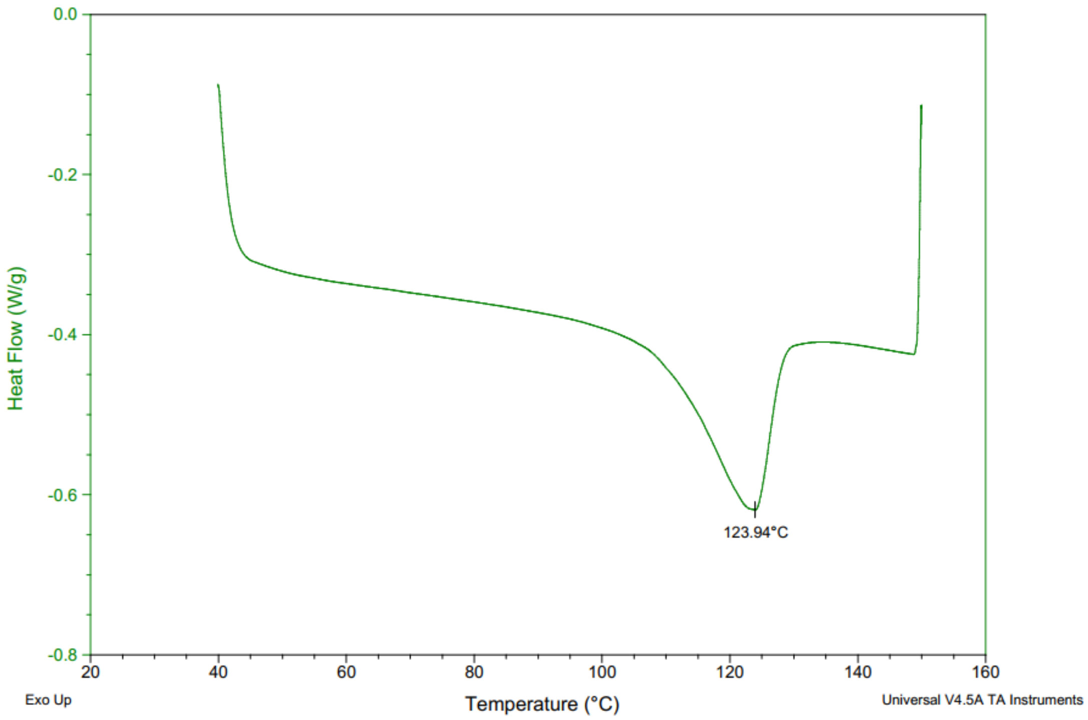

Supplementary Figure 75. DSC of the copolymer from Table 2, Entry 1.

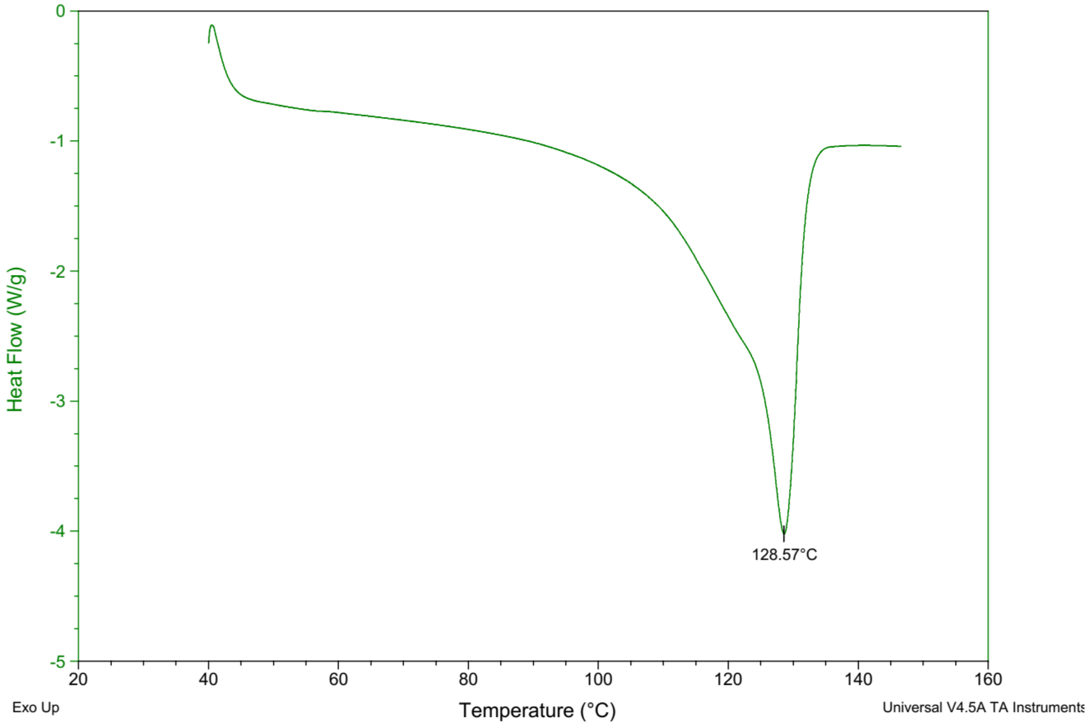

Supplementary Figure 76. DSC of the copolymer from Table 2, Entry 2.

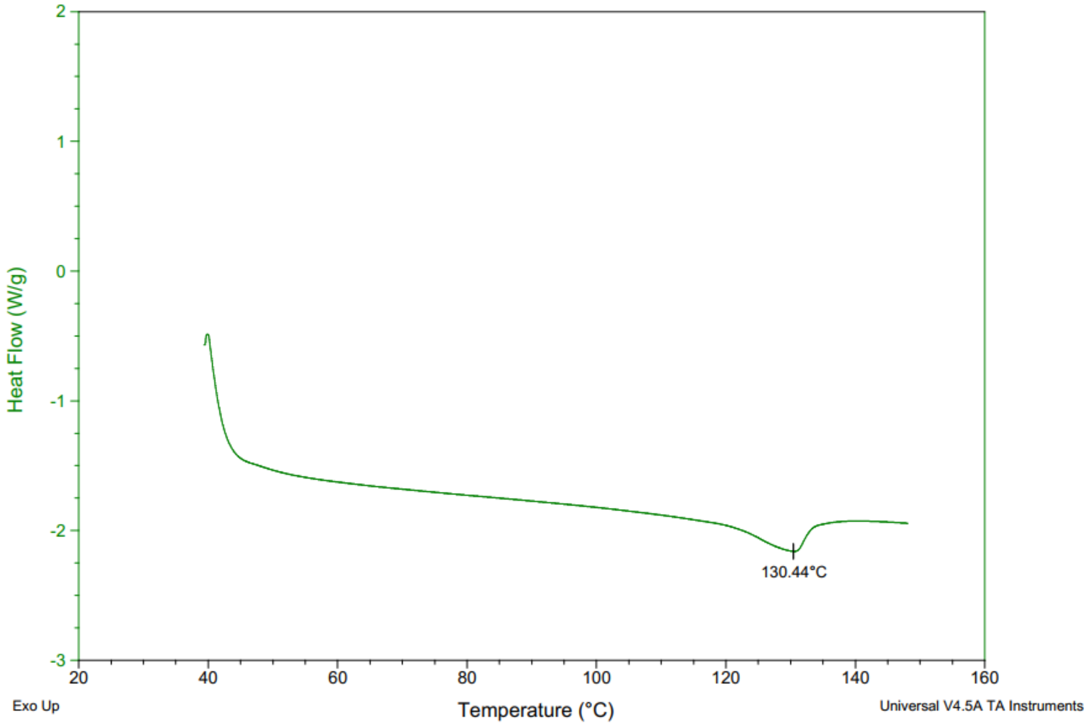

Supplementary Figure 77. DSC of the copolymer from Table 2, Entry 3.

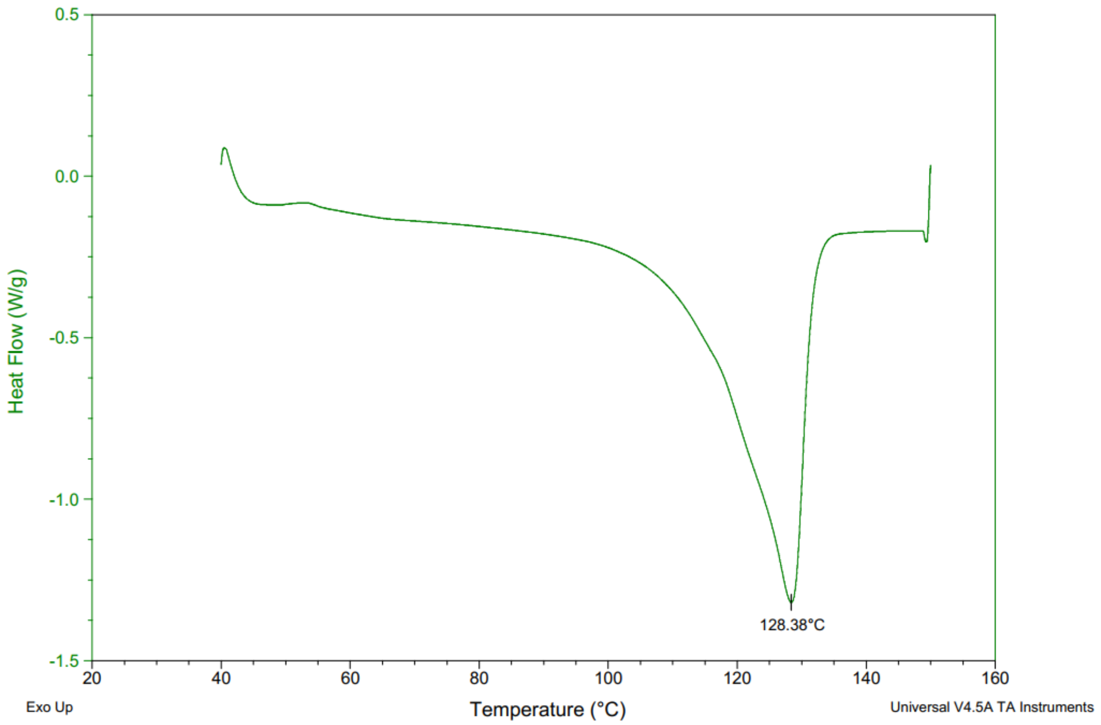

Supplementary Figure 78. DSC of the copolymer from Table 2, Entry 4.

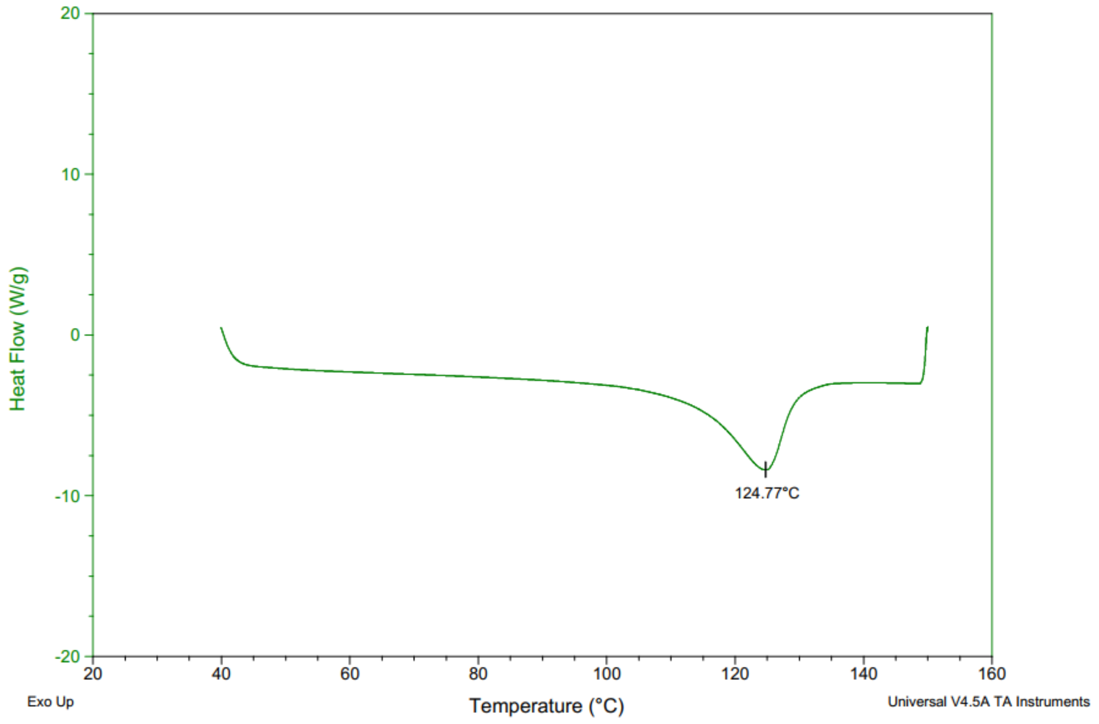

Supplementary Figure 79. DSC of the copolymer from Table 2, Entry 5.

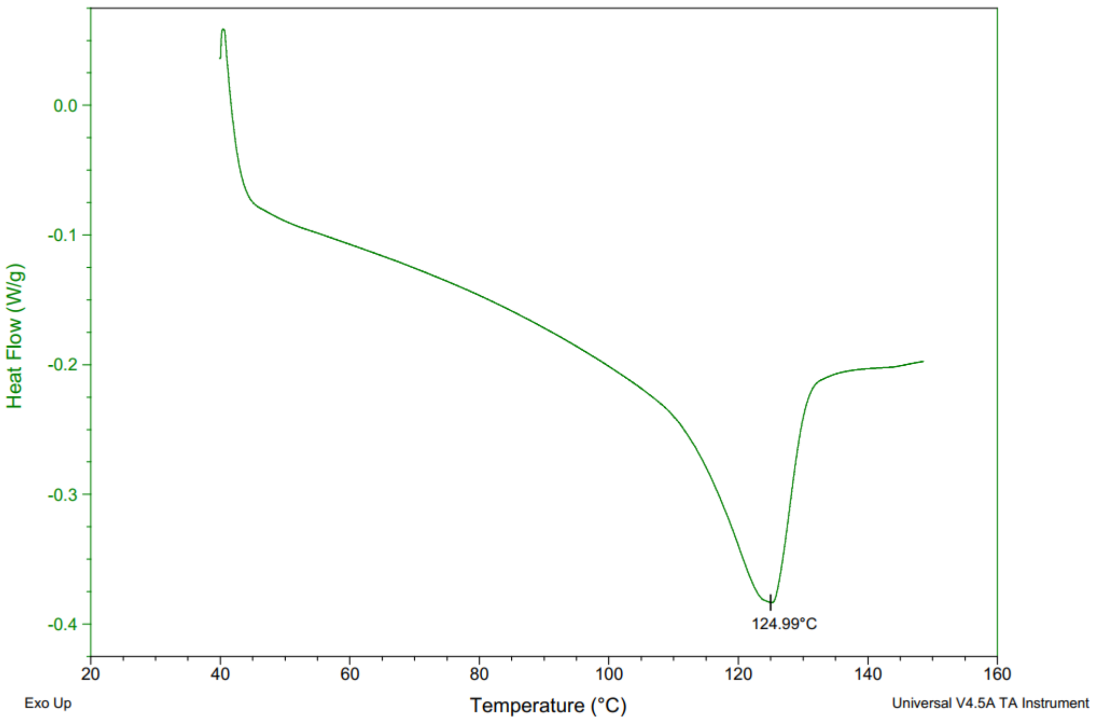

Supplementary Figure 80. DSC of the copolymer from Table 2, Entry 6.

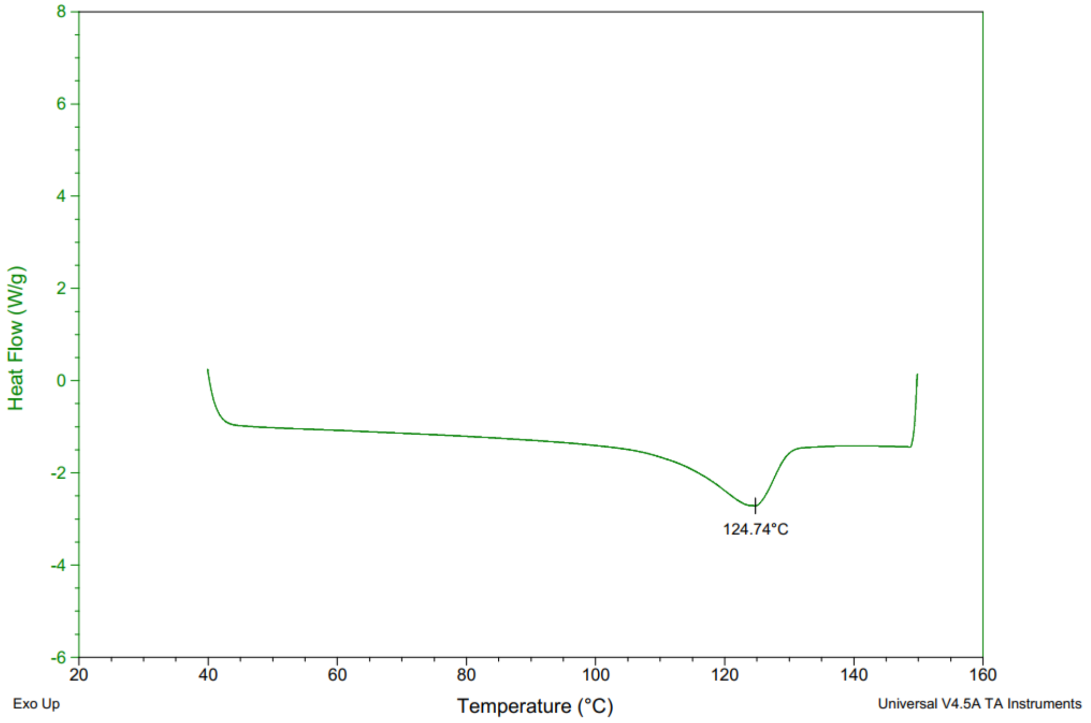

Supplementary Figure 81. DSC of the copolymer from Table 2, Entry 7.

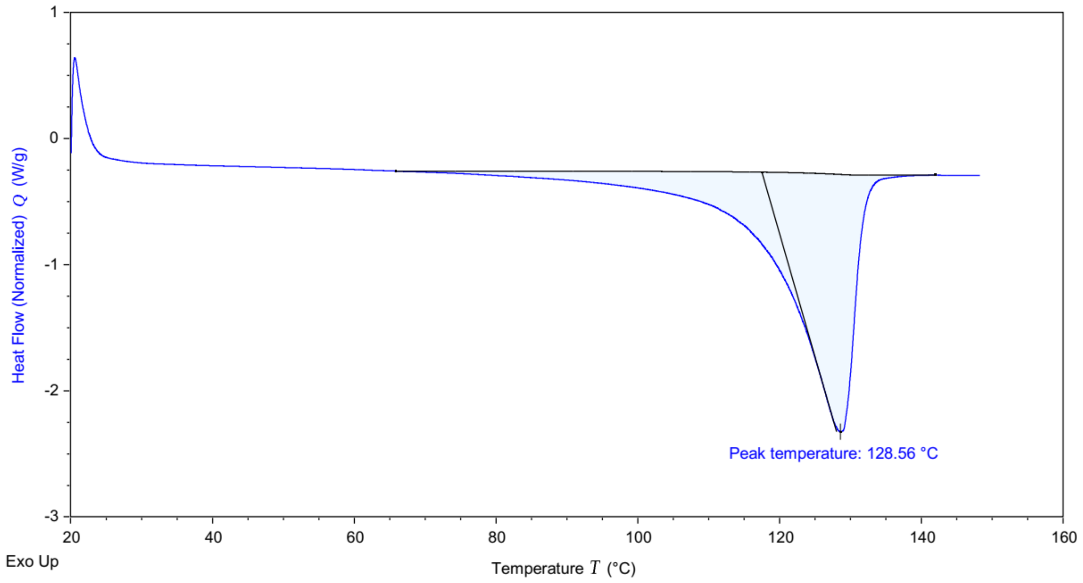

Supplementary Figure 82. DSC of the copolymer from Table 2, Entry 8.

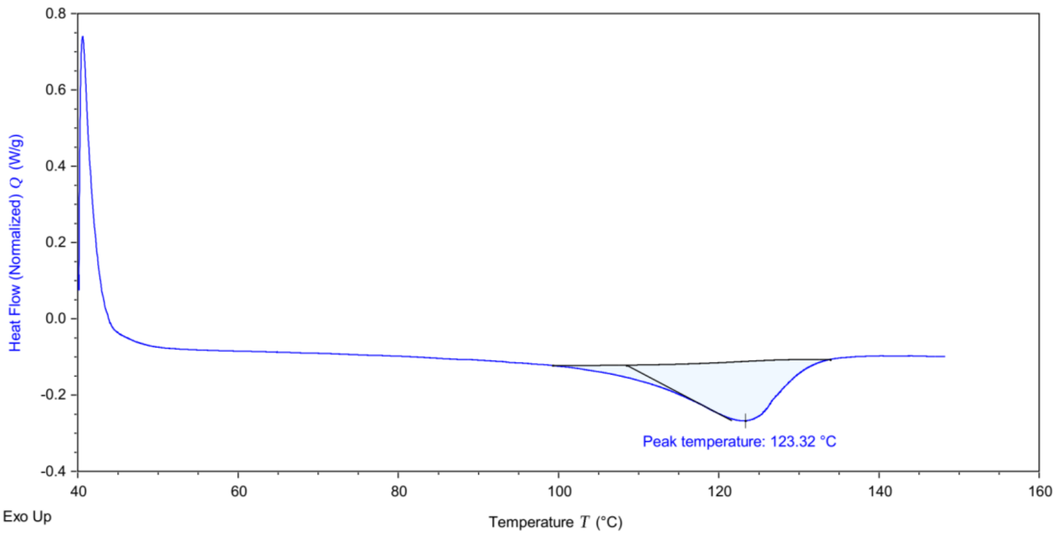

Supplementary Figure 83. DSC of the copolymer from Table 2, Entry 9.

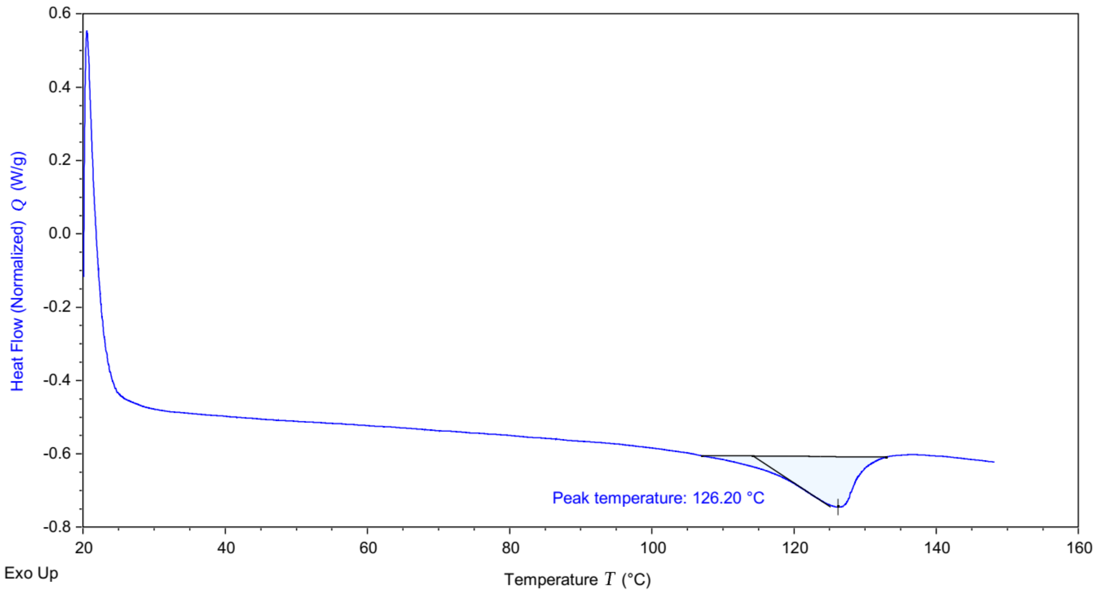

Supplementary Figure 84. DSC of the copolymer from Table 2, Entry 10.

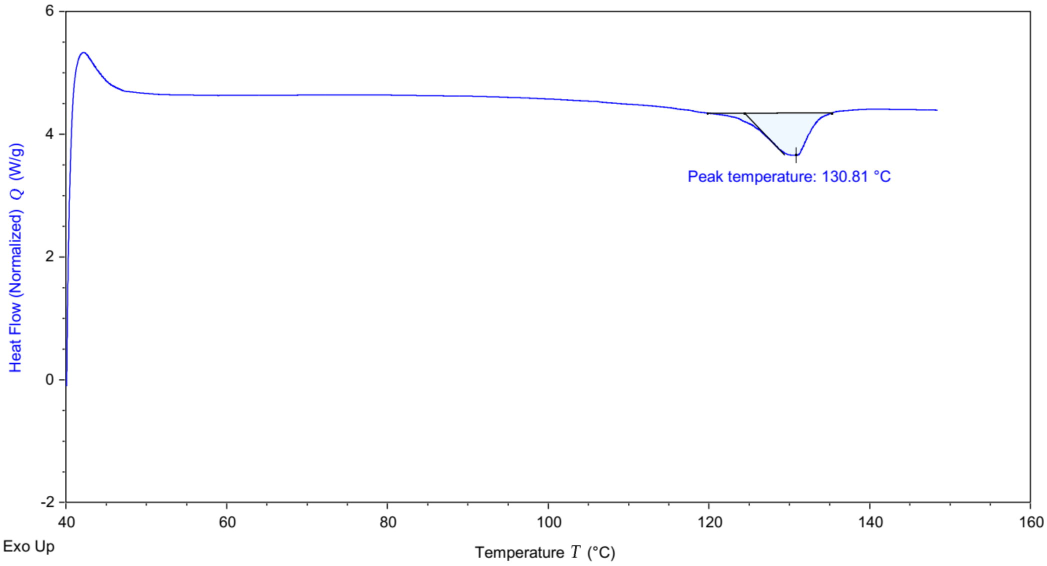

Supplementary Figure 85. DSC of the copolymer from Table 2, Entry 11.

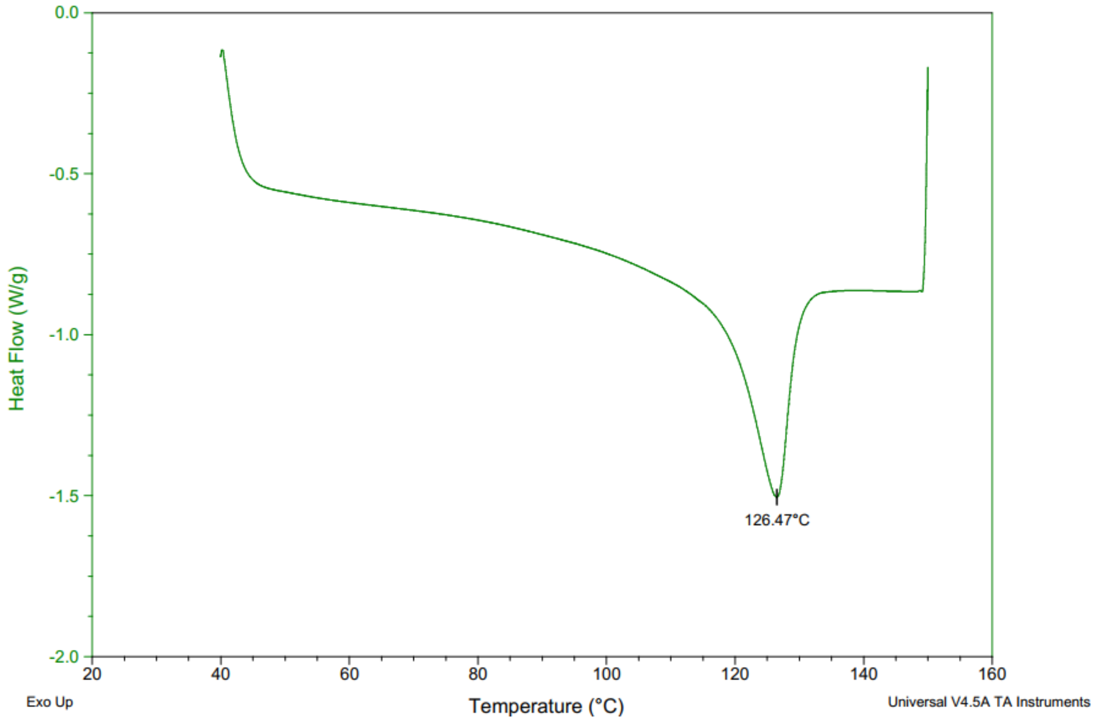

Supplementary Figure 86. DSC of the copolymer from Table 2, Entry 12.

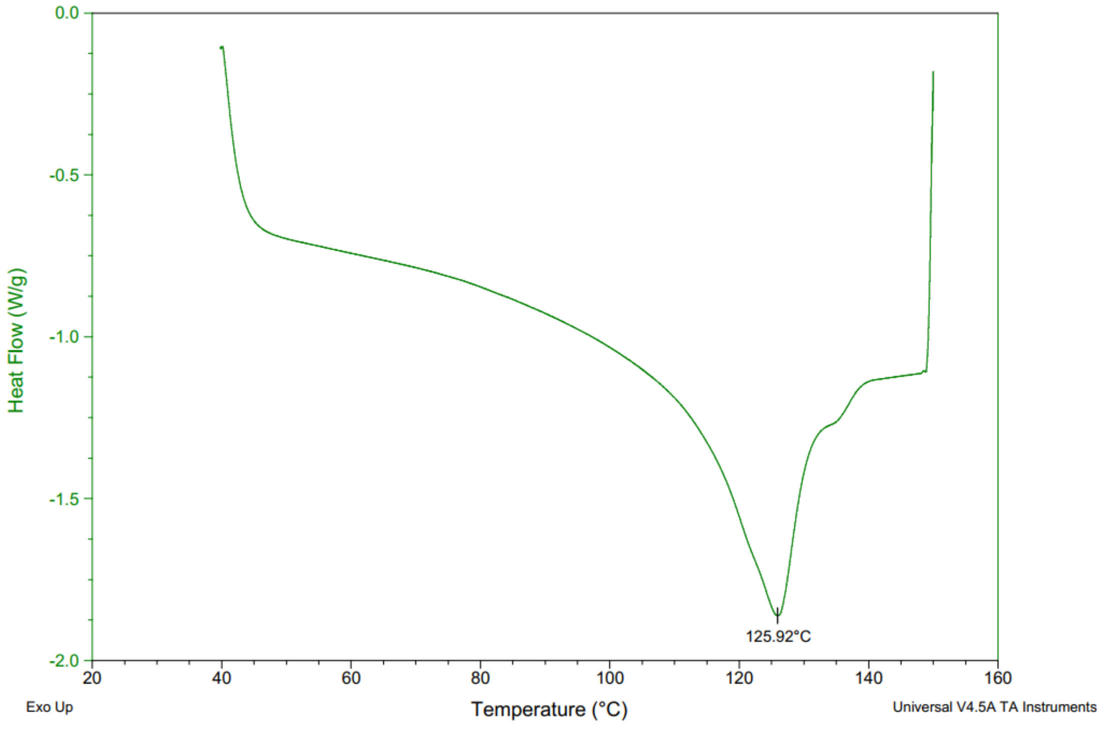

Supplementary Figure 87. DSC of the copolymer from Table 2, Entry 13.

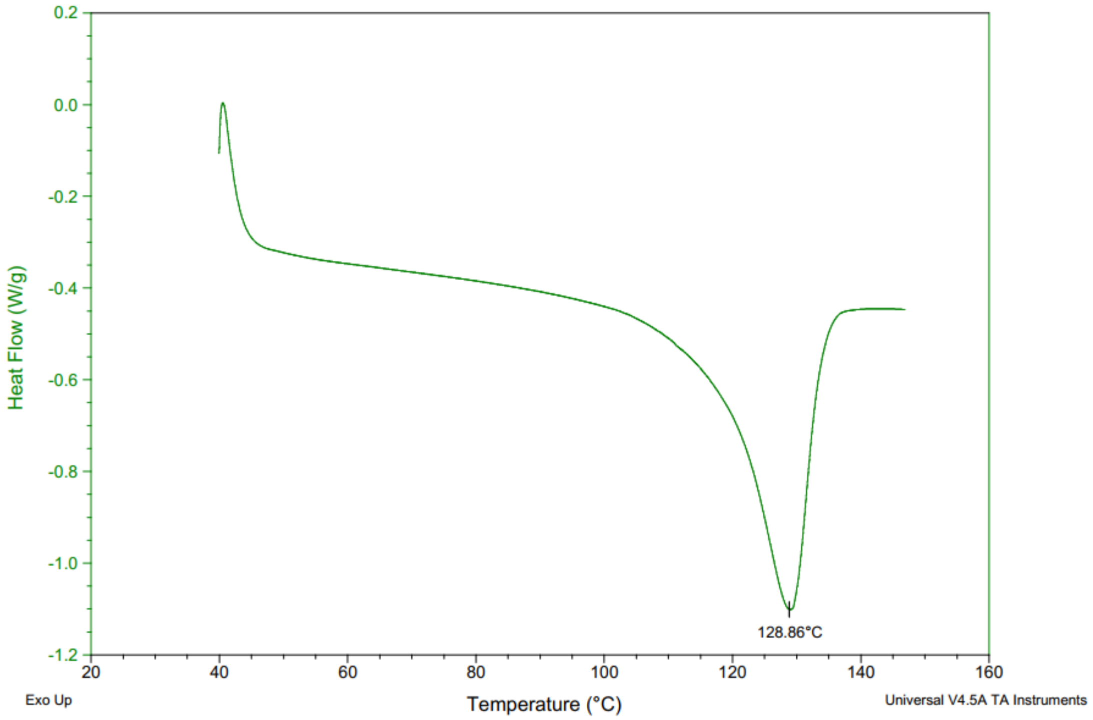

Supplementary Figure 88. DSC of the copolymer from Table 2, Entry 14.

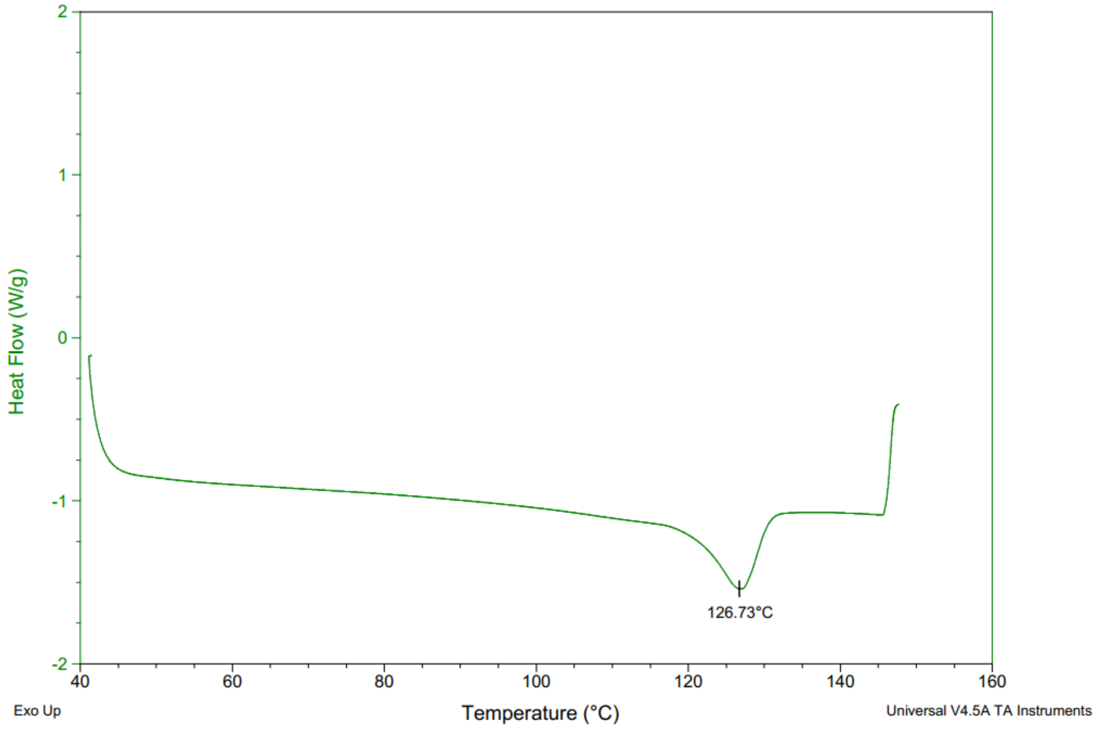

Supplementary Figure 89. DSC of the copolymer from Table 2, Entry 15.

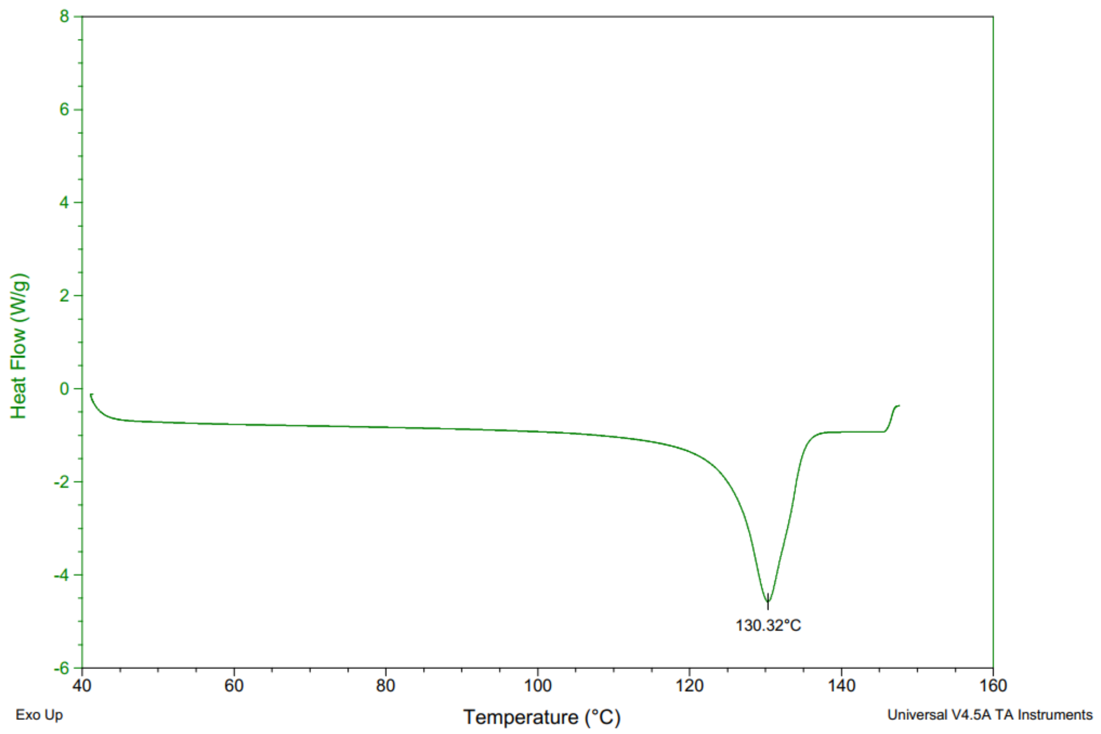

Supplementary Figure 90. DSC of the copolymer from Table 2, Entry 16.

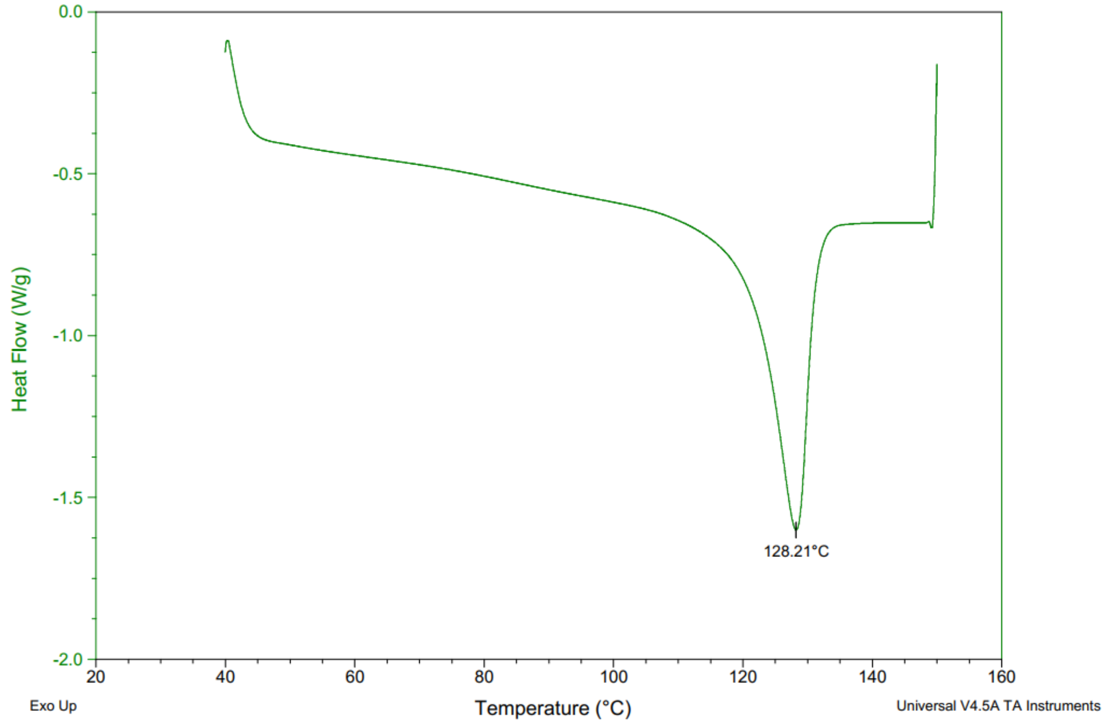

Supplementary Figure 91. DSC of the copolymer from Table 2, Entry 17.

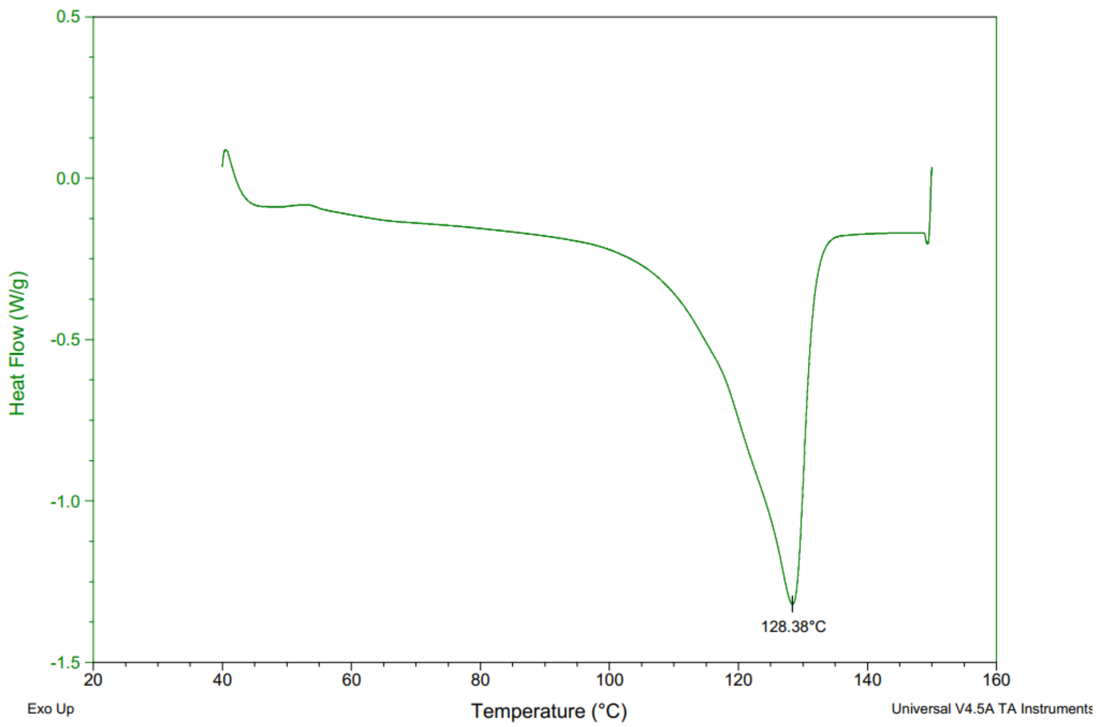

Supplementary Figure 92. DSC of the copolymer from Table 2, Entry 18.

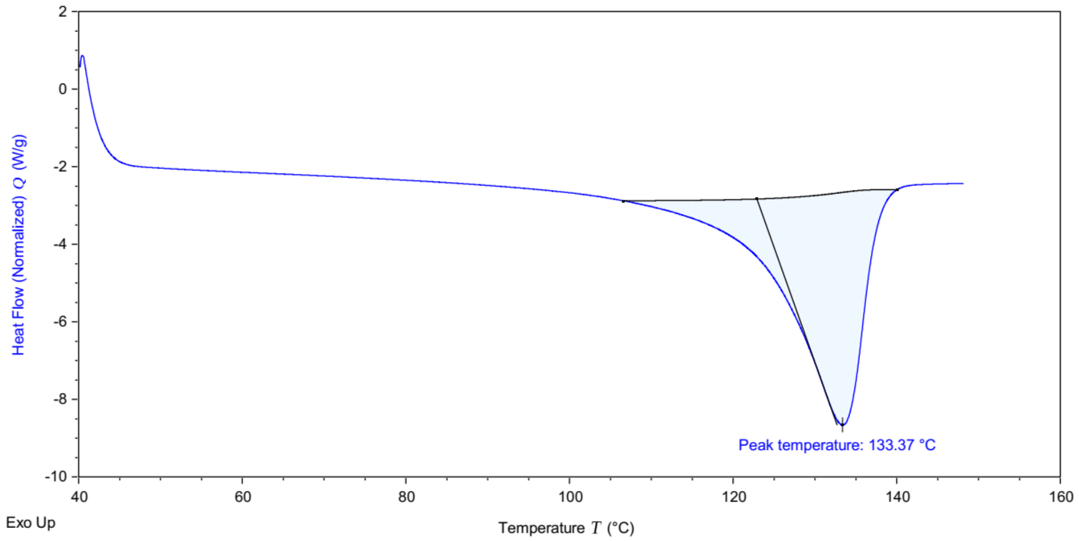

Supplementary Figure 93. DSC of the copolymer from Table 2, Entry 19.

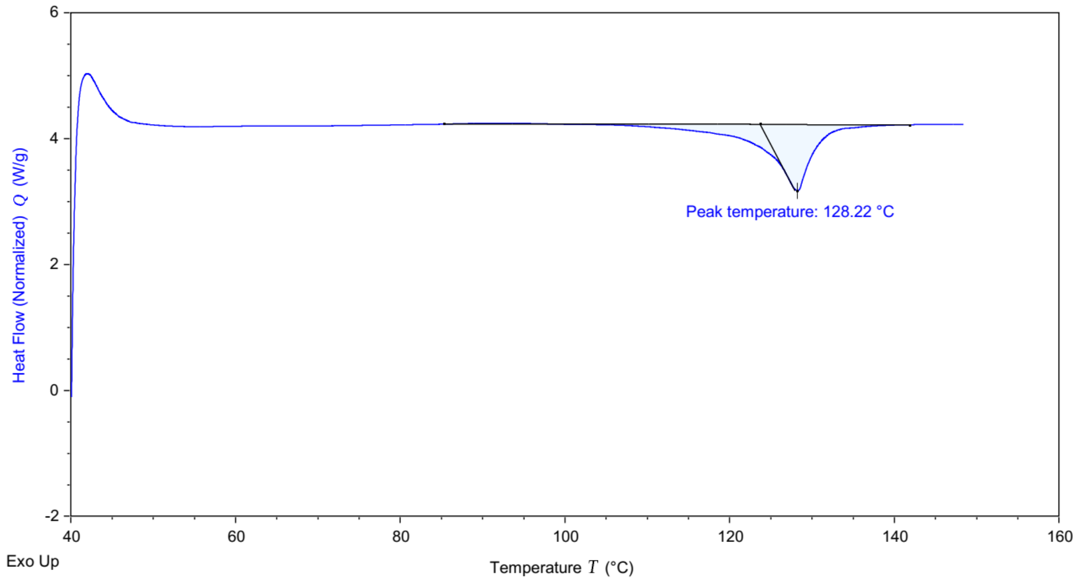

Supplementary Figure 94. DSC of the copolymer from Table 2, Entry 20.

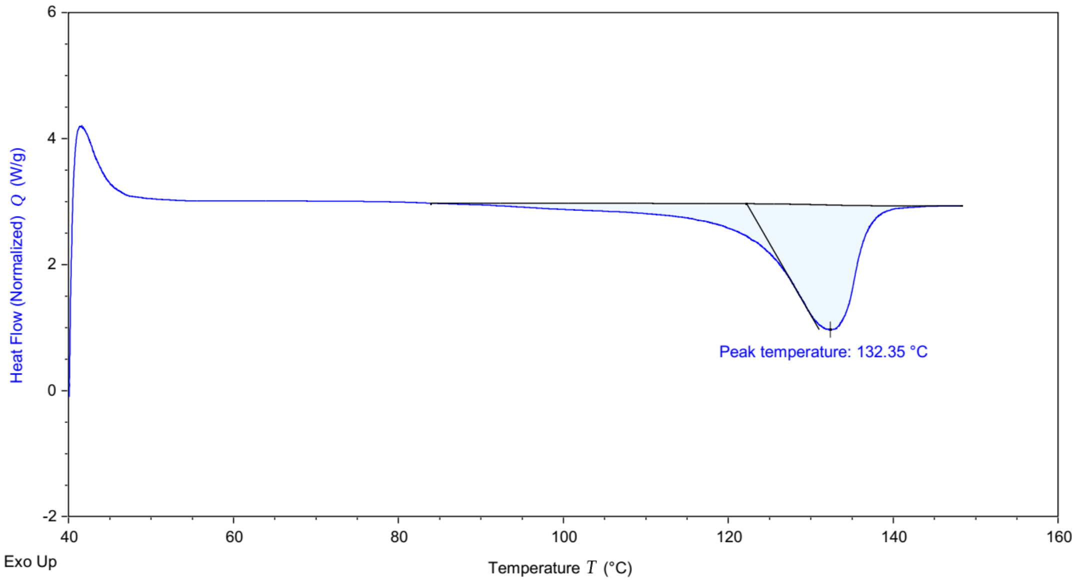

Supplementary Figure 95. DSC of the copolymer from Table 2, Entry 21.

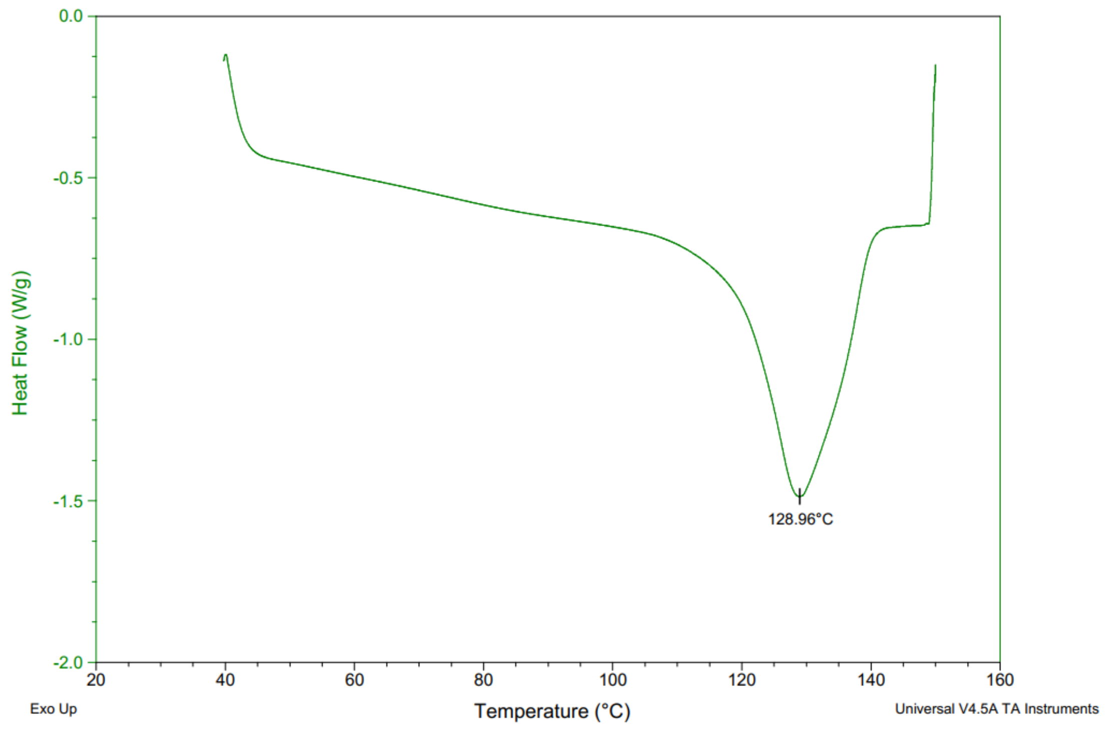

Supplementary Figure 96. DSC of the copolymer from Table 2, Entry 22.

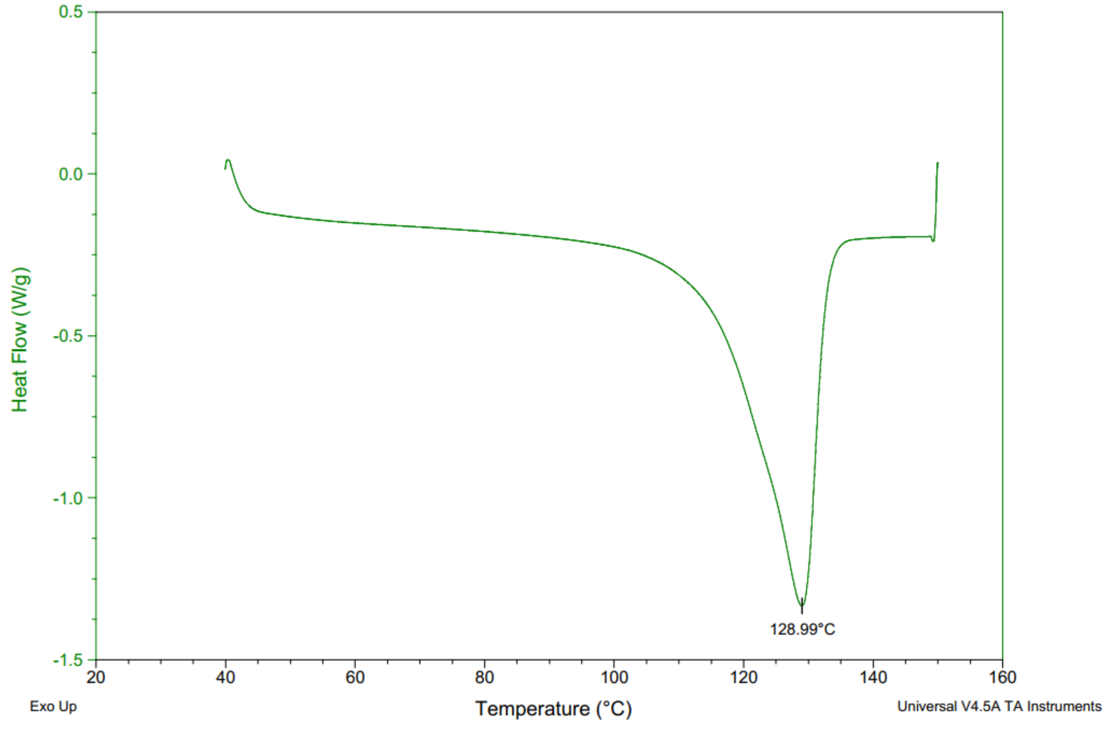

Supplementary Figure 97. DSC of the copolymer from Table 2, Entry 23.

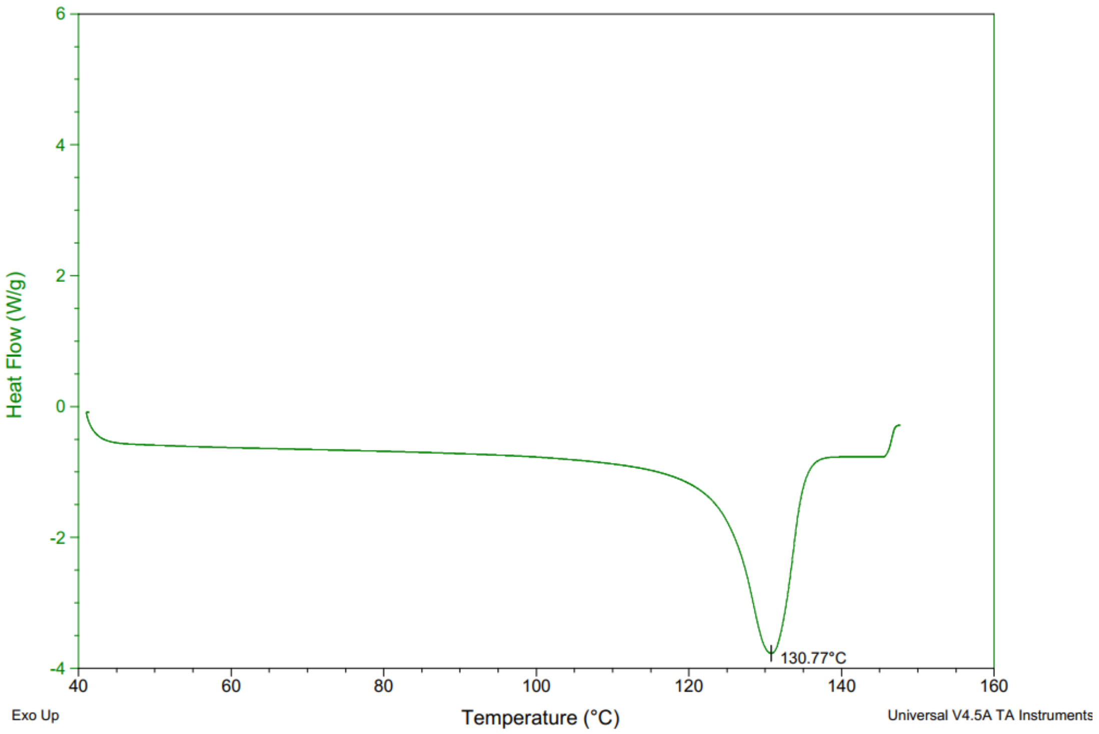

Supplementary Figure 98. DSC of the copolymer from Table 2, Entry 24.

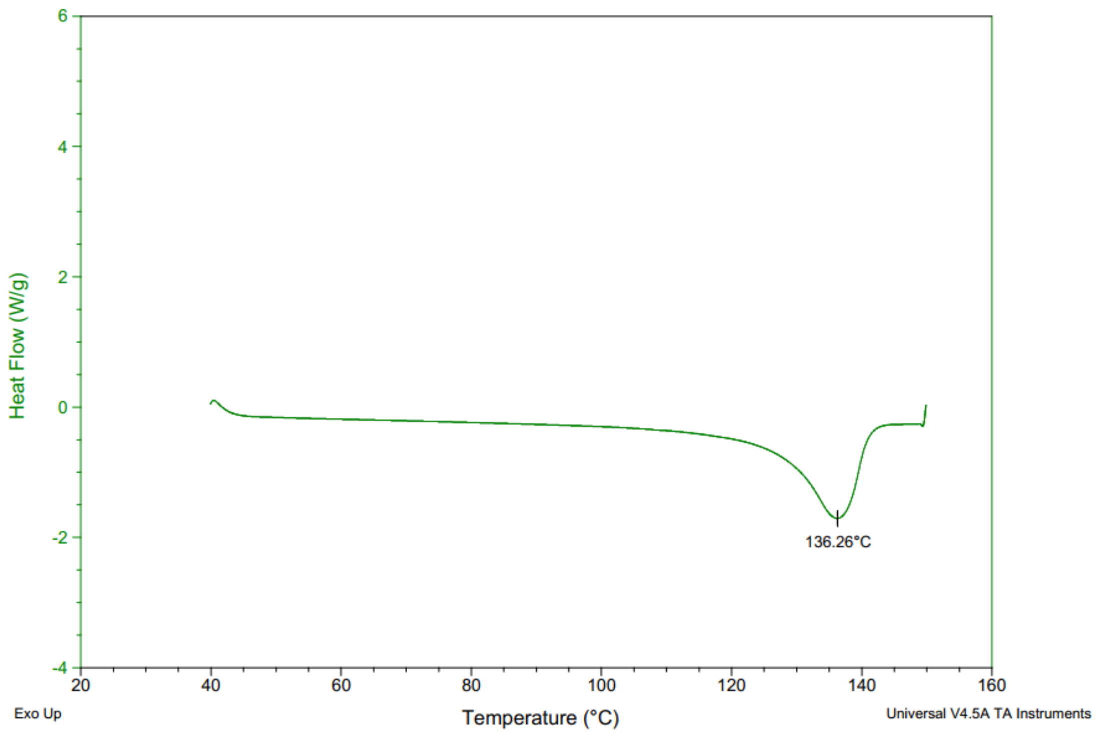

Supplementary Figure 99. DSC of the copolymer from Table 3, Entry1.

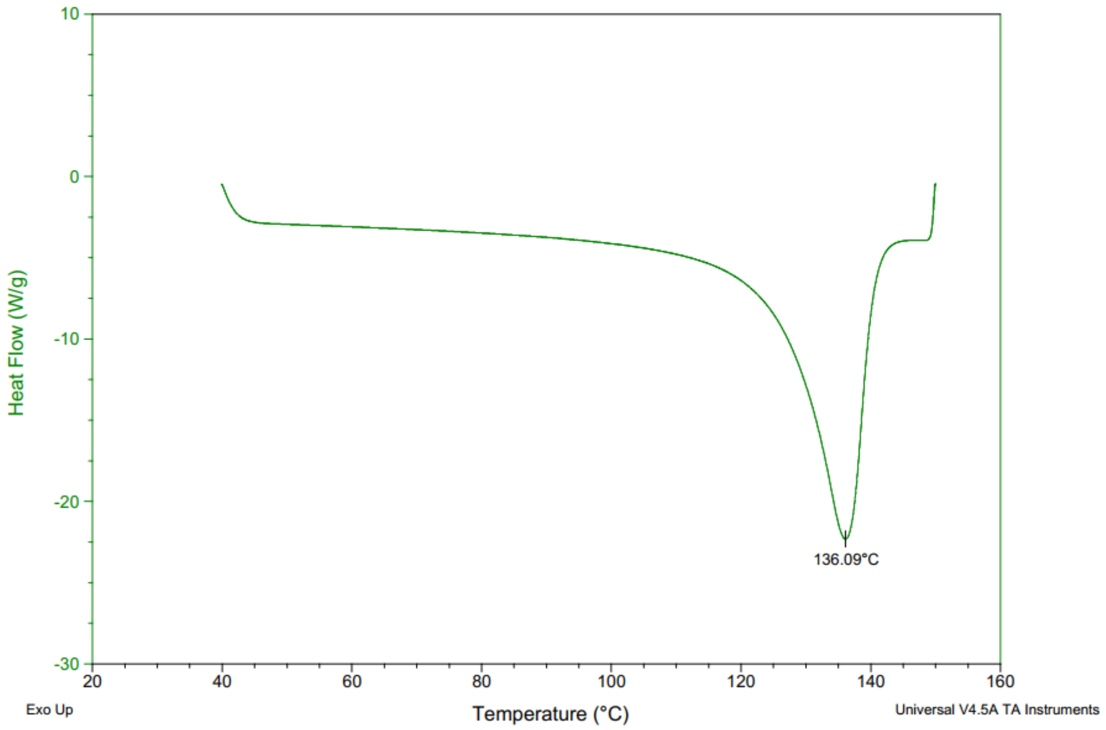

Supplementary Figure 100. DSC of the copolymer from Table 3, Entry 2.

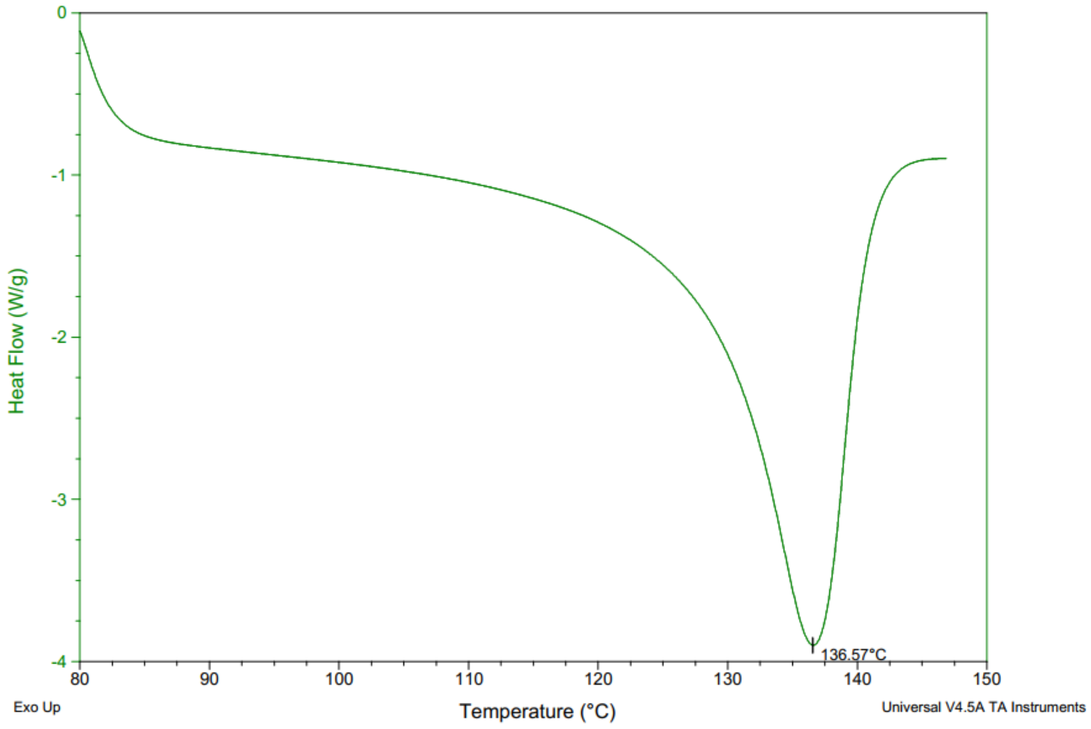

Supplementary Figure 101. DSC of the copolymer from Table 3, Entry 3.

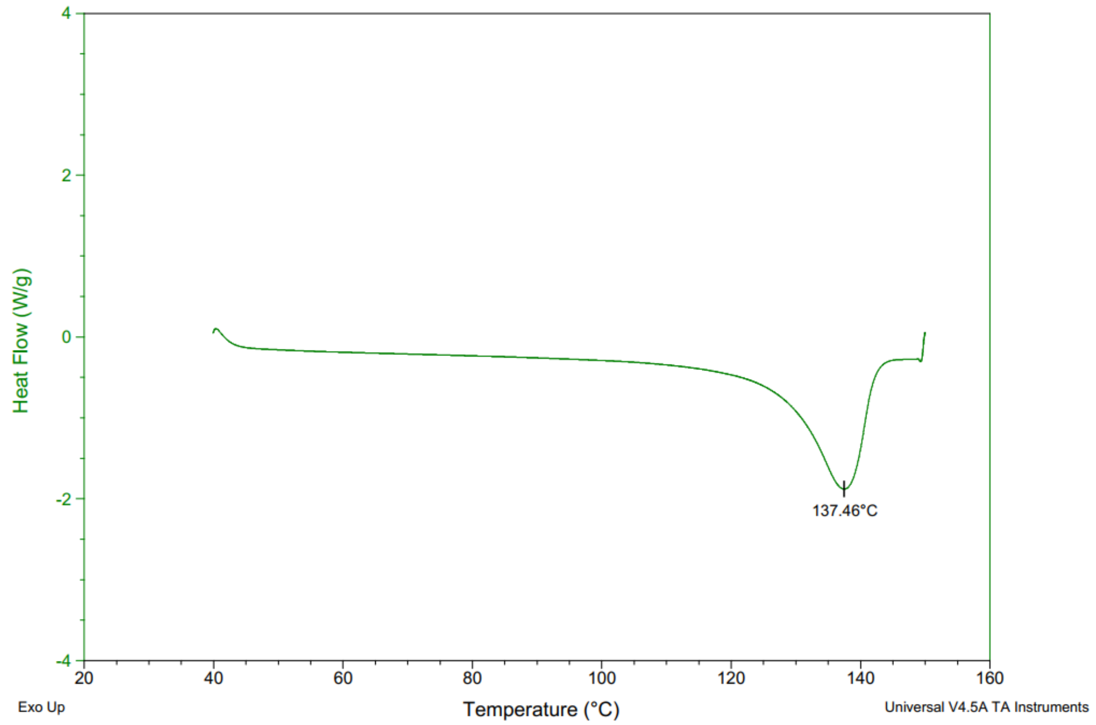

Supplementary Figure 102. DSC of the copolymer from Table 3, Entry 4.

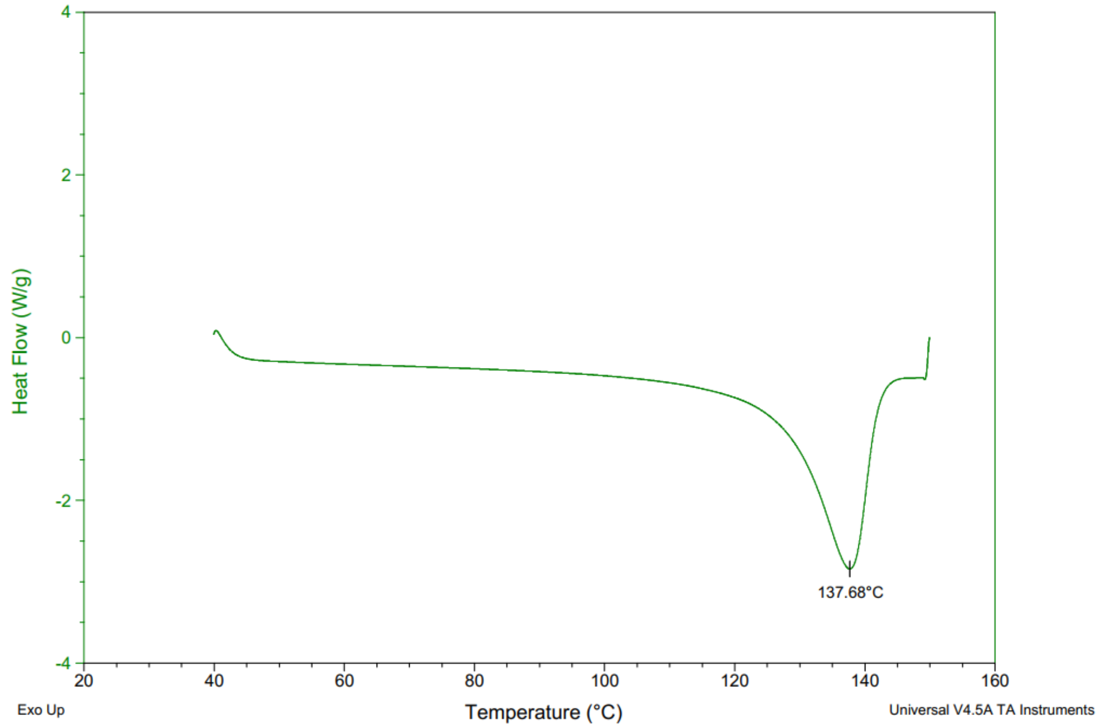

Supplementary Figure 103. DSC of the copolymer from Table 3, Entry 5.

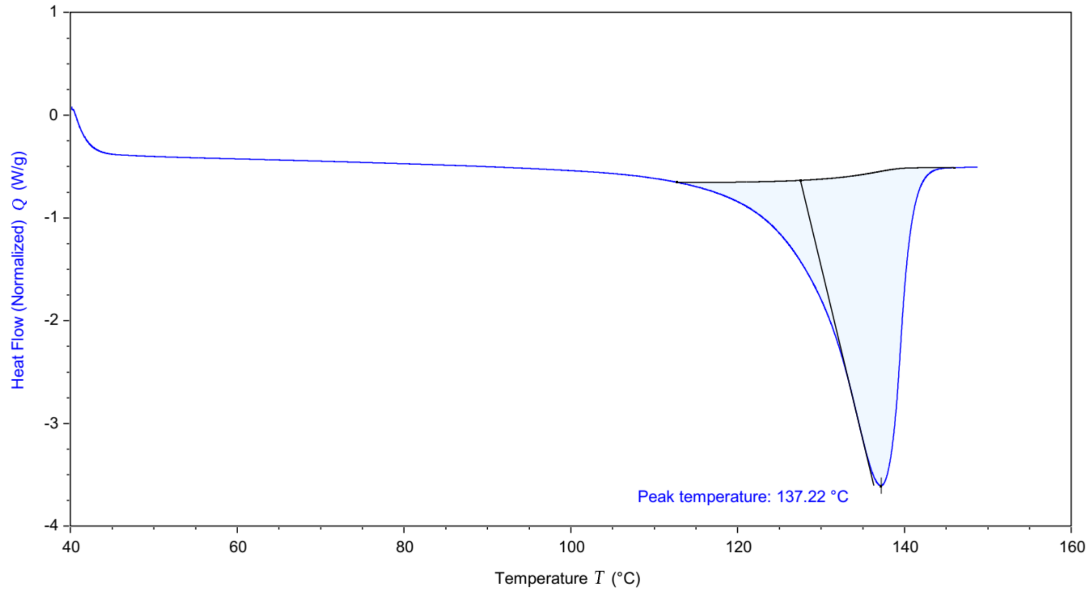

Supplementary Figure 104. DSC of the copolymer from Table 3, Entry 6.

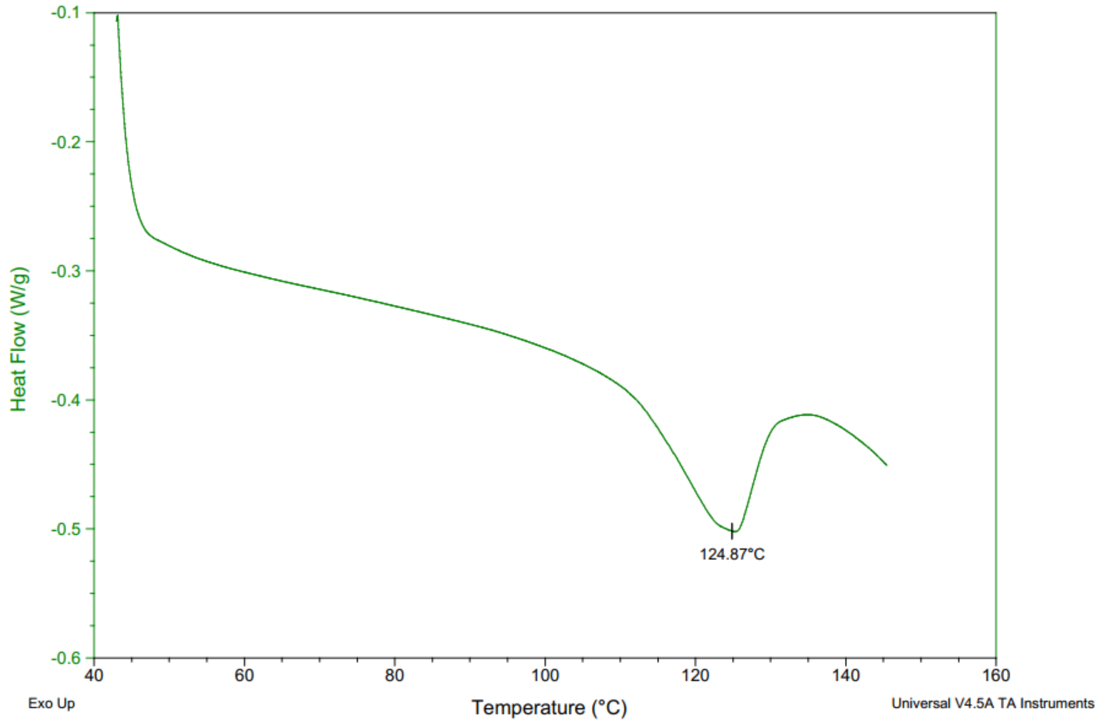

Supplementary Figure 105. DSC of the copolymer from Table 3, Entry 7.

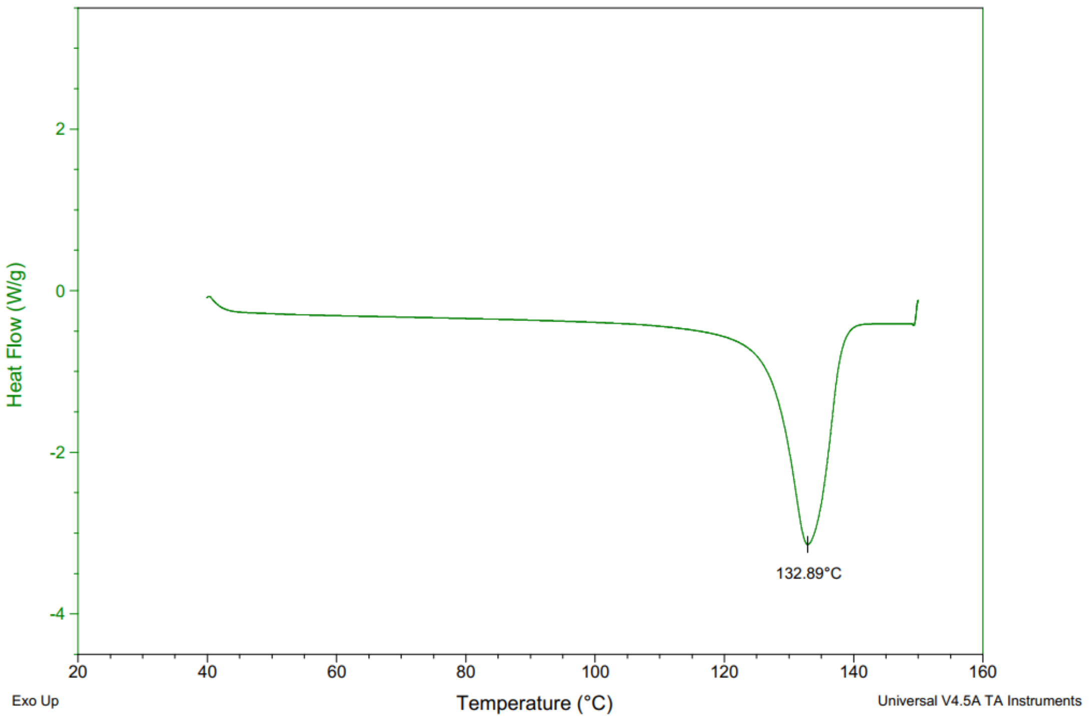

Supplementary Figure 106. DSC of the copolymer from Table 3, Entry 8.

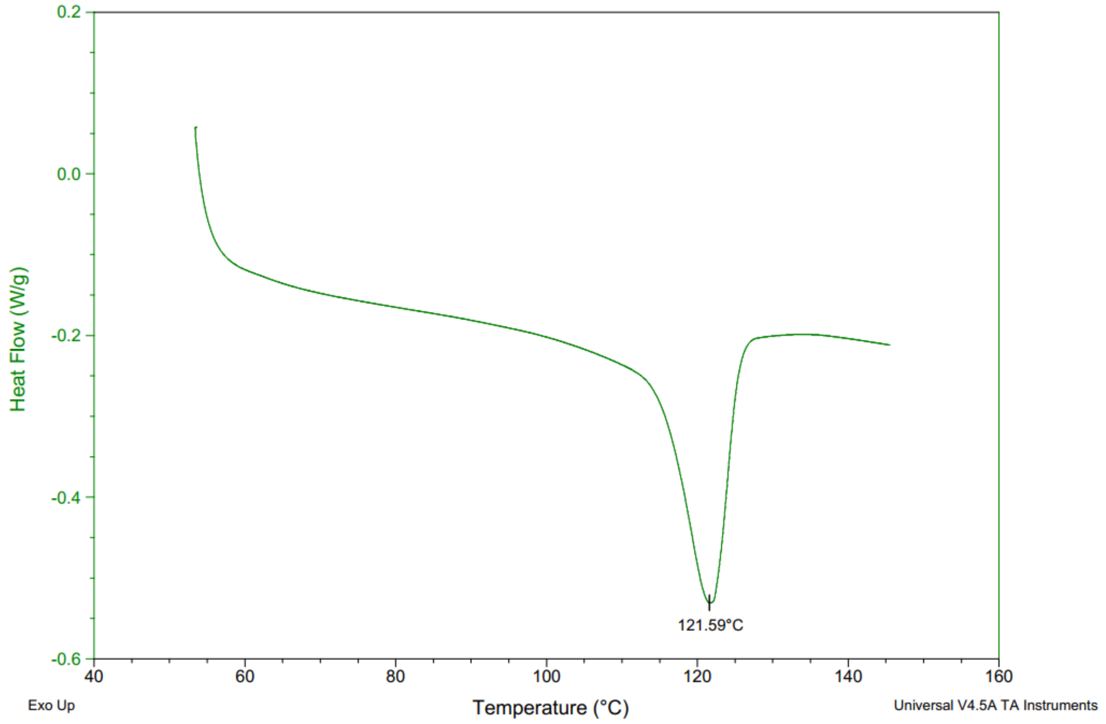

Supplementary Figure 107. DSC of the copolymer from Table 3, Entry 9.

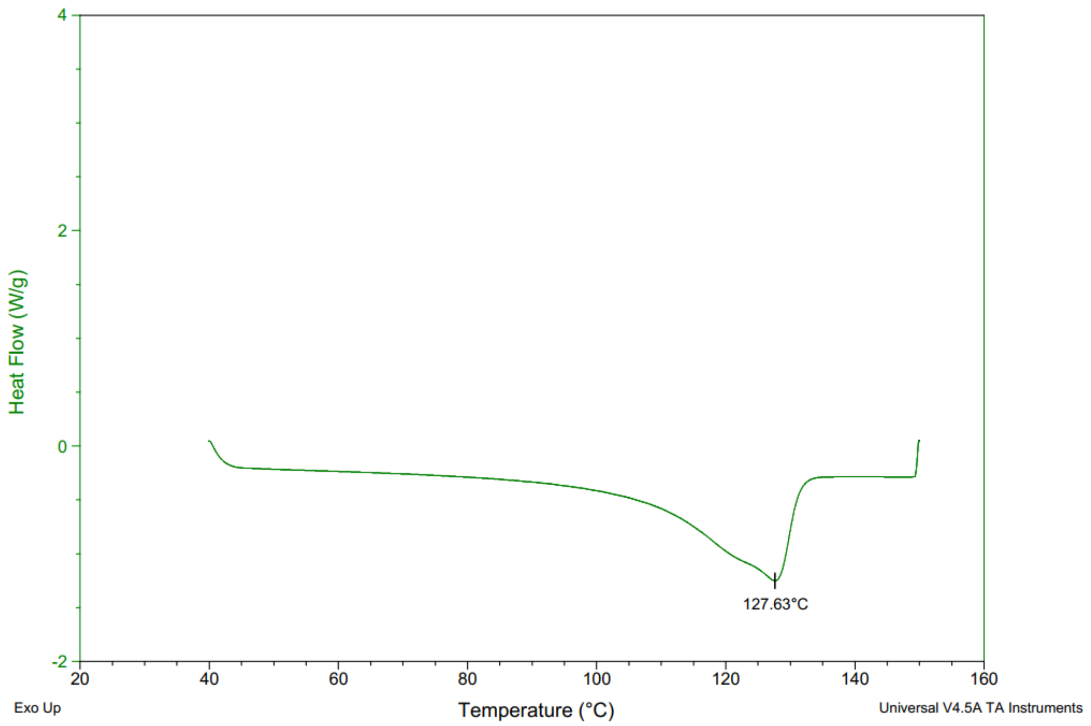

Supplementary Figure 108. DSC of the copolymer from Table 3, Entry 10.

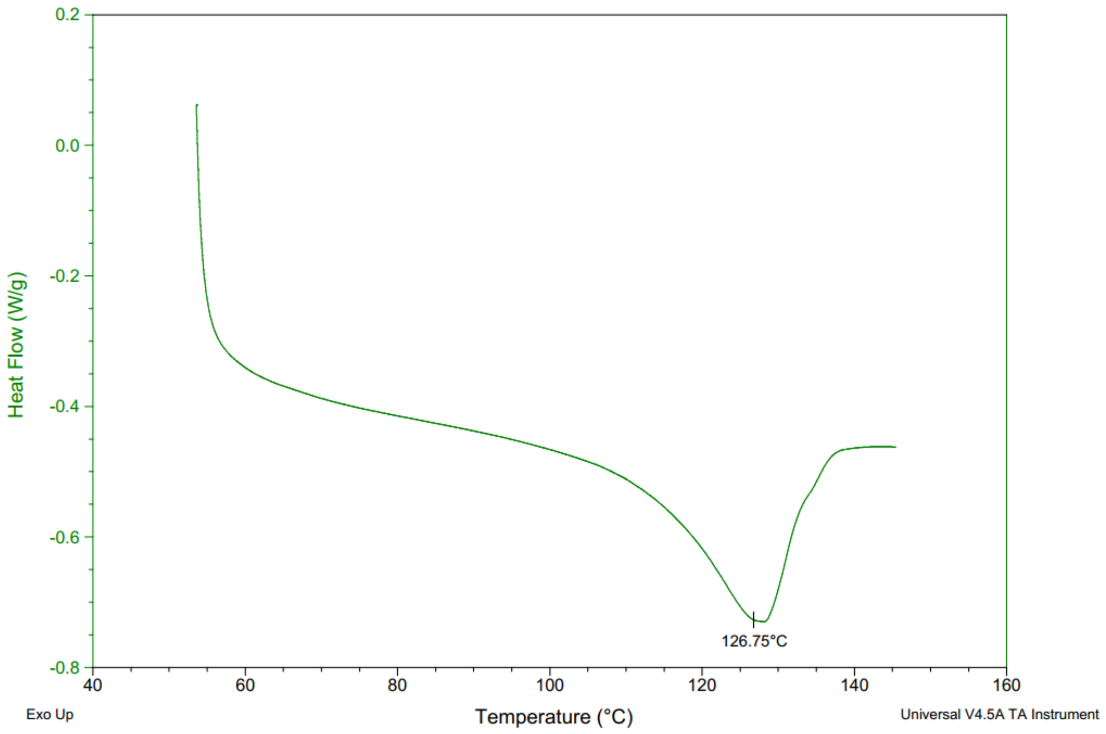

Supplementary Figure 109. DSC of the copolymer from Table 3, Entry 11.

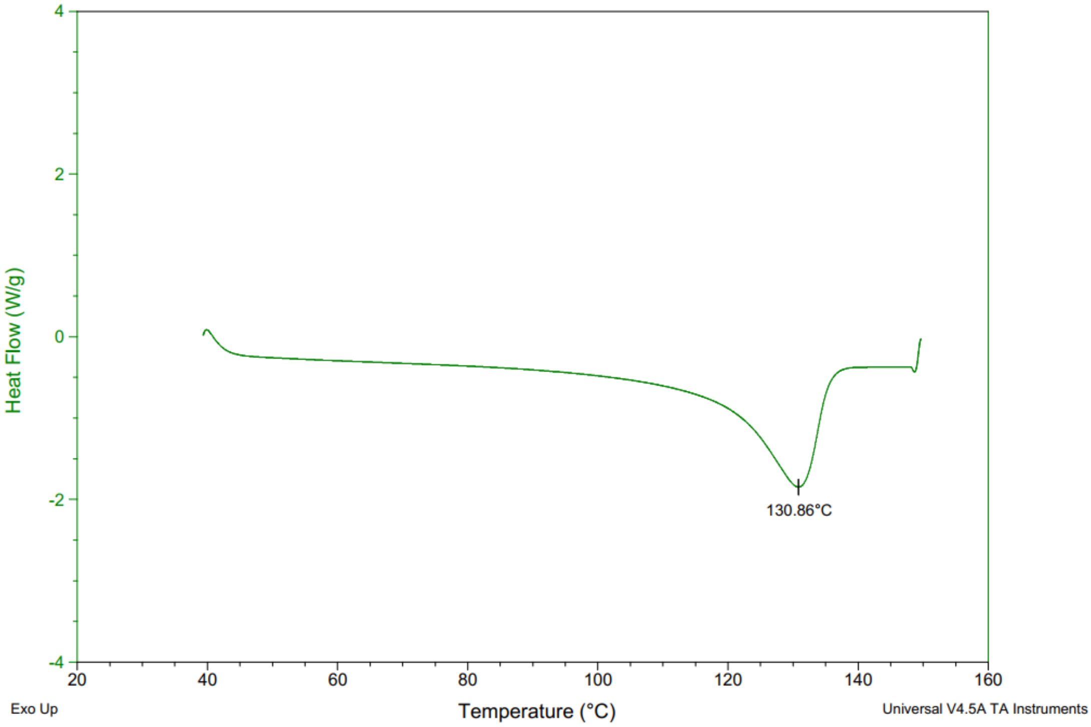

Supplementary Figure 110. DSC of the copolymer from Table 3, Entry12

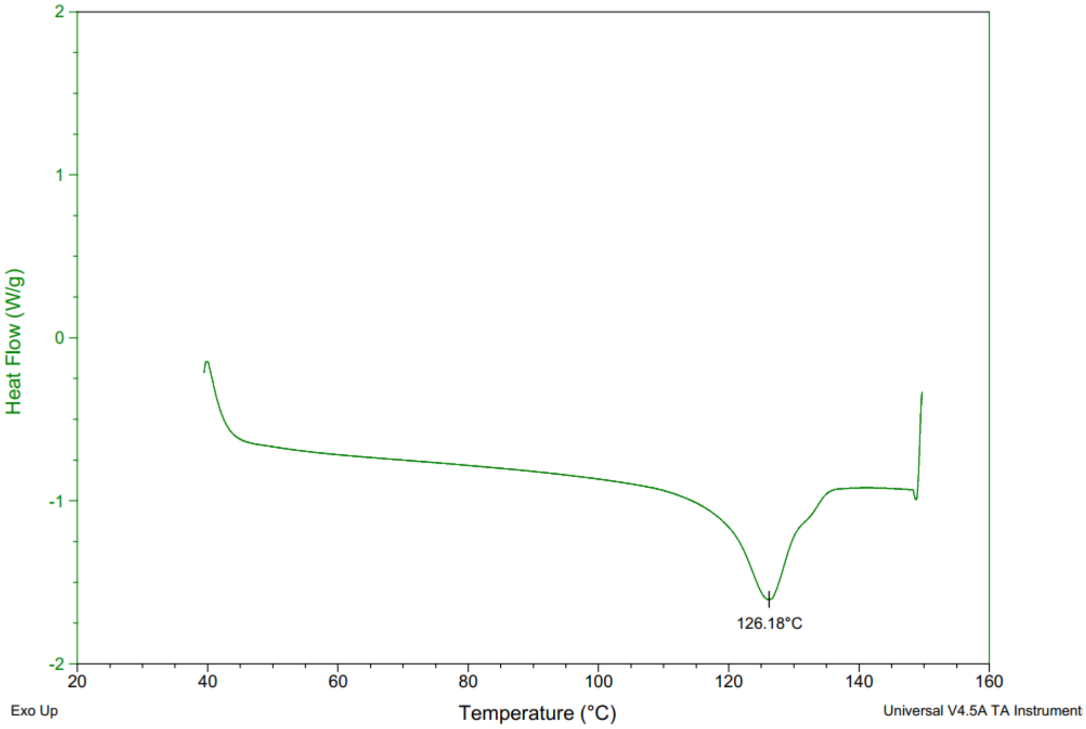

Supplementary Figure 111. DSC of the copolymer from Table 3, Entry 13.

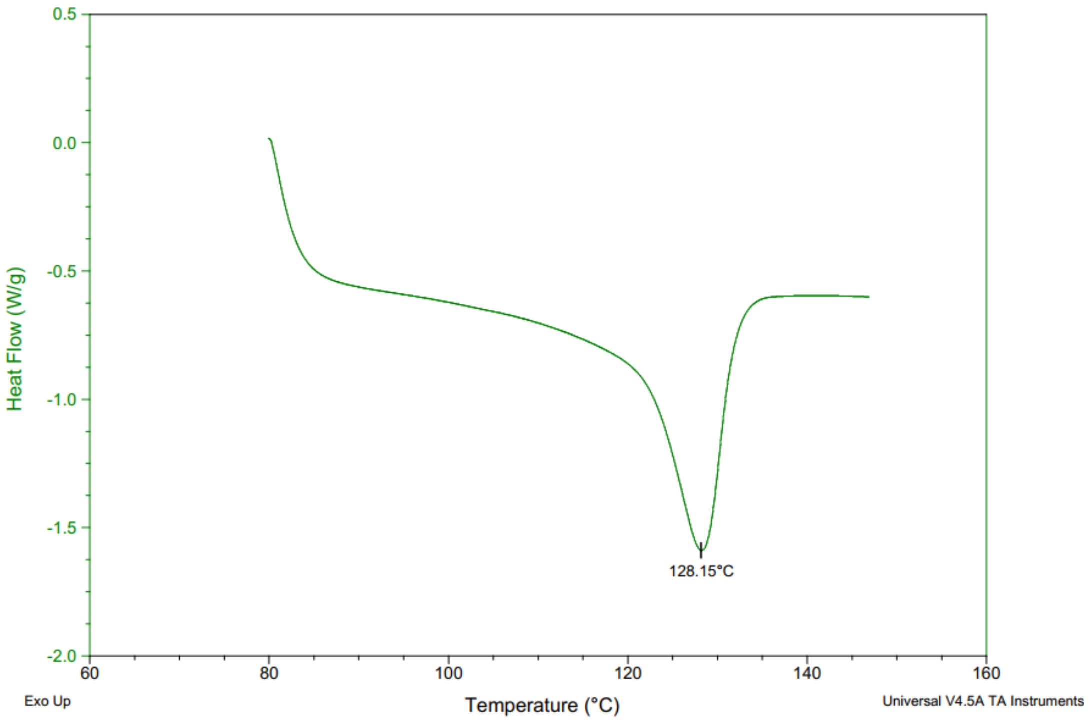

Supplementary Figure 112. DSC of the copolymer from Table 3, Entry 14.

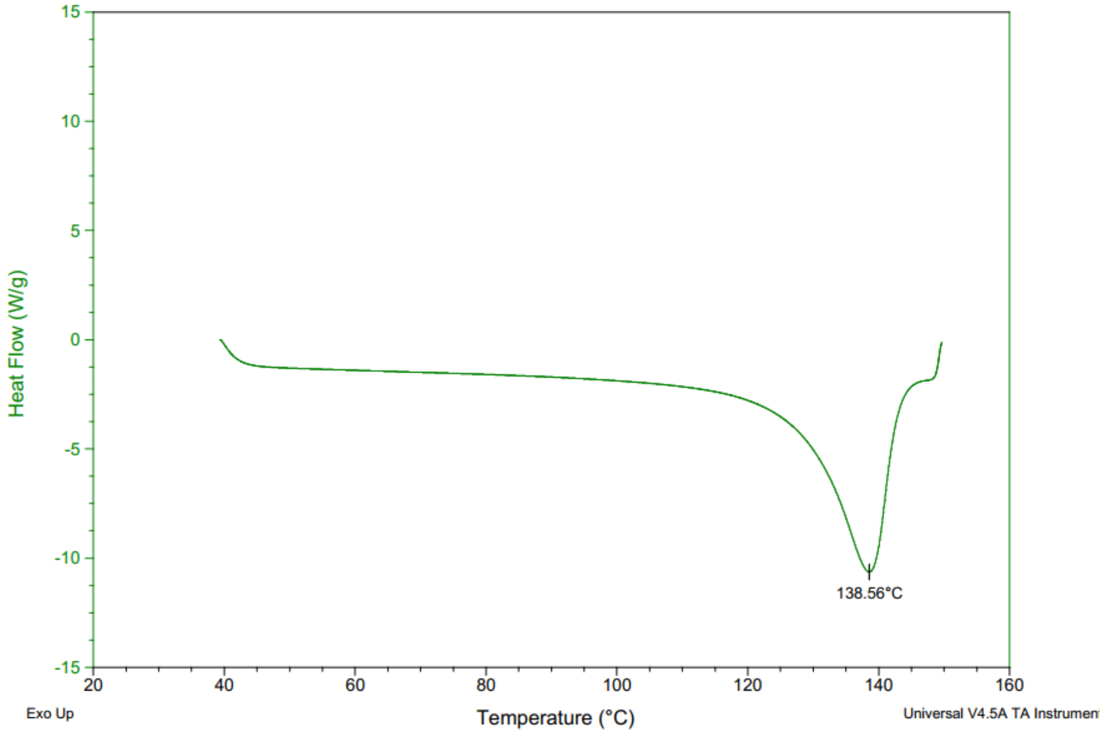

Supplementary Figure 113. DSC of the copolymer from Table 4, Entry 1.

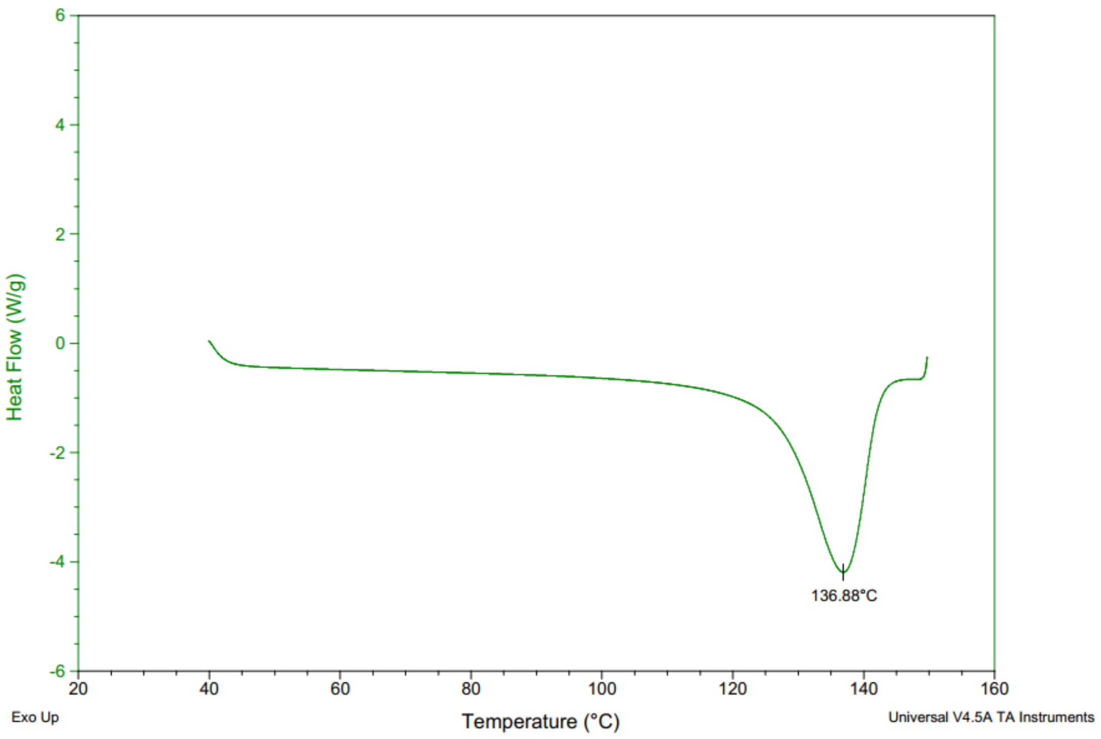

Supplementary Figure 114. DSC of the copolymer from Table 4, Entry 2.

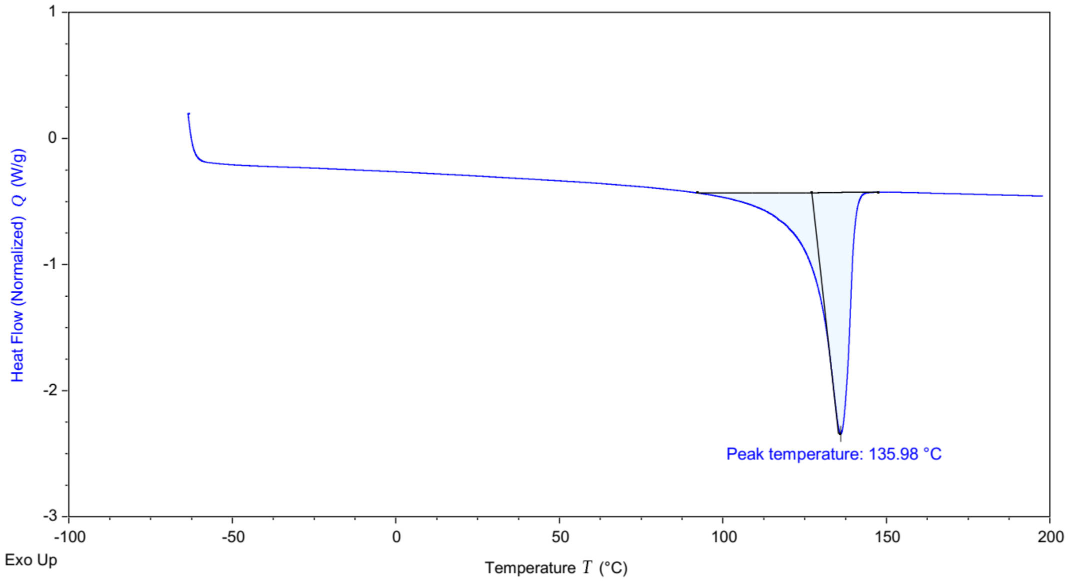

Supplementary Figure 115. DSC of the copolymer from Table 4, Entry 3.

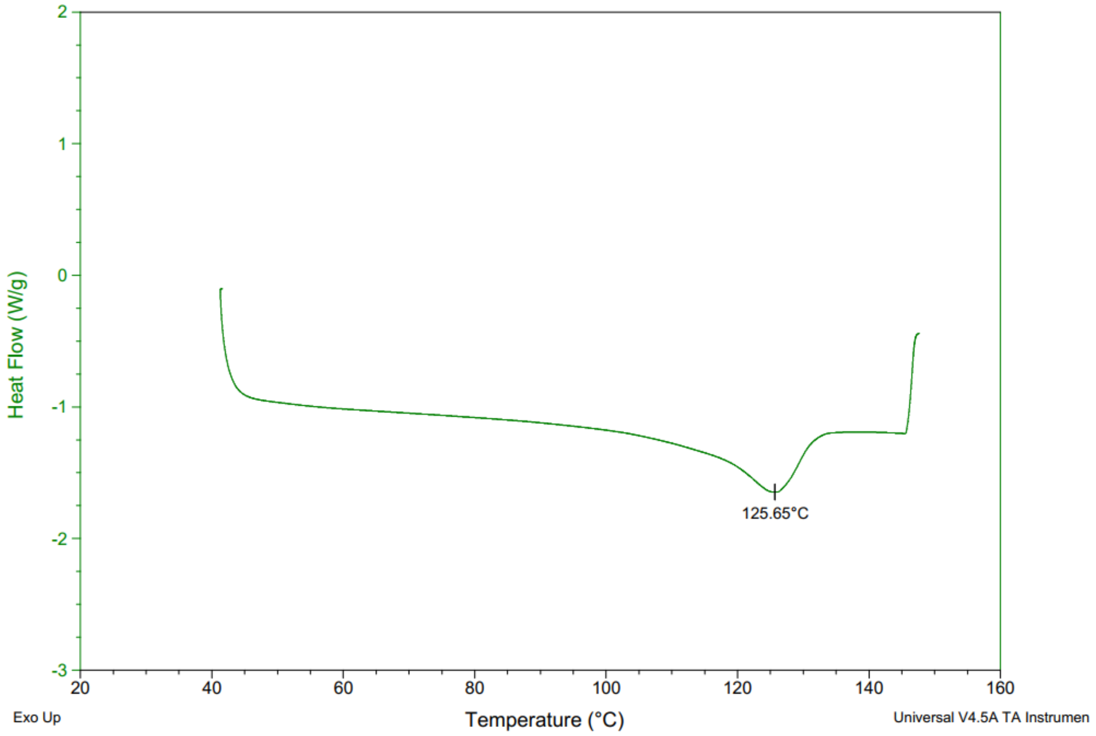

Supplementary Figure 116. DSC of the copolymer from Table 4, Entry 4.

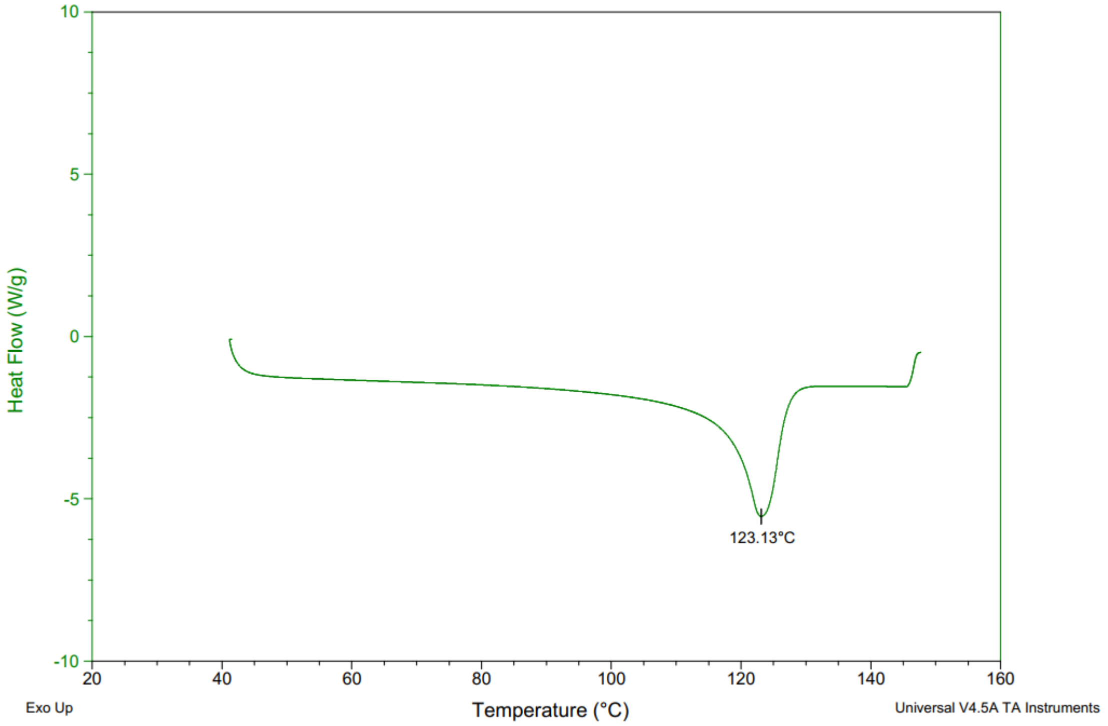

Supplementary Figure 117. DSC of the copolymer from Table 4, Entry 5.

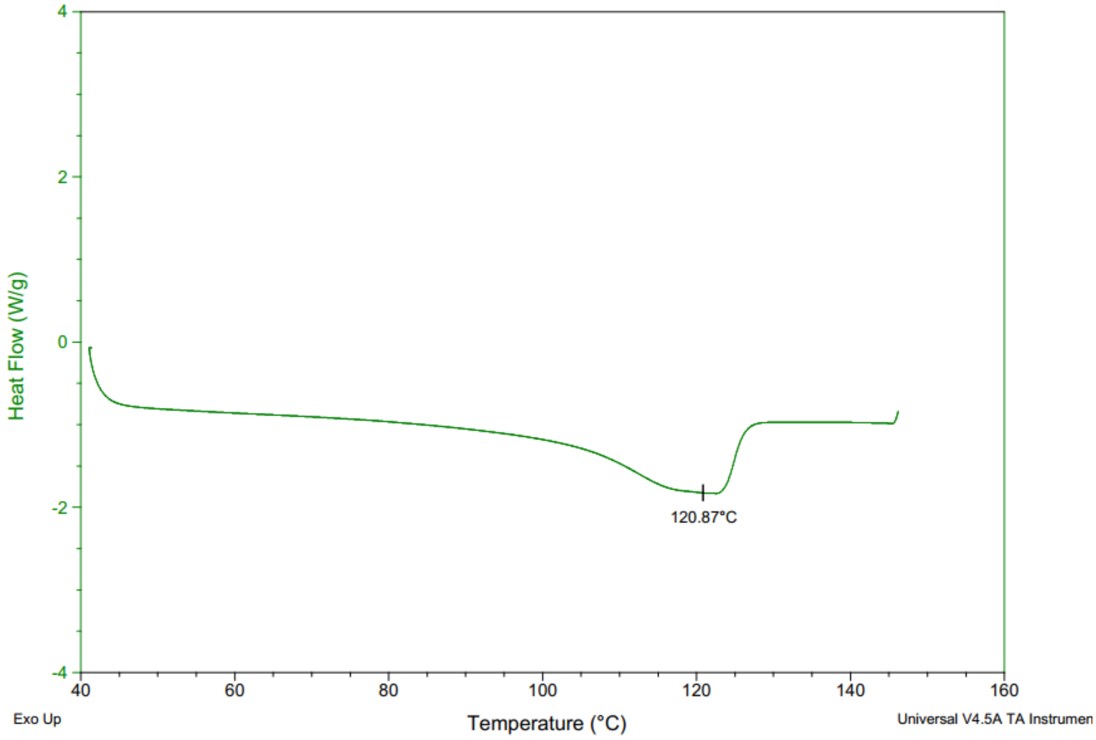

Supplementary Figure 118. DSC of the copolymer from Table 4, Entry 6.

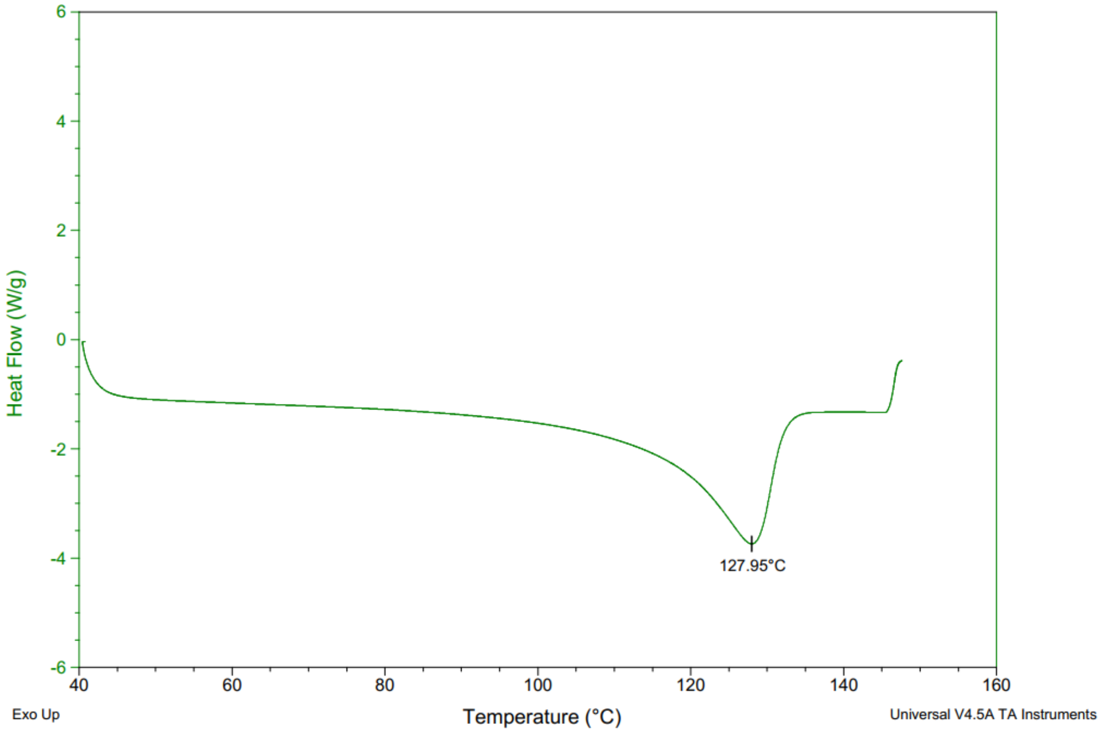

Supplementary Figure 119. DSC of the copolymer from Table 4, Entry 7.

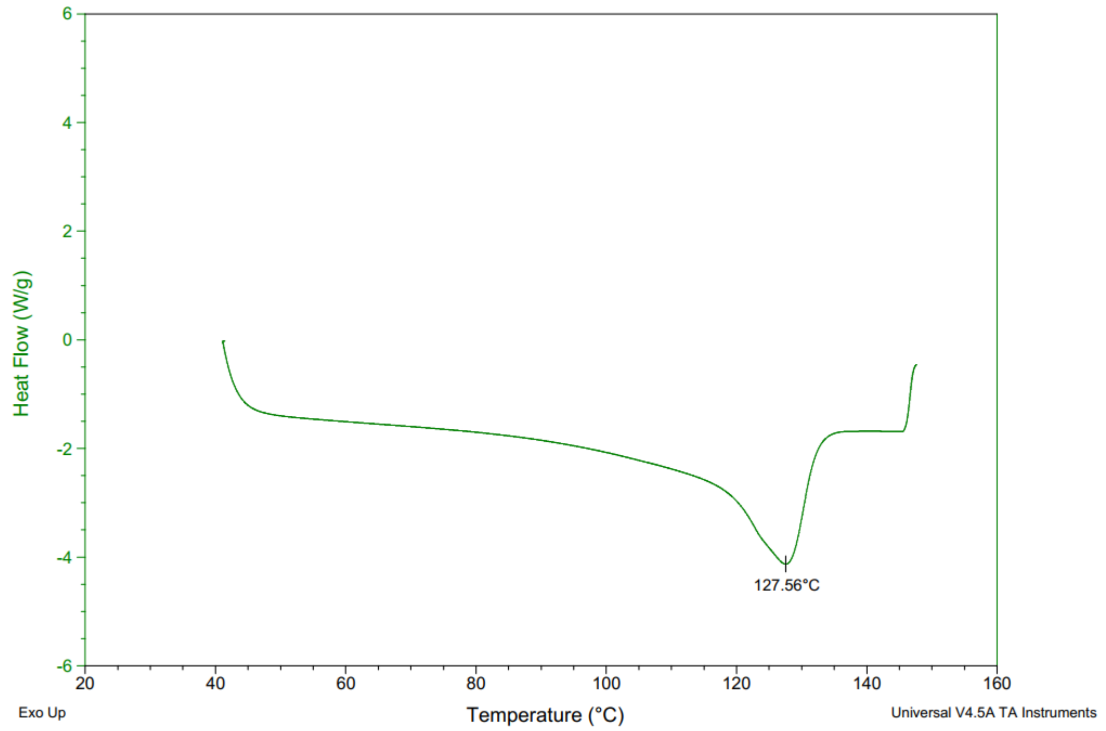

Supplementary Figure 120. DSC of the copolymer from Table 4, Entry 8.

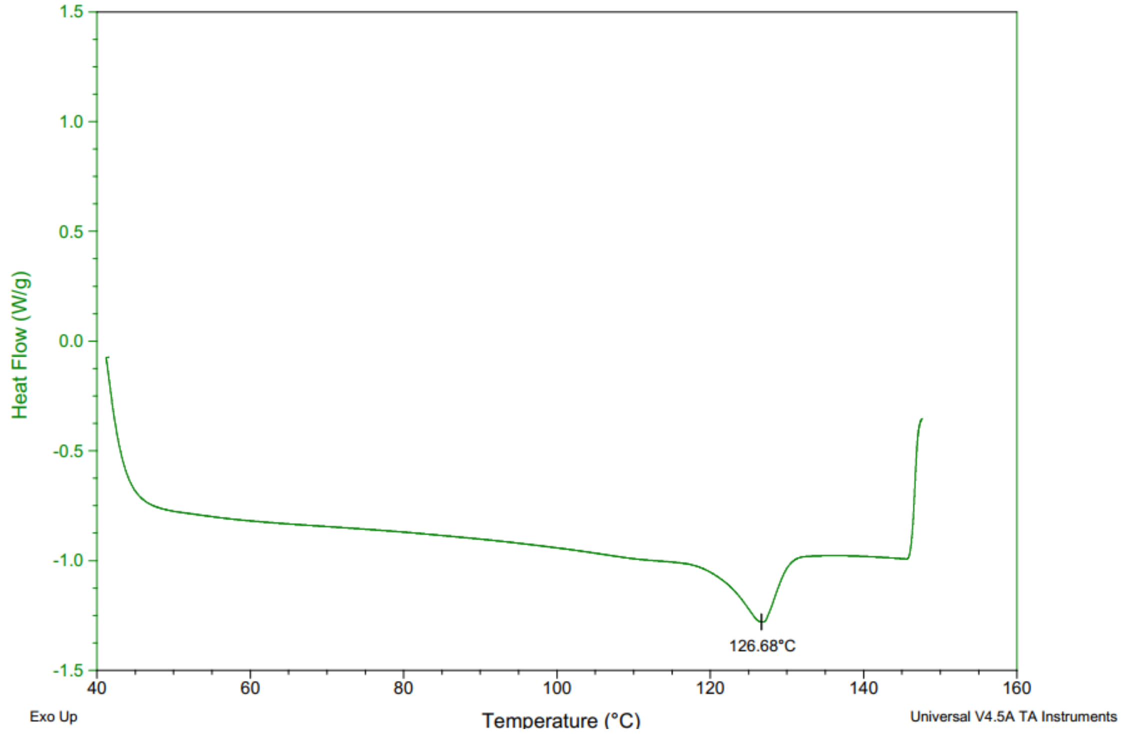

Supplementary Figure 121. DSC of the copolymer from Table 4, Entry 9.

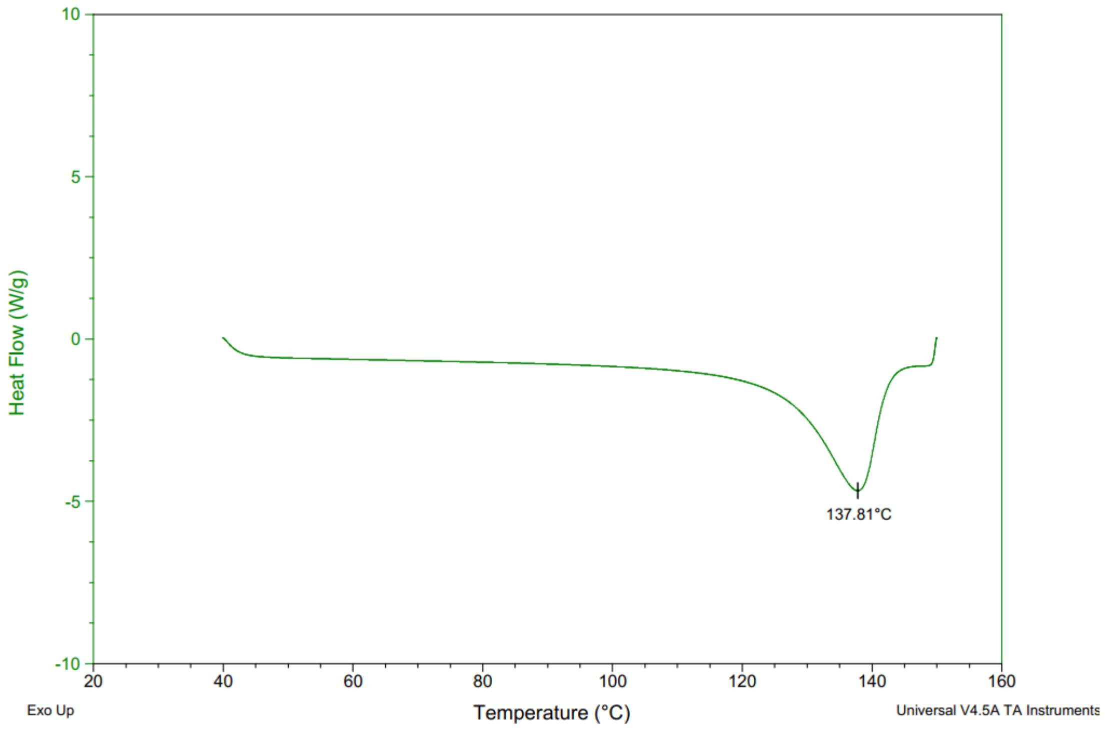

Supplementary Figure 122. DSC of the copolymer from Table 4, Entry 10.

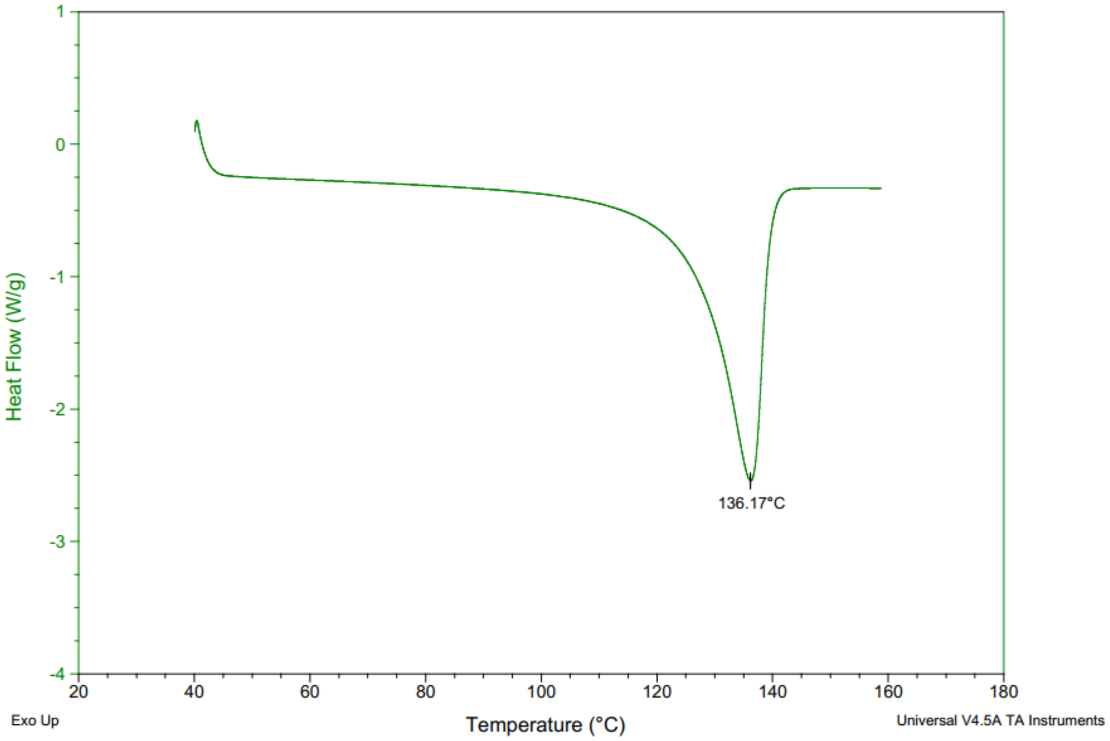

Supplementary Figure 123. DSC of the copolymer from Table 4, Entry 11.

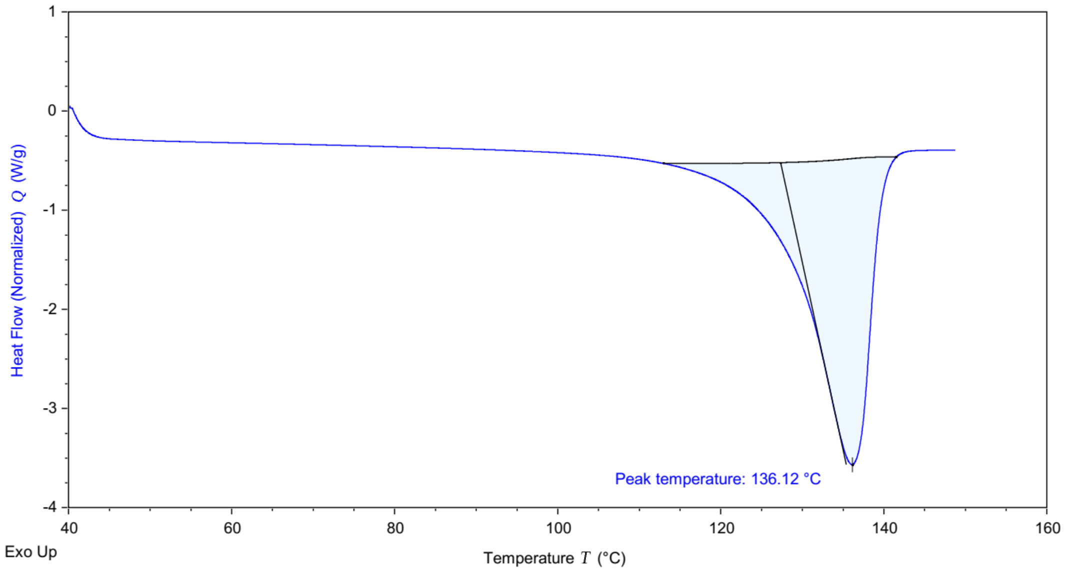

Supplementary Figure 124. DSC of the copolymer from Table 4, Entry 12.

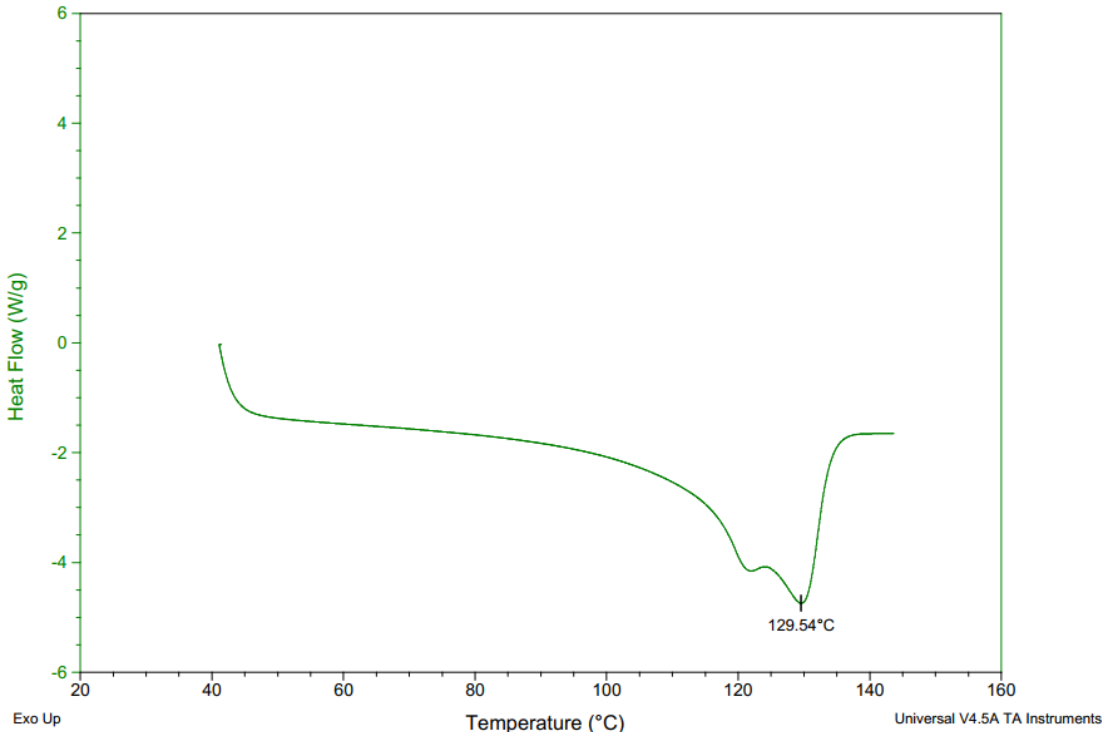

Supplementary Figure 125. DSC of the copolymer from Table 4, Entry 13.

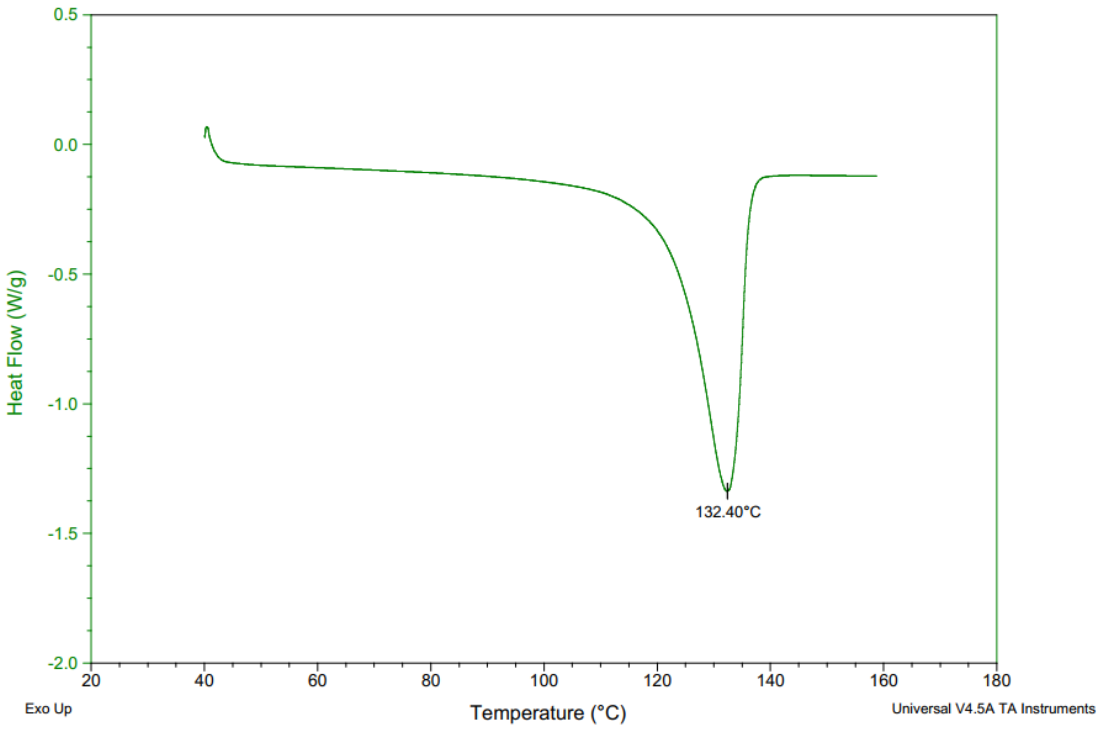

Supplementary Figure 126. DSC of the copolymer from Table 4, Entry 14.

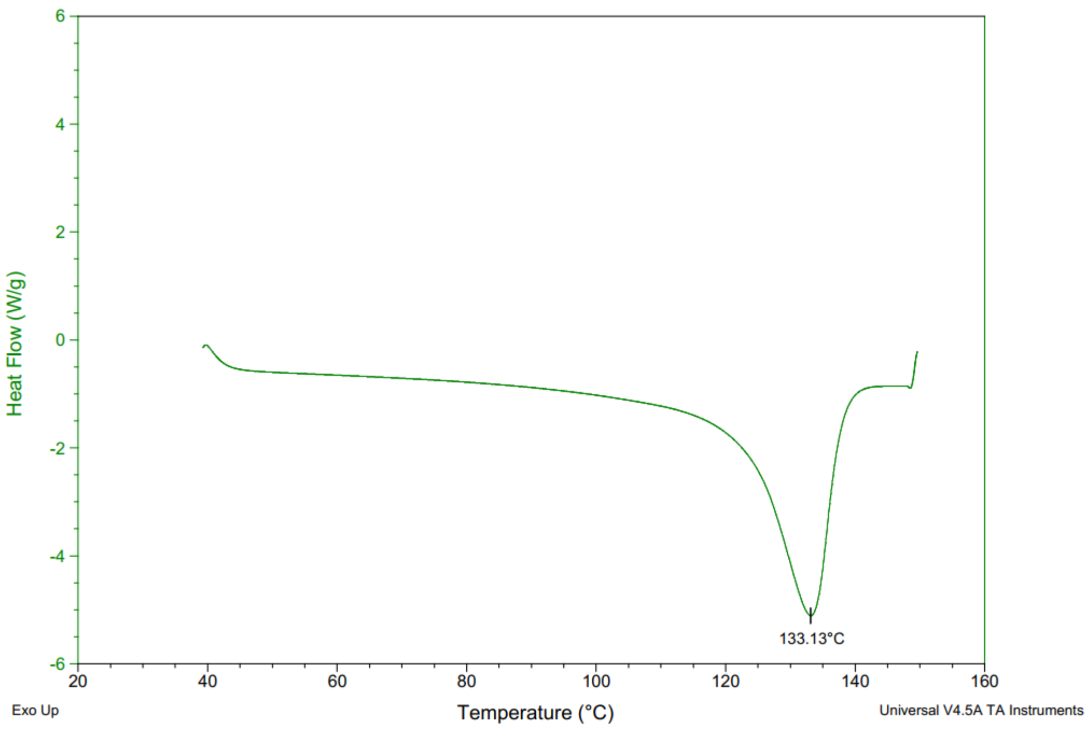

Supplementary Figure 127. DSC of the copolymer from Supplementary Table 5, Entry 1.

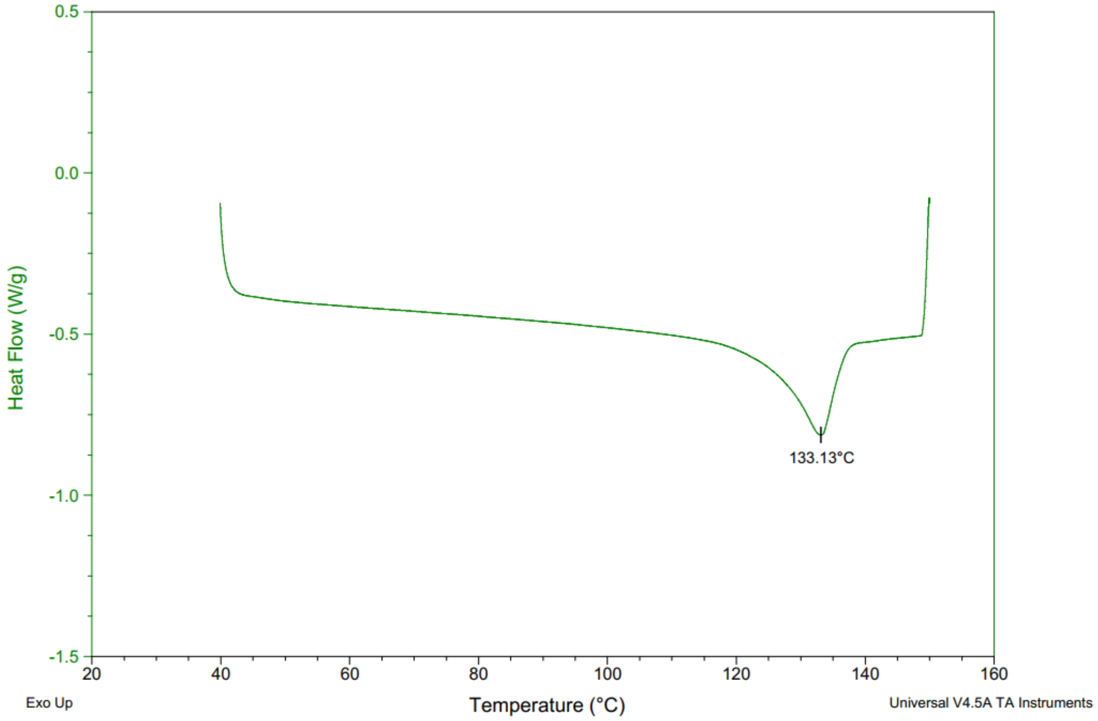

Supplementary Figure 128. DSC of the copolymer from Supplementary Table 5, Entry 2.

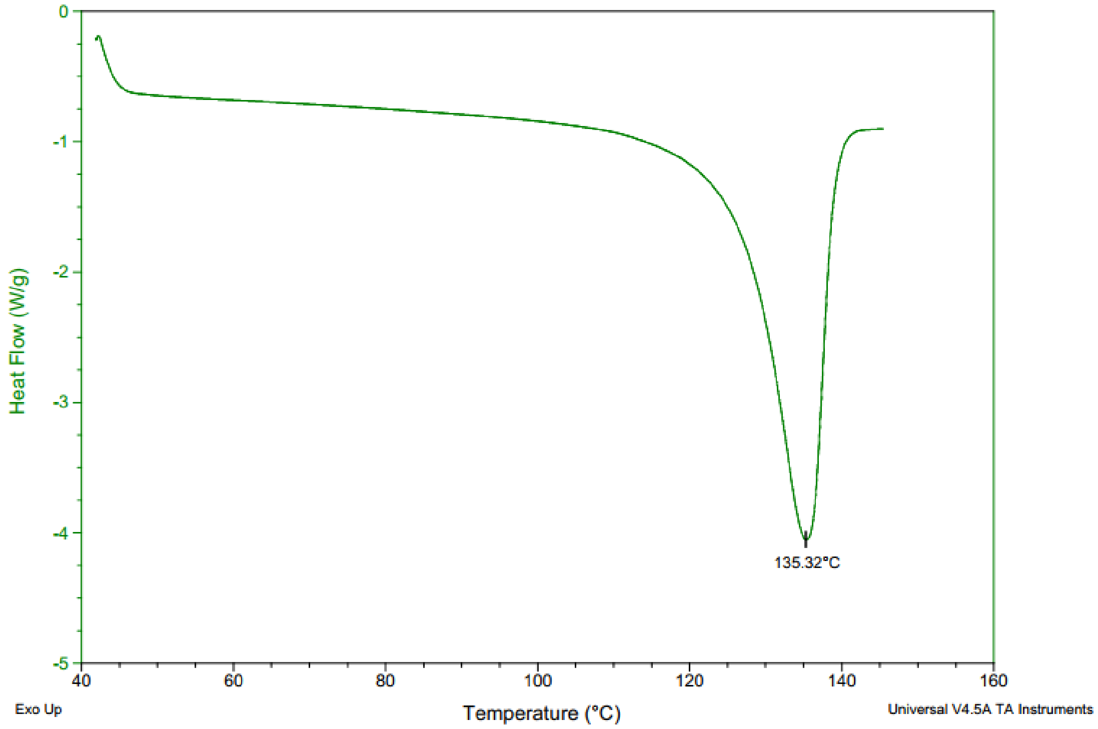

Supplementary Figure 129. DSC of the copolymer from Supplementary Table 5, Entry 3.

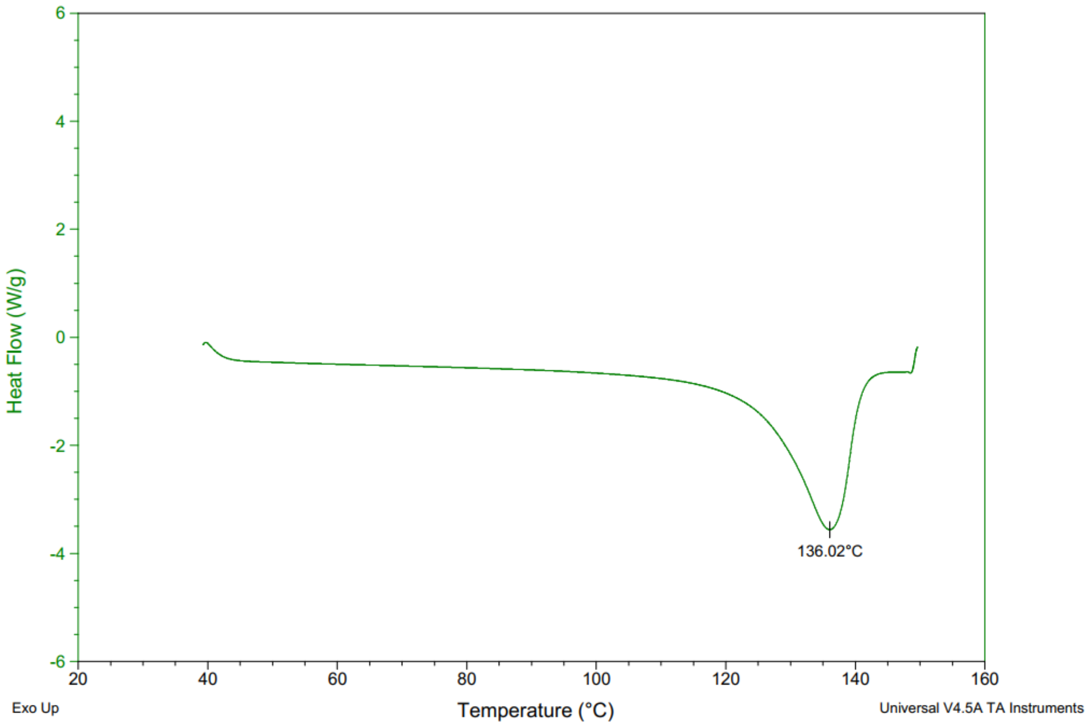

Supplementary Figure 130. DSC of the copolymer from Supplementary Table 5, Entry4.

6 Supplementary Figures of GPC of copolymers.

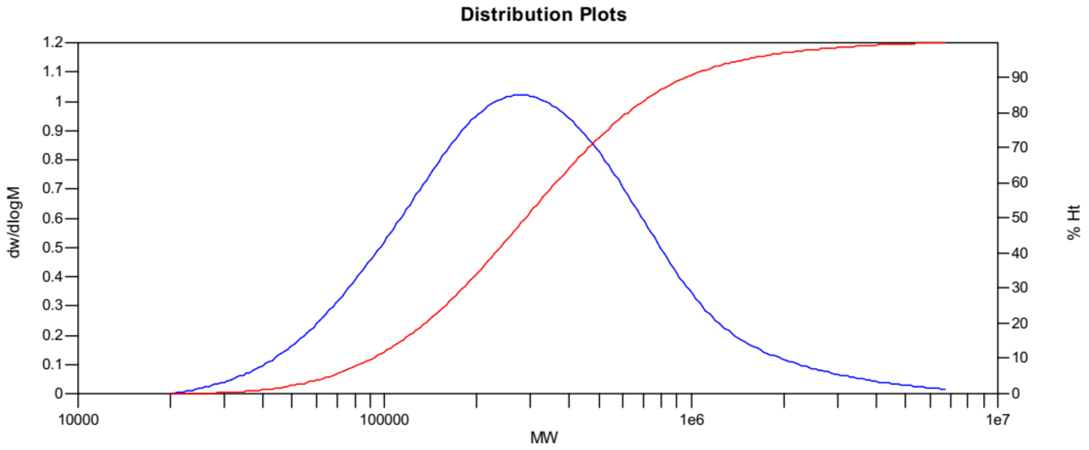

MW Averages

Peak No	Mp	Mn	Mw	Mz	Mz+1	Mv	PD
1	277496	194429	465494	1280292	2774457	401334	2.39416

Processed Peaks

Peak No	Name	Start RT (mins)	Max RT (mins)	End RT (mins)	Pk Height (mV)	% Height	Area (mV.secs)	% Area
1		11.05	13.25	15.07	-42.3402	100	3901.13	100

Supplementary Figure 131. GPC of the polymer from Table 1, Entry 1.

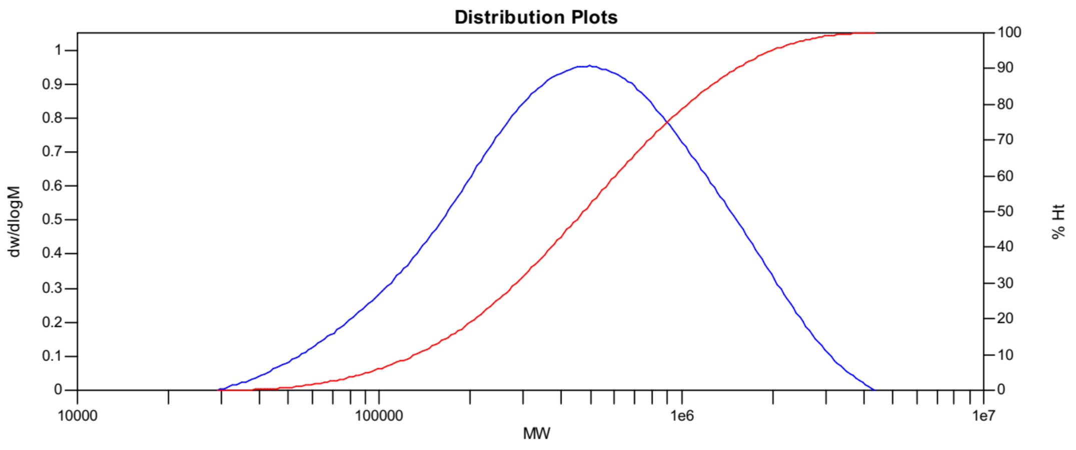

MW Averages

Peak No	Mp	Mn	Mw	Mz	Mz+1	Mv	PD
1	495909	292363	671220	1239319	1808409	601683	2.29584

Processed Peaks

Peak No	Name	Start RT (mins)	Max RT (mins)	End RT (mins)	Pk Height (mV)	% Height	Area (mV.secs)	% Area
1		11.33	12.83	14.78	-27.4957	100	2718.69	100

Supplementary Figure 132. GPC of the polymer from Table 1, Entry 2.

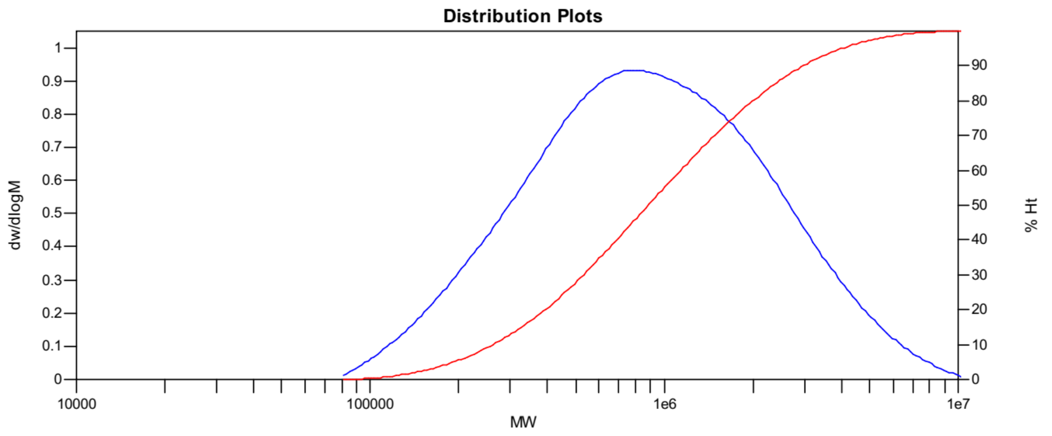

MW Averages

Peak No	Mp	Mn	Mw	Mz	Mz+1	Mv	PD
1	786611	584017	1323085	2623928	4103654	1173944	2.26549

Processed Peaks

Peak No	Name	Start RT (mins)	Max RT (mins)	End RT (mins)	Pk Height (mV)	% Height	Area (mV.secs)	% Area
1		10.77	12.60	14.12	-43.1105	100	4358.25	100

Supplementary Figure 133. GPC of the polymer from Table 1, Entry 3.

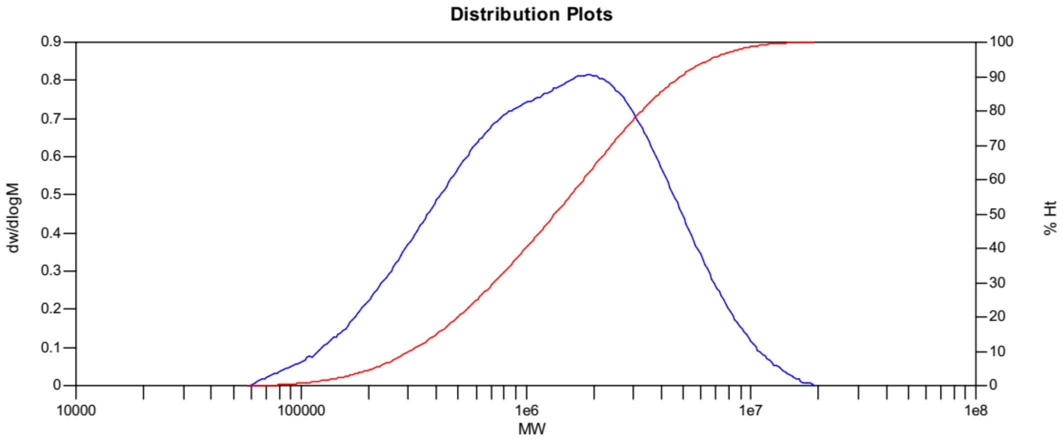

MW Averages

Peak No	Mp	Mn	Mw	Mz	Mz+1	Mv	PD
1	1879743	723677	2100881	4440585	6961620	1823419	2.90306

Processed Peaks

Peak No	Name	Start RT (mins)	Max RT (mins)	End RT (mins)	Pk Height (mV)	% Height	Area (mV.secs)	% Area
1		10.37	12.00	14.37	-12.3714	0	1436.59	100

Supplementary Figure 134. GPC of the polymer from Table 1, Entry 4.

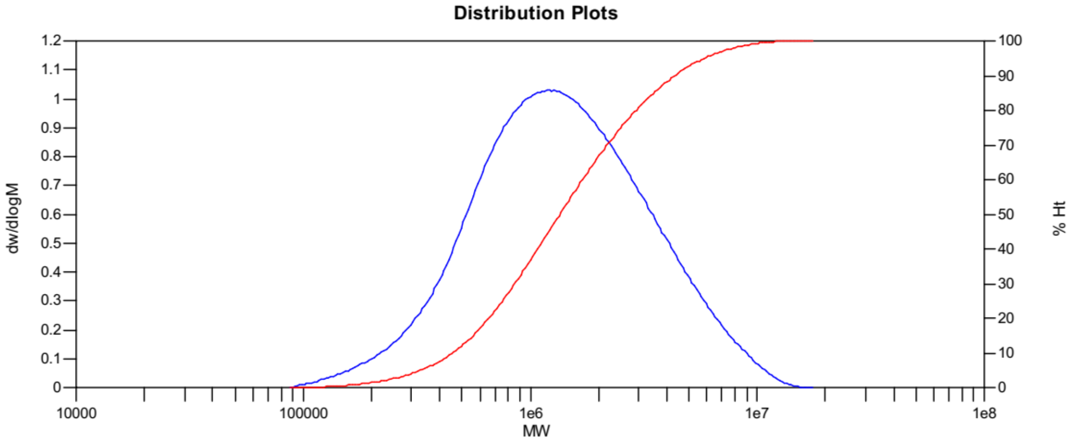

MW Averages

Peak No	Mp	Mn	Mw	Mz	Mz+1	Mv	PD
1	1159358	943602	1980842	3735122	5747018	1780758	2.09923

Processed Peaks

Peak No	Name	Start RT (mins)	Max RT (mins)	End RT (mins)	Pk Height (mV)	% Height	Area (mV.secs)	% Area
1		9.95	11.90	13.67	-36.2054	100	3361.59	100

Supplementary Figure 135. GPC of the polymer from Table 1, Entry 6.

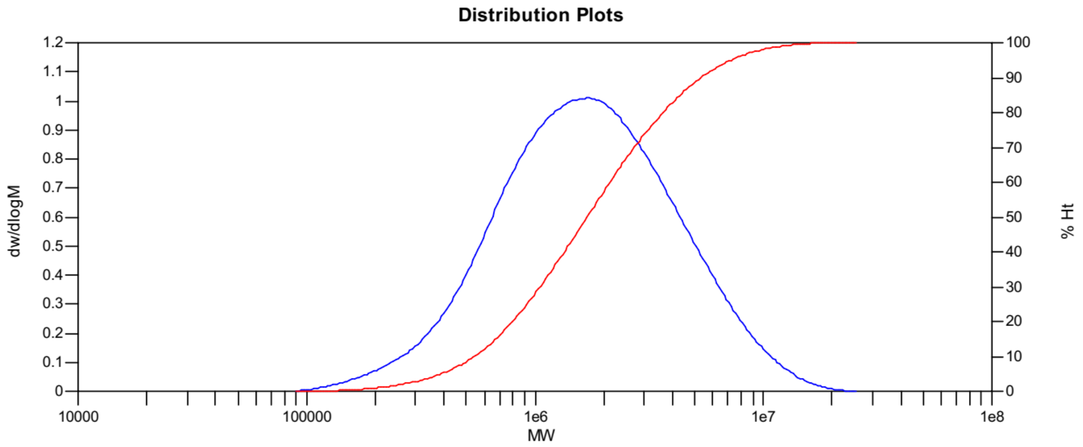

MW Averages

Peak No	Mp	Mn	Mw	Mz	Mz+1	Mv	PD
1	1685352	1134352	2457479	4745431	7598374	2202702	2.16642

Processed Peaks

Peak No	Name	Start RT (mins)	Max RT (mins)	End RT (mins)	Pk Height (mV)	% Height	Area (mV.secs)	% Area
1		10.08	12.02	14.02	-45.5217	100	4289.36	100

Supplementary Figure 136. GPC of the polymer from Table 1, Entry 7.

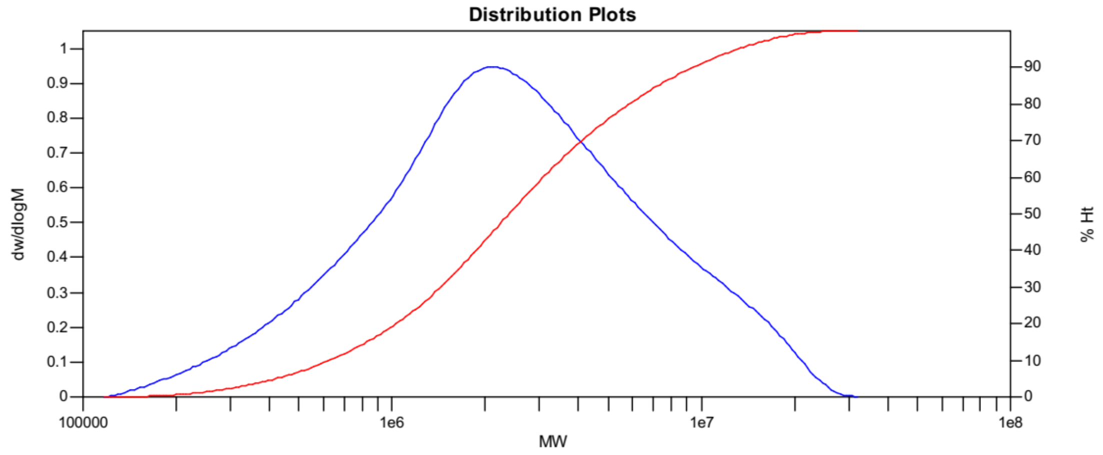

MW Averages

Peak No	Mp	Mn	Mw	Mz	Mz+1	Mv	PD
1	2131356	1438553	3918677	8369335	12814972	3418698	2.72404

Processed Peaks

Peak No	Name	Start RT (mins)	Max RT (mins)	End RT (mins)	Pk Height (mV)	% Height	Area (mV.secs)	% Area
1		9.88	11.83	13.82	-29.1969	100	2947.05	100

Supplementary Figure 137. GPC of the polymer from Table 1, Entry 8.

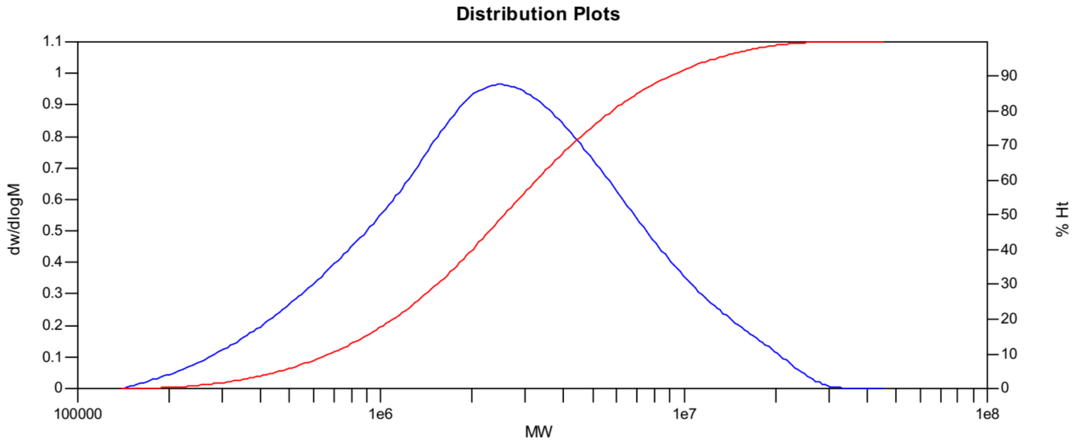

MW Averages

Peak No	Mp	Mn	Mw	Mz	Mz+1	Mv	PD
1	2438576	1549791	3928549	8153489	12850829	3444196	2.53489

Processed Peaks

Peak No	Name	Start RT (mins)	Max RT (mins)	End RT (mins)	Pk Height (mV)	% Height	Area (mV.secs)	% Area
1		9.70	11.80	13.75	-22.6493	0	2234.18	100

Supplementary Figure 138. GPC of the polymer from Table 1, Entry 9.

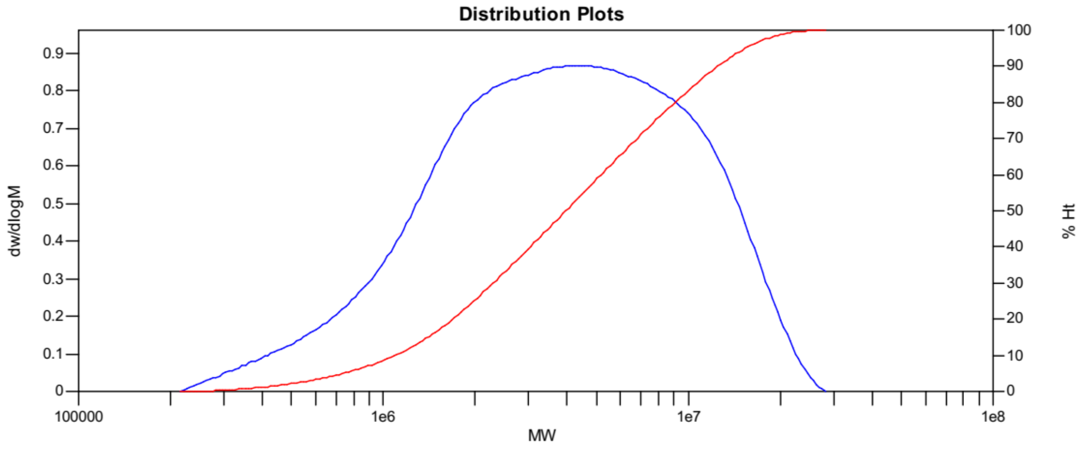

MW Averages

Peak No	Mp	Mn	Mw	Mz	Mz+1	Mv	PD
1	3971401	2410275	5569044	9553933	12836823	5029794	2.31054

Processed Peaks

Peak No	Name	Start RT (mins)	Max RT (mins)	End RT (mins)	Pk Height (mV)	% Height	Area (mV.secs)	% Area
1		9.98	11.47	13.40	-28.2973	100	3175.62	100

Supplementary Figure 139. GPC of the polymer from Table 1, Entry 10.

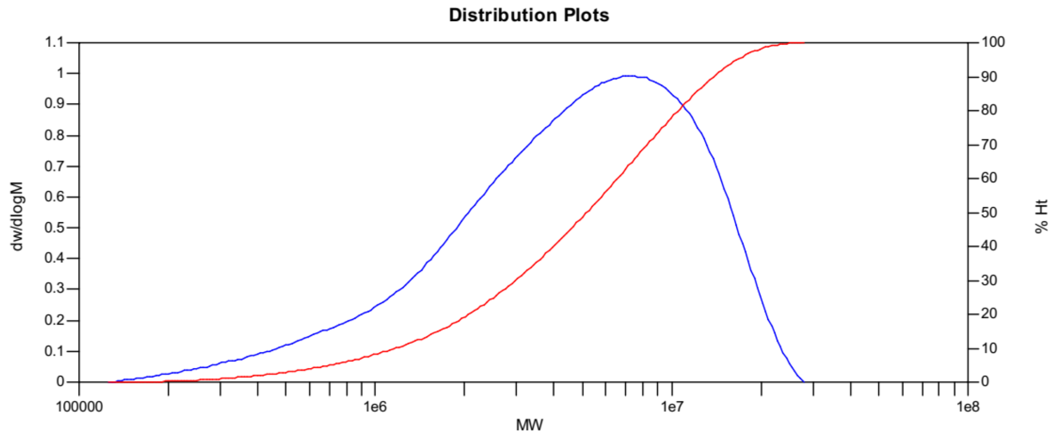

MW Averages

Peak No	Mp	Mn	Mw	Mz	Mz+1	Mv	PD
1	6989929	2568815	6510692	10389763	13346420	5918247	2.53451

Processed Peaks

Peak No	Name	Start RT (mins)	Max RT (mins)	End RT (mins)	Pk Height (mV)	% Height	Area (mV.secs)	% Area
1		10.07	11.07	13.82	-19.3257	0	1898.99	100

Supplementary Figure 140. GPC of the polymer from Table 1, Entry 11.

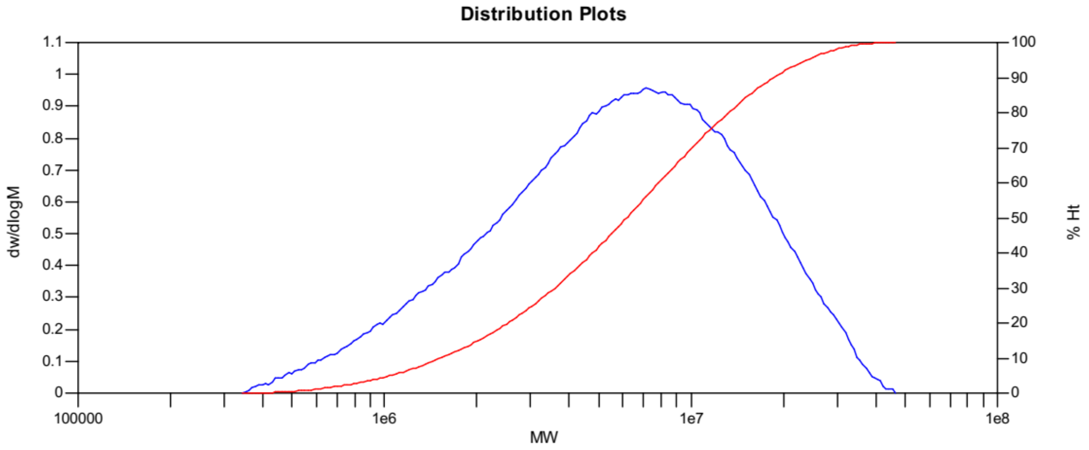

MW Averages

Peak No	Mp	Mn	Mw	Mz	Mz+1	Mv	PD
1	7156925	3594901	8467558	14740206	20342604	7610278	2.35544

Processed Peaks

Peak No	Name	Start RT (mins)	Max RT (mins)	End RT (mins)	Pk Height (mV)	% Height	Area (mV.secs)	% Area
1		9.68	11.05	13.13	-4.55007	0	463.679	100

Supplementary Figure 141. GPC of the polymer from Table 1, Entry 12.

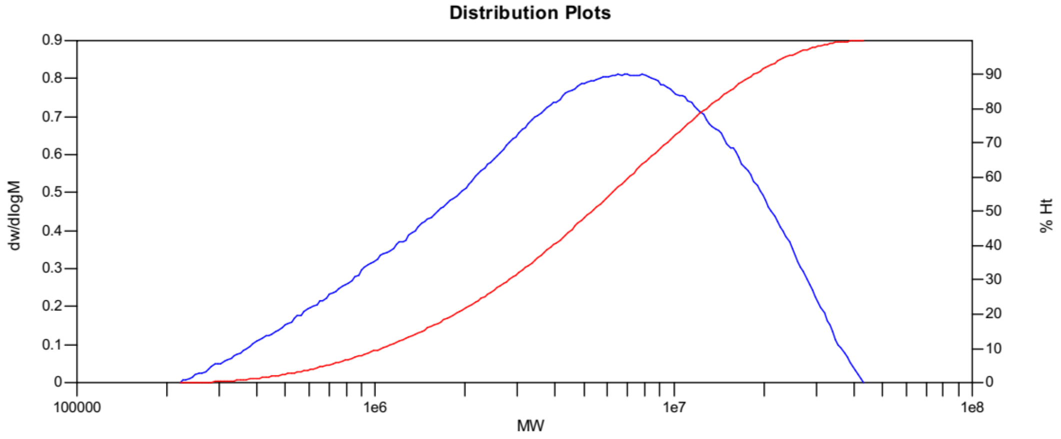

MW Averages

Peak No	Mp	Mn	Mw	Mz	Mz+1	Mv	PD
1	6511327	2640036	7883707	14860563	20486232	6926153	2.98621

Processed Peaks

Peak No	Name	Start RT (mins)	Max RT (mins)	End RT (mins)	Pk Height (mV)	% Height	Area (mV.secs)	% Area
1		9.73	11.15	13.43	-5.90438	0	708.275	100

Supplementary Figure 142. GPC of the polymer from Table 1, Entry 13.

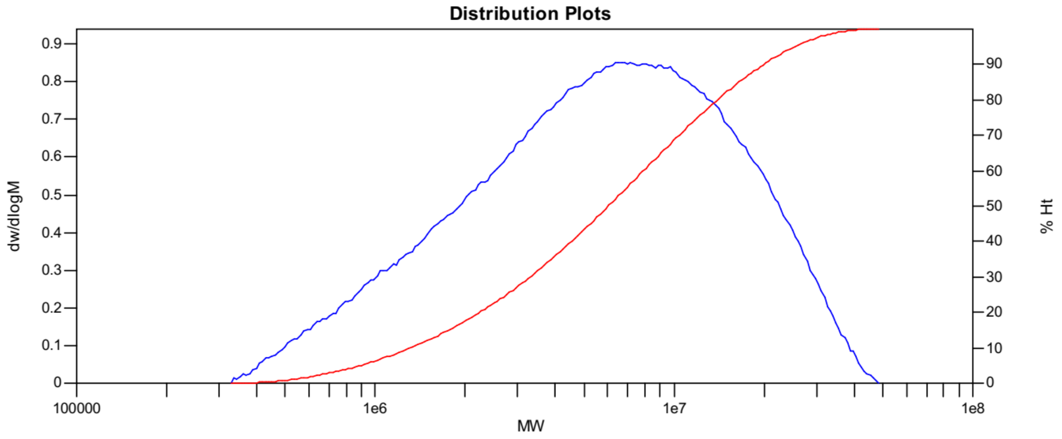

MW Averages

Peak No	Mp	Mn	Mw	Mz	Mz+1	Mv	PD
1	6358986	3265348	8703294	15887821	21878068	7717581	2.66535

Processed Peaks

Peak No	Name	Start RT (mins)	Max RT (mins)	End RT (mins)	Pk Height (mV)	% Height	Area (mV.secs)	% Area
1		9.65	11.13	13.17	-4.1909	0	478.17	100

Supplementary Figure 143. GPC of the polymer from Table 1, Entry 14.

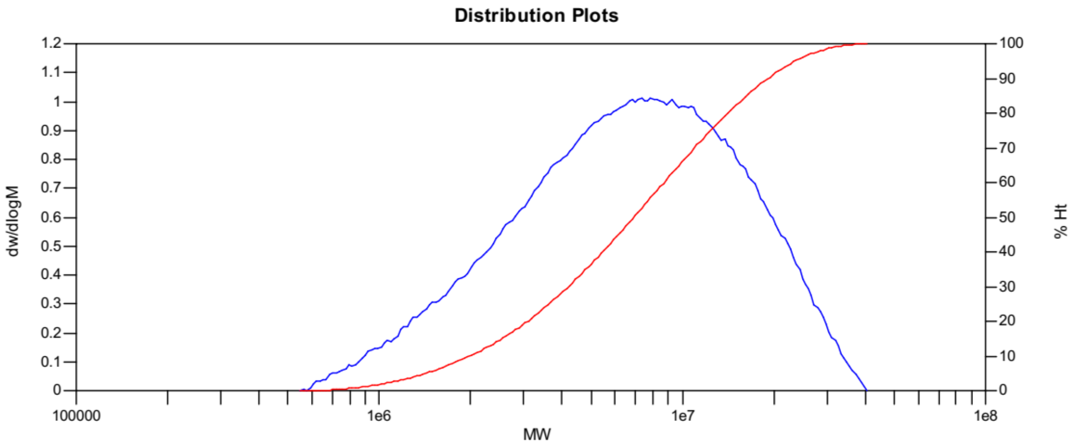

MW Averages

Peak No	Mp	Mn	Mw	Mz	Mz+1	Mv	PD
1	7327789	4492044	9025541	14518290	19269320	8247912	2.00923

Processed Peaks

Peak No	Name	Start RT (mins)	Max RT (mins)	End RT (mins)	Pk Height (mV)	% Height	Area (mV.secs)	% Area
1		9.78	11.03	12.82	-3.70375	0	357.292	100

Supplementary Figure 144. GPC of the polymer from Table 1, Entry 15.

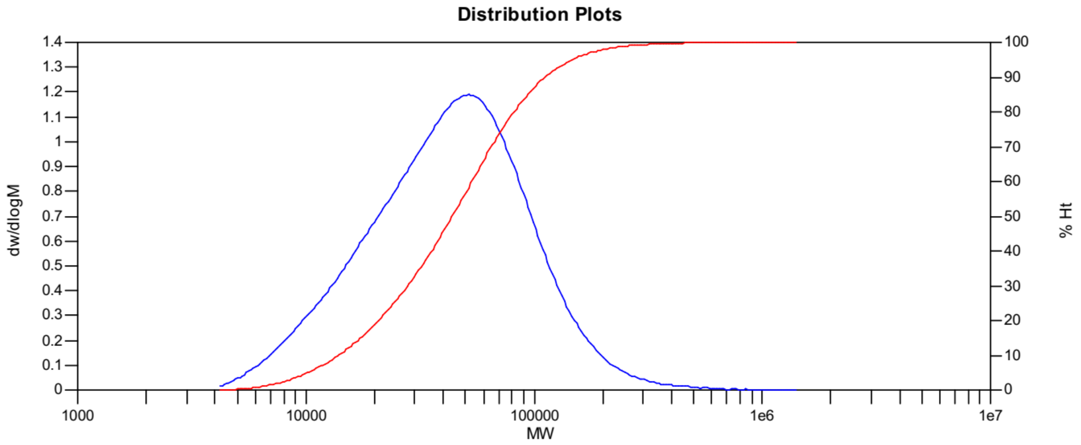

MW Averages

Peak No	Mp	Mn	Mw	Mz	Mz+1	Mv	PD
1	51574	29177	55854	104544	200846	50856	1.91432

Processed Peaks

Peak No	Name	Start RT (mins)	Max RT (mins)	End RT (mins)	Pk Height (mV)	% Height	Area (mV.secs)	% Area
1		12.12	14.38	16.18	-21.7384	0	1749.74	100

Supplementary Figure 145. GPC of the copolymer from Table 2, Entry 1.

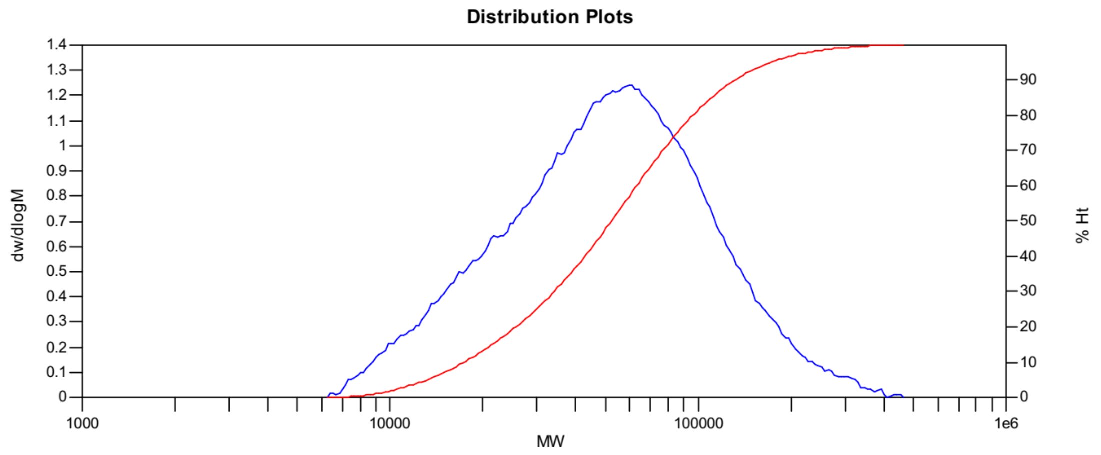

MW Averages

Peak No	Mp	Mn	Mw	Mz	Mz+1	Mv	PD
1	59531	36331	64847	107492	160717	59572	1.78489

Processed Peaks

Peak No	Name	Start RT (mins)	Max RT (mins)	End RT (mins)	Pk Height (mV)	% Height	Area (mV.secs)	% Area
1		12.95	14.35	15.95	-3.05368	0	233.793	100

Supplementary Figure 146. GPC of the copolymer from Table 2, Entry 2.

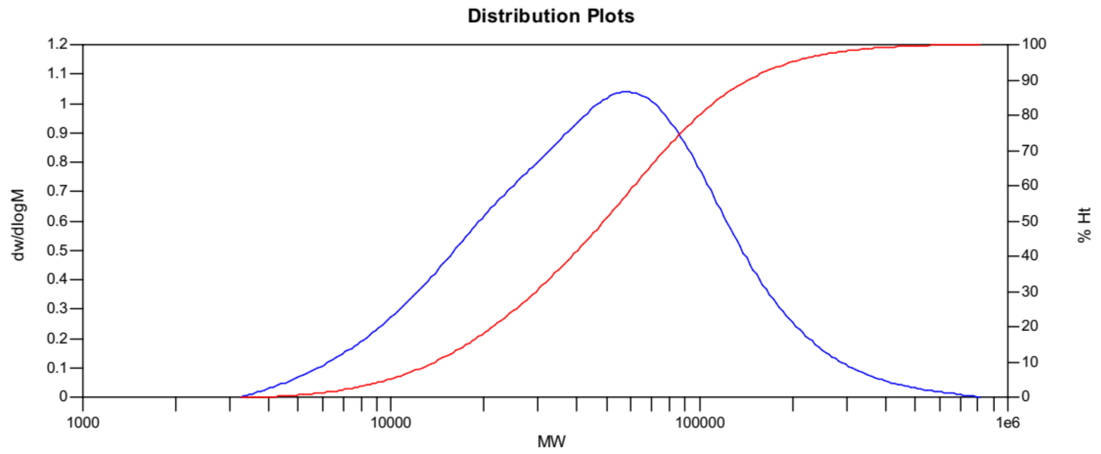

MW Averages

Peak No	Mp	Mn	Mw	Mz	Mz+1	Mv	PD
1	57720	29938	67612	139339	249833	59991	2.2584

Processed Peaks

Peak No	Name	Start RT (mins)	Max RT (mins)	End RT (mins)	Pk Height (mV)	% Height	Area (mV.secs)	% Area
1		12.55	14.35	16.42	-33.8264	0	3095.42	100

Supplementary Figure 147. GPC of the copolymer from Table 2, Entry 3.

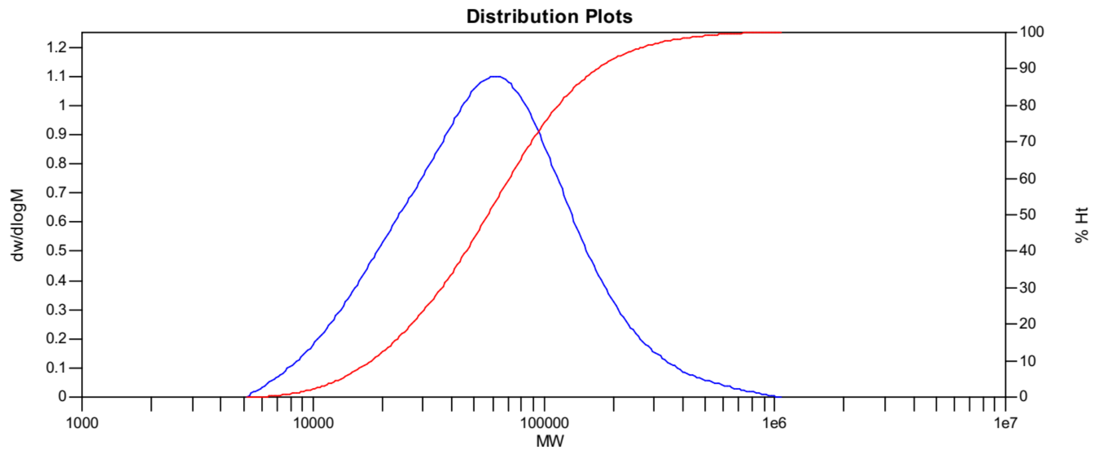

MW Averages

Peak No	Mp	Mn	Mw	Mz	Mz+1	Mv	PD
1	63593	38144	81326	176714	337004	72015	2.13208

Processed Peaks

Peak No	Name	Start RT (mins)	Max RT (mins)	End RT (mins)	Pk Height (mV)	% Height	Area (mV.secs)	% Area
1		12.37	14.28	16.07	-16.9495	100	1465.9	100

Supplementary Figure 148. GPC of the copolymer from Table 2, Entry 4.

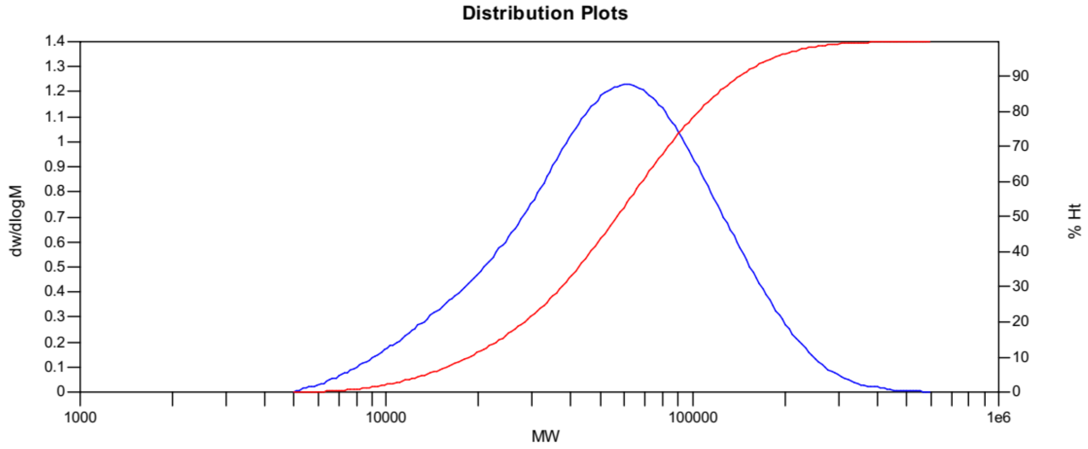

MW Averages

Peak No	Mp	Mn	Mw	Mz	Mz+1	Mv	PD
1	60989	38354	69621	112729	165628	64108	1.81522

Processed Peaks

Peak No	Name	Start RT (mins)	Max RT (mins)	End RT (mins)	Pk Height (mV)	% Height	Area (mV.secs)	% Area
1		12.78	14.33	16.12	-11.1386	0	860.964	100

Supplementary Figure 149. GPC of the copolymer from Table 2, Entry 5.

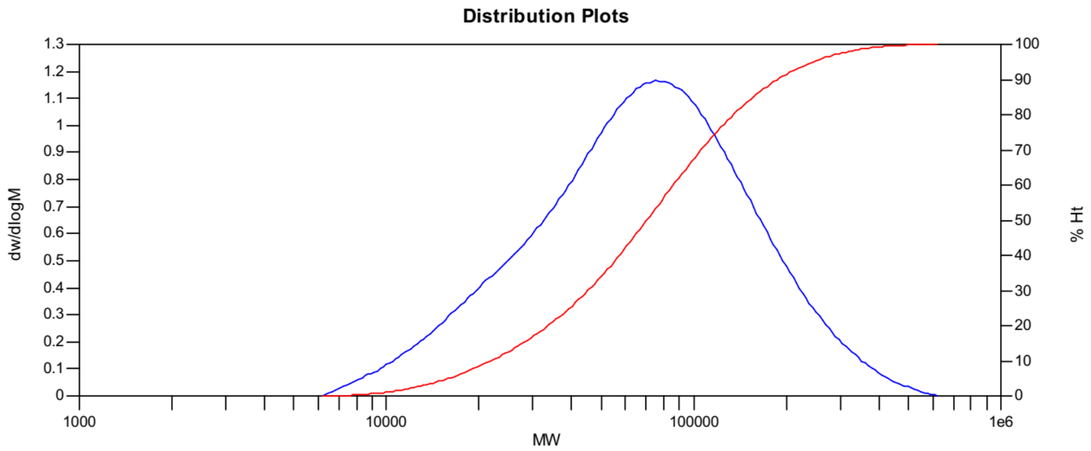

MW Averages

Peak No	Mp	Mn	Mw	Mz	Mz+1	Mv	PD
1	75372	46601	89549	151423	222817	81735	1.92161

Processed Peaks

Peak No	Name	Start RT (mins)	Max RT (mins)	End RT (mins)	Pk Height (mV)	% Height	Area (mV.secs)	% Area
1		12.73	14.17	15.93	-16.6803	0	1355.62	100

Supplementary Figure 150. GPC of the copolymer from Table 2, Entry 6.

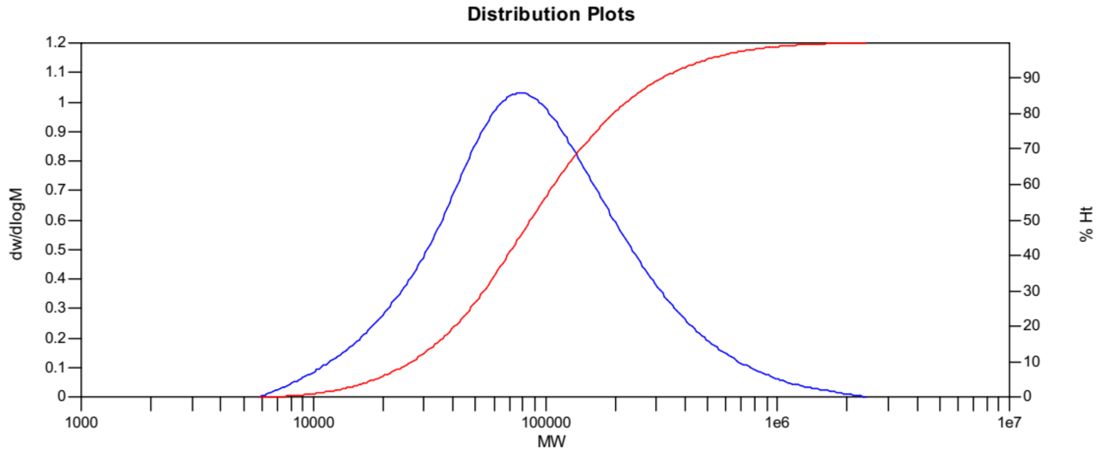

MW Averages

Peak No	Mp	Mn	Mw	Mz	Mz+1	Mv	PD
1	77720	56262	143546	391238	816460	123149	2.55138

Processed Peaks

Peak No	Name	Start RT (mins)	Max RT (mins)	End RT (mins)	Pk Height (mV)	% Height	Area (mV.secs)	% Area
1		11.75	14.10	15.93	-42.6688	100	3936.68	100

Supplementary Figure 151. GPC of the copolymer from Table 2, Entry 7.

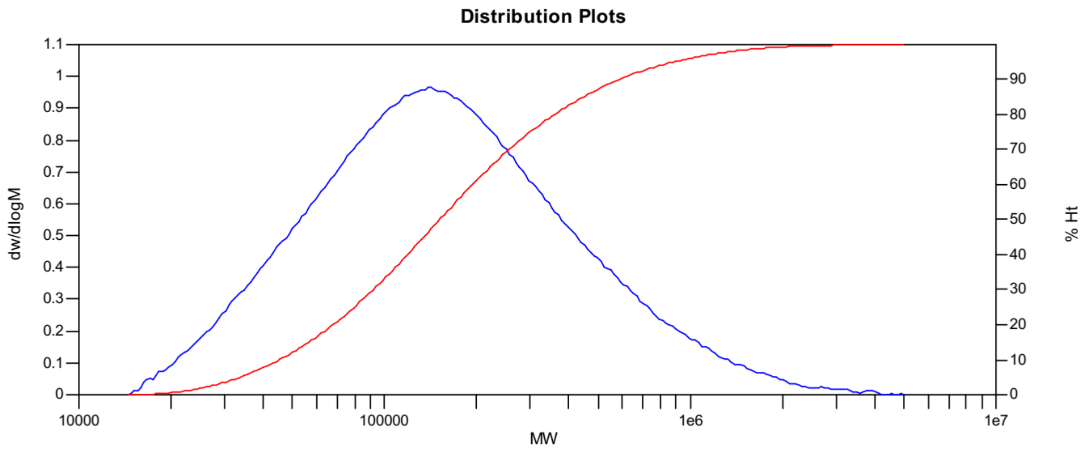

MW Averages

Peak No	Mp	Mn	Mw	Mz	Mz+1	Mv	PD
1	142177	100899	258689	712257	1497223	219835	2.56384

Processed Peaks

Peak No	Name	Start RT (mins)	Max RT (mins)	End RT (mins)	Pk Height (mV)	% Height	Area (mV.secs)	% Area
1		11.30	13.75	15.32	-5.34971	100	521.301	100

Supplementary Figure 152. GPC of the polymer from Table 2, Entry 8.

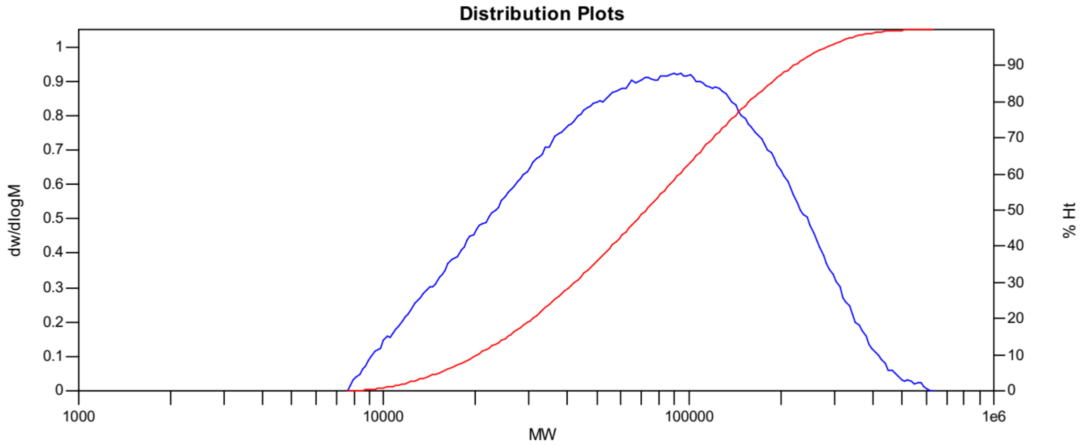

MW Averages

Peak No	Mp	Mn	Mw	Mz	Mz+1	Mv	PD
1	93837	45835	97888	172455	243715	88077	2.13566

Processed Peaks

Peak No	Name	Start RT (mins)	Max RT (mins)	End RT (mins)	Pk Height (mV)	% Height	Area (mV.secs)	% Area
1		12.72	13.97	15.78	-3.37359	100	346.588	100

Supplementary Figure 153. GPC of the polymer from Table 1, Entry 9.

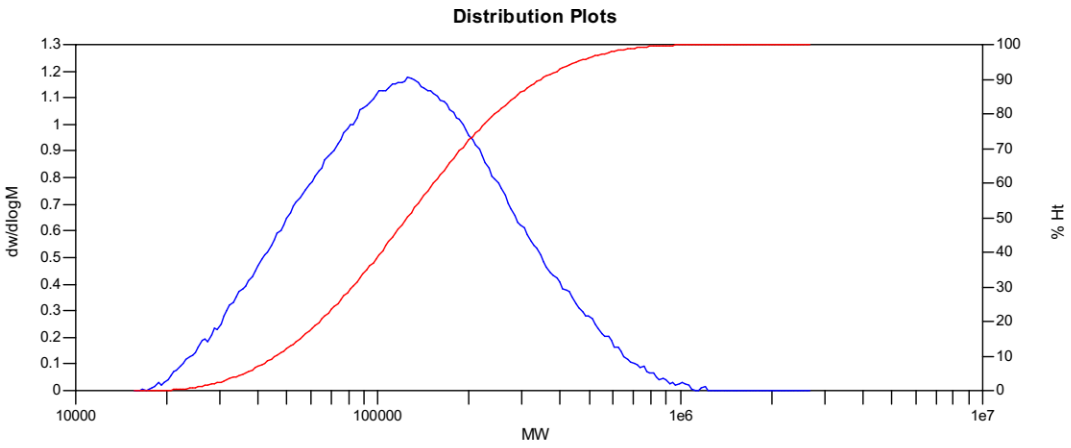

MW Averages

Peak No	Mp	Mn	Mw	Mz	Mz+1	Mv	PD
1	125797	93473	165444	283810	429989	151283	1.76997

Processed Peaks

Peak No	Name	Start RT (mins)	Max RT (mins)	End RT (mins)	Pk Height (mV)	% Height	Area (mV.secs)	% Area
1		11.73	13.90	15.27	-2.49861	0	203.992	100

Supplementary Figure 154. GPC of the polymer from Table 1, Entry 10.

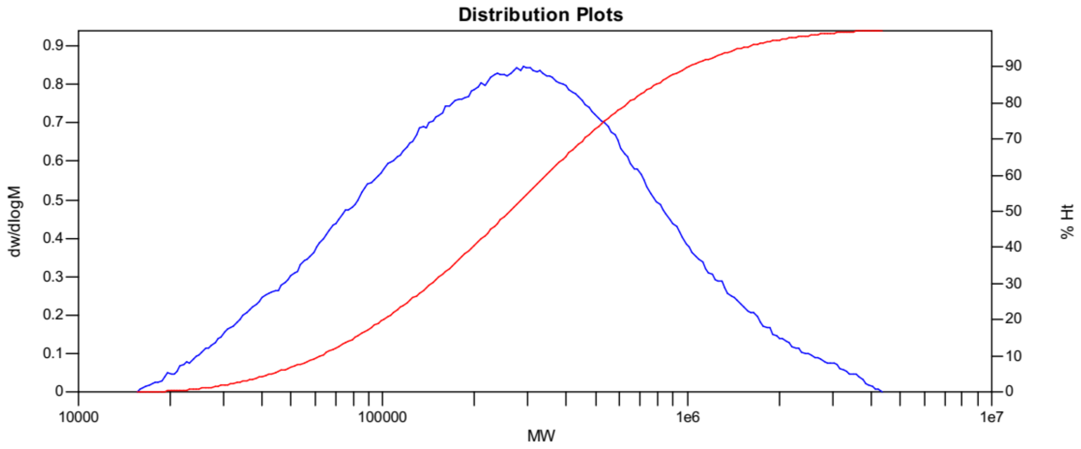

MW Averages

Peak No	Mp	Mn	Mw	Mz	Mz+1	Mv	PD
1	289771	146596	432711	1049841	1793082	368665	2.95172

Processed Peaks

Peak No	Name	Start RT (mins)	Max RT (mins)	End RT (mins)	Pk Height (mV)	% Height	Area (mV.secs)	% Area
1		11.40	13.25	15.27	-3.735	0	412.54	100

Supplementary Figure 155. GPC of the polymer from Table 1, Entry 11.

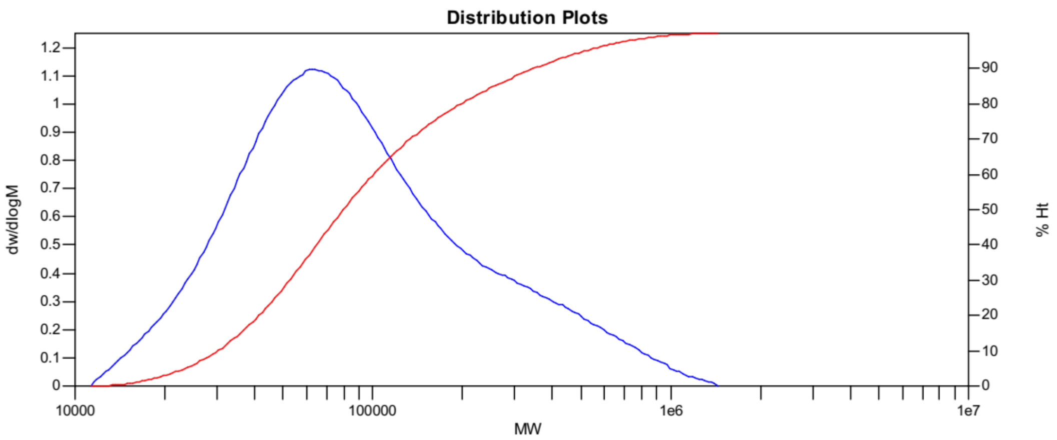

MW Averages

Peak No	Mp	Mn	Mw	Mz	Mz+1	Mv	PD
1	60989	61581	140916	346225	596040	121125	2.2883

Processed Peaks

Peak No	Name	Start RT (mins)	Max RT (mins)	End RT (mins)	Pk Height (mV)	% Height	Area (mV.secs)	% Area
1		12.18	14.32	15.52	-10.8061	100	916.007	100

Supplementary Figure 156. GPC of the copolymer from Table 2, Entry 12.

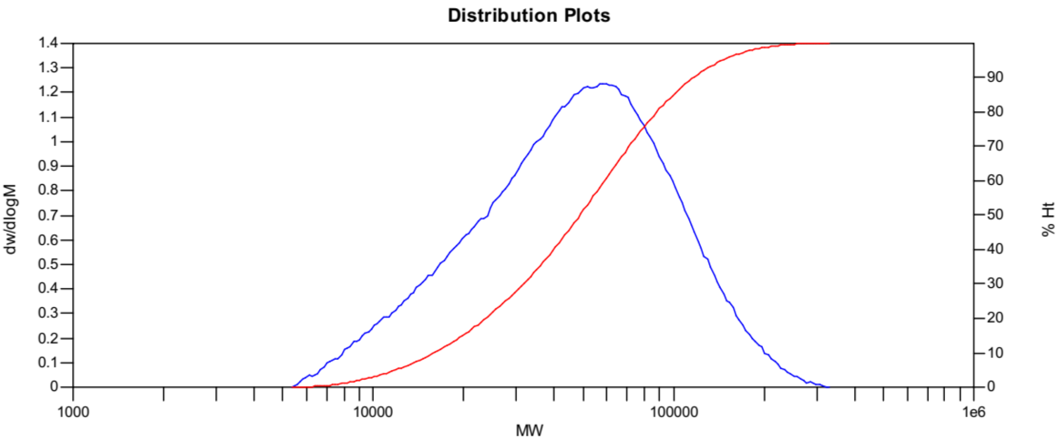

MW Averages

Peak No	Mp	Mn	Mw	Mz	Mz+1	Mv	PD
1	58107	33203	57903	88709	120498	53680	1.74391

Processed Peaks

Peak No	Name	Start RT (mins)	Max RT (mins)	End RT (mins)	Pk Height (mV)	% Height	Area (mV.secs)	% Area
1		13.18	14.37	16.07	-4.59677	0	353.438	100

Supplementary Figure 157. GPC of the copolymer from Table 2, Entry 13.

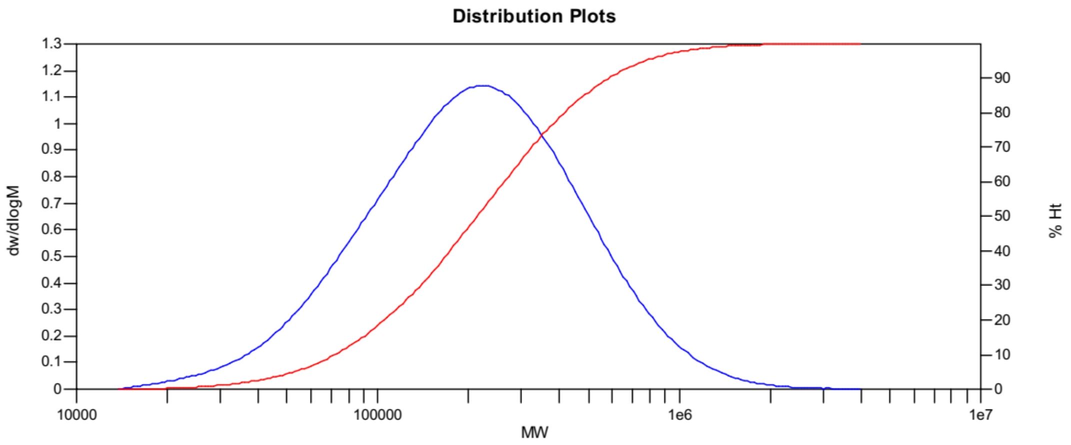

MW Averages

Peak No	Mp	Mn	Mw	Mz	Mz+1	Mv	PD
1	228214	146088	281818	507123	846298	256818	1.9291

Processed Peaks

Peak No	Name	Start RT (mins)	Max RT (mins)	End RT (mins)	Pk Height (mV)	% Height	Area (mV.secs)	% Area
1		11.42	13.38	15.33	-54.3295	100	4481.2	100

Supplementary Figure 158. GPC of the copolymer from Table 2, Entry 14.

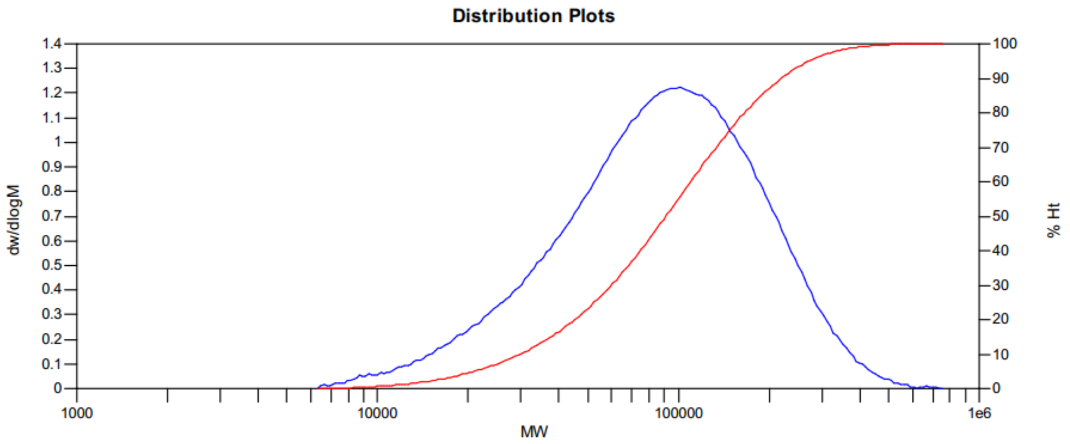

MW Averages

Peak No	Mp	Mn	Mw	Mz	Mz+1	Mv	PD
1	100962	60026	109308	168969	232884	101159	1.82101

Processed Peaks

Peak No	Name	Start RT (mins)	Max RT (mins)	End RT (mins)	Pk Height (mV)	% Height	Area (mV.secs)	% Area
1		12.60	13.97	15.92	-6.05662	100	467.398	100

Supplementary Figure 159. GPC of the copolymer from Table 2, Entry 15.

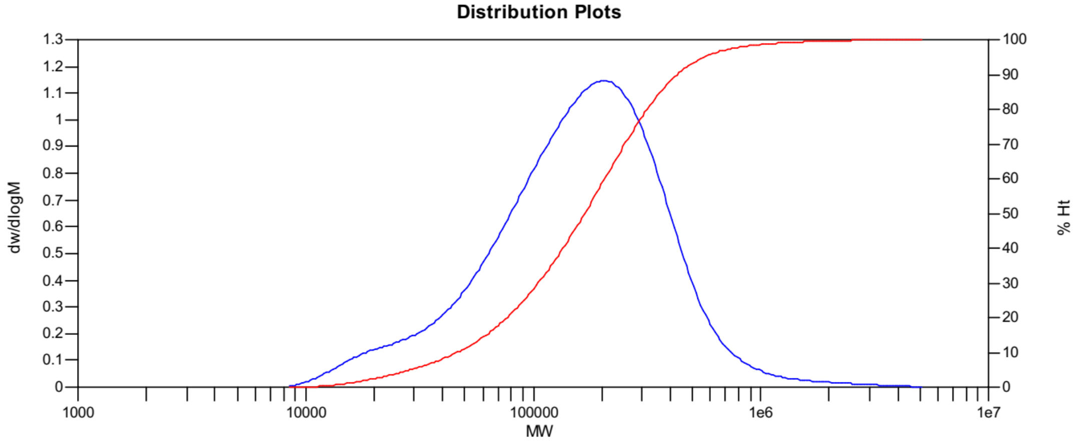

MW Averages

Peak No	Mp	Mn	Mw	Mz	Mz+1	Mv	PD
1	204324	96933	221125	499159	1288490	197370	2.28121

Processed Peaks

Peak No	Name	Start RT (mins)	Max RT (mins)	End RT (mins)	Pk Height (mV)	% Height	Area (mV.secs)	% Area
1		11.28	13.48	15.70	-65.5887	100	5379.87	100

Supplementary Figure 160. GPC of the copolymer from Table 2, Entry 16.

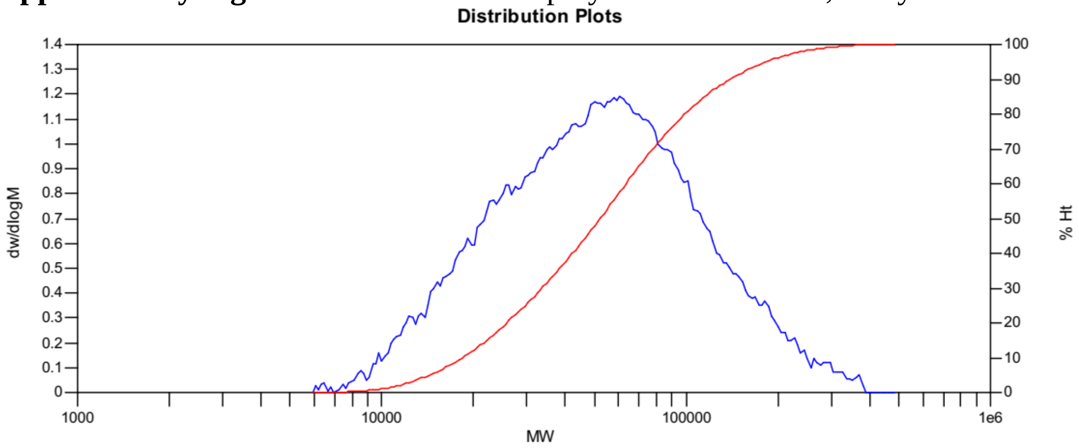

MW Averages

Peak No	Mp	Mn	Mw	Mz	Mz+1	Mv	PD
1	60584	37548	66807	112440	166112	61199	1.77924

Processed Peaks

Peak No	Name	Start RT (mins)	Max RT (mins)	End RT (mins)	Pk Height (mV)	% Height	Area (mV.secs)	% Area
1		12.90	14.32	15.97	-1.21324	100	96.9604	100

Supplementary Figure 161. GPC of the copolymer from Table 2, Entry 17.

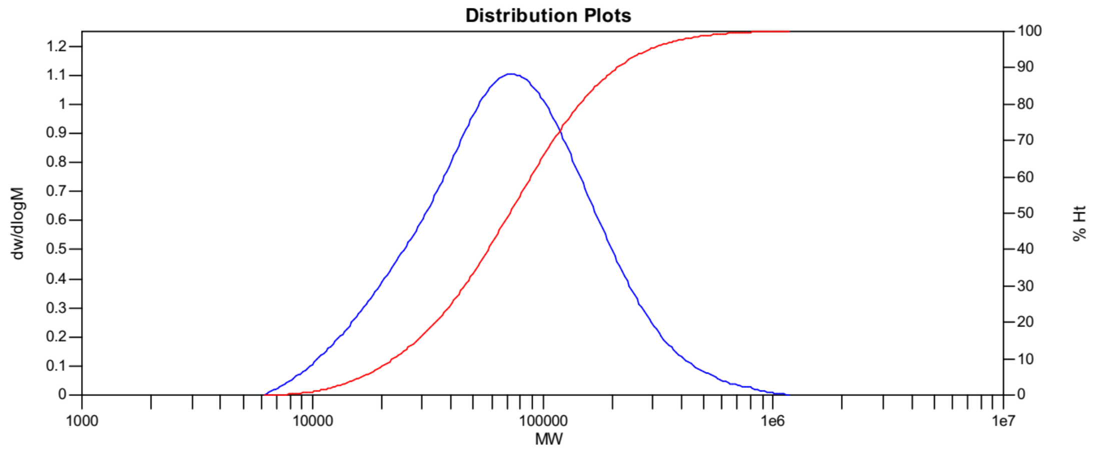

MW Averages

Peak No	Mp	Mn	Mw	Mz	Mz+1	Mv	PD
1	75372	47924	99650	200417	357348	89036	2.07933

Processed Peaks

Peak No	Name	Start RT (mins)	Max RT (mins)	End RT (mins)	Pk Height (mV)	% Height	Area (mV.secs)	% Area
1		12.30	14.18	15.93	-15.3816	0	1319.39	100

Supplementary Figure 162. GPC of the copolymer from Table 2, Entry 18.

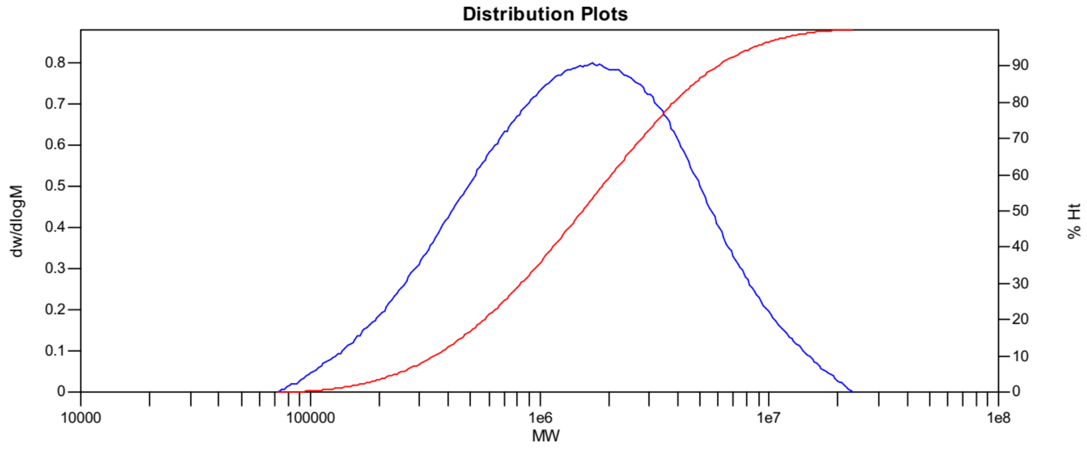

MW Averages

Peak No	Mp	Mn	Mw	Mz	Mz+1	Mv	PD
1	1694285	833665	2549407	5817818	9411797	2184071	3.05807

Processed Peaks

Peak No	Name	Start RT (mins)	Max RT (mins)	End RT (mins)	Pk Height (mV)	% Height	Area (mV.secs)	% Area
1		10.20	12.05	14.20	-8.05773	0	952.661	100

Supplementary Figure 163. GPC of the polymer from Table 2, Entry 19.

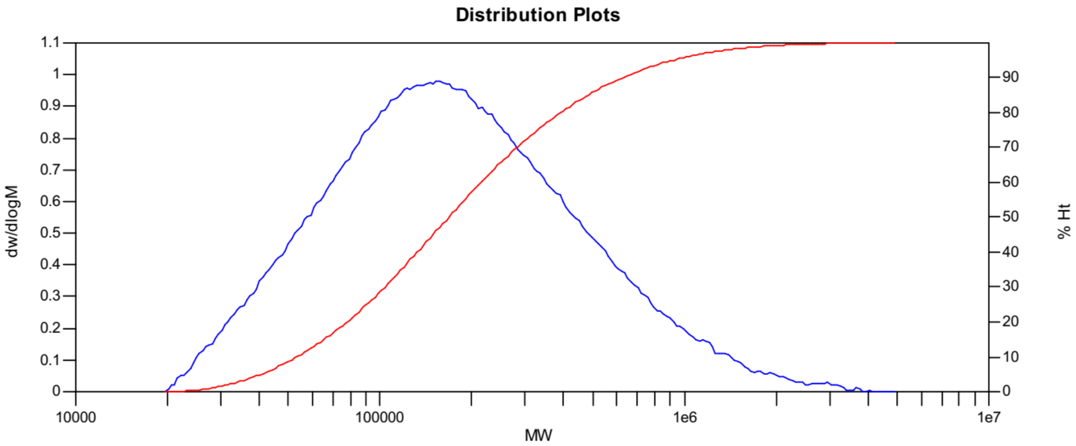

MW Averages

Peak No	Mp	Mn	Mw	Mz	Mz+1	Mv	PD
1	156818	117022	277675	701725	1382106	239118	2.37284

Processed Peaks

Peak No	Name	Start RT (mins)	Max RT (mins)	End RT (mins)	Pk Height (mV)	% Height	Area (mV.secs)	% Area
1		11.32	13.72	15.10	-3.27563	0	315.6	100

Supplementary Figure 164. GPC of the polymer from Table 2, Entry 20.

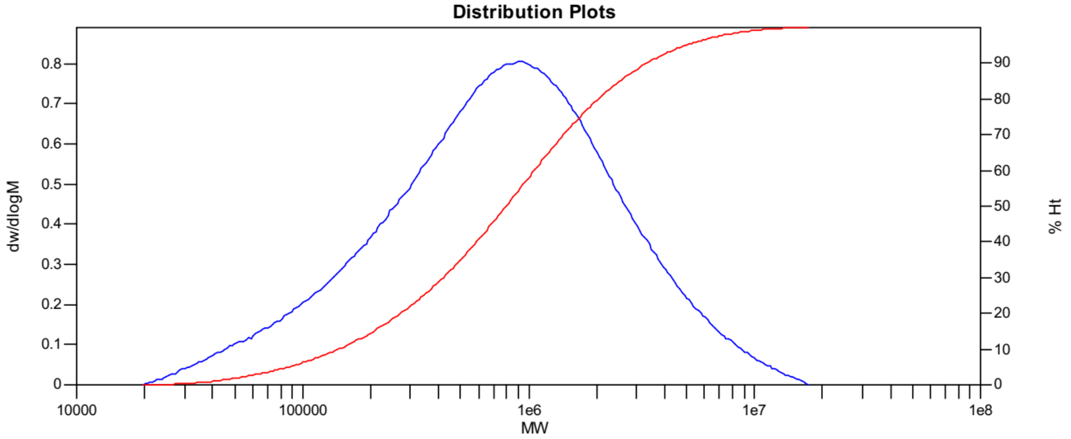

MW Averages

Peak No	Mp	Mn	Mw	Mz	Mz+1	Mv	PD
1	897533	343243	1406729	3769601	6697449	1170456	4.09835

Processed Peaks

Peak No	Name	Start RT (mins)	Max RT (mins)	End RT (mins)	Pk Height (mV)	% Height	Area (mV.secs)	% Area
1		10.42	12.48	15.10	-9.46239	100	1102.65	100

Supplementary Figure 165. GPC of the polymer from Table 2, Entry 21.

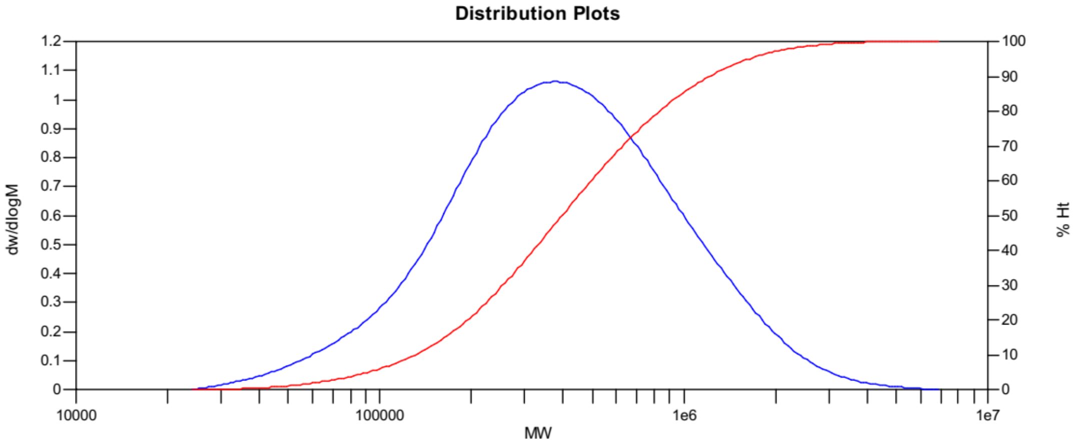

MW Averages

Peak No	Mp	Mn	Mw	Mz	Mz+1	Mv	PD
1	372129	268148	564347	1091493	1825729	507325	2.10461

Processed Peaks

Peak No	Name	Start RT (mins)	Max RT (mins)	End RT (mins)	Pk Height (mV)	% Height	Area (mV.secs)	% Area
1		11.03	13.05	14.93	-48.5695	0	4304.36	100

Supplementary Figure 166. GPC of the copolymer from Table 2, Entry 22.

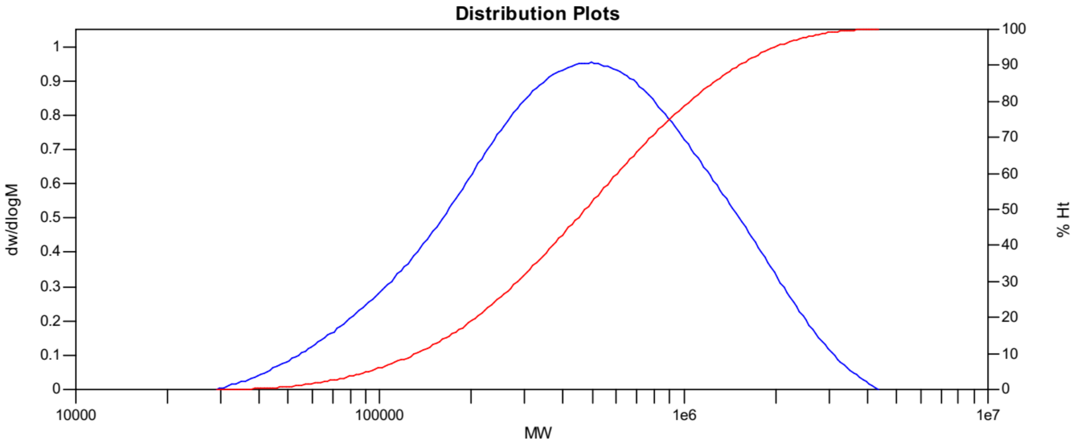

MW Averages

Peak No	Mp	Mn	Mw	Mz	Mz+1	Mv	PD
1	495909	292363	671220	1239319	1808409	601683	2.29584

Processed Peaks

Peak No	Name	Start RT (mins)	Max RT (mins)	End RT (mins)	Pk Height (mV)	% Height	Area (mV.secs)	% Area
1		11.33	12.83	14.78	-27.4957	100	2718.69	100

Supplementary Figure 167. GPC of the copolymer from Table 2, Entry 23.

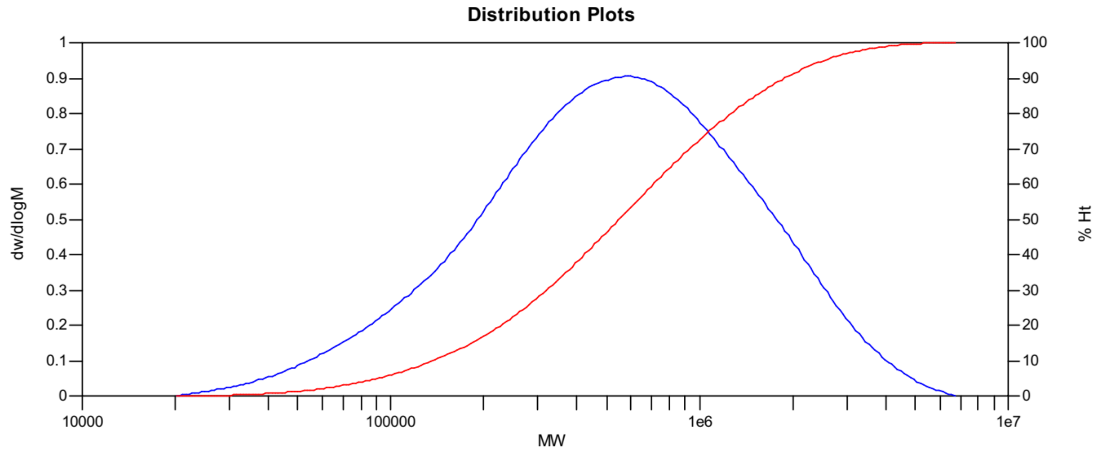

MW Averages

Peak No	Mp	Mn	Mw	Mz	Mz+1	Mv	PD
1	572146	307145	818417	1642032	2524759	720859	2.66459

Processed Peaks

Peak No	Name	Start RT (mins)	Max RT (mins)	End RT (mins)	Pk Height (mV)	% Height	Area (mV.secs)	% Area
1		11.08	12.78	15.08	-46.2242	0	4802.57	100

Supplementary Figure 168. GPC of the copolymer from Table 2, Entry 24.

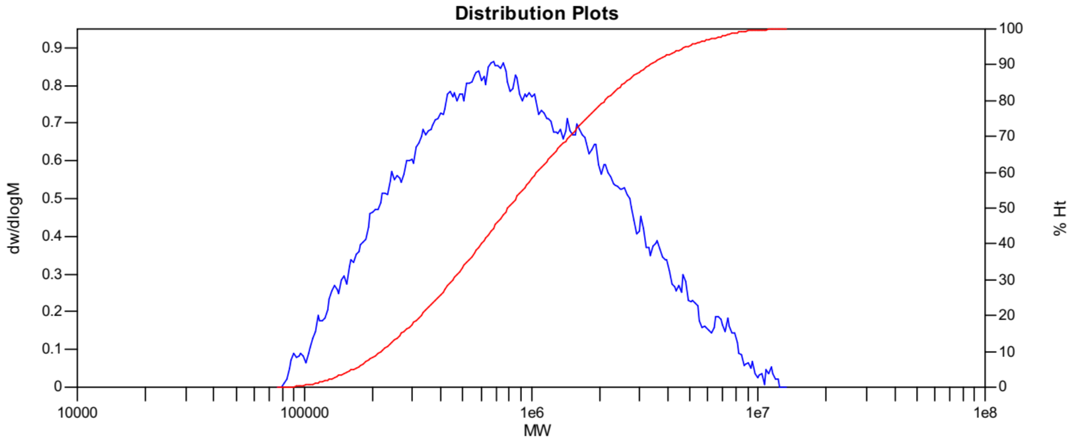

MW Averages

Peak No	Mp	Mn	Mw	Mz	Mz+1	Mv	PD
1	681238	513423	1402315	3311102	5396237	1204034	2.73131

Processed Peaks

Peak No	Name	Start RT (mins)	Max RT (mins)	End RT (mins)	Pk Height (mV)	% Height	Area (mV.secs)	% Area
1		10.53	12.62	14.12	-0.923544	100	100.926	100

Supplementary Figure 169. GPC of the polymer from Table 3, Entry 1.

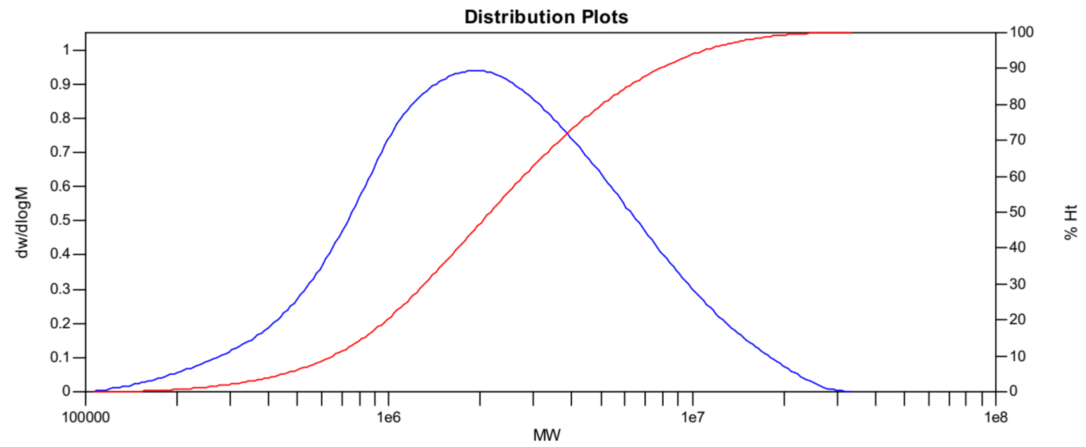

MW Averages

Peak No	Mp	Mn	Mw	Mz	Mz+1	Mv	PD
1	1867443	1388545	3435756	7307561	11820860	3006463	2.47436

Processed Peaks

Peak No	Name	Start RT (mins)	Max RT (mins)	End RT (mins)	Pk Height (mV)	% Height	Area (mV.secs)	% Area
1		9.93	11.98	13.93	-42.0622	100	4243.67	100

Supplementary Figure 170. GPC of the polymer from Table 3, Entry 2.

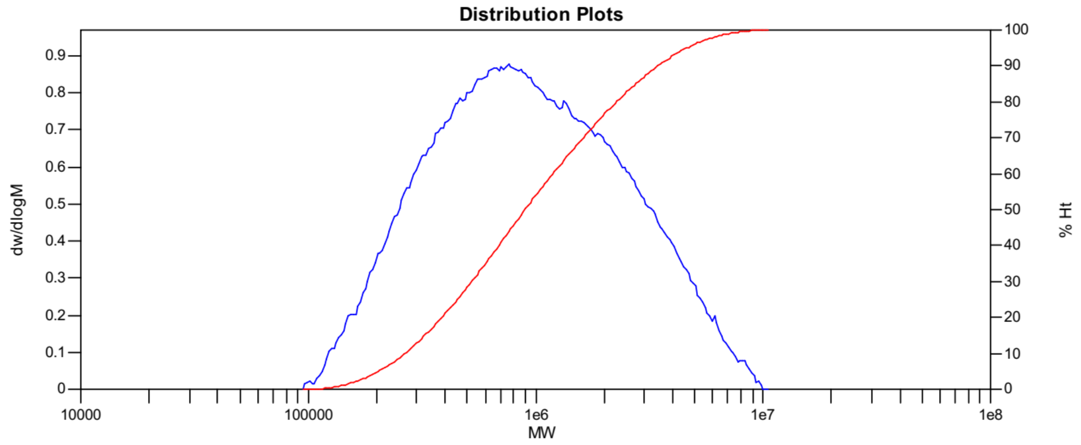

MW Averages

Peak No	Mp	Mn	Mw	Mz	Mz+1	Mv	PD
1	760722	608327	1447369	2948194	4426263	1268640	2.37926

Processed Peaks

Peak No	Name	Start RT (mins)	Max RT (mins)	End RT (mins)	Pk Height (mV)	% Height	Area (mV.secs)	% Area
1		10.80	12.72	14.03	-2.61699	0	283.231	100

Supplementary Figure 171. GPC of the polymer from Table 3, Entry 3.

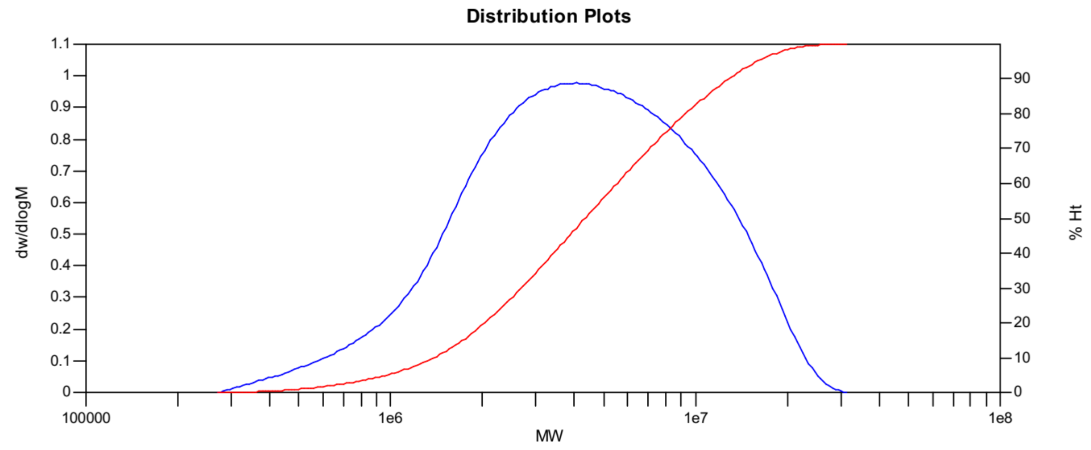

MW Averages

Peak No	Mp	Mn	Mw	Mz	Mz+1	Mv	PD
1	3878063	2897455	5909575	9765173	13200904	5396807	2.03957

Processed Peaks

Peak No	Name	Start RT (mins)	Max RT (mins)	End RT (mins)	Pk Height (mV)	% Height	Area (mV.secs)	% Area
1		9.90	11.45	13.25	-23.0688	100	2288.32	100

Supplementary Figure 172. GPC of the polymer from Table 3, Entry 4.

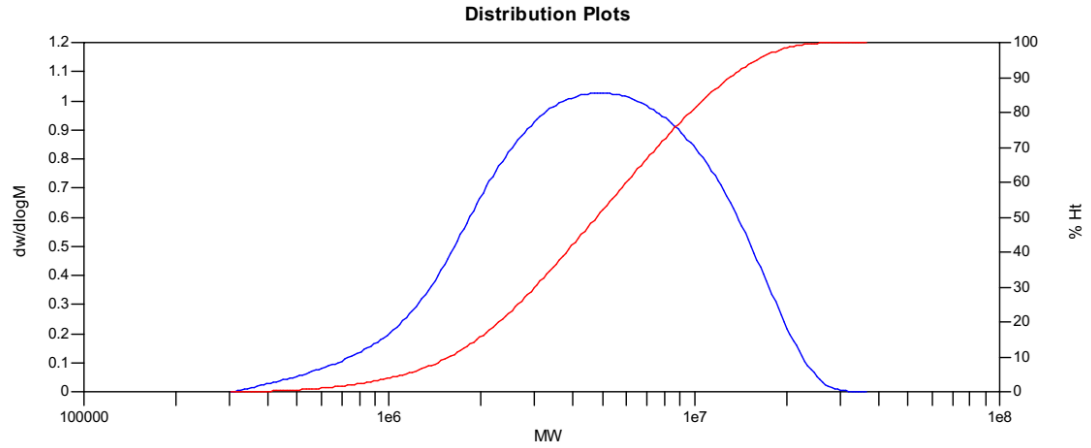

MW Averages

Peak No	Mp	Mn	Mw	Mz	Mz+1	Mv	PD
1	4916234	3250310	6227651	9848229	13111885	5736687	1.91602

Processed Peaks

Peak No	Name	Start RT (mins)	Max RT (mins)	End RT (mins)	Pk Height (mV)	% Height	Area (mV.secs)	% Area
1		9.78	11.32	13.17	-26.8489	100	2548.2	100

Supplementary Figure 173. GPC of the polymer from Table 3, Entry 5.

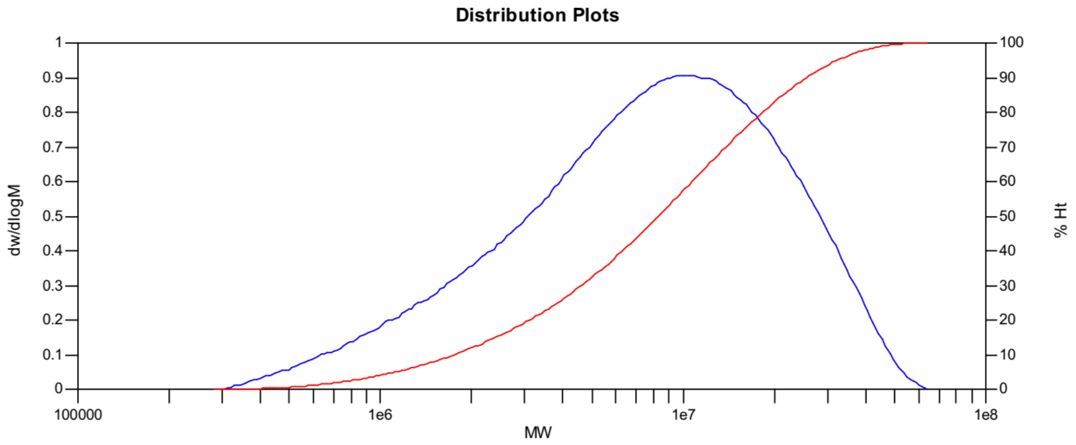

MW Averages

Peak No	Mp	Mn	Mw	Mz	Mz+1	Mv	PD
1	9942636	4241537	11489984	20293320	27680278	10253148	2.70892

Processed Peaks

Peak No	Name	Start RT (mins)	Max RT (mins)	End RT (mins)	Pk Height (mV)	% Height	Area (mV.secs)	% Area
1		9.43	10.83	13.27	-7.20881	0	783.064	100

Supplementary Figure 174. GPC of the polymer from Table 3, Entry 6.

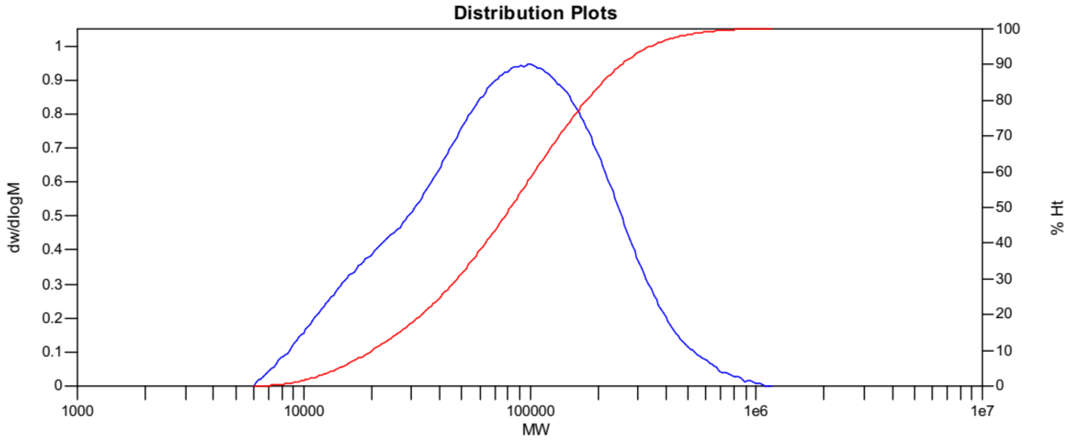

MW Averages

Peak No	Mp	Mn	Mw	Mz	Mz+1	Mv	PD
1	100962	47244	114426	226281	365295	101284	2.42202

Processed Peaks

Peak No	Name	Start RT (mins)	Max RT (mins)	End RT (mins)	Pk Height (mV)	% Height	Area (mV.secs)	% Area
1		12.30	13.98	15.95	-9.46585	0	944.566	100

Supplementary Figure 175. GPC of the polymer from Table 3, Entry 7.

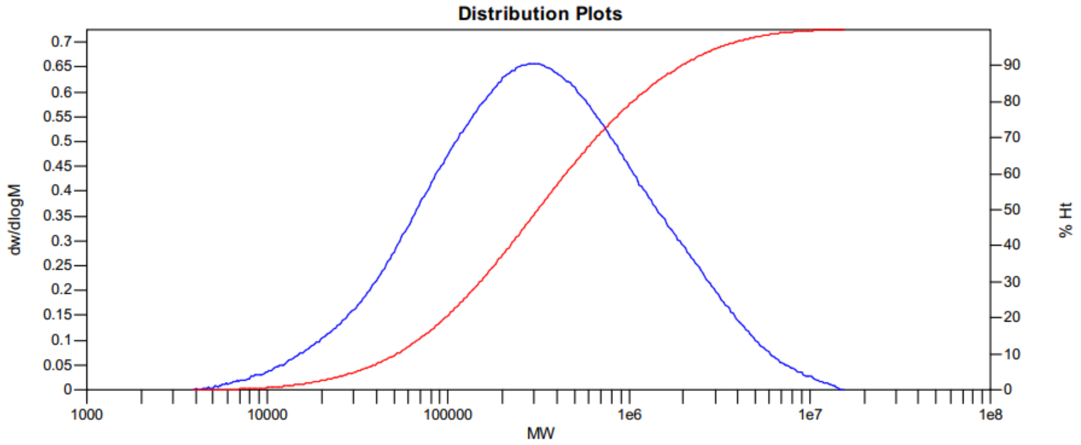

MW Averages

Peak No	Mp	Mn	Mw	Mz	Mz+1	Mv	PD
1	289771	118408	768075	2964000	5791144	590399	6.48668

Processed Peaks

Peak No	Name	Start RT (mins)	Max RT (mins)	End RT (mins)	Pk Height (mV)	% Height	Area (mV.secs)	% Area
1		10.50	13.25	16.28	-12.7776	0	1823.28	100

Supplementary Figure 176. GPC of the polymer from Table 3, Entry 8.

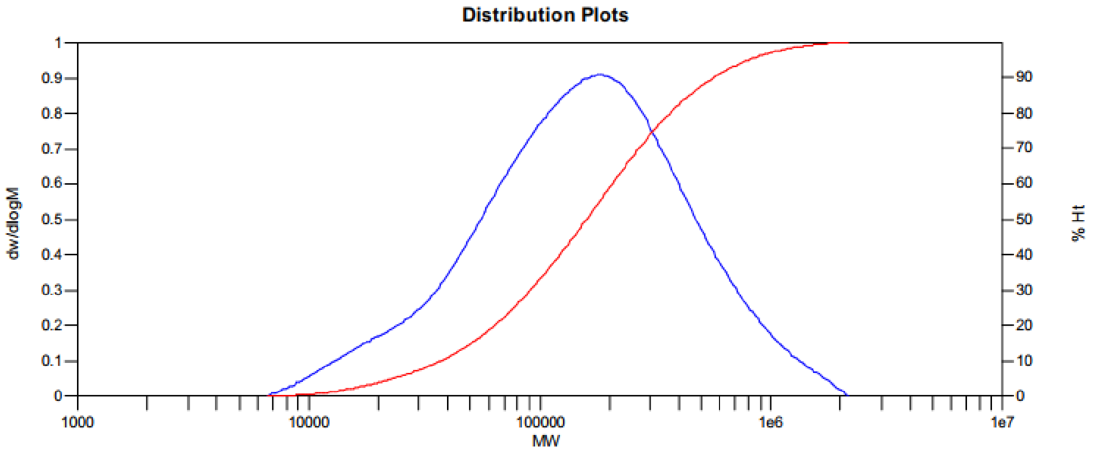

ɅW Averages

Peak No	Mp	Mn	Mw	Mz	Mz+1	Mv	PD
1	186195	83543	242433	532189	871105	210117	2.90189

'rocessed Peaks

Peak No	Name	Start RT (mins)	Max RT (mins)	End RT (mins)	Pk Height (mV)	% Height	Area (mV.secs)	% Area
1		11.88	13.53	15.88	-17.3188	100	1790.44	100

Supplementary Figure 177. GPC of the polymer from Table 3, Entry 9.

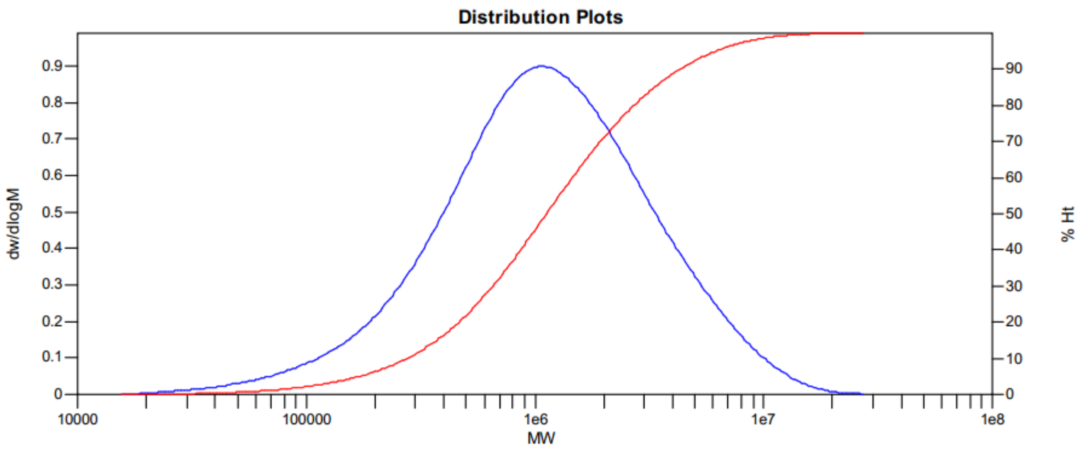

MW Averages

Peak No	Mp	Mn	Mw	Mz	Mz+1	Mv	PD
1	1065423	568848	1861069	4469465	7960122	1592594	3.27165

Processed Peaks

Peak No	Name	Start RT (mins)	Max RT (mins)	End RT (mins)	Pk Height (mV)	% Height	Area (mV.secs)	% Area
1		10.08	12.38	15.27	-41.6771	0	4360.32	100

Supplementary Figure 178. GPC of the polymer from Table 3, Entry 10.

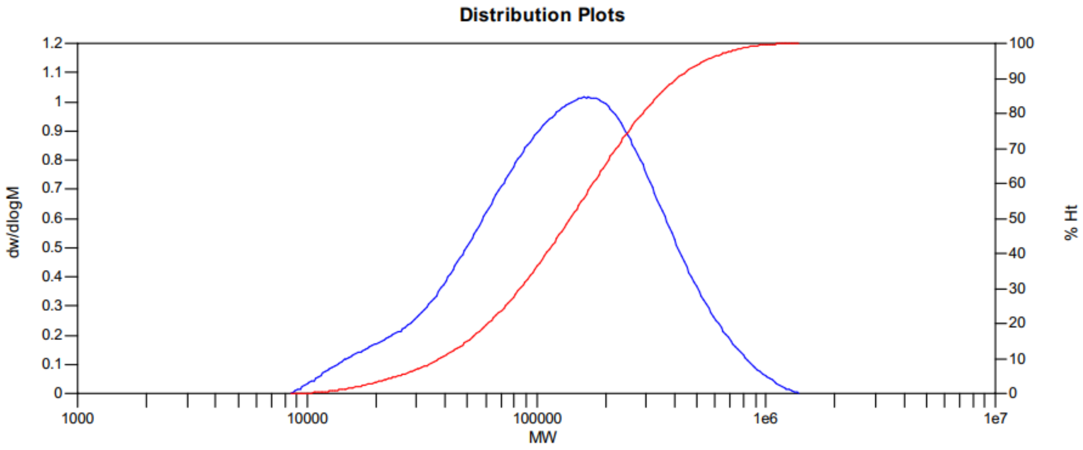

MW Averages

Peak No	Mp	Mn	Mw	Mz	Mz+1	Mv	PD
1	168789	82402	188441	343418	514935	169053	2.28685

Processed Peaks

Peak No	Name	Start RT (mins)	Max RT (mins)	End RT (mins)	Pk Height (mV)	% Height	Area (mV.secs)	% Area
1		12.18	13.62	15.70	-16.0784	0	1485.57	100

Supplementary Figure 179. GPC of the polymer from Table 3, Entry 11.

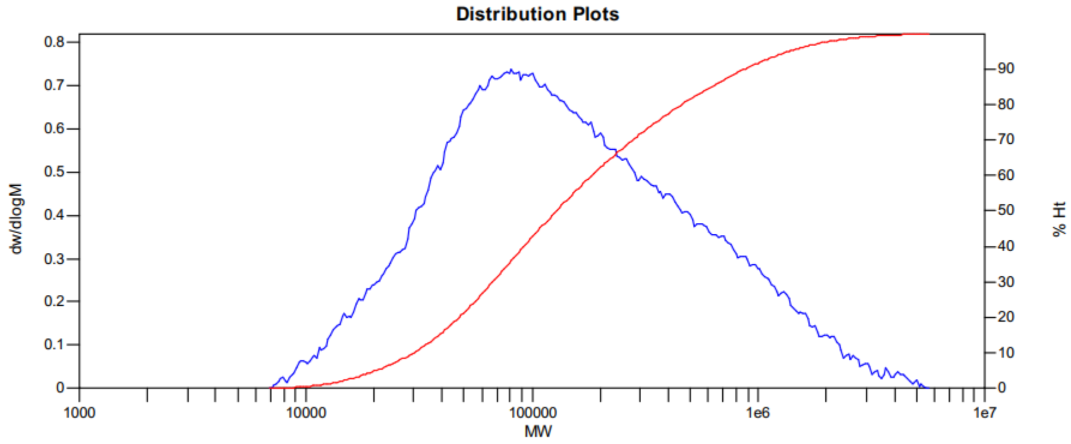

MW Averages

Peak No	Mp	Mn	Mw	Mz	Mz+1	Mv	PD
1	81077	71075	324356	1190323	2233736	252270	4.56357

Processed Peaks

Peak No	Name	Start RT (mins)	Max RT (mins)	End RT (mins)	Pk Height (mV)	% Height	Area (mV.secs)	% Area
1		11.22	14.12	15.85	-1.89005	0	242.61	100

Supplementary Figure 180. GPC of the polymer from Table 3, Entry 12.

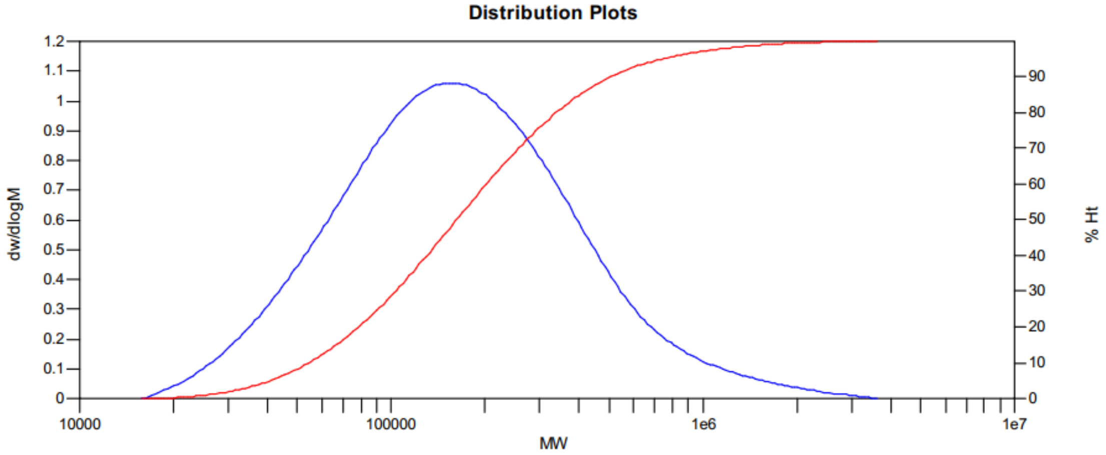

MW Averages

Peak No	Mp	Mn	Mw	Mz	Mz+1	Mv	PD
1	157304	114663	244587	573818	1164828	215703	2.13309

Processed Peaks

Peak No	Name	Start RT (mins)	Max RT (mins)	End RT (mins)	Pk Height (mV)	% Height	Area (mV.secs)	% Area
1		11.10	13.28	14.85	-48.7764	100	4331.6	100

Supplementary Figure 181. GPC of the polymer from Table 3, Entry 13.

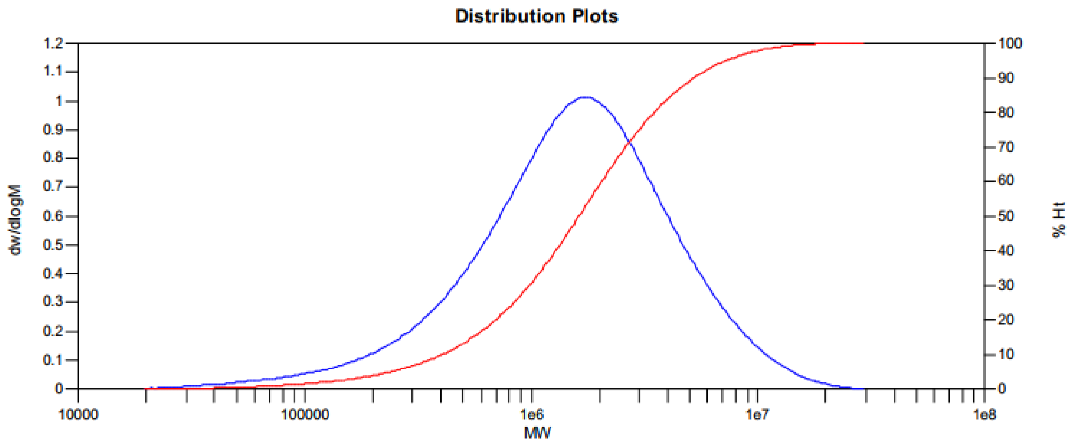

MW Averages

Peak No	Mp	Mn	Mw	Mz	Mz+1	Mv	PD
1	1694285	793790	2394623	5024737	8502978	2101472	3.0167

Processed Peaks

Peak No	Name	Start RT (mins)	Max RT (mins)	End RT (mins)	Pk Height (mV)	% Height	Area (mV.secs)	% Area
1		10.03	12.05	15.10	-42.7627	0	3999.56	100

Supplementary Figure 182. GPC of the polymer from Table 3, Entry 14.

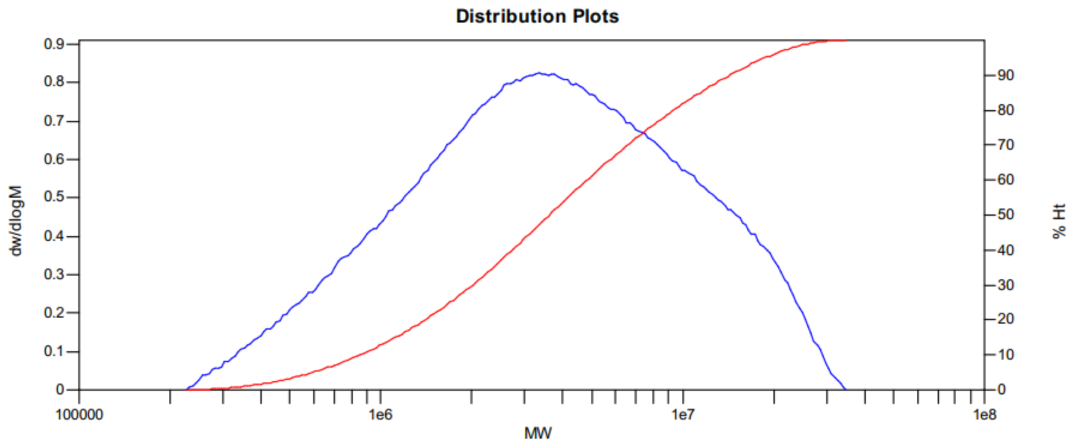

MW Averages

Peak No	Mp	Mn	Mw	Mz	Mz+1	Mv	PD
1	3337251	2084523	5845203	11709943	16608451	5093354	2.8041

Processed Peaks

Peak No	Name	Start RT (mins)	Max RT (mins)	End RT (mins)	Pk Height (mV)	% Height	Area (mV.secs)	% Area
1		9.90	11.58	13.42	-4.66726	0	541.052	100

Supplementary Figure 183. GPC of the polymer from Table 4, Entry 1.

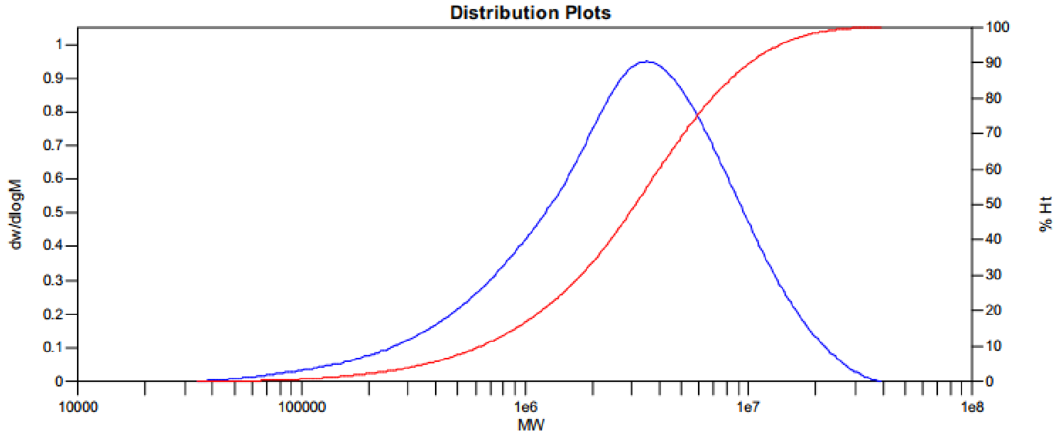

MW Averages

Peak No	Mp	Mn	Mw	Mz	Mz+1	Mv	PD
1	3501670	1332836	4529490	9059297	13971852	3971117	3.39839

Processed Peaks

Peak No	Name	Start RT (mins)	Max RT (mins)	End RT (mins)	Pk Height (mV)	% Height	Area (mV.secs)	% Area
1		9.82	11.55	14.72	-21.3763	0	2157.63	100

Supplementary Figure 184. GPC of the polymer from Table 4, Entry 2.

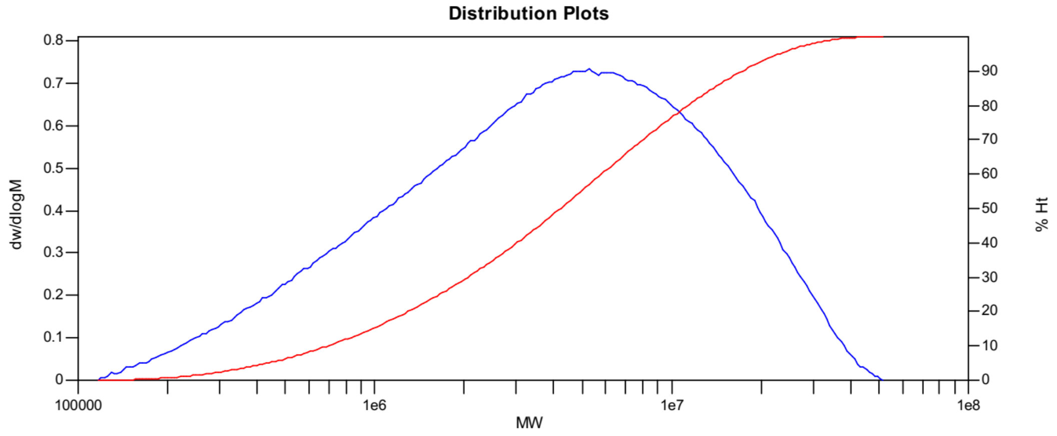

MW Averages

Peak No	Mp	Mn	Mw	Mz	Mz+1	Mv	PD
1	5258880	1791586	7043200	15139352	21980811	6006004	3.93127

Processed Peaks

Peak No	Name	Start RT (mins)	Max RT (mins)	End RT (mins)	Pk Height (mV)	% Height	Area (mV.secs)	% Area
1		9.60	11.27	13.87	-6.31258	0	832.164	100

Supplementary Figure 185. GPC of the polymer from Table 4, Entry 3.

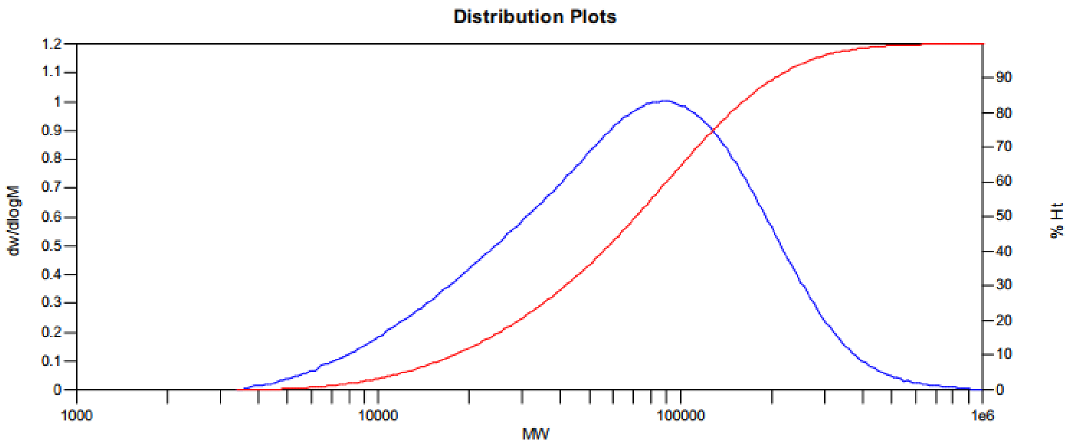

MW Averages

Peak No	Mp	Mn	Mw	Mz	Mz+1	Mv	PD
1	91577	39594	93794	174010	273732	83900	2.36889

Processed Peaks

Peak No	Name	Start RT (mins)	Max RT (mins)	End RT (mins)	Pk Height (mV)	% Height	Area (mV.secs)	% Area
1		12.40	14.03	16.38	-10.8994	0	1028.72	100

Supplementary Figure 186. GPC of the copolymer from Table 4, Entry 4.

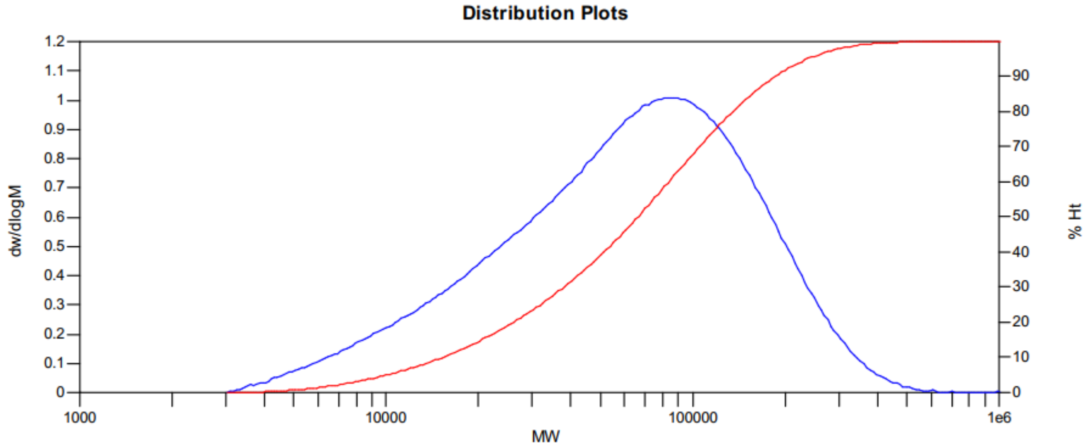

MW Averages

Peak No	Mp	Mn	Mw	Mz	Mz+1	Mv	PD
1	83075	34506	84774	150234	219454	76057	2.45679

Processed Peaks

Peak No	Name	Start RT (mins)	Max RT (mins)	End RT (mins)	Pk Height (mV)	% Height	Area (mV.secs)	% Area
1		12.40	14.10	16.47	-11.4025	0	1068.95	100

Supplementary Figure 187. GPC of the copolymer from Table 4, Entry 5.

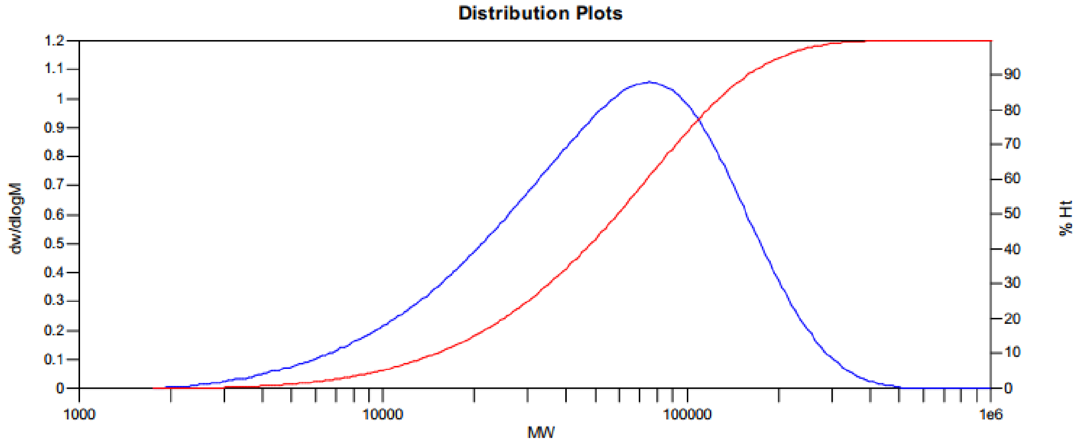

MW Averages

Peak No	Mp	Mn	Mw	Mz	Mz+1	Mv	PD
1	75372	31579	73832	125545	178813	66813	2.33801

Processed Peaks

Peak No	Name	Start RT (mins)	Max RT (mins)	End RT (mins)	Pk Height (mV)	% Height	Area (mV.secs)	% Area
1		12.40	14.17	16.90	-24.5759	0	2202.79	100

Supplementary Figure 188. GPC of the copolymer from Table 4, Entry 6.

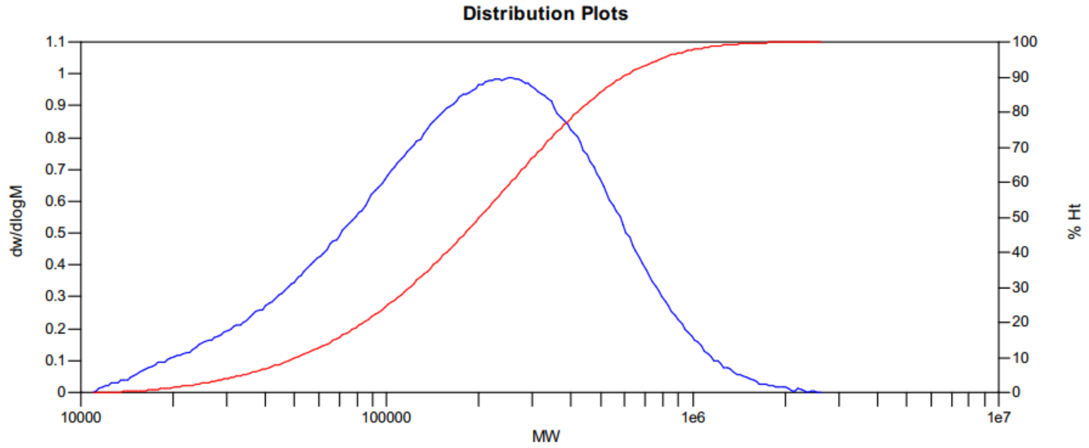

MW Averages

Peak No	Mp	Mn	Mw	Mz	Mz+1	Mv	PD
1	250027	113436	271560	506583	785960	242572	2.39395

Processed Peaks

Peak No	Name	Start RT (mins)	Max RT (mins)	End RT (mins)	Pk Height (mV)	% Height	Area (mV.secs)	% Area
1		11.75	13.35	15.52	-6.24103	0	591.859	100

Supplementary Figure 189. GPC of the copolymer from Table 4, Entry 7.

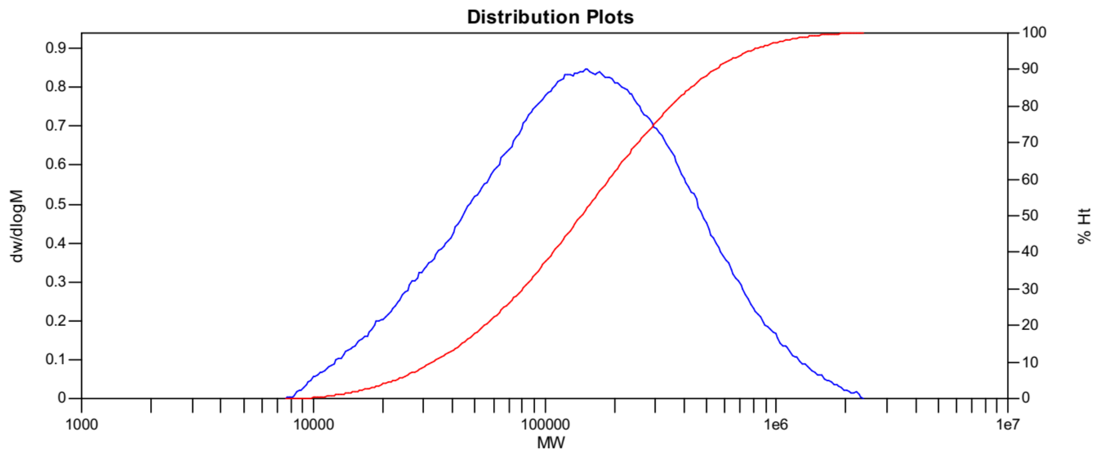

MW Averages

Peak No	Mp	Mn	Mw	Mz	Mz+1	Mv	PD
1	153021	77727	229941	533095	901024	197273	2.95832

Processed Peaks

Peak No	Name	Start RT (mins)	Max RT (mins)	End RT (mins)	Pk Height (mV)	% Height	Area (mV.secs)	% Area
1		11.82	13.68	15.78	-4.78069	0	529.145	100

Supplementary Figure 190. GPC of the copolymer from Table 4, Entry 8.

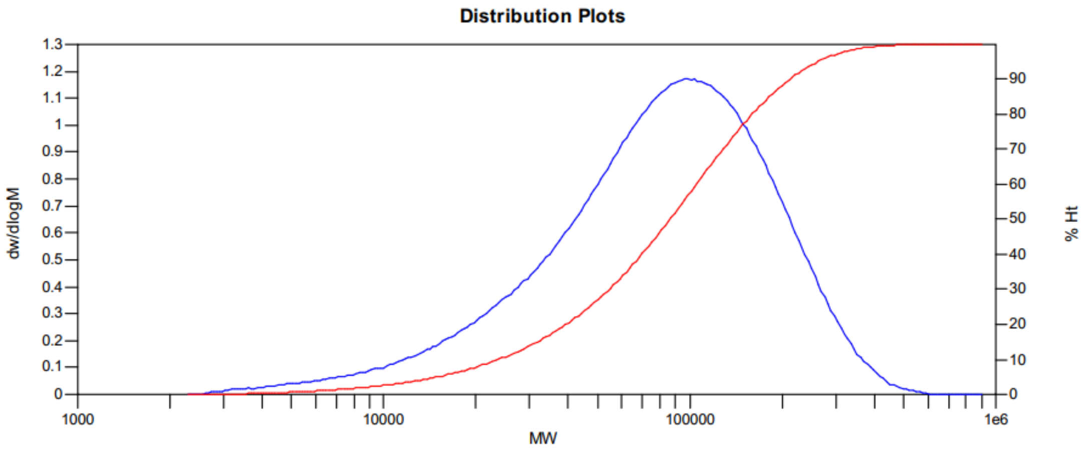

MW Averages

Peak No	Mp	Mn	Mw	Mz	Mz+1	Mv	PD
1	103457	46660	102825	161938	218651	94358	2.20371

Processed Peaks

Peak No	Name	Start RT (mins)	Max RT (mins)	End RT (mins)	Pk Height (mV)	% Height	Area (mV.secs)	% Area
1		12.48	13.95	16.68	-12.0193	0	968.785	100

Supplementary Figure 191. GPC of the copolymer from Table 4, Entry 9.

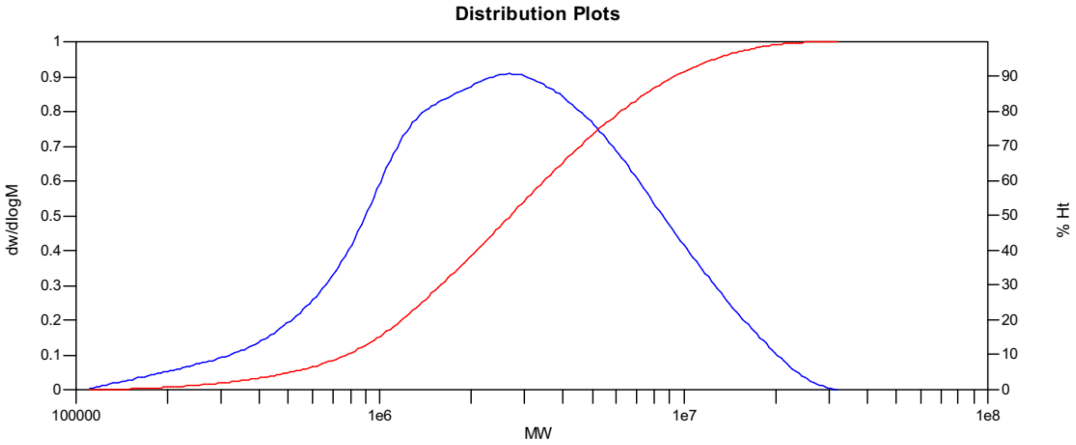

MW Averages

Peak No	Mp	Mn	Mw	Mz	Mz+1	Mv	PD
1	2651875	1617603	4086857	8068207	12233670	3614584	2.52649

Processed Peaks

Peak No	Name	Start RT (mins)	Max RT (mins)	End RT (mins)	Pk Height (mV)	% Height	Area (mV.secs)	% Area
1		9.95	11.75	13.90	-34.3809	100	3620.1	100

Supplementary Figure 192. GPC of the polymer from Table 4, Entry 10.

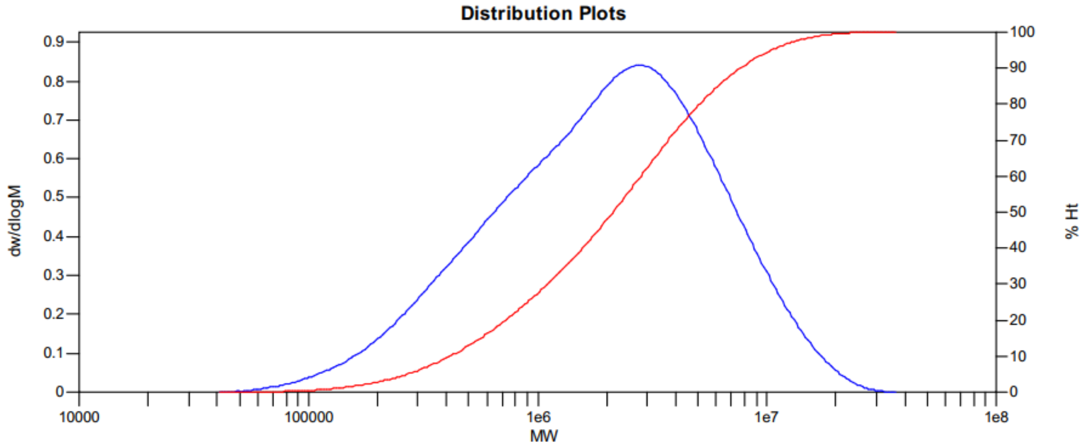

MW Averages

Peak No	Mp	Mn	Mw	Mz	Mz+1	Mv	PD
1	2751934	997316	3299784	7095926	11171289	2847424	3.30866

Processed Peaks

Peak No	Name	Start RT (mins)	Max RT (mins)	End RT (mins)	Pk Height (mV)	% Height	Area (mV.secs)	% Area
1		9.87	11.72	14.58	-24.3394	0	2758.13	100

Supplementary Figure 193. GPC of the polymer from Table 4, Entry 11.

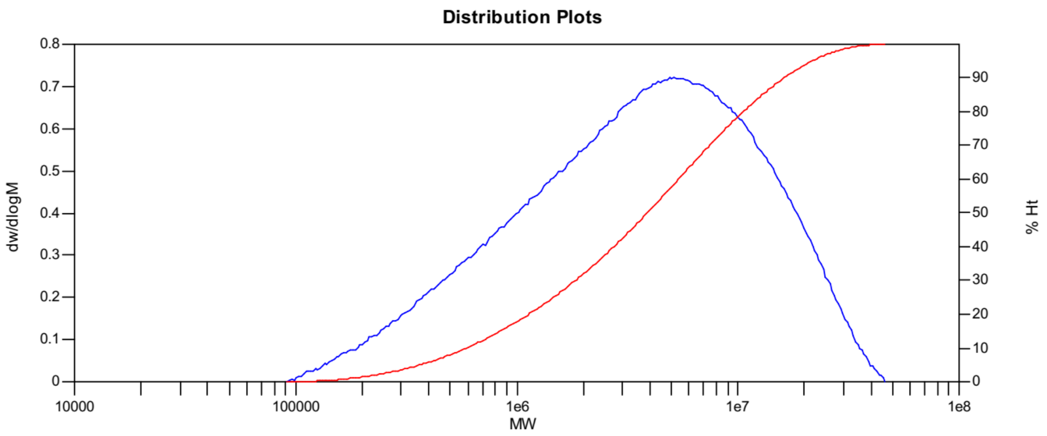

MW Averages

Peak No	Mp	Mn	Mw	Mz	Mz+1	Mv	PD
1	4896108	1540563	6519457	14151281	20500242	5536674	4.23187

Processed Peaks

Peak No	Name	Start RT (mins)	Max RT (mins)	End RT (mins)	Pk Height (mV)	% Height	Area (mV.secs)	% Area
1		9.68	11.32	14.03	-4.88666	0	654.227	100

Supplementary Figure 194. GPC of the polymer from Table 4, Entry 12.

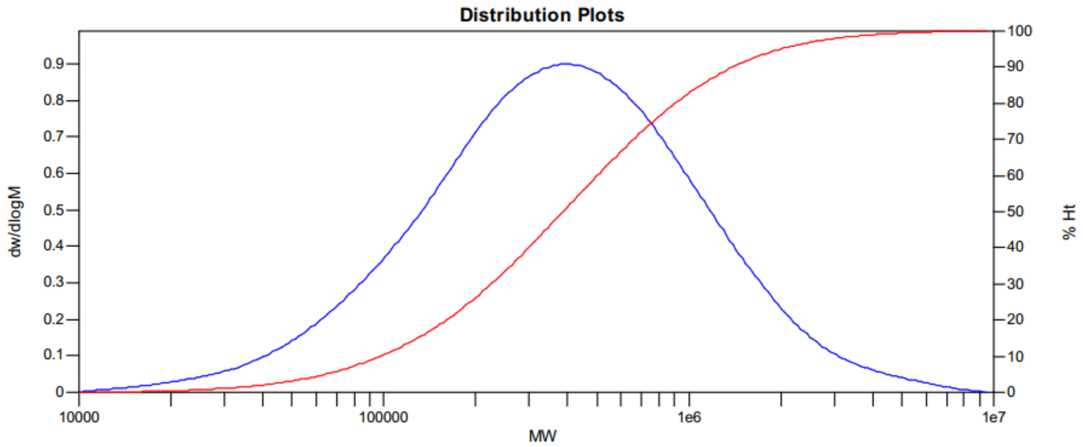

MW Averages

Peak No	Mp	Mn	Mw	Mz	Mz+1	Mv	PD
1	398960	209780	625080	1570711	3061965	535041	2.97969

Processed Peaks

Peak No	Name	Start RT (mins)	Max RT (mins)	End RT (mins)	Pk Height (mV)	% Height	Area (mV.secs)	% Area
1		10.85	13.03	15.58	-32.9206	0	3424.55	100

Supplementary Figure 195. GPC of the copolymer from Table 4, Entry13.

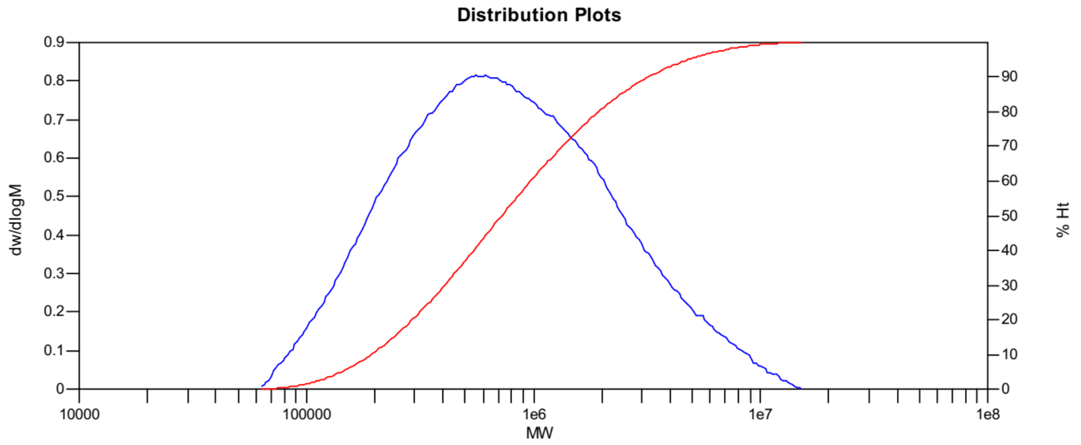

MW Averages

Peak No	Mp	Mn	Mw	Mz	Mz+1	Mv	PD
1	558295	456229	1338059	3488898	6034612	1128416	2.93287

Processed Peaks

Peak No	Name	Start RT (mins)	Max RT (mins)	End RT (mins)	Pk Height (mV)	% Height	Area (mV.secs)	% Area
1		10.50	12.83	14.28	-6.13822	100	709.333	100

Supplementary Figure 196. GPC of the copolymer from Table 4, Entry 14.

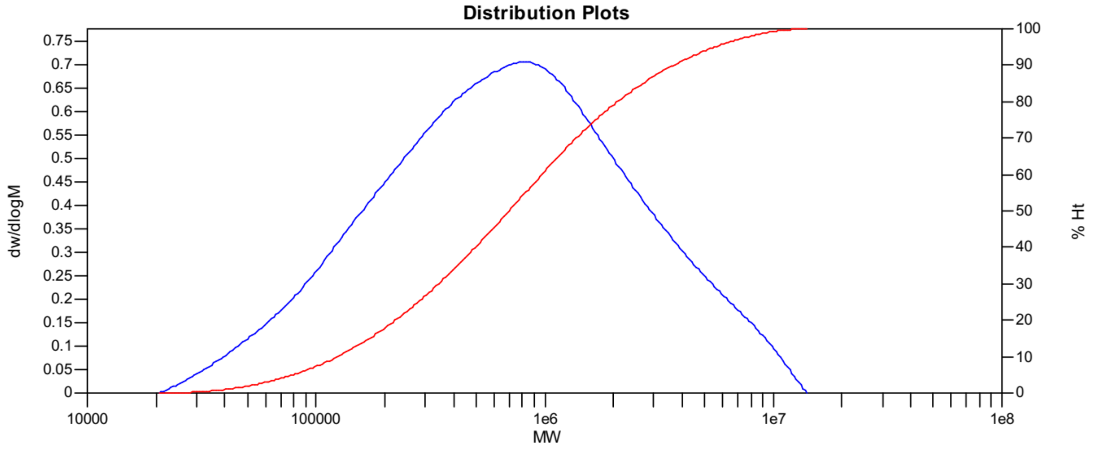

MW Averages

Peak No	Mp	Mn	Mw	Mz	Mz+1	Mv	PD
1	806587	311891	1427299	3998969	6467491	1168585	4.57628

Processed Peaks

Peak No	Name	Start RT (mins)	Max RT (mins)	End RT (mins)	Pk Height (mV)	% Height	Area (mV.secs)	% Area
1		10.12	12.15	14.67	-38.2075	100	5148.2	100

Supplementary Figure 197. GPC of the polymer from Supplementary Table 5, Entry 1.

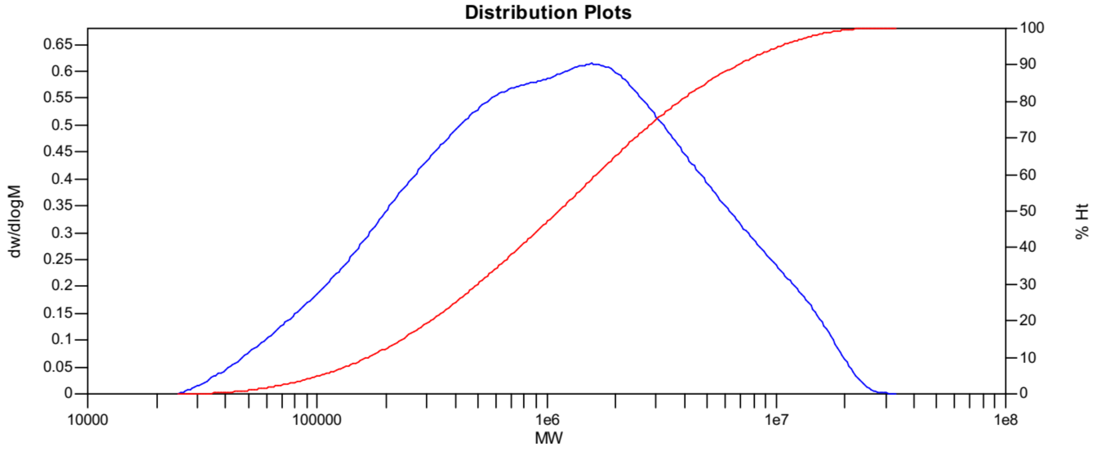

MW Averages

Peak No	Mp	Mn	Mw	Mz	Mz+1	Mv	PD
1	1567346	428892	2545668	7557970	12067711	2037937	5.93545

Processed Peaks

Peak No	Name	Start RT (mins)	Max RT (mins)	End RT (mins)	Pk Height (mV)	% Height	Area (mV.secs)	% Area
1		9.88	12.07	14.92	-27.7412	0	4294.95	100

Supplementary Figure 198. GPC of the polymer from Supplementary Table 5, Entry 2.

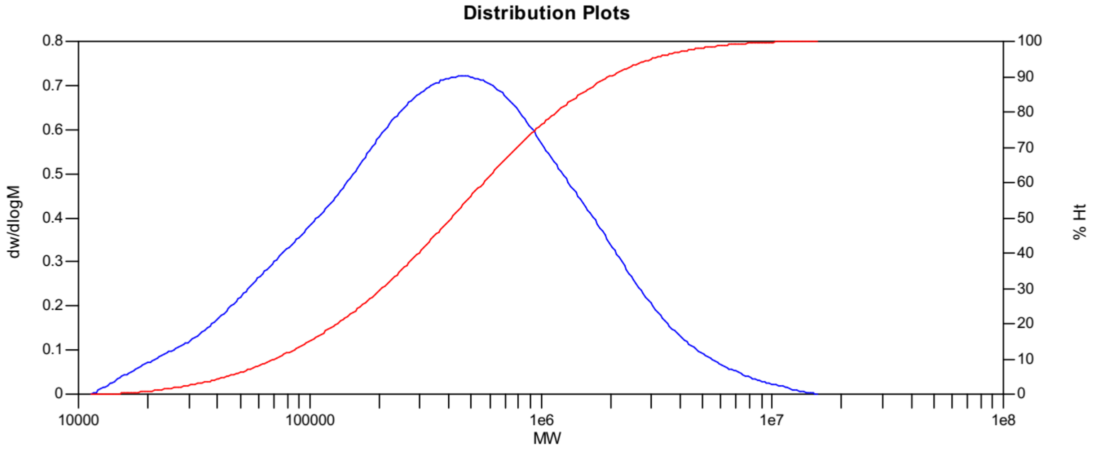

MW Averages

Peak No	Mp	Mn	Mw	Mz	Mz+1	Mv	PD
1	449715	173478	825505	2708211	5434547	668217	4.75856

Processed Peaks

Peak No	Name	Start RT (mins)	Max RT (mins)	End RT (mins)	Pk Height (mV)	% Height	Area (mV.secs)	% Area
1		10.42	12.90	15.45	-20.3793	100	2659.76	100

Supplementary Figure 199. GPC of the polymer from Supplementary Table 5, Entry 3.

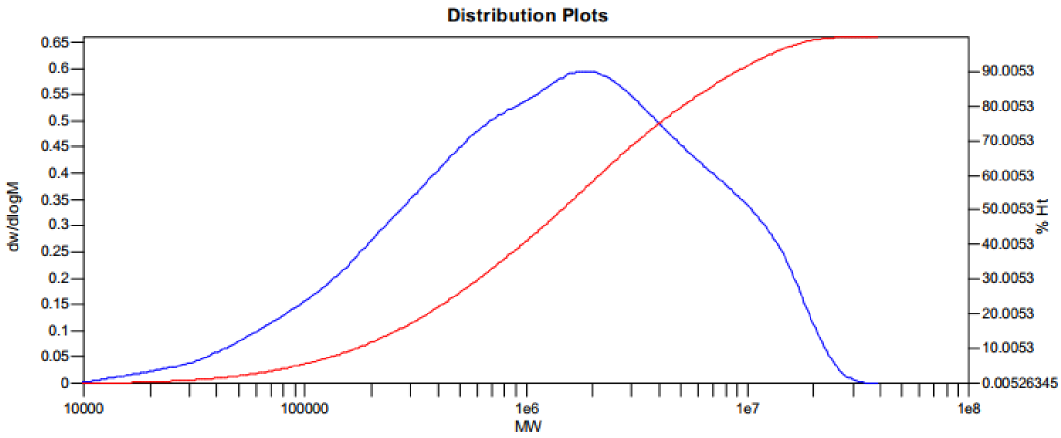

MW Averages

Peak No	Mp	Mn	Mw	Mz	Mz+1	Mv	PD
1	1778789	388605	3216305	8945577	13499671	2560326	8.27654

Processed Peaks

Peak No	Name	Start RT (mins)	Max RT (mins)	End RT (mins)	Pk Height (mV)	% Height	Area (mV.secs)	% Area
1		9.82	12.02	15.63	-24.9869	0	3976.77	100

Supplementary Figure 200. GPC of the polymer from Supplementary Table 5, Entry 4.

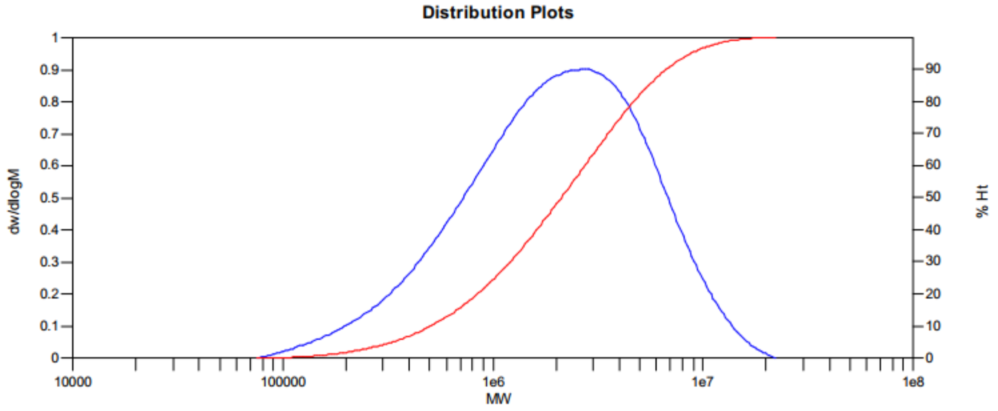

MW Averages

Peak No	Mp	Mn	Mw	Mz	Mz+1	Mv	PD
1	2622113	1152108	3002741	5627959	8291533	2666154	2.6063

Processed Peaks

Peak No	Name	Start RT (mins)	Max RT (mins)	End RT (mins)	Pk Height (mV)	% Height	Area (mV.secs)	% Area
1		10.23	11.75	14.17	-20.1038	0	2125.47	100

Supplementary Figure 201. GPC of the polymer from Fig. 6a (0h).

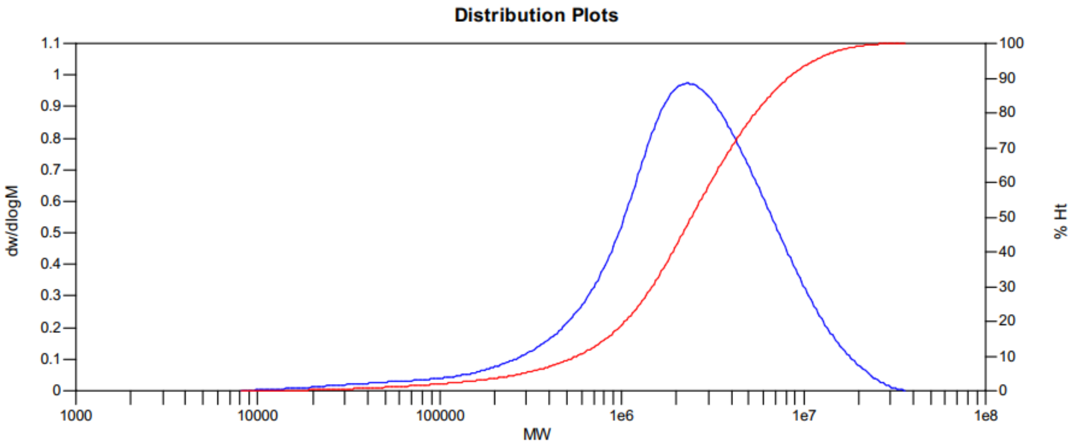

MW Averages

Peak No	Mp	Mn	Mw	Mz	Mz+1	Mv	PD
1	2267675	795242	3672713	7710883	12502715	3204367	4.61836

Processed Peaks

Peak No	Name	Start RT (mins)	Max RT (mins)	End RT (mins)	Pk Height (mV)	% Height	Area (mV.secs)	% Area
1		9.87	11.85	15.73	-50.083	0	4888.06	100

Supplementary Figure 202. GPC of the polymer from Fig. 6a (48h).

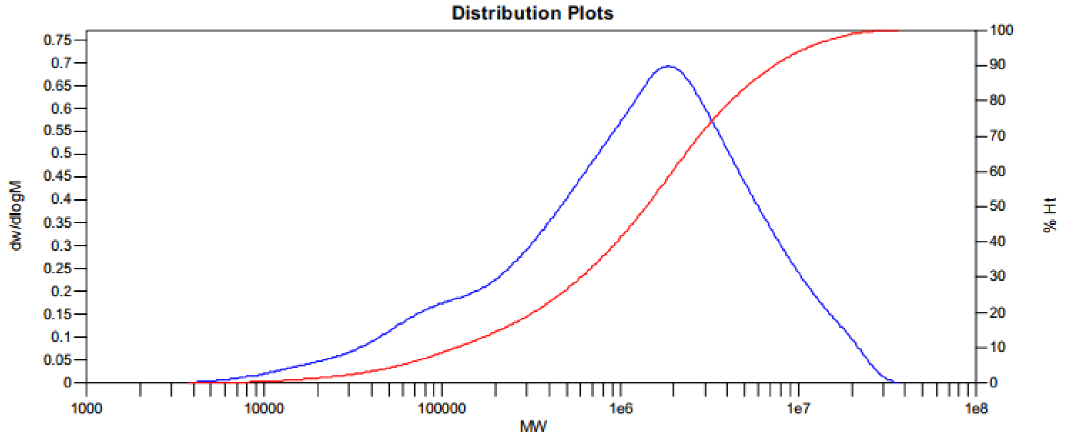

MW Averages

Peak No	Mp	Mn	Mw	Mz	Mz+1	Mv	PD
1	1822585	253228	2858711	8532992	14177144	2268595	11.2891

Processed Peaks

Peak No	Name	Start RT (mins)	Max RT (mins)	End RT (mins)	Pk Height (mV)	% Height	Area (mV.secs)	% Area
1		9.87	12.00	16.30	-33.7552	0	4618.51	100

Supplementary Figure 203. GPC of the polymer from Fig. 6a (96h).

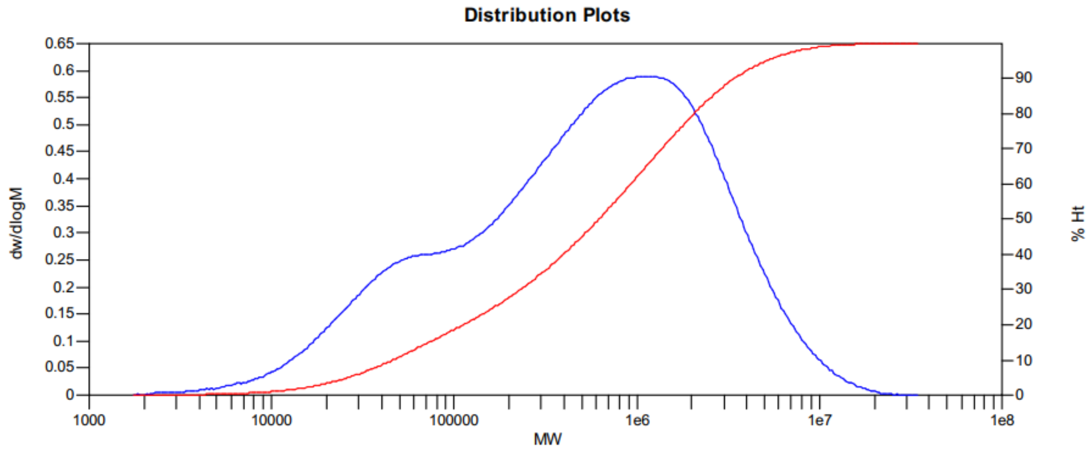

MW Averages

Peak No	Mp	Mn	Mw	Mz	Mz+1	Mv	PD
1	1118867	113839	1334336	4381680	8224375	1037935	11.7213

Processed Peaks

Peak No	Name	Start RT (mins)	Max RT (mins)	End RT (mins)	Pk Height (mV)	% Height	Area (mV.secs)	% Area
1		9.90	12.33	16.90	-29.9164	0	4774.08	100

Supplementary Figure 204. GPC of the polymer from Fig. 6a (192h).

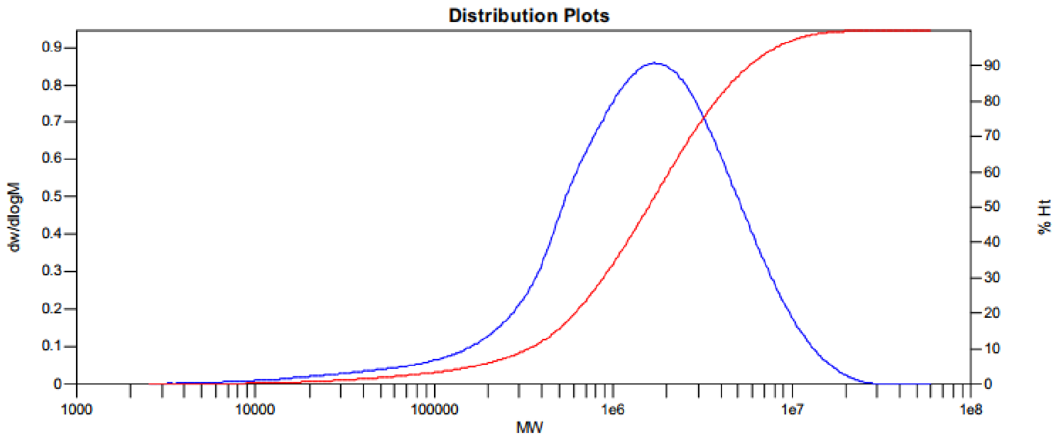

MW Averages

Peak No	Mp	Mn	Mw	Mz	Mz+1	Mv	PD
1	1653530	432213	2498672	5635481	9449812	2145357	5.78111

Processed Peaks

Peak No	Name	Start RT (mins)	Max RT (mins)	End RT (mins)	Pk Height (mV)	% Height	Area (mV.secs)	% Area
1		9.48	12.07	16.60	-63.938	0	7053.03	100

Supplementary Figure 205. GPC of the polymer from Fig. 6b (0h).

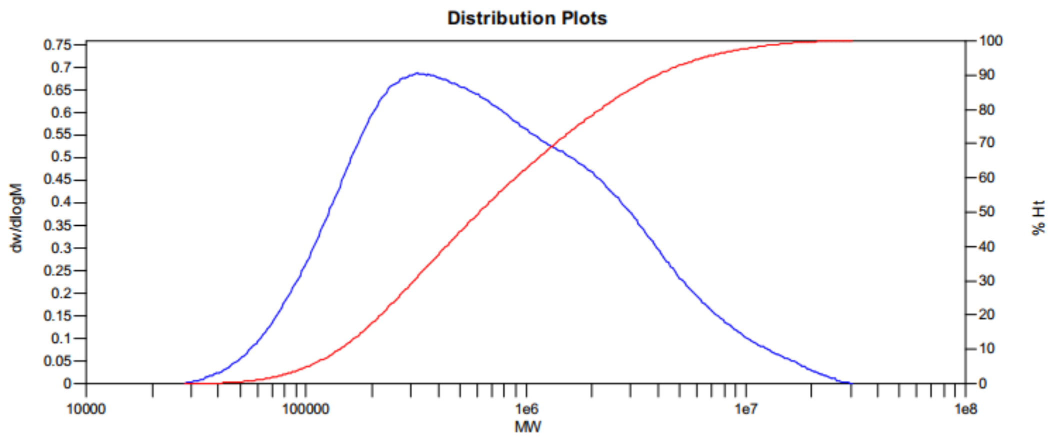

MW Averages

Peak No	Mp	Mn	Mw	Mz	Mz+1	Mv	PD
1	319728	344209	1594501	6044999	11563337	1235129	4.63236

Processed Peaks

Peak No	Name	Start RT (mins)	Max RT (mins)	End RT (mins)	Pk Height (mV)	% Height	Area (mV.secs)	% Area
1		10.00	13.18	14.85	-14.9417	0	2033.43	100

Supplementary Figure 206. GPC of the polymer from Fig. 6b (48h).

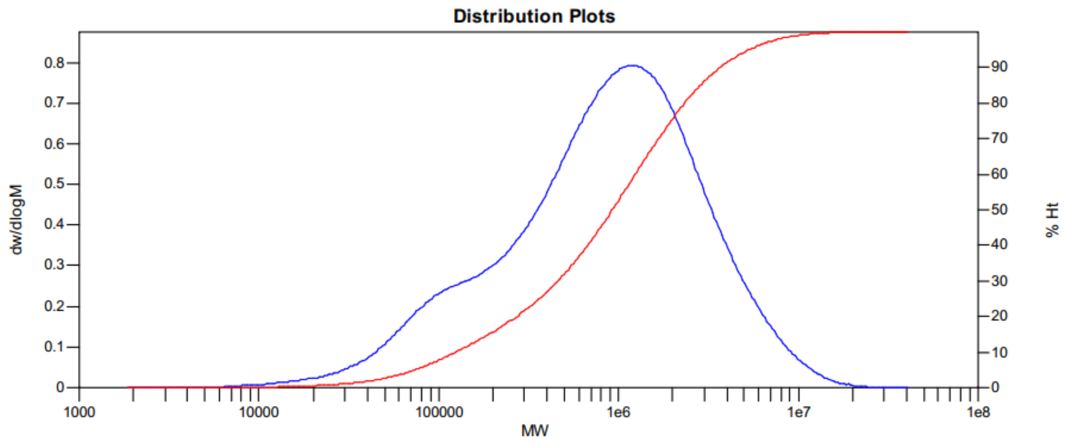

MW Averages

Peak No	Mp	Mn	Mw	Mz	Mz+1	Mv	PD
1	1204046	294818	1560599	4107256	7640993	1298192	5.29343

Processed Peaks

Peak No	Name	Start RT (mins)	Max RT (mins)	End RT (mins)	Pk Height (mV)	% Height	Area (mV.secs)	% Area
1		9.78	12.28	16.85	-34.4047	0	4089.33	100

Supplementary Figure 207. GPC of the polymer from Fig. 6b (96h).

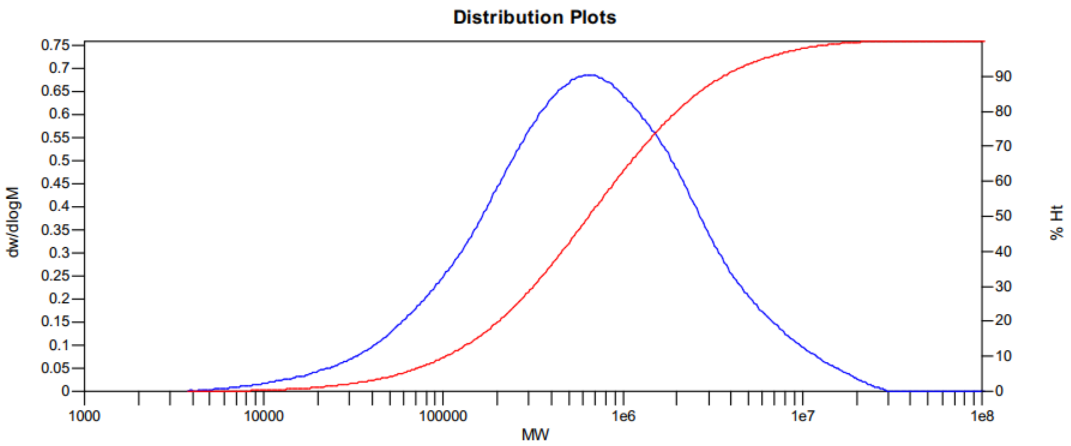

MW Averages

Peak No	Mp	Mn	Mw	Mz	Mz+1	Mv	PD
1	652363	212337	1528420	6145438	14815890	1178401	7.19809

Processed Peaks

Peak No	Name	Start RT (mins)	Max RT (mins)	End RT (mins)	Pk Height (mV)	% Height	Area (mV.secs)	% Area
1		9.05	12.70	16.30	-28.1949	0	3845.23	100

Supplementary Figure 208. GPC of the polymer from Fig. 6b (192h).

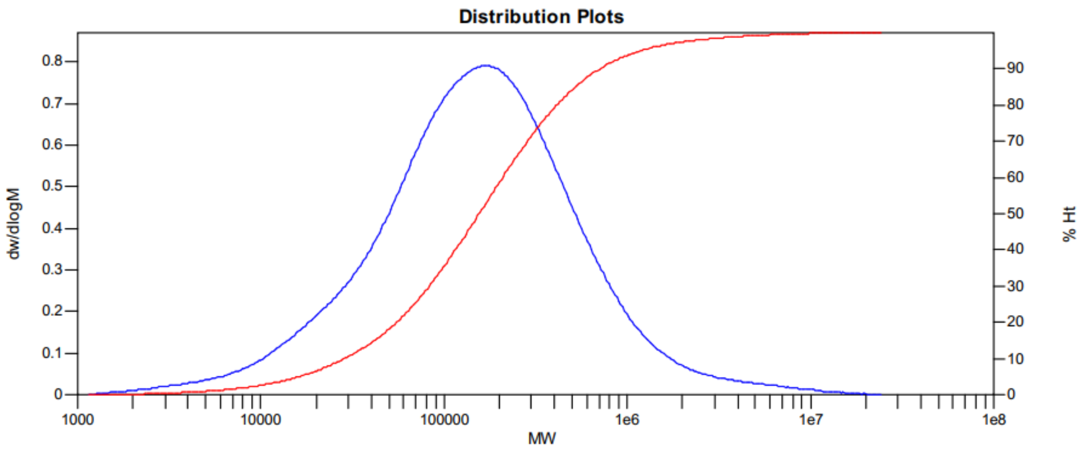

MW Averages

Peak No	Mp	Mn	Mw	Mz	Mz+1	Mv	PD
1	168789	56234	361234	2613034	8093726	267641	6.42376

Processed Peaks

Peak No	Name	Start RT (mins)	Max RT (mins)	End RT (mins)	Pk Height (mV)	% Height	Area (mV.secs)	% Area
1		10.17	13.62	17.23	-48.9477	0	5807.02	100

Supplementary Figure 209. GPC of the polymer from Fig. 6c (0h).

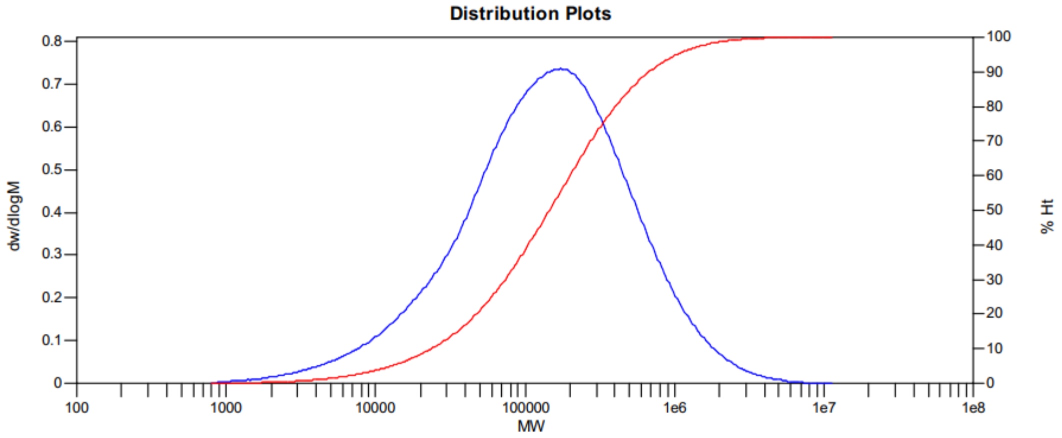

MW Averages

Peak No	Mp	Mn	Mw	Mz	Mz+1	Mv	PD
1	172981	45199	284635	1005756	2315196	227470	6.29737

Processed Peaks

Peak No	Name	Start RT (mins)	Max RT (mins)	End RT (mins)	Pk Height (mV)	% Height	Area (mV.secs)	% Area
1		10.72	13.60	17.53	-65.3336	0	8329.07	100

Supplementary Figure 210. GPC of the polymer from Fig. 6c (48h).

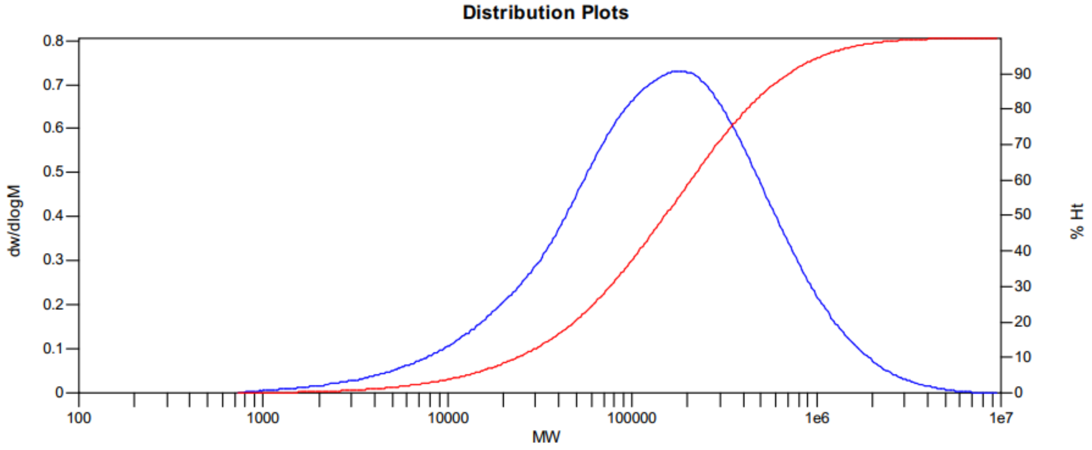

MW Averages

Peak No	Mp	Mn	Mw	Mz	Mz+1	Mv	PD
1	181681	44259	292996	1006097	2261789	234728	6.62003

Processed Peaks

Peak No	Name	Start RT (mins)	Max RT (mins)	End RT (mins)	Pk Height (mV)	% Height	Area (mV.secs)	% Area
1		10.85	13.57	17.62	-45.6182	100	5853.14	100

Supplementary Figure 211. GPC of the polymer from Fig. 6c (96h).

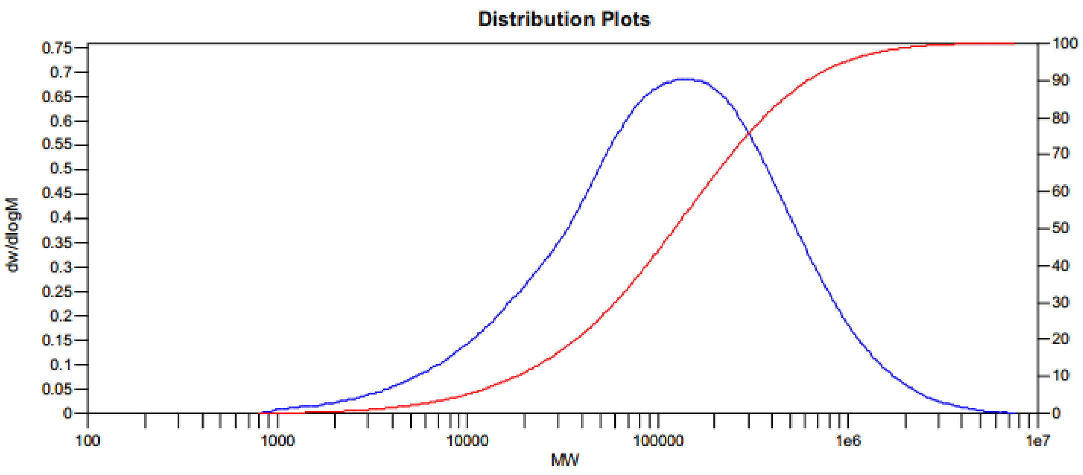

MW Averages

Peak No	Mp	Mn	Mw	Mz	Mz+1	Mv	PD
1	149317	35446	253137	935155	2106094	199310	7.14148

Processed Peaks

Peak No	Name	Start RT (mins)	Max RT (mins)	End RT (mins)	Pk Height (mV)	% Height	Area (mV.secs)	% Area
1		11.02	13.70	17.53	-30.1192	0	4122.72	100

Supplementary Figure 212. GPC of the polymer from Fig. 6c (192h).

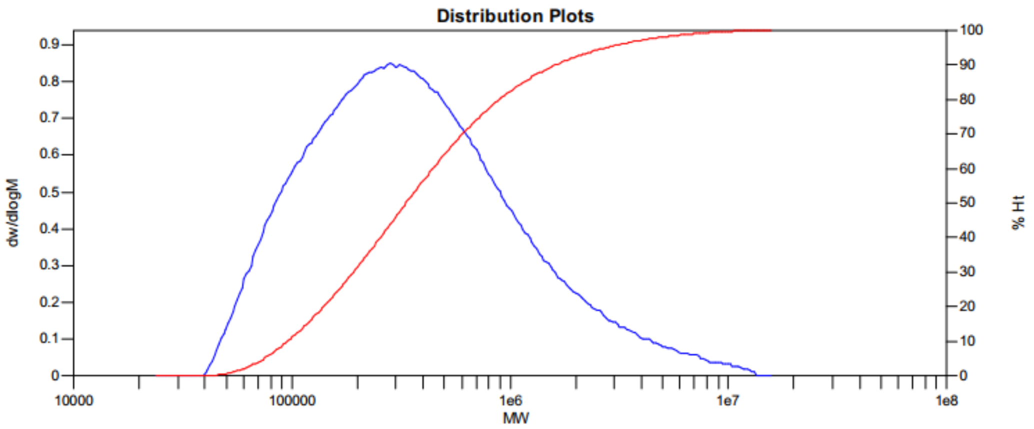

MW Averages

Peak No	Mp	Mn	Mw	Mz	Mz+1	Mv	PD
1	282732	224162	725429	2811609	5954136	578044	3.23618

Processed Peaks

Peak No	Name	Start RT (mins)	Max RT (mins)	End RT (mins)	Pk Height (mV)	% Height	Area (mV.secs)	% Area
1		10.48	13.27	14.97	-5.83513	0	641.108	100

Supplementary Figure 213. GPC of the polymer from Fig. 6d (48h)

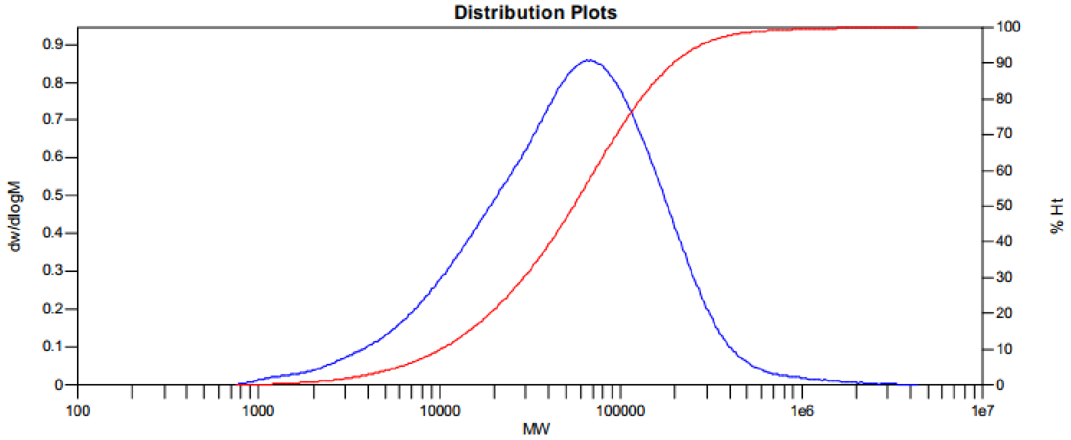

MW Averages

Peak No	Mp	Mn	Mw	Mz	Mz+1	Mv	PD
1	68393	21032	89423	333311	1171406	73824	4.25176

Processed Peaks

Peak No	Name	Start RT (mins)	Max RT (mins)	End RT (mins)	Pk Height (mV)	% Height	Area (mV.secs)	% Area
1		11.40	14.23	17.58	-29.277	0	3239.49	100

Supplementary Figure 214. GPC of the polymer from Fig. 6d (196h).

7 Supplementary References.

(1) Gates, D. P., Svejda, S. A., Oñate, E., Killian, C. M., Johnson, L. K.; White, P. S. & Brookhart, M. Synthesis of Branched Polyethylene Using (α-Diimine) nickel (II) Catalysts: Influence of Temperature, Ethylene Pressure, and Ligand Structure on Polymer Properties. *Macromolecules* **33**, 2320-2334 (2000**)**.

(2) Rhinehart, J. L., Brown, L. A. & Long, B. K. A Robust Ni(II) α-Diimine Catalyst for High Temperature Ethylene Polymerization. *J. Am. Chem. Soc.* **135**, 16316-16319 (2013).

(3) Chen, M. & Chen, C. A Versatile Ligand Platform for Palladium- and Nickel-Catalyzed Ethylene Copolymerization with Polar Monomers. *Angew. Chem. Int. Ed.* **57**, 3094-3098 (2018).

(4) Zhou, X., Bontemps, S. & Jordan, R. F. Base-Free Phosphine−Sulfonate Nickel Benzyl Complexes. *Organometallics* **27**, 4821-4824 (2008).

(5) Delferro, M., McInnis, J. P. & Marks, T. J. Ethylene Polymerization Characteristics of an Electron-Deficient Nickel(II) Phenoxyiminato Catalyst Modulated by Non-Innocent Intramolecular Hydrogen Bonding. *Organometallics* **29**, 5040-5049 (2010).

(6) Kenyon, P. & Mecking, S. Pentafluorosulfanyl Substituents in Polymerization Catalysis. *J. Am. Chem. Soc.* **139**, 13786-13790 (2017).

(7) Zhang, Y., Mu, H., Pan, L., Wang, X. & Li, Y. Robust Bulky [P,O] Neutral Nickel Catalysts for Copolymerization of Ethylene with Polar Vinyl Monomers. *ACS Catal.* **8**, 5963-5976 (2018).

(8) Tao, W.T., Nakano, R., Ito, S. & Nozaki, K. Copolymerization of Ethylene and Polar Monomers by Using Ni/IzQO Catalysts. *Angew. Chem. Int. Ed.* **55**, 2835-2839 (2016).
(9) Kocen, A., Brookhart, M. & Daugulis, O. A highly active Ni(II)-triadamantylphosphine catalyst for ultrahigh-molecular-weight polyethylene synthesis. *Nat. Commun.* **10**, 438 (2019).
(10) Thi V. T., Yennie H. N., & Loi H. D. Development of highly productive nickel–sodium phenoxyphosphine ethylene polymerization catalysts and their reaction temperature profiles. *Polym. Chem.* **10**, 3718-3721 (2019).
(11) Hicks, F. A. & Brookhart, M. A Highly Active Anilinotropone-Based Neutral Nickel(II) Catalyst for Ethylene Polymerization. *Organometallics* **20**, 3217-3219 (2001).
(12) Perrotin, P., McCahill, J. S. J.,Wu, G. & Scott, S. L. Linear, high molecular weight polyethylene from a discrete, mononuclear phosphinoarenesulfonate complex of nickel(II)**.** *Chem. Commun.* **47**, 6948-6950 (2011).
(13) Gao, J., Yang, B. & Chen, C. Sterics versus electronics: Imine/phosphine-oxide-based nickel catalysts for ethylene polymerization and copolymerization. *J. Catal.* **369**, 233-238 (2019).
(14) Zou, C., Dai, S. & Chen, C. Ethylene Polymerization and Copolymerization using Nickel 2-Iminopyridine-N-oxide Catalysts: Modulation of Polymer Molecular Weights and Molecular-weight Distributions. *Macromolecules* **51**, 49-56 (2018).
(15) Chen, M. & Chen, C. Rational Design of High-Performance Phosphine Sulfonate Nickel Catalysts for Ethylene Polymerization and Copolymerization with Polar Monomers. *ACS Catal.* **7**, 1308-1312 (2017).
(16) Li, M., Wang, X., Luo, Y. & Chen, C. A Second-coordination-sphere Strategy to Modulate Nickel- and Palladium-catalyzed Olefin Polymerization and Copolymerization. *Angew. Chem. Int. Ed.* **56**, 11604-11609 (2017).
(17) Tran, Q. H., Brookhart, M. & Daugulis, O. New Neutral Nickel and Palladium Sandwich Catalysts: Synthesis of Ultra-High Molecular Weight Polyethylene (UHMWPE) via Highly Controlled Polymerization and Mechanistic Studies of Chain Propagation. *J. Am. Chem. Soc.* **142**, 7198-7206 (2020).
(18) Fu, X., Zhang, L., Tanaka, R., Shiono, T. & Cai, Z. Highly Robust Nickel Catalysts Containing Anilinonaphthoquinone Ligand for Copolymerization of Ethylene and Polar Monomers. *Macromolecules* **50**, 9216-9221 (2017).
(19) Tafazolian, H.; Culver, D. B. & Conley, M. P. A Well-Defined Ni(II) α-Diimine Catalyst Supported on Sulfated Zirconia for Polymerization Catalysis. *Organometallics* **36**, 2385-2388 (2017).
(20) Liang, T., Goudari, S. & Chen, C. A Simple and Versatile Nickel Platform for the Generation of Branched High Molecular Weight Polyolefins. *Nat. Commun.* **11**, 372 (2020).
(21) Zhang, H., Zou, C., Zhao, H., Cai, Z. & Chen, C. Hydrogen-Bonding-Induced Heterogenization of Nickel and Palladium Catalysts for Copolymerization of Ethylene with Polar Monomers. *Angew. Chem. Int. Ed.* **60**, 17446 -17451 (2021).
(22) Xu, R., Liu, D., Wang, S. &Mao, B. Preparation of Spherical $MgCl_2$-Supported Late-Transition Metal Catalysts for Ethylene Polymerization. *Macromol. Chem. Phys.*

207, 779-786 (2006).
(23) Okada, M., Nakayama. Y. & Shiono, T. Heterogenization of an Anilinonaphthoquinone-Chelated Nickel Complex for Ethylene Polymerization Using Silica-Supported Modified Methylaluminoxane. *Macromol. Chem. Phys.* **215**, 1792-1796 (2014).
(24) Preishuber. P. & Brookhart, M. Highly Active Supported Nickel Diimine Catalysts for Polymerization of Ethylene. *Macromolecules* **35**, 6074-6076 (2002).
(25) AlObaidi, F., Ye, Z. & Zhu, S. Ethylene Polymerization with Silica-Supported Nickel-Diimine Catalyst: Effect of Support and Polymerization Conditions on Catalyst Activity and Polymer Properties. *Macromol. Chem. Phys.* **204**, 1653-1659 (2003).
(26) Choi, Y. & Soares, J. B. P. Synthesis of Supported Nickel Diimine Catalysts for Ethylene Slurry Polymerization. *Macromol. Chem. Phys.* **210**, 1979-1988 (2009).
(27) Ye, Z., Alsyouri, H., Zhu, S. & Lin, Y. Catalyst impregnation and ethylene polymerization with mesoporous particle supported nickel-diimine catalyst. *Polymer* **44**, 969-980 (2003).
(28) Severn. J. R., Chadwick, J. C. & Castelli, V. V. A. $MgCl_2$-Based Supports for the Immobilization and Activation of Nickel Diimine Catalysts for Polymerization of Ethylene. *Macromolecules* **37**, 6258-6259 (2004).
(29) Hu, T., Li, Y., Liu, J. & Li, Y. Syntheses and Ethylene Polymerization Behavior of Supported Salicylaldimine-Based Neutral Nickel(II) Catalysts. *Organometallics* **26**, 2609-2615 (2007).
(30) Schrekker, H. S., Kotov, V., Preishuber. P, White, P. & Brookhart, M. Efficient Slurry-Phase Homopolymerization of Ethylene to Branched Polyethylenes Using α-Diimine Nickel(II) Catalysts Covalently Linked to Silica Supports. *Macromolecules* **39**, 6341-6354 (2006).
(31) Culver, D. B., Tafazolian, H. & Conley, M. P. A Bulky Pd(II) α-Diimine Catalyst Supported on Sulfated Zirconia for the Polymerization of Ethylene and Copolymerization of Ethylene and Methyl Acrylate. *Organometallics* **37**, 1001-1006 (2018).
(32) Wucher, P., Schwaderer, J. B. & Mecking, S. Solid-Supported Single-Component Pd(II) Catalysts for Polar Monomer Insertion Copolymerization. *ACS Catal.* **4**, 2672-2679 (2014).
(33) Wan X., Zhang, Y., Wang, F., Pan, L, Wang, B. & Li. Y. Robust and Reactive Neutral Nickel Catalysts for Ethylene Polymerization and Copolymerization with a Challenging 1,1-Disubstituted Difunctional Polar Monomer. *ACS Catal.* **11**, 2902-2911 (2021).
(34) Zhang, Y., Mu, H., Pan, L., Wang, X. & Li, Y. Robust Bulky [P, O] Neutral Nickel Catalysts for Copolymerization of Ethylene with Polar Vinyl Monomers. *ACS Catal.* **8**, 5963-5976 (2018).
(35) Xin, B. S., Sato, N., Tanna, A., Oishi, Y., Konishi, Y. & Shimizu, F. Nickel Catalyzed Copolymerization of Ethylene and Alkyl Acrylates. *J. Am. Chem. Soc.* **139**, 3611-3614 (2017).
(36) Cui, L. & Jian, Z. A N-bridged strategy enables hemilabile phosphine–carbonyl palladium and nickel catalysts to mediate ethylene polymerization and

copolymerization with polar vinyl monomers. *Polym. Chem.* **11**, 6187-6193 (2020).
(37) Saki, Z.,D'Auria, I., Dall'Anese, A., Milani, B. & Pellecchia C. Copolymerization of Ethylene and Methyl Acrylate by Pyridylimino Ni(II) Catalysts Affording Hyperbranched Poly(ethylene-*co*-methyl acrylate)s with Tunable Structures of the Ester Groups. *Macromolecules* **53**, 9294-9305 (2020).
(38) Zou, C., Liao, D., Pang, W., Chen, M. & Tan, C. Versatile PNPO ligands for palladium and nickel catalyzed ethylene polymerization and copolymerization with polar monomers. *J. Catal.* **393**, 281-289 (2021).
(39) Zhang, W., Waddell, P. M., Tiedemann, M. A., Padilla, C. E., Mei, J.,Chen, L. & Carrow, B. P. Electron-Rich Metal Cations Enable Synthesis of High Molecular Weight, Linear Functional Polyethylenes. *J. Am. Chem. Soc.* **140**, 8841-8850 (2018).
(40) Contrella, N. D., Sampson, J. R. & Jordan, R. F. Copolymerization of Ethylene and Methyl Acrylate by Cationic Palladium Catalysts That Contain Phosphine-Diethyl Phosphonate Ancillary Ligands. *Organometallics* **33**, 3546-3555 (2014).
(41) Mitsushige, Y.,Yasuda, H., Carrow, B. P., Ito, S., Kobayashi, M., Tayano, T., Watanabe, Y., Okuno, Y., Hayashi, S., Kuroda, J., Okumura, Y. & Nozaki. K. Methylene-Bridged Bisphosphine Monoxide Ligands for Palladium-Catalyzed Copolymerization of Ethylene and Polar Monomers. *ACS Macro Lett.* **7**, 305-311 (2018).
(42) Mitsushige, Y., Carrow, B. P., Itoa S. & Nozaki, K. Ligand-controlled insertion regioselectivity accelerates copolymerisation of ethylene with methyl acrylate by cationic bisphosphine monoxide–palladium catalysts. *Chem. Sci.* **7**, 737 (2016).
(43) Nakano R. & Nozaki. K. Copolymerization of Propylene and Polar Monomers Using Pd/IzQO Catalysts. *J. Am. Chem. Soc.* **137**, 10934-10937 (2015).
(44) Ota, Y., Ito, S., Kuroda, J., Okumura, Y. & Nozaki, K. Quantification of the Steric Influence of Alkylphosphine–Sulfonate Ligands on Polymerization, Leading to High-Molecular-Weight Copolymers of Ethylene and Polar Monomers. *J. Am. Chem. Soc.* **136**, 11898-11901 (2014).
(45) Tao, W., Akita, S., Nakano, R., Ito, S., Hoshimoto, Y., Ogoshi, S. & Nozaki, K. Copolymerisation of ethylene with polar monomers by using palladium catalysts bearing an N-heterocyclic carbene–phosphine oxide bidentate ligand. *Chem. Commun.,* **53**, 2630 (2017).
(46) Dai, S., Sui, X. & Chen, C. Highly Robust Pd(II) α–diimine Catalysts for Slow-Chain-Walking Polymerization of Ethylene and Copolymerization with Methyl Acrylate. *Angew. Chem. Int. Ed.* **54**, 9948-9953 (2015).
(47) Sui, X., Dai, S. & Chen, C. Ethylene Polymerization and Copolymerization with Polar Monomers by Cationic Phosphine Phosphonic Amide Palladium Complexes. *ACS Catal.* **5**, 5932-5937 (2015).
(48) Xiong, S., Shoshani, M. M., Zhang, X., Spinney, H. A., Nett, A. J., Henderson, B. S., Miller, T. F. & Agapie, T. Efficient Copolymerization of Acrylate and Ethylene with Neutral P, O-Chelated Nickel Catalysts: Mechanistic Investigations of Monomer Insertion and Chelate Formation. *J. Am. Chem. Soc.* **143**, 6516-6527 (2021).

(49) Meng, J., Li, X., Ni, X. & Shen. Z. Vinyl-type homo- and copolymerization of norbornene catalyzed by bis(phenoxyimine) titanium complex. *Polymer International*, **66**, 1617-1623 (2017).
